# Plants acquired mitochondrial linear plasmids horizontally from fungi likely during the conquest of land

**DOI:** 10.1186/s13100-023-00304-7

**Published:** 2023-10-17

**Authors:** Yutong Wei, Zhen Gong, Guan-Zhu Han

**Affiliations:** College of Life Sciences, Nanjing Normal University, Nanjing, 210023 Jiangsu China

**Keywords:** Genome evolution, Horizontal gene transfer, Phylogenetics, Plant terrestrialization, Mitochondrial linear plasmids

## Abstract

Mitochondrial linear plasmids have been sporadically reported in fungi and plants. Yet, much remains obscure about the diversity, distribution, and evolution of mitochondrial linear plasmids. Here, through phylogenomic analyses across 7,163 cellular organisms (including 991 plants), we find that mitochondrial linear plasmids are widely present in land plants and fungi. Phylogenetic analyses indicate that plants are likely to have acquired mitochondrial linear plasmids horizontally from fungi before or during the conquest of terrestrial environments by plants. Gene content analyses show that mitochondrial linear plasmids harbor a highly dynamic and promiscuous repertoire of genes. Our study refines the understanding of the origin and evolution of mitochondrial linear plasmids.

**Supplementary Information:**

The online version contains supplementary material available at 10.1186/s13100-023-00304-7.

## Main text

Transposable elements (TEs) comprise a major proportion of the plant genomes, contributing substantially to the evolution of their genome complexity. Among diverse TEs, Polintons (also known as Mavericks) are unusual large DNA transposons that have been thought to be present widely in the genomes of animals and protists [1–3]. Typically, the Polinton genomes are flanked by terminal inverted repeats (TIRs) and encode several conserved proteins, including protein-primed family-B DNA polymerase (pPolB), retroviral-like (RVE) integrase, DNA packaging ATPase, and maturation protease [3]. Moreover, many Polintons encode homologs of viral capsid proteins, indicating that they might produce virions and represent *bona fide* viruses [4]. Therefore, Polintons have been proposed to possess dual lifestyles as both transposons and viruses [3]. Interestingly, Polintons share a set of conserved genes with a variety of mobile genetic elements that pertain to viruses, virophages, TEs, or plasmids, forming a highly complex evolutionary network [3, 5]. Polintons, adenoviruses, bidnaviruses, virophages, transpovirons, cytoplasmic linear plasmids, mitochondrial linear plasmids, and diverse phages (e.g., tectiviruses) encode a common hallmark protein, namely pPolB [3]. For convenience, we use Polinton-like mobile genetic elements (Polin-MEs) to refer to these pPolB-encoding mobile genetic elements.

Among Polin-MEs, mitochondrial linear plasmids have been sporadically reported in fungi and plants, but their origins remain largely mysterious [6, 7]. Here, through phylogenomic analyses across 7,163 eukaryotes, we found that plants are likely to have acquired mitochondrial linear plasmids horizontally from fungi at least before or during the conquest of terrestrial environments. Our phylogenomic analyses provide insights into the origin, evolution, and diversity of mitochondrial linear plasmids in plants.

### Distribution of mitochondrial linear plasmids in plants

**Fig. 1:**
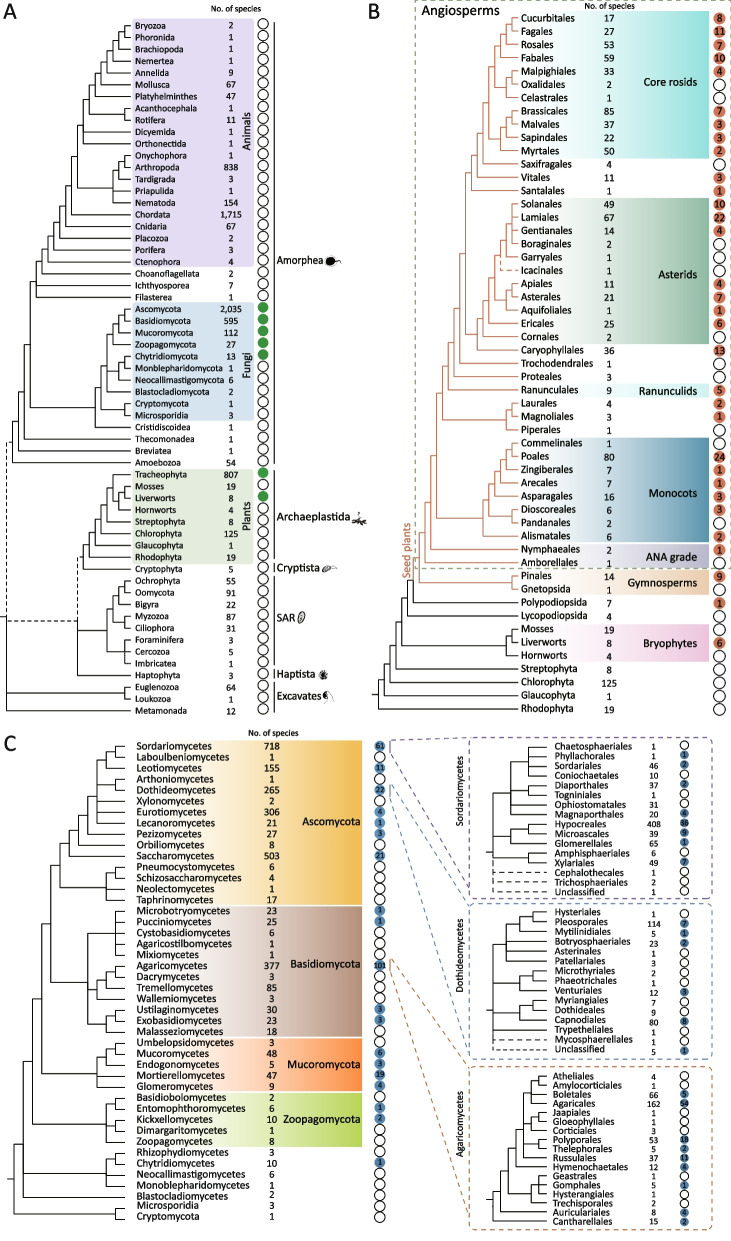
Distribution of the mitochondrial linear plasmids in eukaryotes. **A** Distribution of the mitochondrial linear plasmids in major eukaryote groups. Numbers indicate the numbers of species used. Closed and open green circles indicate the presence and absence of the mitochondrial linear plasmids in the corresponding eukaryote groups, respectively. **B** Distribution of mitochondrial linear plasmids in plants. Numbers near plants indicate the numbers of species used. Closed and open orange circles indicate the presence and absence of the mitochondrial linear plasmids in the corresponding plant groups, respectively. Numbers in the circles represent the number of species with mitochondrial linear plasmids. **C** Distribution of mitochondrial linear plasmids in fungi. Numbers near fungi indicate the numbers of species used. Closed and open blue circles indicate the presence and absence of the mitochondrial linear plasmids in the corresponding fungal groups, respectively. Numbers in the circles represent the number of species with mitochondrial linear plasmids

First, to explore the distribution of the mitochondrial linear plasmids in plants, we used a combined similarity search and phylogenetic analysis approach to identify mitochondrial linear plasmids in a total of 991 representative plant species (including nuclear genomes and mitochondrial genomes) (Table S1). These plant species cover the major diversity of plants, including 781 angiosperms, 15 gymnosperms, 4 hornworts, 8 liverworts, 19 mosses, 125 chlorophytes, 1 glaucophyte, and 19 rhodophytes (Table S1). Mitochondrial linear plasmids were identified in nuclear genomes and/or mitochondrial genomes of a limited range of green plants, including angiosperms, gymnosperms, ferns, and liverworts (Fig. 1B), indicating that mitochondrial linear plasmids are likely to be only present in land plants.

### Horizontal transfer of mitochondrial linear plasmids from fungi to plants

**Fig. 2:**
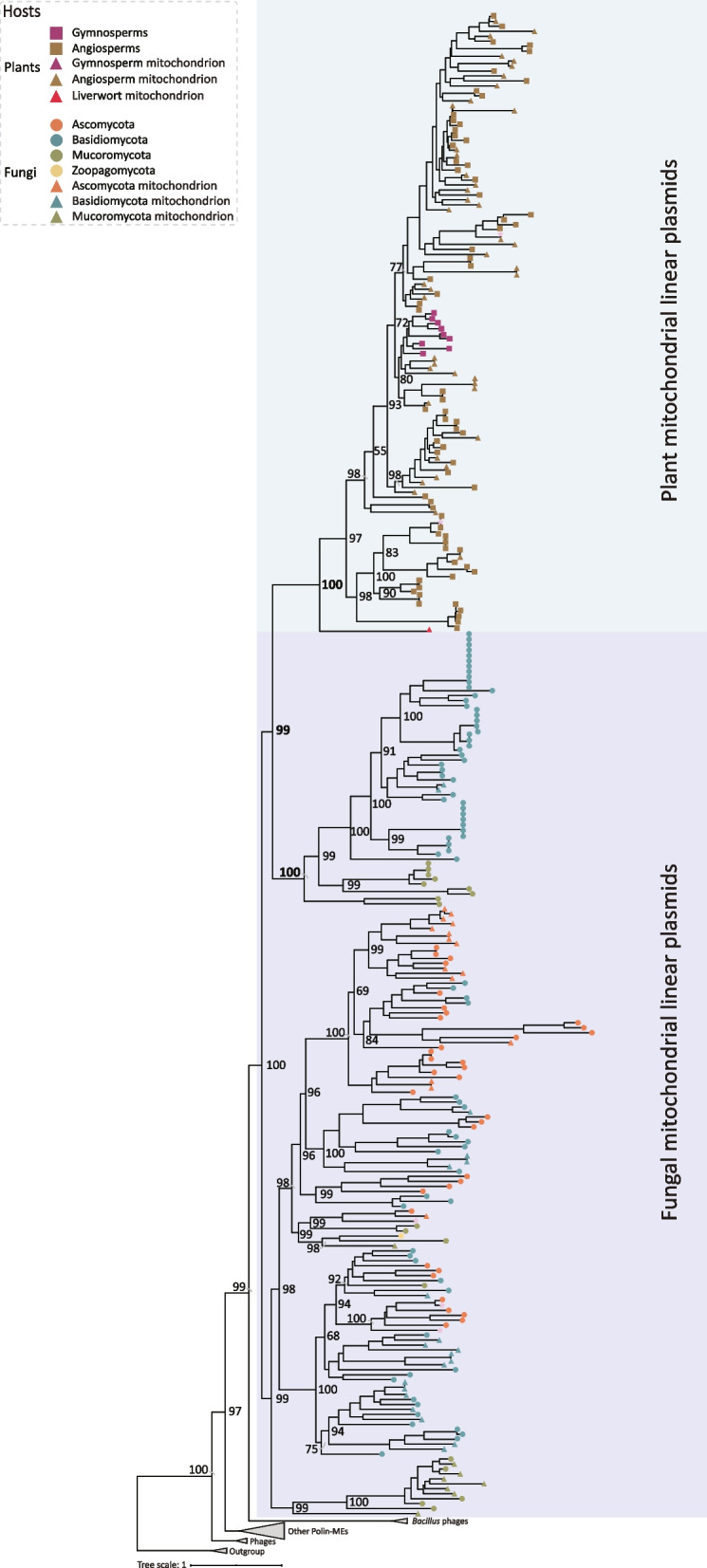
Phylogenetic relationship among representative mitochondrial linear plasmids from plants and fungi. Mitochondrial linear plasmids from fungal and plant nuclear genomes are labeled with circles and rectangles, and are highlighted in purple and blue, respectively. Mitochondrial linear plasmids were identified in mitochondrial genomes are labeled with triangles. Representative pPolB proteins are labeled in pink stars. Branch support values are given near the selected nodes

To explore the potential source of plant mitochondrial linear plasmids, we used the combined similarity search and phylogenetic analysis approach to identify mitochondrial linear plasmids in the genomes of 6,172 eukaryotes, including 77 Excavata species, 5,792 Amorphea species, 3 Haptista species, 295 SAR (Stramenopila, Alveolata, and Rhizaria) species, and 5 Cryptista species (Fig. 1A) (Table S1). Mitochondrial linear plasmids were identified in nuclear genomes and/or mitochondrial genomes of a large number of fungi, suggesting that horizontal transfer(s) of mitochondrial linear plasmids might have taken place between fungi and plants (Fig. 1A). Different from fungal retroplasmids that replicate via reverse transcription and encode reverse transcriptases, mitochondrial linear plasmids we identified here encode pPolB proteins. To further explore the evolutionary relationships of mitochondrial linear plasmids from plants and fungi, we performed phylogenetic analyses based on pPolB, a hallmark protein for analyzing the relationship of Polin-MEs [3]. Phylogenetic analyses of pPolB proteins clearly show that mitochondrial linear plasmids from land plants cluster together (UBoot = 100%) and nest within the diversity of the plasmids from fungi with robust support (Fig. 2 and Fig. S1). It should be noted that most of the mitochondrial linear plasmid sequences from liverworts and ferns were too short to be used for reliable phylogenetic analyses, and we used only one pPolB protein from the mitochondrial genome of *Marchantia paleacea* (Supplemental Data Set 1). Nevertheless, our phylogenomic analyses support that plants are likely to have acquired mitochondrial linear plasmids horizontally from fungi.

We then closely interrogated the distribution of mitochondrial linear plasmids in fungi, and found that they are widely distributed in the genomes of terrestrial fungi, including Zygomycota, Mucoromycota, Basidiomycota, and Ascomycota (Fig. 1C). Interestingly, fungal plasmids that are closely related to plant plasmids are from species of Mucoromycota (Mortierellales, Mucorales, and Endogonales) and Basidiomycota (Microbotryales, Agaricales, Polyporales, and Russulales) (Fig. 1C), many of which can establish symbiosis with diverse plants, such as species within Agaricales, Russulales, and Endogonales [8, 9]. Taken together, we hypothesize that plants might have acquired mitochondrial linear plasmids horizontally from fungi probably through symbioses such as mycorrhizal symbiosis.

### Frequent host switching of mitochondrial linear plasmids in plants

We observed that the phylogeny of mitochondrial linear plasmids (based on the pPolB proteins) is generally incongruent with the phylogeny of their plant hosts. Moreover, we compared the phylogeny of mitochondrial linear plasmids with that of their plant hosts. Indeed, we found no statistically significant congruence in phylogenies between plant mitochondrial linear plasmids and their hosts (Fig. S2, Table S2). These results suggest that plant mitochondrial linear plasmids might have undergone frequent host switching.

### Dynamic evolution of mitochondrial linear plasmid genome architectures

**Fig. 3:**
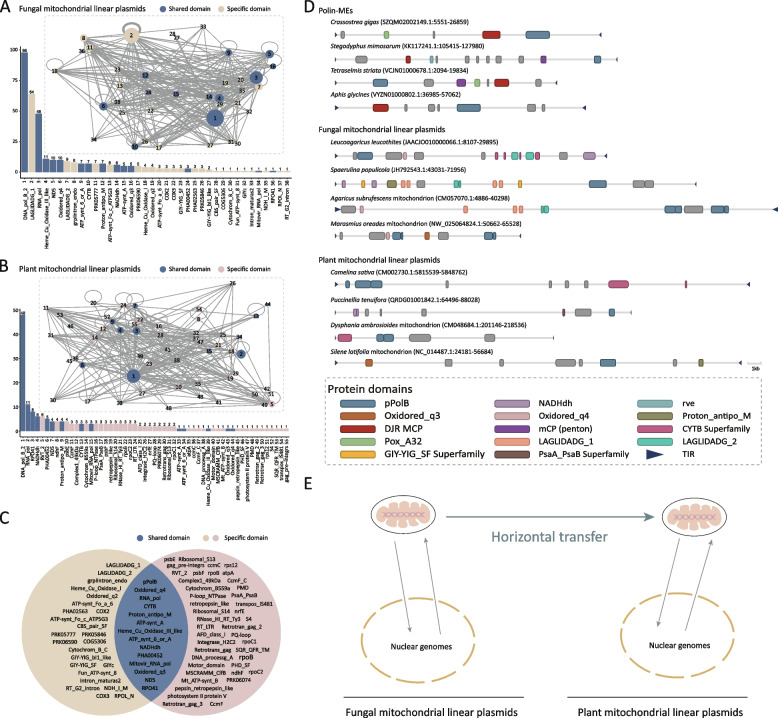
Origin of the mitochondrial linear plasmids in seed plants. **A** Annotated protein domain architectures of complete mitochondrial linear plasmids from fungi. **B** Annotated protein domain architectures of complete mitochondrial linear plasmids from plants. Protein domains shared by fungal and plant mitochondrial linear plasmids are labeled in dark blue. Fungal and plant specific protein domains are labeled in beige and pink, respectively. Charts indicate the occurrence frequency of different protein domains in the corresponding groups. The circle size is proportional to the occurrence frequency of protein domains. Numbers near the circles represent domain names (important domains are specified) and are consistent with the numbers near the protein domain names in the charts. The thickness of lines linking two circles is proportional to the frequency at which the two protein domains appear in a complete element. **C** Venn diagram of annotated protein domains in fungal and plant mitochondrial linear plasmids. Protein domains shared by fungal and plant mitochondrial linear plasmids are highlighted in dark blue. Fungal and plant specific protein domains are highlighted in beige and pink, respectively. **D** Genome maps of representative Polin-MEs, fungal mitochondrial linear plasmids and plant mitochondrial linear plasmids. **E** Hypothetical scenarios for the origins and evolution of plant mitochondrial linear plasmids

To explore the diversity and evolution of gene contents within mitochondrial linear plasmids, we identified a total of 95 complete elements characterized by the presence of two flanking TIRs from plants and fungi, and annotated their protein domain architectures. Unlike canonical Polintons, mitochondrial linear plasmids of fungi or plants do not encode viral capsid proteins (MCP or Penton), which is consistent with their nature as plasmids (Fig. 3D). As expected, the hallmark protein domain, pPolB, was most frequently identified. Most mitochondrial linear plasmids encode pPolB and RNA_pol (accession: cl44477), which indicates that they are capable of autonomous replication and transcription. Some domains are related to various transposable elements, and these domains might be derived from transposable element insertions rather than integral components of mitochondrial linear plasmids. Many host-derived domains also appear in mitochondrial linear plasmids but with relatively low frequency, such as mitochondrion-encoding genes and PsaA_PsaB-encoding genes (accession: cl08224) (Fig. 3A-C). Given the frequent movement between nuclear genomes and mitochondrial genomes observed for the plasmids (Fig. 1A), we suspect that mitochondrial linear plasmids might act as gene transfer agents which can transfer genes between nuclear genomes and mitochondrial genomes (Fig. 3E).

Domains related to homing endonucleases, such as GIY-YIG (accession: PF01541), PDDExK (accession: cl40440), LAGLIDADG (accession: PF00961), and HNH (accession: PF01844), were also frequently identified in mitochondrial linear plasmids (Fig. 3A-C). Homing endonuclease genes are selfish genetic elements that mobilize themselves through generating double strand breaks at specific target sites and getting copied across to the broken sites [10]. Therefore, we suspect that homing endonuclease domains might help drive the rapid spread of mitochondrial linear plasmids into related target sites within the host genome.

### On the origin of mitochondrial linear plasmids in plants

In this study, we found that mitochondrial linear plasmids are widely present in the nuclear and/or mitochondrial genomes of land plants, and the distribution indicates that plants are likely to have acquired mitochondrial linear plasmids before or during the conquest of terrestrial environments by plants. Mitochondrial linear plasmids were identified in the genomes of terrestrial fungi (including Zygomycota, Mucoromycota, Basidiomycota, and Ascomycota), suggesting that mitochondrial linear plasmids might have emerged in fungi during their early transition to land in the Cryogenian period (~ 720 million years ago) [11]. However, these timescales should be taken with caution, because plants or fungi might have acquired mitochondrial plasmids later than estimated above (but spread to earlier-branching lineages through host switching) or earlier (but lost in earlier-branching lineages). Nevertheless, our analyses do show that plants might have acquired mitochondrial linear plasmids horizontally from fungi through the ancient symbiosis between plants and fungi.

## Material and methods

### Identification of mitochondrial linear plasmids in plants

We used a combined similarity search and phylogenetic analysis approach to identify mitochondrial linear plasmids based on the hallmark protein pPolB within the genomes of 991 plants [12, 13]. All the genomes of plants were retrieved from NCBI genome resources (Table S1). First, we used the tBLASTn algorithm to search plant genomes using various complete and truncated pPolb proteins (Supplemental Data Set 2) as queries with an *e* cut-off value of 10^–5^. Then, we performed initial large-scale phylogenetic analyses of pPolB homologs from plants and representative pPolB proteins from Polin-MEs. Protein sequences were aligned using MAFFT 7.450 [14]. Large-scale phylogenetic analyses were performed using an approximate maximum likelihood method implemented in FastTree 2.1.10 [15].

### Identification of mitochondrial linear plasmids in eukaryotes

To explore the potential source of the mitochondrial linear plasmids in plants, we used the aforementioned combined similarity search and phylogenetic analysis approach to screen the mitochondrial linear plasmids that are closely related to plant mitochondrial linear plasmids in a total of 6,172 eukaryotes. All the genomes of eukaryotes were retrieved from NCBI genome resources (Table S1).

### Phylogenetic analysis

To explore the evolutionary relationships of the mitochondrial linear plasmids, we performed phylogenetic analyses using pPolB proteins of mitochondrial linear plasmids, some phages and Polin-MEs. The pPolB proteins were aligned using MAFFT 7.450 [14]. Phylogenetic analysis was performed using a maximum likelihood (ML) method implemented in IQ-tree 2 [16]. ModelFinder implemented in IQ-tree 2 was used to determine the best-fitting substitution model [16]. The node supports were evaluated using an ultrafast bootstrap method with 1,000 replicates [17]. Phylogenetic trees were annotated using iTOL [18].

### Phylogeny congruence analysis

The phylogeny of plant mitochondrial linear plasmids was compared with that of their hosts using Jane 4 [19]. Different sets of cost values for five types of events (for cospeciation, duplication, duplication with host switch, loss, and failure to diverge: 0, 1, 2, 1, 1; –1, 0, 0, 0, 0; and 0, 1, 1, 2, 0) were examined [20]. The statistical analyses were performed using the method of random parasite tree with the sample size of 500.

### Domain architecture annotation

To identify complete mitochondrial linear plasmids, we used gt tirvish implemented in GenomeTools to identify the flanking TIRs [21]. We identified a total of 95 complete elements characterized by the presence of two flanking TIRs (Table S3). Complete mitochondrial linear plasmids with flanking TIRs were retrieved and annotated using various domain search or similarity search tools, including Conserved Domain search [22], HMMER [23, 24], tBLASTn, and BLASTp.

### Supplementary Information

**Additional file 1: Figure S1.** Phylogenetic relationship among representative mitochondrial linear plasmids from plants and fungi with all taxon names. **Figure S2.** Comparison of the phylogenies between plant mitochondrial linear plasmids and their hosts. The left is the phylogeny of plant mitochondrial linear plasmids based on the pPolB proteins, whereas the right is the host tree based on the plant tree of life. **Dataset S1. **Alignment of pPolB encoded by liverworts and ferns. **Dataset S2.** Representative pPolB proteins used in this study. **Table S1.** Information on specie genomes used in this study. **Table S2.** Phylogeny congruence test for plant mitochondrial linear plasmids. **Table S3.** Information on fungus and plant genomes with the presence of mitochondrial linear plasmids.

## Data Availability

Data sharing is not applicable to this article as no datasets were generated during the current study.

## Abbreviations

TEs: Transposable elements
TIRs: Terminal inverted repeats
pPolB: Protein-primed family-B DNA polymerase
RVE: Retroviral-like endonuclease
Polin-MEs: Polinton-like mobile genetic elements
ML: Maximum likelihood
