# Supplementary material for: Plants acquired mitochondrial linear plasmids horizontally from fungi likely during the conquest of land
Source: Mob DNA. 2023 Oct 17;14:15. doi: 10.1186/s13100-023-00304-7 (PMC10583447; doi:10.1186/s13100-023-00304-7)
Supplement: Supplementary file 1 — Additional file 1: Figure S1. Phylogenetic relationship among representative mitochondrial linear plasmids from plants and fungi with all taxon names. Figure S2. Comparison of the phylogenies between plant mitochondrial linear plasmids and their hosts. The left is the phylogeny of plant mitochondrial linear plasmids based on the pPolB proteins, whereas the right is the host tree based on the plant tree of life. Dataset S1. Alignment of pPolB encoded by liverworts and ferns. Dataset S2. Representative pPolB proteins used in this study. Table S1. Information on specie genomes used in this study. Table S2. Phylogeny congruence test for plant mitochondrial linear plasmids. Table S3. Information on fungus and plant genomes with the presence of mitochondrial linear plasmids. [file 13100_2023_304_MOESM1_ESM.pdf]

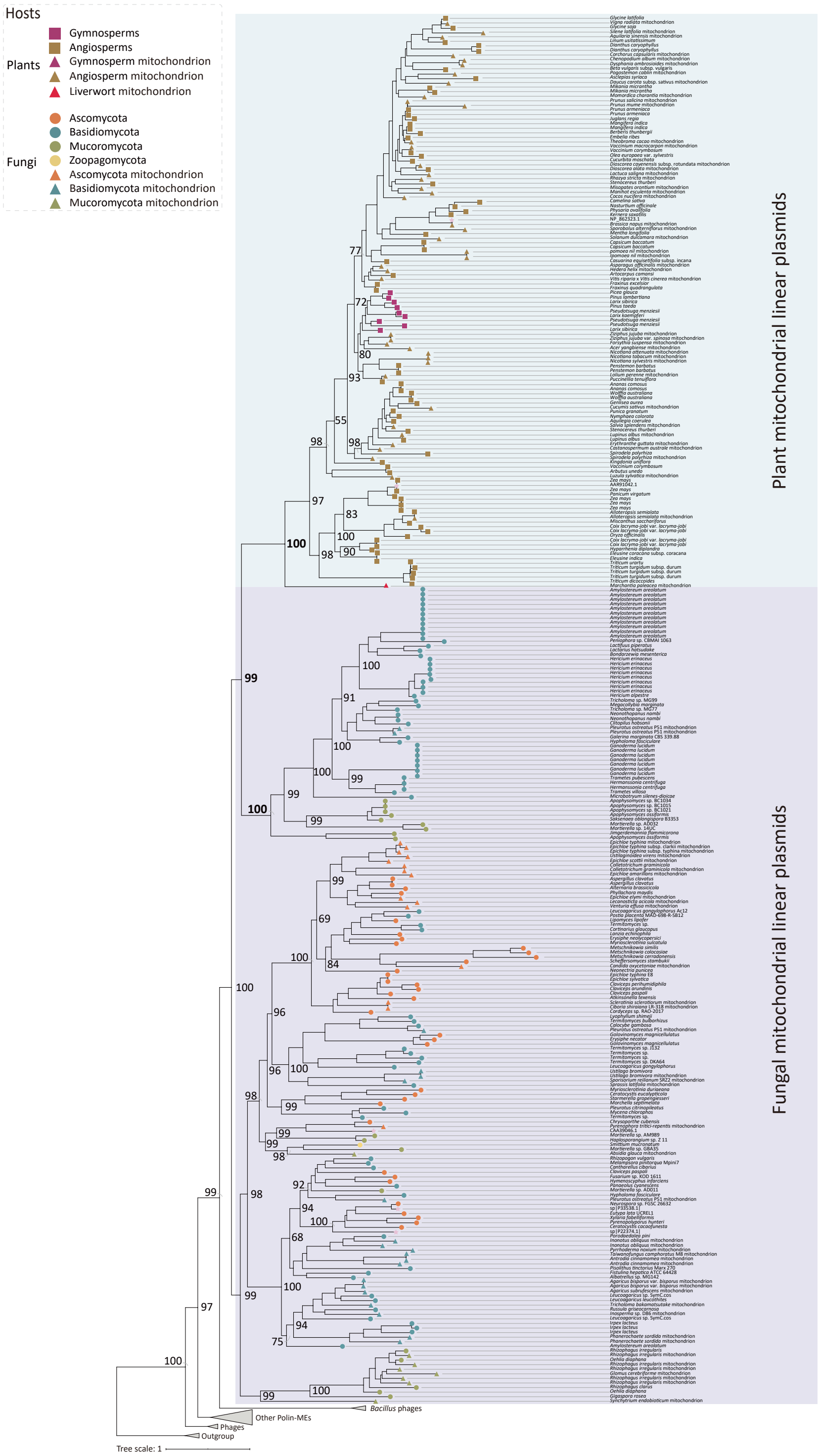

Figure S1. Phylogenetic relationship among representative mitochondrial linear plasmids from plants and fungi with all taxon names.

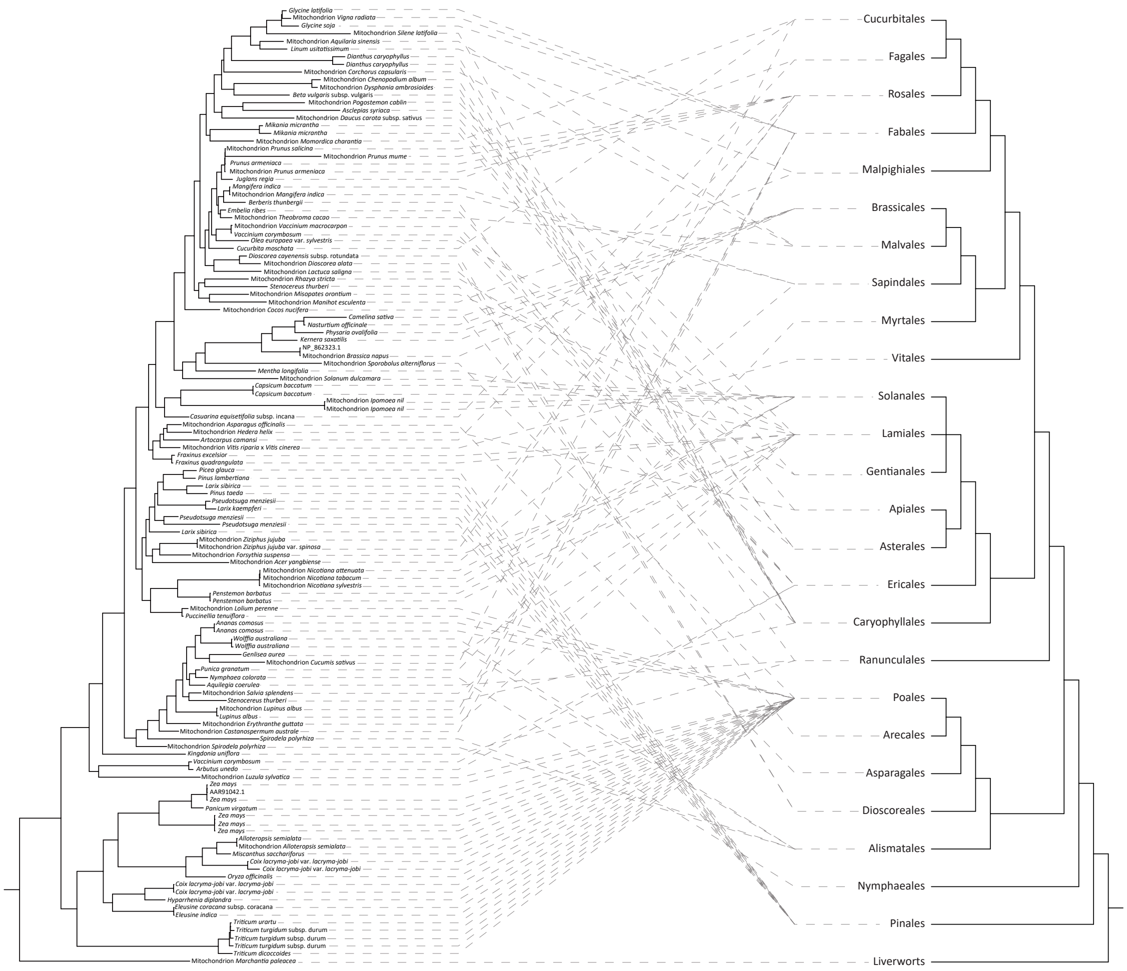

Figure S2. Comparison of the phylogenies between plant mitochondrial linear plasmids and their hosts. The left is the phylogeny of plant mitochondrial linear plasmids based on the pPolB proteins, whereas the right is the host tree based on the plant tree of life.

Dataset S1. Alignment of pPolB encoded by liverworts and ferns

>plant_Liverworts_mitochondrion_Wiesnerella_denudata_NC_053538.1_9.89e-10_89027_89197_239_295

GKALEAQFIRKAFYGGHTDVYKPYGENLYVVYDSNSLYPFSM-TKKMP-----------GGK----------PK-
TDDRS--

>plant_Liverworts_mitochondrion_Monosolenium_tenerumMK230931.1_4.89e-08_89615_89734_238_276

LGKALEAQFLRKAFYGGHTDVYKPYGENLYVVYDSNSLYPF--

>plant_Liverworts_NC_001660.1_2.87e-11_89292_89630_238_360_Marchantia_paleacea

----

LGKTLEAQFLRKAFYGGHTDVYKPYGENLYVVYDSNSLYPFSM-
TKKCPVVSGPMIEASTKGKNVFFEANIECPL-DFNRPF-------------------------------
LPFRLPQLKYLIFPTGNWKGTYYSKKLKYAKSLG---

>plant_Liverworts_mitochondrion_Conocephalum_conicum_MK230928.1_2.82e-08_88124_88246_239_278

GKALEAQFLRKPFYGGHTDVYKPYDENLYVVYDSNSLYPFSM--

>plant_Liverworts_mitochondrion_Pleurozia_purpurea_NC_013444.1_6.13e-06_81023_81157_240_285

---EGLDP-
FLRTAFYGGHTDVYKPYGDNLYVYDSNSLDPFSI-TKKMP-----------A--

>plant_Liverworts_mitochondrion_Treubia_lacunosa_NC_016122.1_6.41e-07_75802_75951_243_293

DQLMRNGFYGGHTDVYKPYDENLYVYDSDSLHPFPM-TKKMP-----------DGK----------PKWTDD--
----

>WP_014905375.1

-
MTTTNRKKRREIKLFTLDTETRGLDGDVFRIGLFDGKQYYTGYTFADVLPVFEKYKAYDCHVYI
HNLDFDLSKIIAELRDYAEPTFNNSLFINGNIVTFTASHIILHDSFRLLPSSLENLCRDFDLLEGGKM
DIVDYMEENNYGIYNVKNRKLNKRLTKGNFFTTVDKDDPVLCEYMEYDCRSLYKILEIVIGLSK
LEVEQFINCPTTASLAKTVYKEQYKKDYKVAISTKQYNHKQIGKGLEA-
FIRKGYYGGRTEVFTPRIENGYHYDKNSLYPYVMKMAEMP-----------VGY----------
PNVLDNEEAELSFDLWKRRKYGAGFIHAKVHVPEDMYIPILPKKDYTGK-
LIFPVGKIEGVWTFPELALAEAEGCRIEKIESGVVFEKTAPVFREFISYFEEIKNTSKGAKRAFSKL
MQNALYGKFAMQRERIMYADISERDKLEAEGHTVSEIIYDMNGIRMEFLEYDGYAKAEYIQPHIS
AYITSIARILLFKGLKYAHEKGILAYCDTDSCATTTKFPDKMVHDKEYGKWKLEGYVIEGLYFQP
KMYAEKAINTDGEYEEVLRMKGVPKWVVEEQLDYNTFKKWYLQVKRGKAE-
IPIYKGGERVQKFLTKSKNNIEMNELAEMHKTINFAREQKRNIDLNKNTTSPLVRNDYGENKDEK
SEYEFDEWYERLEEFNDDMNAVEELCMKFGKIQIPEKKQRKLYGLYKEYSSKAKAMCFSNEGLP
IQDWCKKTGWDMKELLGELSFL--

>WP_000039472.1

MSKKTKKERQKSAKLLTLDTETRGLTGDVFRVGLFDGENYYVANTFDEILNIFDQYKNYECHVY
VHNLDFDLGKIATTLFTRDRVCFAKSIFINGNVVTLHADSMILHDSLRLLPGTLEKLCKDFGLTDN

AKKDLSDIMKEQGYAIYNA-DGSFNKRKSLGNYFENVPADDPTLNEYLEYDCRSLHEILSIVMDIAGIDLETLVKCPTTASLSMRVFKEQYSEDYD-KVATHWYT-GEWGRFLED-HVRLSYYGGRTEVFTPHLQNGYHLDVNSLYPYVMKIAKFP-----------VGY----------PNLLKDGQAEKKWRHWKRRAVGGGVMWCRVDVPEDMYLPVLPKRDPSGK-LLFPVGKLEGVWTLPELLEAERNGCTIEAVYQMVYWEQMEYIFKDFVENFEVLKKTSEGAKKIFAKLIQNALYGKFGMQRKRTSYGDMRDIHDLQEKGIEYRVHKHTNNGIEMEFTEYLSESKAMYIQPHIASYVTAYARILLYRALKEQHEKGVIGYCDTDSIACESMMDANMIDKEEYGKWDLEGVIKEGIFLQPKFYAERHENG----KEVIRAKGIPRDKMEE-ISFDNYKEWLEIMKEGEQERIHIFDGHQARKKFSTTLKADEHFDTQREMKKSINLLLEQKRIIDYKNNVTRPHARYDYGPKKDEMNYKDYQEYEKKLNDMYDDVDDIKELINDLGYIKCMSKGD-MYYEEYKQFPRSVRSKYFRKTGIPIDVWSEEAGWELNDLLEEFRLMGV

>plant_mitochondrion_Azolla_filiculoides_MN400566.1_2.75e-06_24856_24695_312_364

--TFIE--FV--------SEIYKERQEA---KKRGDMVTSSLHKLTMNSLYGRFGIKPE--------------------------------MNITE--ICDQE---

Dataset S2. Representative pPolB proteins used in this study

>CAD60788.1

MATKRCLDSDGNEQYRTVRFRGDSNDGHHNQQNTTETLRHLIQTVEDMDRPRSTTDALIELLNS
VQSMSDNQPEPSFDLNVFLSSPQNSDVSEDNQRELCFDPDQFLNMSQNIDACDEWSTTDFDMDH
FLNTSQSSVSEQNSKSDQDQIVLSCQTGEGSDTRPYQTFNPTAANMGLSQDEIETLVRQGGSVHK
YTVIPRARFNGLEIHRRINLREISSTDLADYTTFLHELLSEIVSFSRLLAGDSGVINITLRGESLTSDV
NTLLSPRNNYDLDLFINQLEKIMQSNSEVQTDQALDLCVSIATCKNGGGRRKIQDLAHDEVIRRN
KMNLFCPTNITNNLCFTICLAHFLNPQKSHSELEAIAANMQTTAGHKVQYKIAFDDIARFEQMLN
IKIVTFYRSNTGMLEKYTTTDKPHSKTAYLYLHDGHYFLIKNLTAFIGTPYVCEYCHVGYTCRRN
HRCEYVCDVCNYPDCHTQTKQIIQCNDCLRFCRSRYCFEMHKQPPPGQQFAQCDVTKYCKMCN
RRYYISGSKPKPHRCEAEYCVHCGESLATGGEHECFIQPYKPMEPSDRYIFYDFKTRFENGKHVA
NFVCAITFNGEEFVAEGVDCVDRLIKKFRRPKYRNYTWVAHNASGFDNFILLEYFVRMGIPPKIT
MRGCRLIFMFDRTYKQRFLDSYLFMPMRLSKTPEAFQLDNIEKGYFPHVFNRAENDCYIGPYPEK
HFYGYETMSDKERSDFDAWYCTVSGKVFDFKKELAKYGKNDVVLLRKACIKYRHEFILCTDLD
PFTFTTLAGCCMGVYKSKFLPKDTLALTRNDGYTNQNKTYSNASIEWLEYVAKSRSIEIQHALNG
GEVSFGKYHVDGFYDDGRVKKAFDFLGCFYHGCDRCYNPYDINPLSKLSYGVLRRQVNDRCEIL
QRMYGLQVEFMWECDWKAAKNSDVGVMDFMSTYKRPERLKPRDALFGGRVNAFKLYHKTSD
DEKVFYRDITSLYPFVQSTKSYPVGHPKIIFKDFENLENYFGFIKAEVIPPRGLFHPVLPYRTGGRL
MFPLCRSCAEEQNQDTPCQHADKERALTGCWVSVELSKAVERGYVVSEVYEVWHFSQRSDTLF
SDYVKTFLQFKQESSGFPEDVVTDEEKESYVKDYFEKEGVKLNIDNITFNAARRSINKQILNSLW
GRFSLKSQLPSSELISDPEQFARHIFAKGHDINYFSFLSDSIALVQYTQPYGKGKSTRDTNVFLGAF
TTCYGRLELYDLLEKLGERVLYCDTDSVIYTYRDGEYDPPLGPYLGELTDELDGGDYINIFASSG
PKSYGYSTAKGKVCMKAKGITLNTVNSQAIRLDTLIGLVDDYVSGRDNSRHVLAHTDTIVRNKT
QFTLHNKALVKKFKVVYTKRVLLPDYSTVPYGF

>XP_002933046.1

MVGGGRLEKFPKLSVNRTKTINRAVKMLISARASLKARAHRRLCCNNANLHATTSTSLPIQSGV
LKTHDTPHKSDRLQIQATQQNSGRSNVQDSDQRLCLEAVNADHDQNTGVYLDAIYHQQRDLAN
FNGVMYIDHFRFINLDRIHSFVDAVNAVHSSIQNLLNRTLPDIAPGDFVQLRLEGGNAFDPVYSTK
QSSEAFNADTFLNCIANALQSNAECLAGNSLKLVVVVIRNRRGGVKKRLRAIPYSKIISGKKQWL
YDFNNYTTNLCLAASLYALMDNDDVGDAVLLERAKQLHRVLDIPEDQLVSFNDIAEFENYLNV
NIKILYFSQGRWQFYHTGAASREKILFVLHHENHYYGIKNVKSFIGESYFCERCNSVYHHKNNHG
CQQFCKACHRMDCRDEIGIQPRCFNCRVFCRSKDCLELHRQLALDDESICRLKTFCDSCYRYVCN
GDEHKCGGLRCSVCGVRVGKFDTHICYMQKCKAQKRCEKYIIYDFECMQETGTHIPNYIYAANL
HGSPAWEFEGNDCVQKFVQFFTSGVFDHYTFIAHNAGRYDSYFIVQELIREKLQIQIINQGGKLLC
VTLPDLKMRFIDSLNFLPMKLSKLPEAMGFSGSKGYFPHFFNTEQNQNYIGPMPSIKFYGTDYMM
PGEKNEFMTWYTEHKDDTFNFQKELKAYCKQDVEVLRKACECYRDRIMAMTKKKRTYYCKRK
KRRVVVRRSIDPFQLVTLASVCMAMYRFKFIPLNTIAIVPGDNYHKTQKRFSTPAIQWLLYVAHT
ENIPIQHALRGGERRVGRYFLDGYAFVDGKHVAFEFQGCFYHGCPVCYNEADSNDVTNSTYGQ

LYYTFLVKKRYLQECGFIIRLMWEHEWHEMLEKDERLKEFIHKMQFPIPLDPRDALYGGRTNAI
KLYHKVEDGENINYYDFTSLYPFVNKTKTYPVGHPKIIYENFGYIKKYFGLAKVKVYPPRDLFFP
VLPMKLNKKLMFPLCYTCALNCQAELCTHSDEQRSLTGTWTTMELEVAIEKGYRIAQIYEIWHF
DNSSNDLFTQYINLHLRDKQEASGYPNWCTDAAKKKQYIAAFYEKEGIQLRADKIAVNPTKRQI
SKLFLNSLWGKFGQRSNLPHTSIVTDPDELFKLAFLPYYELSEVNFINDETAAVNWKYSKERYTI
NKNTNIFIACFTTAYARLELYKLLDRLQERCLYHDTDSVIFVSKEGDWNPPLGDYLGELTSEVPN
NTHITEFVSAGPKTYGYRLNTGKTTLKVKGITLNVANTQVINFDSLKDLVLDYPHNTDVKTQKTI
GTEQSGIVRNKKRWQIETRTLRKTQKCVYTKRQLSNDFTTLPFGY

>ETO19856.1

MSSQTPLESATNLLLNRTSAEISDLNVHFSYQFVKVGDVMFGHEFDDYFEIENIVRITSSFGSYRR
DKSPPINKSDDVHKYFLKALEYIPDEHIINSFMVVVNVELQSYFSVDQVDSMTNVLSFFFKDVKT
KDKESFRHSIFSKNFNENVRSVTSSSFHSINSFQVISIKQKPEAKKRRGRPKSKKTKASVHKTRSSS
PMFEGGCRLKNDKTQVFRYLNGKVKVIFPKQKKDDNNCALYSFCRALSHEFNNVSRDKRKTLT
RGRIFNAMKLRALLEIPENDLIKFSHLQRITEIALEKYEIDVGIKVYIVADQKLQLQFQTTSSAPETP
EKKWVSLLHTFPVENHYGAILSITEKEKKVKCKHCRINIFESEEHECDPEIVTRVQSYFRRSRRPIT
APSYVKDYGKICEKEVLPSKDWPLKRSNYSHKWQDFGVIDVETFMDKTPGDADWKEGTIMFHR
PYAVGFLFRNVFRCFKGPNCVDEFLKEFGEQEITVCGFNNGRYDNYFFLRNYLRNRKNKSVQFII
DTARVMCLRVGKLKFVDLAGFIPSSLDSACKSFGVPQTIAKGKFSHQLITSFEDVDCYENVVEVD
DNVMMNVMKFARSKRTYEEWEKMNKIEILAMCIQEFKEFKSFRWGVHDNAEKLSEMHRSLMD
FNQDAHEVFPNPWKKYLENDCRCTFWLACKLQDTFAEVLKNNGKVPWIFDSPTISSFAYLIWRY
SLRESRLAAHAIEIPNCELKYNFIRAAMYGGAVTNFFRNYQSVDSHLPFDQIKDSLVYLDVNSLY
PSAQMGGFNLLHAGEDEEREFIPLYPVGPSFWSDNAEADYCNDVMGFYFVEVFPPDDLLIPIIPQR
KKEFQMGYVKENDLRTWTSSGLIRSLLPFKGIYSSVLLKLGEKFGYKYKFQSEALVYKECIAGLF
DCARKIHKMKNAEDEKKDRGEPFNPAMRLTTKLVLNAGYGQQCMKTRKDKKVVISSIMEMMN
YLEKGNIVKFSLFTSGEKIKKIIALFQDSKFDNTRPTQNGVLILDYSKCIYFSHLYRICGRKREDGT
LKLFHEYVMAYGDTDSAIIHADYAVGMEGKGLGQMVNEKPDCKIICFRSNGPKSYLLTTINAKG
EVKYEGFRFKGIPRSCWVRKDSKNDHRFDPTKDKVFDEKTVVSFSNNFKRNLNIKGGNQFLTFT
SVESTRTIDPTQFKSMRYDEDYKVWFPFGYNMNKVDSEAIRKHGPSHHANLDFEDVRDLVRLFT
PEPIEIASSLPGSLEHDKMDNGDDDLILEDLEKQFPHLKAKRERKEFNEAEEKAHVTLGDELPERF
K

>POLB-2_TCp_gi270012658

IVFHNLTNYDSHLFIKELEINLGKKEKYIFSCLHLRFIDSFFLASLEKLAAALDDLKGFPYSFIDDLT
ELPIDFYDDDYTQTLEYDLYLKSDILLLADVFNFRNCKYELDPAHYTAPSLSWAMLKTKLTDDM
LHFFKKGIRGGVSCSYILYLDATNLYGWAMSE-
LPLGFTLQDNGYVLEVDISYPHNSPFLPNCAKLIPNCAKNIIHNLKQAQNGLE-LTKIHRVLFNQS-
LKPYIDNTKLRQTKNKEKLFKLMNNSVYGK-
TMENVDNRVIKHWKKAEIAKPQFNFTENIMDPIYVGFSILEISKTMYDFLKVYQLLYDTDSLILEY
IPKVSIIGKMKEILLYGTGAKAY-NDIKKAKGVDKLTL

>POLB-1_DYp

VVFHNLSKYDIHLFITELDLSIPCKELYILTKYKIRYIDSVFLNSLDKLSSYMEDQKGFPYDYLDND
TQLPDSFYNDNFKRTIDYKLYLESDVLILADVFNFRKCKYKLDPINYTAPSISWAMLKTNISDDM
YNFLKRAIRGGLTCNFLSYIDANNLYGWAMSQPLPLSFQLKEDGYMLEVDLEYPHDLPFCPNQR
KLIADTNKEIIHQLQLCEHG---IVKVHRVLFTQC-
LKPYIDNTQQRLAENDEKFFKLMNNAVYGKTMENVAKRRQALHYQSFRIARNDFSFGSDISDPI
YIGVTVLELSKWMYELLIKPKIIYDTDSFILSEIQANKEPGKMKEMSFAGLKAKAYSPEIKKIKGVI
KLNH

>sp|P05468.1|
MNDELAFLNSQIDDYSDDIEFLRTEVFRINRYSKSIYSLMKNSSKEAIKETIEFNKYGQEIHSKIKE
KEISLIEAIKLMEKKYKYLRSLNKTKDKSKKKKIHTKDIETLKTIYKTKADFDIRNFKNAETFTYE
NYSLLIPSEKELIDLIPEIEEDYSYYFYVSLTITGPTIFGGVTRKVPIIDTYGDINKDFYNKIKENITKV
LDRVPFYEEKESEIICKKVLITLTVNKWESIDKWYKNVKSKSKKFKDFTLFLASDTSKNCVQQCV
EYLGGKWNTNYSLEKMIPQQKIVTYIPLLMDIQYVQSMEDLISDFDEPNTDCKNIARLLKWNGH
MGVITKIDKDVKINRIQRKRIIDVENKEVKEVFFDIESFSDETKHQIPYLICWSYLGELYYRTGKNC
IKEFVEEILSYNVDIILYAWYGSGYDYQHVLPYMKSKCIKDKYIIKNNMITYGELYYENAIIYLKD
PYLFILTSLDKASKAFNVINKGEFPHSIIHSWEDLNKILPNWVKIQKRMIEYKDDNKLNIYYKNIEI
FENDRNYNTILENAIEYCKVDVLAMEKVWIKFKKLLEKNLNITVSVKTFTLSQLSMKIMESKLPK
YVKLYVPTLEEYSKIRNAIYGGRVISKNGIYEENIVYADVVSLYPSAMKLLKHSYGKPKLVDYID
FEKHGIYRVTLLNKHDQEPKNYLNFVPRRINRKLTWEWFKIHEGWYHTYDLIIAKSEGFDIYCHE
GFEYPEKDYIFNDFIDTLYSMKETHTACSCEEQPCPIRMVAKIALNGGGYGKFVQKPIDKEIYIVK
RDVVAGECDKLQENENGEICIGKRLVKRPLFYNLDSENYDKMIIEKEDEPIYSTQCGISILSASRYR
LYKLCKQFENIDIIYSDTDSIFVKQKSVDWELFKSKCGSELGQLDSTIDDTKNAIISKMYIGGAKM
YAFEYLNNKNKLITKLHCKGVPNYMLSLNQFKYLMESPDNTIAYKFEIIRRKLVNVITKDLIKDIK
QT

>AET14262.1

MEYDKNYDFAVSLLVEQYGELFNSFPLEAKDGLIEQKRQILNMNWEQDNLRKAEERSKKAREM
AKQMKSKMQIERLGQSSMGAYKRYKSVSISMEESCEYKHVEPYPTIVDGAKIRGFRKETILGMK
NRIINAIRESESYQNITRTPVSYMYFKMYNNGKICGSTRTISVYEDTFEEDVDALFIAYDNIVKHIN
DTVYGYESLFSQDANVYTVFYMTALYFPSPRSVLFWGKNIPKIINCGIFRIFSANDTDKDCIEQCS
QYLWNKHFYTLDQIIEYANGNVCILTPQFYIRNIKEIKSYSDIRVHTDSPIKTLNVIDPSVKYLLQF
KEHIGVLFDFKQQKRDNKITRFKPLTKFPKTEKITVCFDIEAYFDPDSENDQTHIPYLCCACFVYN
DVIGNVIEFEGRDCVAQMIEYIVDICSELKNKNIELIAHNGGAYDFHYILSSMYNPSDIKNILIRNN
NFISFSFVHDNITFNIKDSYSFLLCSLANAAKAFLSGNHDFGKTDFPHHDVRSKADLEKVYRKWR
SIDNIIDIEIEKEKLLISSHNIINYETDGKSKKLLDWSREYCCNDVIVLAKVWLEFKVAVFEIFNSKV
VDQSYTLAGMSFKLFESNLDPNIDLRHPKKEDYMNMKDSLIGGRCISVNGIYENILCLDVKSLYP
AAMAFYDQPYGQFRRVQQRMTEELGIYYVRVIPHTKINSNFFPVRYNNKITYNNYGSTQYNAW
YTSVDIDIGISEGHDIQYIPFDEEGNIGYSWINKGKIFQQYIEDTLYKLKLKYEEEGNKVKRNVIKII

MNSLWGKFAQKWIDTNYSIKAESDVDFDEEEAYKIWDTEYMLIKQHKESEYSSKPIQNGVFTLS
WARHHMKLLWDASTRPDTECIYSDTDSIFVRKEEFNLNSVFELNNQKIPVIGTDVGQLELECEFD
KLLCAGKKQYMGFYKYLDAQENPQIGEKKRFKGVPAAYIKPELYTHLLKSPDKMAQVKFLKFR
REWGSVKGYIESKNIRAT

>YP_007714631.1

MIRKPFCNFKMPLVKITEDTPATPGYYYSTSKDELASFLEKVLDNTPIGQLSVSFHVAGSFLKDDE
RKIIHTTPDVFNIHSDGIIPIDVQIDNFIEDYLDPEKSSFSKYSGSGFSLQVILSYWVNIVPYSPNNIA
VPTSRNTQDYRDGTPAPIELNLSSSIRRNFIGVIAQHFTEPGYTKSGKVKSTYPLEVIEEFIKQRGFE
RYAELAVNVSTIPAIHAELNEFNIIIWSITGIPIYAKILGEVDEVPIHMLKKGDNLHLIRSIRAFLNEN
KTNQNRVFCTLCKKFHIPNACNITGEIICIDDIKDNEKDKGLISPISKPKIIYPNTKHCLVAYADFEA
IIDNNIHKPASYSLIPIIAGDPPAENFIFTKSVLNLEDEVFNEDGEIIKFDNIIEMFLTDLYSIIKKFQTN
DYGKEANLIDEAGERFKCLACNKNKKGKYYYARHYGLGIFGYYCRSCFLAHNNTFIVYFHNFK
GYDHHILLEQLLNKDSKHNTCRGKSINKMDVITHKDILSDFIRITFKDTFNFLPESLASLANKLTTL
KYTPDKFKEAFNSGKGEFPYEWFDDFNKLEEIEVPQDPADWDSRLTNKKGTEEIIKKANQIWIDN
NMQIFHDYVLLYNELDVWLLLEVFEAFRDTTVNEDKIDPVYFDGAPGLTFYLARMYENSLDMH
VIPDKNVYLDVSRNIRGGVTQVVTKYANIEDVDETIVYLDVNTMYSYCMKQKLANKYLGTLDA
LPDNYDSDDNFCYFIKGDFSYPEYLHDLPAHLSMPLMPHQYNNKLCTTFLDKKDMLIHSKVFKY
YLSKGLVCDKIHYVYKFKQEYIIKDYVETNIQKRNSSTDPGTKDYYKLKNNALFGKTCENVFKY
KIFSVTNVNSGDRENKCMSKAKSHITLGNCILYEECVTRYLLDKPIQIGFTILELAKLMIYEFIYEL
FDVIPEGSTATMLYTDTDSVIFKFKGFNGVHPYKYLLTTSLASKLDIPINKDGSFGSATKTPGLWS
DDTKYKTITEFIGLRAKQYAYSIANDRDILKHKGIPKNALKDDNNPMNVNDFRNVLFEMKDLTV
NIAQIRATKNVLTSTVSKKLALSTKDNKRITYTDKVTTLPFGYKGELYSNYMDDVVIE

>AKU37609.1

MIRKPFCNFKMPLVKITEDTPATPGYYYSTSKDELASFLEKVLDNTPIGQLSVSFHVAGSFLKDDE
RKIIHTTPDVFNIHSDGIIPIDVQIDNFIEDYLDPEKSSFSKYSGSGFSLQVILSYWVNIVPYSPNNIA
VPTSRNTQDYRDGTPAPIELNLSSSIRRNFIGVIAQHFTEPGYTKTGKVKSTYPLEVIEEFIKQRGFE
RYAELAVNVSTIPAIHAEINEFNIIIWSITGIPIYAKILGEVDEVPIHMLKKGDNLHLIRSIRAFLNEN
KTNQNRVFCTLCKKFHIPNACNITGEIICIDDIKDNEKDKGLISPISKPKIIYPDTKHCLVAYADFEA
IIDNNVHKPASYSLIPIIAGDPPAENFIFTKSVLNLEDEVFNEDGEIIKFDNIIEMFLTDLYSIIKKFQT
NDYGKEANLIDEAGERFKCLACNKNKKGKYYYARHYGLGIFGYYCRSCFLAHDNTFIVYFHNF
KGYDHHILLEQLLNKDSKHNTCRGKSINKMDVITHKDILSDFIRITFKDTFNFLPESLASLASKLTT
LKYTPDKFKDAFNSGKGEFPYEWFDDFNKLEEIEVPQDPADWDSRLTNKKGTEEIIKKANQIWID
NNMQIFHDYVLLYNELDVWLLLEVFEAFRDTTVNEDKIDPVYFDGAPGLTFYLARMYENSLDM
HVIPDKNVYLDVSRNIRGGVTQVVTKYANIEDVDETIVYLDVNTMYSYCMKQKLANKYLGTLD
TLPDNYDSDDNFCYFIKGDFSYPEYLHDLPAHLSMPLMPHQYNNKLCTTFLDKKDMLIHSKVFK
YYLSKGLVCDKIHYVYKFKQEYIIKDYVETNIQKRNSSTDPGTKDYYKLKNNALFGKTCENVFK
YKIFSVTNVNSGDRENKCMSKAKSHITLGNCILYEECVTRYLLDKPIQIGFTILELAKLMIYEFIYE
LFDVIPEGSTATMLYTDTDSVIFKFKGFNGVHPYKYLLTTSLASKLDIPINKDGSFGSATKTPGLW

SDDTKYKTITEFIGLRAMQYAYSIANDRDILKHKGIPKNALKDDNNPMNVNDFRNVLFEMKDLT
VNIAQIRATKNVLTSTVTKKLALSTKDNKRITYTDKITTLPFGYKGELYSNYMDDVVIE

>YP_009704122.1

MSVVSSDGTCCFYSESTDTADKFASNRPCNPSSATASPSSRPSRSCTSKSGRRSVIQKRSTLSVKG
TLGEGKHLMLKYHSDFLNALKNLFAVHLVPWSNFLDIGVLHPHNIVTKMSALQPSILQIFTFYKG
QFHLTEKTWNSNLTPFPIRILIRHGKIFLVSAVETIQKCLYCSDFFKNSHTCNVRRREFYFHHVNFT
TKHWWEPISFRPIGSIPTTKRLFVTYDIETYTWHGKYGKQLIPYLLVFHLSGDPDLIISAKKVALEN
NWNQWQNEDTFFVLTPEKREIGKRFKSFRSQLQKINTDFLWNDFLTKNPSIRQFQSEKKILTPED
VPFKVLQKLPLMGDPVFLEIYIIGHNISGFDEIVLAAQVIANKADIAPCFRISRNFMPRAGKILFNDI
TFELPNPLYQKRKTFPLWKNGSLHPSELPCQYVKFMVRDTLSLTHTSLRTAASAYSLNVEKGHC
PYEAVNAFYRTGMYLQDEDGFPHVTYWKSEDEYNFNKELWRKKKMGAYDIIHHALCYCIQDV
KVTAELVQKLQQSYNTFIQQEINLPEASFNIFQRPTISSNSHAIFKQVLYSAEKMTGNTLGDILLAP
SNEMYDYVRESIRGGRCYPTFLGILSEPVYVYDICGMYASALTHPLPIGKPLNPLERAKAAFAWK
TKLESKKQIDYFCSDLLPGIFTIDADPPEENFLDVLPPFCSRRGGRLCWTNEPLRGEVATSVDLITL
HNRGWEVRIVPDERTTIFPQWKCIAREYVNLNISAKEKADTEKNQTLRSIAKLLSNALYGSFATK
LDNKKIVFSDQIDADTAKQIANGNYVVKSSSFIETDDLSAEIIPEFVVAYPPVSSSEAQDNDKNTV
SSSPYIASEPRNYLYKPITFLDFEEDDVCLHTLEKTTPTVENNRYPSQIASFVLAWTRAFISEWCEF
LYLEDRGIPFEDRVLKSVYGDTDSLFLTEKGRQLMETKGKHRLKKNGGNLVFDPSNPAITWLVE
CETKCLKCGEDAFSPESVFLAPKLYALHSLKCNKCGFIGTGKLRAKGHPTNELSYELLKECFLSH
YQQGTTTFQTARLSLKKTLANANRNEAPFTVTETTLKRTIRPWNDKTLRELTSQNLVPYSQSNPN
PRNQNVCLMNLPWDA

>YP_009362839.1

MSNLKGTYVAQRACGLAQGIDEEGTPLEIKYFNNLEKSLNNLFKVNLITFPFCLQSITYKNLIEKIE
QACPTSSIVYTAFKSKFKQFKVTIPNPKLSIPLNFLIKNYKVYLIHSVVSLNKCEHCGRFYKVNHA
CSLRRRDYYYHHINTQTCDWWENIAFRPIGSCSKIKKIFITYDIETYTWHGKFGKQLVPFMLVLNF
SGDDELVKICSSIAIRENWNKCTEENTYFYINPHKREVGQKFKVLREKIQQYLIIKLWDKLQVQNP
CINDLKNKLGLISCYDIPQEEFFKLKLKGEPTFFEIYIVGHNINGFDEIVLAAQVINNKLEIPQPFKVI
RNFLPRCGKILFNDITYALPNPEYESPGKNDFEQWEKGIFESKHLKYQMVKIMVRDTFALTNTSL
KNAAKAYNLPIEKGSCPYSAVNEFYMFGSYLSDPDGFPSIKYWQSPEEYNTNKELWKKSNTKYD
IIQETLKYCILDVLVTTKLVQELQKSYQKFICESVNLPECNFNIFQRPTISSNSHAIFKQIVYRAEKP
LSKHIGKTLVAPSKEMYDYVRASIRGGRCYPNYLGVLSEKIYVYDICGMYASALTHPFPVGLPLS
AFDCGVQIQIWKQYLSRNENISYFNPDLLPGIFTIDADPPDEQFLDVLPPYCSKKGGRLCWTNEPL
RNEVATSIDVITLHNRGWEVKILPDKKTTIFPEWKCVAAEYVQLNILAKEKADKEKNQTLRSIAK
LLSNALYGSFATKIDNKKTVFSDQIDKETQNKIASGQYYVKASSFIETDTFSSELLPEFKVLYSPDT
SQKGPHSPVDTDSDDEDVTLNTPQNHVTYTYKPIIFLDVEDDDACLHTLELNTPLVTNNRYPSQI
ASFVLAWTRAFVSEWAGFLYDDDKGIPLHERPLKSVYGDTDSMFVTEKGRELMESKGKHRLKK
NGGKLIFDPQNPSLTWLVECETQCSKCGCDAYSSESVFLAPKLYALKNTCCDNCGHIGPGKLRA

KGHATKTLSFDLLSACYYTDVQEGSERYQTSRMTLKRTLASCQTNVQPFTVMESTLTRKLRPWK
DKTMVLVDQHKLVPYSKSNPNPRNKEVYWTELPWDM

>ALG96753.1

MANLQILNNGKGKVQLKLNNFSNFVPSFKIRSGFFTVHKINFEKEHDTSYEKEKQQSAFDIETNG
KIIFFGAYNGQEYKYVIIRKPEDVHTALNLLTEETYFYGDYDLPVSLANYVLLGKHSKFAKKIAG
ESFFSTDNFRIKRFKNFYRILFGDRAINAINLLQFYSESLFEAYSRYYNKLKELGFDVFDNQTLKE
WKEDKEKRVNFDKLDFNDKQTIEEIARYNKLDVIATYQLALLKNSLFGIQVKTTLPRTAISYIISQ
VQSKFVKGLKPYPEIELTLKRLYKGGLFDSNELGKFKKVYKYDVNSMYPYMMTWLPELELVDI
QKGFEVNEEPILRGTEFNEDVKYLYIYKISLKQSKKYVASKANGMLLRMMYSRGSFFDFELADQ
KSELSKDIKIVGEYYTMKFRITKHRIFKDVIEDLYSKRLQLKKQKNPLEKVYKLILNSSYGKFGER
IGFNAKFQNVIYASMITALGRTFIQNVDPYAISYLTDSVISKQPIKAELVGDQLGQLKMEGEGPAI
VIGNGQYILEDPEEKMVKLRGFNVDEKLAEKIIEYIGNNLAKGKIIRVQIPTKIMVRNLQQFKVLS
EKDSNVLMGLLTNQIKSFTPLNTKQRYIYNSYWYGEMFRDEIDHKEYTKSWRKYIETIEPIDLNTI
IS

>sp_A4ZU91.1

MLQILNNANDKIQYKLNDFSDLTPSFKIKSGFFTIHRINYEKLEENEEEKPRQISAFDIETNGREIFF
GVYDGSEYKYVIIRKPEDVKNALDLLTEETYFYGDYDLPVSLANYVLLGKHSKFAKKITGENYF
TTKNLRIKRYKNFYKILYNGRSINSINLLQFYSESLYEAYSRYYTKLQEMGFQVFDEQTMKEWKE
DKEKRANFDKLDFNDPKTIQEIARYNKLDVIATYQLALLKNSLFGIKVKSTLPRTAITYIISQVQSE
FVKGLRSYTEIELTLKRLYKGGLFDSNELGKFKKVYKYDVNSMYPFMMSWLPELELVETQKGFE
VNEEPLLKGTQFNEDAKYVYIYKITLKQDRKYVASKANGMLLRMMYNRGSFFDFELADEKHEL
IPDIKIVGQYYTMKFKITKHRIFKDVIYNLYNKRLQLKKEKNPLEKVYKLILNSSYGKFGERIGFN
AKFQNVIYASMITALGRTFIQNVDPNAISYLTDSVISKAPIKSELVGDQLGQLKQEGVGEAIVIGN
GQYILNDPQEKMIKLRGFNVDESIAEKIIEYVGNYLAKGKIVRVQIPTKIMIRNLQQYKILAEKDS
NVLMGMLSNQIKIFTPLNTKQRYTYNVHWFGSMFRDEQEHKEYRKTWEKYIETIEPIDLNSILS

>YP_529524.1

MAKCDKSLEAIDLDRAYTAPRKAKWAENKRINGLDTETSDGDIFCISVCWEGEKPMVQHNDRE
KLTSKQVWQVLTDHKARSSLNMWYNLDFDANVVLNHVCSEEQLAELVVSGTTLANSDRTYRQ
YMDTDKELRKGEYLITYIQSKFLEIKDHNSHIYTHYDASQFFYTSLENAVTEWLGESKANDGLEA
GLFGSQTPNQLRETVAESDCVTWTNLSLTYNVSKGDKWTIHNAKSYISKNWSDILKYAQIDAEL
VRDLWQEAVNVGEELDIPMGRPFSTGYLAESYLDNRLREKPGLGPMPMAKMAWESYAGGRFE
VLKRGNVGRVAGPDINSAYPAVLAELPDPKTLRWKRAKHASISEIETADYGFMTVKVSTDPTREI
QPFAVKDEKQDKLVYPSPQNTEITVVKDIFIHAYNQGYVTDYEVIDCWLGYKTEGTTFPFDFIPEL
YDNRKTAEANGLEKRGLLLKIVLNSMYGKTCQTTPKRRELAESTELELHESYVPDMSLPKMIRE
KYSEGFIESLTAGAWFNPFLASYITGLTRLELHKQICKHDLEENTVMLATDCVMIEEKPFEESNFV
ENLVQDGLGYWDMEYKGDAFVLGAGVYQIDFDTCQKGCKDNCNKFSHKHKVKTRGFSEADLE

KGLVNAAEKANGHIEIESTRPQTISEIIWSNEELSQVGNFLEQERKIKPEMDTKRKWSENTDFKKL
LSTCETSLPLKI

>NP_040682.1

MPRRSRKKVEYKIAAFDFETDPFKHDRIPKPFSWGFYNGEIYKDYWGDDCIEQFIYWLDTIEEPH
VIYAHNGGKFDFLFLMKYFRGKLKIVNGRILEVEHGIHKFRDSYAILPVPLAASDEKIEIDYGKME
RETREQHKAEILEYLKGDCVTLHKMVSLFIAEFGMRLTIGGTAMNELKQFHPYDPVRKGFDEAM
RPFYFGGRCQAFEKGIIEDDIKVYDVNSMYPHAMRNFRHPFSDEFYEANEITEETYFIEWEGENN
GAVPVRTKTGLDFNQRSGIFHTSIHEWRAGIDTGTIKPNRIIRTINFTETTTFGAFIDHFFSKRDAAK
KAGDLFHNIFYKLILNSSYGKFAQNPENYKEWCITEGGIYLEGYDGEGCEVQEHLDYILWGRPAE
MFNYFNVAVAASITGAARSVLLRALAQAERPLYCDTDSIICRDLKNVPLDAYQLGAWDLEATGD
KIAIAGKKLYALYAGDNCVKIASKGASLVPRDIGFLMPPDMEPKAAKKVAQQKAKNIGGEKILK
VANGGVYDFVNDAPSFKLNGNVQFIKRTIKGT

>AAX45532.1
MPRRSRKKVEYKIAAFDFETDPFKHDRIPKPFSWGFYNGEIYKDYWGDDCIEQFIYWLDTIEEPH
VIYAHNGGKFDFLFLMKYFRGKLKIVNGRILEVEHGIHKFRDSYAILPVPLAASDEKIEIDYGKME
RETREQHKAEILEYLKGDCVTLHKMVSLFIAEFGMRLTIGGTAMNELKQFHPYDPVRKGFDEAM
RPFYFGGRCQAFEKGIIEDDIKVYDVNSMYPHAMRNFRHPFSDEFYEADEVTEETYFIEWEGENN
GAVPVRTKTGLDFNQRSGIFHTSIHEWRAGLDTGTIKPNRIIRTINFTETTTFGAFIDHFFSKRDAA
KKAGDLFHNIFYKLILNSSYGKFAQNPENYKEWCITEGGIYLEGYDGEGCQVQEHLDYILWGRP
AEMFNYFNVAVAASITGAARSVLLRALAQAERPLYCDTDSIICRDLKNVPLDAYQLGAWDLEAT
GDKIAIAGKKLYALYAGDNCVKIASKGASLVPRDIGFLMPPDMEPKAAKKVAQQKAKNIGGEKI
LKVANGGVYDFVNDAPSFKLNGNVQFIKRTIKGT

>WP_000039472.1

MSKKTKKERQKSAKLLTLDTETRGLTGDVFRVGLFDGENYYVANTFDEILNIFDQYKNYECHVY
VHNLDFDLGKIATTLFTRDRVCFAKSIFINGNVVTLHADSMILHDSLRLLPGTLEKLCKDFGLTDN
AKKDLSDIMKEQGYAIYNADGSFNKRKSLGNYFENVPADDPTLNEYLEYDCRSLHEILSIVMDIA
GIDLETLVKCPTTASLSMRVFKEQYSEDYDKVATHWYTGEWGRFLEDHVRLSYYGGRTEVFTP
HLQNGYHLDVNSLYPYVMKIAKFPVGYPNLLKDGQAEKKWRHWKRRAVGGGVMWCRVDVP
EDMYLPVLPKRDPSGKLLFPVGKLEGVWTLPELLEAERNGCTIEAVYQMVYWEQMEYIFKDFV
ENFEVLKKTSEGAKKIFAKLIQNALYGKFGMQRKRTSYGDMRDIHDLQEKGIEYRVHKHTNNGI
EMEFTEYLSESKAMYIQPHIASYVTAYARILLYRALKEQHEKGVIGYCDTDSIACESMMDANMID
KEEYGKWDLEGVIKEGIFLQPKFYAERHENGKEVIRAKGIPRDKMEEISFDNYKEWLEIMKEGEQ
ERIHIFDGHQARKKFSTTLKADEHFDTQREMKKSINLLLEQKRIIDYKNNVTRPHARYDYGPKKD
EMNYKDYQEYEKKLNDMYDDVDDIKELINDLGYIKCMSKGDMYYEEYKQFPRSVRSKYFRKT
GIPIDVWSEEAGWELNDLLEEFRLMGV

>WP_014905375.1

MTTTNRKKRREIKLFTLDTETRGLDGDVFRIGLFDGKQYYTGYTFADVLPVFEKYKAYDCHVYI
HNLDFDLSKIIAELRDYAEPTFNNSLFINGNIVTFTASHIILHDSFRLLPSSLENLCRDFDLLEGGKM
DIVDYMEENNYGIYNVKNRKLNKRLTKGNFFTTVDKDDPVLCEYMEYDCRSLYKILEIVIGLSK
LEVEQFINCPTTASLAKTVYKEQYKKDYKVAISTKQYNHKQIGKGLEAFIRKGYYGGRTEVFTPR
IENGYHYDKNSLYPYVMKMAEMPVGYPNVLDNEEAELSFDLWKRRKYGAGFIHAKVHVPEDM
YIPILPKKDYTGKLIFPVGKIEGVWTFPELALAEAEGCRIEKIESGVVFEKTAPVFREFISYFEEIKN
TSKGAKRAFSKLMQNALYGKFAMQRERIMYADISERDKLEAEGHTVSEIIYDMNGIRMEFLEYD
GYAKAEYIQPHISAYITSIARILLFKGLKYAHEKGILAYCDTDSCATTTKFPDKMVHDKEYGKWK
LEGYVIEGLYFQPKMYAEKAINTDGEYEEVLRMKGVPKWVVEEQLDYNTFKKWYLQVKRGKA
EIPIYKGGERVQKFLTKSKNNIEMNELAEMHKTINFAREQKRNIDLNKNTTSPLVRNDYGENKDE
KSEYEFDEWYERLEEFNDDMNAVEELCMKFGKIQIPEKKQRKLYGLYKEYSSKAKAMCFSNEG
LPIQDWCKKTGWDMKELLGELSFL

>QSJ05034.1

MAKSDRNLDEVNLYPAYQDQYSATFVNGKLINAFDTETSSGTVFMLTSAYGDKTQAYYNRDVS
ELDAETIMDALTDYKTRSNINIWYNLDFDANAILSGILSQKEMSELVVTNETTTTVAGIEYEIFYIK
GKMLRIVDENGNISPHYDIAQFFYTSLDNAAEEWLGENKKEGIDTSKFDDKEYIKDNFDEILKYA
KKDASLTQDLAIELTDEAENLDIPMGRPISTGYLSAEYLRANTEEKPSLGNETMQNLFWESYYGG
RFEVFQRGNVGEVVAPDINSAYPAIMKDLPDPTTLNWNHYLNEVSDKEPFSHSINKFGYEEIENG
HYGVVKARVTTDSSRMIQPFACKIDGKVKFPAMTNKVVTVIKPIFEFAVNNGLVTDFELIEAWIG
NITDRTSKPFEFIGDMYAERKVFEQLKNKPKKGQLLKIVLNSSYGKTCQTTEKRHKHDLGKDGK
KIMQAHETQYPRFYLSKKQREALGDDEIIITELEAGKRFNPFFASYITGLTRLELHKQVVEHDIEDS
TVMFATDCLMVEKEAYENSSFDEQIHVPDDSLPESEFRKEATRSLGAWDFDYEGSAFIVGSGVY
EVDTIQGKTKTKTRGFIESNLGDTLKGLAKKHKEAIPLDNERPLTMAEVLINTERGSVSEFVENSK
KLKPDFDDKRNWNRDNPDFHDLLNEKEYSKPIDLQEQKEEMIEQHMDIDEKMIGDATPNGNET
VVVKDD

>DAV39626.1

MGIYAADFETTTDPDDCRVWAWCICDIYNIDETIEYGETIYSFIEYISNLHGKIYFHNLKFDGTFIV
DYLLKHNFEHSQERKIYHNEFSTLISDMRQWYQVRFVPGRESGVEDEIQITDSLKILPMPISDMPK
SFNIEEKKLKIDYKADREIGHELTQEEKDYVAHDVIILAKALKFMYDHNQTKLTTGSNALNDYIH
RLGKEEYKVRYPELDLPTFTDFKKAYKGGFTYVNPAYKDKDVKEGAVFDVNSMYPWAMKNCL
LPYGEPVYFPKKYKENPMYPLYIQCILCEFKLKPNHYPCIQIKGHFMYHDTEYLTQSIEPTYLYLT
SVDEKLVFDHYDVNVIEWCGGYMLKGTHGLFDEYIDYWYNEKTEARIEGNPGREKIAKLMLNS
LYGKFGSKKRGKSCIPYLREDGRIGFKLSEEEIRKGGYIPMACFITAYCRDKIIRGAQICGDRFIYA
DTDSLHVAGTEPPEGLWVDNKALGAFKLEETFIRAKFIRQKTYLEVTLGKDYQEKINIKCAGMP
KNVKETITESEFKEGAVFDGKLLPKIVPGGVILKETTFKIKKAKGVDNSLSL

>DAV34512.1

MRIFSCDFETTVDDDTKQQTSTEVWSAAIAELYSDFVTVYNNIHDFIKFFHNLCEEKVIAYFHNV
KFDGNFLLNTLMENGYKFHHREKPYEKLYKGEFDAIISGQNRWYSITVCTGRTLIEIRDSAKLMP
MTLARMGKAFNTKHRKLEMEYKGERHAGGLIKPEEMQYIINDVLVLKEALEFMLDSGNTRLTIG
SNCIAEYKKCFDKEQWNAMYPDLKAITLDEQAYKYPNADAYIRRSYRGGWCYCNPKYMNKWI
DADGMTYDVNSLYPSVMHSKSGNIYPVGKPTFWTGNKIPEEALQENRVFFVRLKARFTIKPNHL
PTMQIKDSLMYKSTEWLTSSDVQFGGKKYAYYYDADGILQLAYAEFTLTSLDYKLFLEHYDIHE
IEILSGCYFNAVSGLFDEYIDKYMAMKMNSEGGAREEAKLFLNNCYGKLATNDDSSYQEPYLDE
DGLLRFILHEEHNKKTLSIAQGSFVTSYARYFTITHAQANYDNFIYADTDSLHMFKCEPNKIVEHS
SKLLCWKLESEWSRAKFIRQKTYCEFIRKENHKKVTPHWEIKCAGMQDRTKQYLLATRPISCFD
YGLTLNSQLKQKQVKGGILLVDADFTLYKQKAYKPPKSFGKVLDKIQ

>BNT_ADNK01001243.1

DVNKCYTWAL-
KSISQIPIFHYFDNYVKYDDHSIEDLAMYVVMSLDGGEAHNILLPKRCNRVYGFKLKQMNKN-
SYDILYYIRPS-
NIATVKFSKAVDKLYETELGTINANKFICNKITGMLEKKYNHKSVCKIYKNLNEANHYKKLYGG
E---INPMNDDLFLLVRTTKKL-VRETFKPIKDMIYDMVSIKMCNLYDKCVSVGLHPIAIKTDCIIV

>PLV_YSL2

NLMAWDINKCYSSCMNKPTEDWIRYDFNDSWEDYDNELKLGLYYVKTNDKTLFKQTGYYSTA
IIKKAMTENIEFEILKQLIPSHTESKDTFTKIIDVVLKYSKGDEDISKPIINIMSGMLGQSSSTISKHIK
INNDIEQIFNFLNNYYDLGEGIMINKIENTDYYMYGFNKEIKFSETNIPMYIQVVDESNIKLYDMV
KKMGGKIGRILVAYKVDCAVV

>PLV_SAF5

NTIARDINKCYGSIMYNPLTKWIRYDFNDCWEKYDGKLKLGLYYVRTEDTRLFHKSDIYSNCIIE
KAMTTNIDFEIEYQLIPKYQEKKNMFKLVIDKILEYSKGDKNIYKLMINMISGMCGKTKRSAGNY
KINKDINQIFQFLHKYPDMDTVIHQIPETDFYLYGAEKEMKITENNLAMYFQIIDQANIKLFDMVQ
DMRGTLIGRKVDCAVV

>PLV_TIR

NKMGLDINKSYTNVILNSGYDFNQYNVFDEVVKYDGREIVQGSYFLSKGIVIERIKVCLPPGLFE
YPIVQWALKNKFIDEKEITHMIIPQKMIDSKIFERFVLTSIAAQESDEIESFDFFDPDTFEPCVIQGK
KKYGFAKHLVNNFIGCLAKSHNKKVQGTMINNYLEACALFNKVMSEGNESSIYQHQNMFFVTS
TSKSMQLKNSIPIYRQIIELGMMQLFQLALDACNEKSKVVSYNTDYVSI

>AEY99251.1_Mimivirus_lentille

NLRTIDKNKCYSFALYSLPYLIKFDFRKNNIRSRPTEIVDHYLYVAKPNNWTILMPCTKLYAGYFL
KDCFNAGVKFELLEELETETVDNYFRQIIKLMYDNMSSQSFKEAMNIFIGKFERSFSKSYEYKYV
GIYDDEAIDTKEGFNVRLGNYNLLFKENEQYLHVRDRLPIATQIKDMSRMFIHKKIKELKIRDQDI
VQINTDSISY

>AEY99257.1_mamavirus

NLRTIDKNKCYSFALYSLPYLIKFDFRKNNIRSRPTEIVDHYLYVAKPNNWTILMPCTKLYAGYFL
KDCFNAGVKFELLEELETETVDNYFRQIIKLMYDNMSSQSFKEAMNIFIGKFERSFSKSYEYKYV
GIYDDEAIDTKEGFNVRLGNYNLLFKENEQYLHVRDRLPIATQIKDMSRMFIHKKIKELKIRDQDI
VQINTDSISY

>BNT_ADNK01003537.1

TLEGYNLYVLKPSKNSNVIEEYGKKMNDFHQFKAITGYDYKRSN-YDDTEMSLYIWNQ-
FRPQEVEFGKTTEMEEEWVY-KASRGGIRYSVDGEYQNK-
DYIKLDLNKKYTSVLCSNYFTIPLGTPMTRTITQEQVDKYYKFNYGLYNIEISKDDDVFFKYNKH
NIYTHLELKQAKE-LGLKLNLLDT---NYLYYKEKLKSIKLFGKFFHRLLPHS-
KSQDLVKLIMNKLWGIFQTKLRRK

>BNT_ADNK01003404.1

TLEGYNLYVLKPSKNSNVIEEYEKKMNDFHQFKAITGYDYKRSN-YDDTEMSLYIWNQ-
FRPQEVEFGRTTEMEEEWVY-
KASRGGIRYSVDGEYQNKYYIKLDLNKKYTSVLCSNYFTIPLGTPMTRTITQEQVDKYYKFNYG
LYNIEISKDDNVFFKYNKHNIYTHLELKQAKE-LGLKLNLLDT---
NYLYYKEKLKSIKLFGKFFHRLLPHSK-SQDLVKLIMNKLWGTFQT---
KLRRKVNTKQVIEIDDRESNETPTYKIGPDGYTILSLTKFEEEKKYFKYYACRIAPFLTSYVRTYM
VGLINEYKDDVVYSHTDSLVIARSKAIHFEISSELG

>BNT_ADNK01002942.1

TLEGYNLYVLKPSKNSNVIEEYGKKMNDFHQFKAITGYDYKRSN-YDDTEMSLYIWNQ-
FRPQEVEFGKTTEMEEEWVY-KASRGGIRYSVDGEYQNK-
DYIKLDLNKKYTSVLCSNYFTIPLGTPMTRTITQEQVDKYYKFNYGLYNIEISKDDDVFFKYNKH
NIYTHLEL---KQAKALGLKLKLLDT-NYLYYKEKLKSIKLFGKFFYRLLPHS-
KSQDLVKLIMNKLWGTFQTKLRRK

>BNT_ADNK01001734.1

GGIRYSVDGEYQNKYYIKLDLNKKYTSVLCSNYFTIPLGTPMTRTITQEQVDKYYKFNYGLYCV
EISKDDDIFFKHNKHNIYTHLELKQAKE-LGLKLNLLDT---
NYLYYKEKLKSIKLFGKFFHRLLPHSKSQ-DLVKLIMNKLWGIFQT---
KLRRKVNTKQVIEIDDRESNETPTYKIGPDGYTILSLTKFEEEKKYFKYYACRIAPFLTSYVRTYM
VGLINEYKDDVIYSHTDSLVIARSKAIHFEISSELG

>BNT_ADNK01000904.1

TLEGYNLYVLKPSKNSNVIKEYEKKMNDFHQFKAITGYDYKRSN-YDDTEMSLYIWNQ-
FRPQEVEFGKTTEMEEEWVY-KASRGGIRYSVDGEYQNK-
DYIKLDLNKKYTSVLCSNYFTIPLGTPMTRTITQEQVDKYYKFNYGLYNIEISKDDDVFFKYNKH

NIYTHLEL---KQAKALGLKLKLLDT-NYLYYKEKLKSIKLFGKFFYRLLPHSK-
SQDLVKLIMNKLWGTFQT---
KLRRKVNTKQVIEIDDRESNETPTYKIAPDGYTILSLTKFEEEKKYFKYYACRIAPFLTSYVRTYM
VDLINEYKDDVVYSHTDSLVITRSKAIHFEISDELGKWKEEK

>BNT_ADNK01000127.1

TLEGYNLYVLKPSKNSNVIEEYGKKMNDFYQFKAITGYDYKRSN-YDDTEMSLYIWNQ-
FRPQEVEFGRTTEMEEEWVY-KASRGGIRYSVDGEYQNK-
DYIKLDLNKKYTSVLCSNYFTIPLGTPMTRTITQEQVDKYYKFNYGLYCVEISKDDDIFFKYNKH
NIYTHLELKQAKE-LGLKLNLLDT---NYLYYKEKLKSIKLFGKFFHRLLPHSK-
SQDLVKLTMNKLWGIFQT---
KLRRKVNTKQVIEIDDRESNETPTYKIGPDGYTILSLTKFEEEKKYFKYYACRIAPFLTSYVRTYM
VGLINEYKDDVVYSHTDSLVIARSKAIHFEISDELG

>KU052222.1

MNDEEIDLLEYVQNLTRQDITPKDKKKQLKLDGKPVKADYYKIIDEEQAEDHNTTIEILNELLKQ
EEEYQDKLLQISFTYDDKLSFSISHGFFEVKQDGNLYGDTYSFFDDYPDTKHRVQDVITGFAVFY
VDKPQNEGGCDGKYNNCLYHCLQDSMGDRVPWKQAQNMKKFLKLKKNDKVPYELLDQLEEK
IKANIILSGSYVRESSMQYAETIRITLKDGHYSLTEKRTRLQKQIEGNCRHYHNEPEKKLLIFQNH
KNKIRAWDGATFTYVDKSKYTTLEGLKIKCPDDEDIKEFYNMTKTDFNLLKELSNGKYNPFKFY
NIIQLAEDIFLQNFKGVEPEPIEQEEALILNKAMGGGVRYAKPGTYKKAFDYDINRFYPHLQLTLS
VPVSKPTFKTIEELPDILPYGIYKCQIKTKNKLFKANRYNLYTHYDLNAIRETMKAEIKLIKNDGW
NLMQYEGKRIQLSRLGKDYKKLYDISKKGCKVAKMAMNCLWGAISRRKFKLLYTDTLNEPLEL
NLKDKLRKLEYSDVIQVEKTDTFKAYANDIGGRFSVFLTAKGRYKLNQIIQPHKKYIKYVHTDG
WISSKELDLTLSDEFGGVRLDKKGDVEIINKNIKKYI

>HQ712116.1

MNDEEIDLLEYVQNLTRQDITPKDKKKQLKLDGKPVKADYYKIIDEEQAEDHNTTIEILNELLKQ
EEEYQDKLLQISFTYDDKLSFSISHGFFEVKQDGNLYGDTYSFFDDYPDTKHRVQDVITGFAVFY
VDKPQNEGGCDGKYNNCLYHCLQDSMGDRVPWKQAQNMKKFLKLKKNDKVPYELLDQLEEK
IKANIILSGSYVRESSMQYAETIRITLKDGHYSLTEKRTRLQKQIEGNCRHYHNEPEKKLLIFQNH
KNKIRAWDGATFTYVDKSKYTTLEGLKIKCPDDEDIKEFYNMTKTDFNLLKELSNGKYNPFKFY
NIIQLAEDIFLQNFKGVEPEPIEQEEALILNKAMGGGVRYAKPGTYKKAFDYDINRFYPHLQLTLS
VPVSKPTFKTIEELPDILPYGIYKCQIKTKNKLFKANRYNLYTHYDLNAIRETMKAEIKLIKNDGW
NLMQYEGKRIQLSRLGKDYKKLYDISKKGCKVAKMAMNCLWGAISRRKFKLLYTDTLNEPLEL
NLKDKLRKLEYSDVIQVEKTDTFKAYANDIGGRFSVFLTAKGRYKLNQIIQPHKKYIKYVHTDG
WISSKELDLTLSDEFGGVRLDKKGDVEIINKNIKKYI

>YP_004300281.1
MNDEEIDLLEYVQNLTRQDITPKDKKKQLKLDGKPVKADYYKIIDEEQAEDHNTTIEILNELLKQ
EEEYQDKLLQISFTYDDKLSFSISHGFFEVKQDGNLYGDTYSFFDDYPDTKHRVQDVITGFAVFY

VDKPQNEGGCDGKYNNCLYHCLQDSMGDRVPWKQAQNMKKFLKLKKNDKVPYELLDQLEEK
IKANIILSGSYVRESSMQYAETIRITLKDGHYSLTEKRTRLQKQIEGNCRHYHNEPEKKLLIFQNH
KNKIRAWDGATFTYVDKSKYTTLEGLKIKCPDDEDIKEFYNMTKTDFNLLKELSNGKYNPFKFY
NIIQLAEDIFLQNFKGVEPEPIEQEEALILNKAMGGGVRYAKPGTYKKAFDYDINRFYPHLQLTLS
VPVSKPTFKTIEELPDILPYGIYKCQIKTKNKLFKANRYNLYTHYDLNAIRETMKAEIKLIKNDGW
NLMQYEGKRIQLSRLGKDYKKLYDISKKGCKVAKMAMNCLWGAISRRKFKLLYTDTLNEPLEL
NLKDKLRKLEYSDVIQVEKTDTFKAYANDIGGRFSVFLTAKGRYKLNQIIQPHKKYIKYVHTDG
WISSKELDLTLSDEFGGVRLDKKGDVEIINKNIKKYI

Table S1. Information on specie genomes used in this study

Organism Name	Gene ID
Physcomitrium patens	GCA_000002425.2
Populus trichocarpa	GCA_000002775.3
Sorghum bicolor	GCA_000003195.3
Vitis vinifera	GCA_000003745.2
Cucumis sativus	GCA_000004075.3
Arabidopsis lyrata subsp. *lyrata*	GCA_000004255.1
Brachypodium distachyon	GCA_000005505.4
Micromonas commoda	GCA_000090985.2
Ostreococcus lucimarinus CCE9901	GCA_000092065.1
Selaginella moellendorffii	GCA_000143415.2
Volvox carteri f. nagariensis	GCA_000143455.1
Oryza glaberrima	GCA_000147395.3
Chlorella variabilis	GCA_000147415.1
Carica papaya	GCA_000150535.1
Micromonas pusilla CCMP1545	GCA_000151265.1
Ricinus communis	GCA_000151685.2
Amaranthus tuberculatus	GCA_000180655.1
Lotus japonicus	GCA_000181115.2
Oryza barthii	GCA_000182155.4
Fragaria vesca subsp. *vesca*	GCA_000184155.1
Theobroma cacao	GCA_000208745.2
Ostreococcus tauri	GCA_000214015.2
Schrenkiella parvula	GCA_000218505.1
Solanum tuberosum	GCA_000226075.1
Oryza brachyantha	GCA_000231095.3
Coccomyxa subellipsoidea C-169	GCA_000258705.1
Setaria italica	GCA_000263155.2
Brassica rapa	GCA_000309985.3
Cucumis melo	GCA_000313045.1
Musa acuminata subsp. *malaccensis*	GCA_000313855.2
Pyrus x *bretschneideri*	GCA_000315295.1
Citrus sinensis	GCA_000317415.1
Leersia perrieri	GCA_000325765.3
Betula nana	GCA_000327005.1
Gossypium raimondii	GCA_000327365.1
Cicer arietinum	GCA_000331145.1
Oryza meridionalis	GCA_000338895.3

Cajanus cajan	GCA_000340665.1
Prunus persica	GCA_000346465.2
Prunus mume	GCA_000346735.1
Nelumbo nucifera	GCA_000365185.2
Capsella rubella	GCA_000375325.1
Nicotiana tomentosiformis	GCA_000390325.2
Nicotiana sylvestris	GCA_000393655.1
Pinus taeda	GCA_000404065.3
Leavenworthia alabamica	GCA_000411055.1
Sisymbrium irio	GCA_000411075.1
Aethionema arabicum	GCA_000411095.1
Picea glauca	GCA_000411955.6
Morus notabilis	GCA_000414095.2
Zizania latifolia	GCA_000418225.1
Azadirachta indica	GCA_000439995.3
Elaeis oleifera	GCA_000441515.1
Genlisea aurea	GCA_000441915.1
Elaeis guineensis	GCA_000442705.1
Tarenaya hassleriana	GCA_000463585.1
Amborella trichopoda	GCA_000471905.1
Eutrema salsugineum	GCA_000478725.1
Citrus clementina	GCA_000493195.1
Populus euphratica	GCA_000495115.1
Phaseolus vulgaris	GCA_000499845.1
Erythranthe guttata	GCA_000504015.1
Beta vulgaris subsp. *vulgaris*	GCA_000511025.2
Fragaria x *ananassa*	GCA_000511835.1
Fragaria nubicola	GCA_000511995.1
Fragaria nipponica	GCA_000512025.1
Dianthus caryophyllus	GCA_000512335.1
Sesamum indicum	GCA_000512975.1
Fragaria orientalis	GCA_000517285.1
Oryza punctata	GCA_000573905.2
Oryza nivara	GCA_000576065.2
Oryza glumipatula	GCA_000576495.2
Solanum habrochaites	GCA_000577655.1
Hordeum pubiflorum	GCA_000582825.1
Monoraphidium neglectum	GCA_000611645.1
Gossypium arboreum	GCA_000612285.2

Solanum arcanum	GCA_000612985.1
Camelina sativa	GCA_000633955.1
Brassica napus	GCA_000686985.2
Helicosporidium sp. ATCC 50920	GCA_000690575.1
Brassica oleracea var. *oleracea*	GCA_000695525.1
Aquilaria agallochum	GCA_000696445.1
Klebsormidium nitens	GCA_000708835.1
Capsicum annuum	GCA_000710875.1
Nicotiana otophora	GCA_000715115.1
Nicotiana tabacum	GCA_000715135.1
Auxenochlorella protothecoides	GCA_000733215.1
Penstemon grinnellii	GCA_000737425.1
Penstemon centranthifolius	GCA_000737435.1
Vigna radiata var. *radiata*	GCA_000741045.2
Amaranthus hypochondriacus	GCA_000753965.2
Raphanus raphanistrum	GCA_000769845.1
Vaccinium macrocarpon	GCA_000775335.2
Solanum melongena	GCA_000787875.1
Primula veris	GCA_000788445.1
Raphanus sativus	GCA_000801105.2
Coccomyxa sp. LA000219	GCA_000812005.1
Arachis ipaensis	GCA_000816755.2
Ensete ventricosum	GCA_000818735.3
Trebouxia gelatinosa	GCA_000818905.1
Ziziphus jujuba	GCA_000826755.1
Humulus lupulus var. *lupulus*	GCA_000831365.1
Picochlorum sp. SENEW3	GCA_000876415.1
Catharanthus roseus	GCA_000949345.1
Thlaspi arvense	GCA_000956625.1
Eragrostis tef	GCA_000970635.1
Selaginella kraussiana	GCA_001021135.1
Primula vulgaris	GCA_001077355.1
Oropetium thomaeum	GCA_001182835.1
Zostera marina	GCA_001185155.1
Vigna angularis	GCA_001190045.1
Coccomyxa sp. SUA001	GCA_001244535.1
Cymbomonas tetramitiformis	GCA_001247695.1
Phalaenopsis equestris	GCA_001263595.1
Ocimum tenuiflorum	GCA_001278415.1

Vitis cinerea x *Vitis riparia*	GCA_001282645.1
Vicia faba	GCA_001375635.1
Solanum pennellii	GCA_001406875.2
Juglans regia	GCA_001411555.2
Micromonas sp. ASP10-01a	GCA_001430725.1
Auxenochlorella pyrenoidosa	GCA_001430745.1
Oryza sativa Japonica Group	GCA_001433935.1
Pinus lambertiana	GCA_001447015.2
Arabis montbretiana	GCA_001484125.1
Arabis montbretiana	GCA_001484125.2
Arabis nordmanniana	GCA_001484925.1
Pseudotsuga menziesii	GCA_001517045.1
Cynara cardunculus var. *scolymus*	GCA_001531365.1
Ananas comosus	GCA_001540865.1
Silybum marianum	GCA_001541825.1
Oryza rufipogon	GCA_001551805.1
Vitis aestivalis	GCA_001562795.1
Gonium pectorale	GCA_001584585.1
Dorcoceras hygrometricum	GCA_001598015.1
Zoysia japonica	GCA_001602275.1
Zoysia matrella	GCA_001602295.1
Zoysia pacifica	GCA_001602315.1
Dendrobium catenatum	GCA_001605985.2
Daucus carota subsp. *sativus*	GCA_001625215.1
Coelastrella sp. M60	GCA_001630525.1
Carthamus tinctorius	GCA_001633085.1
Quercus lobata	GCA_001633185.2
Dichanthelium oligosanthes	GCA_001633215.2
Thlaspi arvense	GCA_001642375.1
Pontederia paniculata	GCA_001647135.1
Musa itinerans	GCA_001649415.1
Hevea brasiliensis	GCA_001654055.1
Manihot esculenta	GCA_001659605.1
Metrosideros polymorpha var. *glaberrima*	GCA_001662345.1
Metrosideros polymorpha var. *glaberrima*	GCA_001662345.2
Chlamydomonas applanata	GCA_001662365.1
Chlamydomonas asymmetrica	GCA_001662385.1
Chlamydomonas sphaeroides	GCA_001662425.1
Rosa x *damascena*	GCA_001662545.1

Chenopodium quinoa	GCA_001683475.1
Chenopodium pallidicaule	GCA_001687005.1
Chenopodium suecicum	GCA_001687025.1
Lolium perenne	GCA_001735685.1
Trifolium subterraneum	GCA_001742945.1
Rhazya stricta	GCA_001752375.1
Embelia ribes	GCA_001753735.1
Lupinus angustifolius	GCA_001865875.1
Asparagus officinalis	GCA_001876935.1
Nicotiana attenuata	GCA_001879085.1
Ipomoea nil	GCA_001879475.1
Ruellia speciosa	GCA_001909325.1
Barbarea vulgaris	GCA_001920985.1
Drosera capensis	GCA_001925005.1
Citrus x paradisi x *Citrus trifoliata*	GCA_001929425.1
Cephalotus follicularis	GCA_001972305.1
Capsella bursa-pastoris	GCA_001974645.1
Corchorus capsularis	GCA_001974805.1
Corchorus olitorius	GCA_001974825.1
Momordica charantia	GCA_001995035.1
Botryococcus braunii	GCA_002005505.1
Citrus maxima	GCA_002006925.1
Spinacia oleracea	GCA_002007265.1
Atalantia buxifolia	GCA_002013935.1
Citrus medica	GCA_002013955.2
Citrus cavaleriei	GCA_002013975.2
Asclepias syriaca	GCA_002018285.1
Nicotiana obtusifolia	GCA_002018475.1
Artocarpus camansi	GCA_002024485.1
Porphyra umbilicalis	GCA_002049455.2
Xerophyta viscosa	GCA_002076135.1
Phalaenopsis hybrid cultivar	GCA_002079205.1
Boechera stricta	GCA_002079875.1
Nothapodytes nimmoniana	GCA_002091855.1
Malus domestica	GCA_002114115.1
Trebouxia sp. TZW2008	GCA_002118135.1
Helianthus annuus	GCA_002127325.2
Passiflora edulis	GCA_002156105.1
Triticum dicoccoides	GCA_002162155.2

Herrania umbratica	GCA_002168275.2
Macleaya cordata	GCA_002174775.1
Cenchrus americanus	GCA_002174835.2
Eleusine coracana subsp. *coracana*	GCA_002180455.1
Utricularia gibba	GCA_002189035.1
Kappaphycus alvarezii	GCA_002205965.3
Prunus avium	GCA_002207925.1
Panicum hallii	GCA_002211085.2
Bathycoccus prasinos	GCA_002220235.1
Micractinium conductrix	GCA_002245815.2
Capsicum baccatum	GCA_002271885.2
Capsicum chinense	GCA_002271895.2
Dunaliella salina	GCA_002284615.2
Durio zibethinus	GCA_002303985.1
Kalanchoe fedtschenkoi	GCA_002312845.1
Scenedesmus quadricauda	GCA_002317545.1
Fagopyrum tataricum	GCA_002319775.1
Chlamydomonas eustigma	GCA_002335675.1
Ipomoea batatas	GCA_002525835.2
Rosa multiflora	GCA_002564525.1
Aegilops tauschii	GCA_002575655.1
Coelastrella sp. UTEX B 3026	GCA_002588565.1
Cucurbita maxima	GCA_002738345.1
Cucurbita moschata	GCA_002738365.1
Aquilegia coerulea	GCA_002738505.1
Pachycereus pringlei	GCA_002740445.1
Stenocereus thurberi	GCA_002740465.1
Pereskia humboldtii	GCA_002740485.1
Carnegiea gigantea	GCA_002740515.1
Lophocereus schottii	GCA_002740545.1
Olea europaea var. *sylvestris*	GCA_002742605.1
Viola pubescens var. *scabriuscula*	GCA_002752925.1
Handroanthus impetiginosus	GCA_002762385.1
Apostasia shenzhenica	GCA_002786265.1
Cucurbita pepo subsp. *pepo*	GCA_002806865.2
Kokia drynarioides	GCA_002814295.1
Monoraphidium sp. 549	GCA_002814315.1
Picochlorum sp. *'soloecismus'*	GCA_002818215.1
Monotropa hypopitys	GCA_002855965.1

Lactuca sativa	GCA_002870075.1
Cissus quadrangularis	GCA_002878655.1
Lagenaria siceraria	GCA_002890555.2
Tetrabaena socialis	GCA_002891735.1
Panicum miliaceum	GCA_002895445.2
Cicer echinospermum	GCA_002896215.2
Cicer reticulatum	GCA_002896235.1
Chlorella sp. ArM0029B	GCA_002896455.3
Citrus unshiu	GCA_002897195.1
Eschscholzia californica subsp. *californica*	GCA_002897215.1
Dioscorea alata	GCA_002904275.2
Quercus suber	GCA_002906115.1
Santalum album	GCA_002911635.1
Parasponia andersonii	GCA_002914805.1
Trema orientale	GCA_002914845.1
Juglans mandshurica	GCA_002916435.1
Juglans mandshurica	GCA_002916435.2
Juglans nigra	GCA_002916485.2
Euphorbia esula	GCA_002919075.1
Nicotiana glauca	GCA_002930595.1
Eutrema heterophyllum	GCA_002933915.1
Eutrema yunnanense	GCA_002933935.1
Miscanthus sacchariflorus	GCA_002993905.1
Rosa chinensis	GCA_002994745.2
Phalaenopsis aphrodite	GCA_003013225.1
Liriodendron chinense	GCA_003013855.2
Selaginella tamariscina	GCA_003024785.1
Marchantia polymorpha	GCA_003032435.1
Arachis monticola	GCA_003063285.2
Chlorella sp. A99	GCA_003063905.1
Triticum urartu	GCA_003073215.1
Arachis hypogaea	GCA_003086295.2
Yamagishiella unicocca	GCA_003116995.1
Eudorina sp. 2006-703-Eu-15	GCA_003117195.1
Juglans cathayensis	GCA_003122765.1
Phaseolus coccineus subsp. coccineus	GCA_003122825.1
Pterocarya stenoptera	GCA_003123785.1
Juglans sigillata	GCA_003123805.1
Juglans hindsii	GCA_003123825.1

Juglans microcarpa	GCA_003123845.1
Juglans nigra	GCA_003123865.1
Chlorella sorokiniana	GCA_003130725.1
Gracilariopsis chorda	GCA_003194525.1
Raphidocelis subcapitata	GCA_003203535.1
Dryas drummondii	GCA_003254865.1
Purshia tridentata	GCA_003254885.1
Nissolia schottii	GCA_003254905.1
Chamaecrista fasciculata	GCA_003254925.1
Mimosa pudica	GCA_003254945.1
Alnus glutinosa	GCA_003254965.1
Ochetophila trinervis	GCA_003254975.1
Begonia fuchsioides	GCA_003255005.1
Datisca glomerata	GCA_003255025.1
Casuarina glauca	GCA_003255045.1
Cercis canadensis	GCA_003255065.1
Citrus reticulata	GCA_003258625.1
Silene latifolia	GCA_003260165.1
Argania spinosa	GCA_003260245.1
Cuscuta australis	GCA_003260385.1
Brassica cretica	GCA_003260655.2
Berberis thunbergii	GCA_003290165.1
Penstemon barbatus	GCA_003313485.1
Quillaja saponaria	GCA_003338715.1
Gracilariopsis lemaneiformis	GCA_003346895.1
Eleusine indica	GCA_003369855.1
Mucuna pruriens	GCA_003370565.1
Echium plantagineum	GCA_003412495.2
Chara braunii	GCA_003427395.1
Xanthoceras sorbifolium	GCA_003430845.1
Medicago truncatula	GCA_003473485.2
Trifolium medium	GCA_003490085.1
Saccharum spontaneum	GCA_003544955.1
Cinnamomum micranthum f. *kanehirae*	GCA_003546025.1
Trebouxiophyceae sp. KSI-1	GCA_003568905.1
Magnolia ashei	GCA_003571905.1
Papaver somniferum	GCA_003573695.1
Ipomoea triloba	GCA_003576645.1
Ipomoea trifida	GCA_003576665.1

Picocystis sp. ML	GCA_003665715.1
Pogostemon cablin	GCA_003675935.1
Coffea eugenioides	GCA_003713205.1
Coffea arabica	GCA_003713225.1
Anethum foeniculum	GCA_003724115.1
Anethum foeniculum	GCA_003724115.2
Casuarina equisetifolia subsp. *incana*	GCA_003795335.1
Abrus precatorius	GCA_003935025.1
Morella rubra	GCA_003952965.2
Haematococcus lacustris	GCA_003970955.1
Oryza meyeriana var. *granulata*	GCA_003991445.1
Jaltomata sinuosa	GCA_003996215.1
Eugenia uniflora	GCA_004012085.1
Gossypium thurberi	GCA_004027125.1
Cucurbita argyrosperma	GCA_004115005.1
Mamiellophyceae sp. 2017MT	GCA_004115355.1
Vigna unguiculata	GCA_004118075.1
Alloteropsis semialata	GCA_004135705.1
Ulva prolifera	GCA_004138255.1
Actinidia eriantha	GCA_004150315.1
Larix sibirica	GCA_004151065.1
Glycine soja	GCA_004193775.2
Fagopyrum esculentum	GCA_004303065.2
Perilla frutescens	GCA_004303085.1
Spatholobus suberectus	GCA_004329165.1
Nannochloris sp. X1	GCA_004335555.1
Nannochloris sp. RS	GCA_004335565.1
Haematococcus sp. NG2	GCA_004335575.1
Chloroidium sp. JM	GCA_004335615.1
Chloroidium sp. CF	GCA_004335625.1
Chloromonas sp. AAM2	GCA_004335635.1
Dunaliella sp. WIN1	GCA_004335645.1
Dunaliella sp. YS1	GCA_004335685.1
Chlamydomonas sp. WS7	GCA_004335715.1
Chlorella sp. KRBP	GCA_004335735.1
Chlamydomonas sp. WS3	GCA_004335755.1
Dunaliella sp. RO	GCA_004335775.1
Chlamydomonas sp. 3222	GCA_004335795.1
Scenedesmus sp. ARA3	GCA_004335835.1

Characiochloris sp. AAM3	GCA_004335845.1
Chlamydomonas sp. 3112	GCA_004335865.1
Dunaliella sp. M2	GCA_004335885.1
Chlamydomonas sp. AIC	GCA_004335895.1
Scenedesmus sp. ARA	GCA_004335915.1
Crucihimalaya himalaica	GCA_004349715.1
Vitis riparia	GCA_004353265.1
Chrysanthemum seticuspe	GCA_004359105.1
Mentha longifolia	GCA_004368105.2
Salvia splendens	GCA_004379255.1
Cyanophora paradoxa	GCA_004431415.1
Scenedesmus vacuolatus	GCA_004764505.1
Juglans microcarpa x *Juglans regia*	GCA_004785595.1
Digenea simplex	GCA_004798425.1
Calotropis procera	GCA_004801955.1
Citrus hindsii	GCA_004802465.1
Musa balbisiana	GCA_004837865.1
Caryocar brasiliense	GCA_004918865.1
Lindernia brevidens	GCA_004919715.1
Trichopus zeylanicus subsp. *travancoricus*	GCA_005019695.1
Raddia distichophylla	GCA_005191435.1
Populus alba	GCA_005239225.1
Nicotiana undulata	GCA_005239495.1
Nicotiana paniculata	GCA_005239505.1
Nicotiana knightiana	GCA_005239525.1
Nicotiana rustica	GCA_005239535.1
Setaria viridis	GCA_005286985.1
Aquilaria sinensis	GCA_005392925.1
Gossypium australe	GCA_005393395.2
Prunus yedoensis	GCA_005406145.1
Gossypioides kirkii	GCA_005610355.1
Scutellaria baicalensis	GCA_005771605.1
Beta patula	GCA_005862465.1
Trifolium repens	GCA_005869975.1
Solanum chilense	GCA_006013705.1
Cocos nucifera	GCA_006176705.1
Marchantia inflexa	GCA_006177815.1
Galdieria phlegrea	GCA_006232345.1
Hibiscus syriacus	GCA_006381635.2

Tetraselmis striata	GCA_006384855.1
Sedum album	GCA_006409495.1
Malus baccata	GCA_006547085.1
Chlorella sp. *Dachan*	GCA_006782975.1
Vachellia collinsii	GCA_006871305.1
Pleurozium schreberi	GCA_006891605.1
Carpinus fangiana	GCA_006937295.1
Rosa lucieae	GCA_006954505.1
Sequoiadendron giganteum	GCA_007115665.2
Dactylis glomerata	GCA_007115705.1
Sequoia sempervirens	GCA_007258455.1
Sequoia sempervirens	GCA_007258455.2
Glycine tomentella	GCA_007407185.1
Desmodesmus armatus	GCA_007449985.2
Punica granatum	GCA_007655135.2
Eucalyptus pauciflora	GCA_007663325.1
Eragrostis curvula	GCA_007726485.1
Chlorophyta sp.	GCA_007760615.1
Populus simonii	GCA_007827005.2
Rhamnella rubrinervis	GCA_007844105.2
Pyrus betulifolia	GCA_007844245.1
Chloropicon primus	GCA_007859695.1
Gossypium darwinii	GCA_007990325.1
Gossypium hirsutum	GCA_007990345.1
Acer yangbiense	GCA_008009225.1
Messastrum gracile	GCA_008037345.1
Gossypium turneri	GCA_008044935.1
Persea americana	GCA_008087245.1
Oryza officinalis	GCA_008326285.1
Vitis x *labruscana* x *Vitis vinifera*	GCA_008326845.1
Ficus erecta	GCA_008635985.1
Striga asiatica	GCA_008636005.1
Trebouxia sp. A1-2	GCA_008636185.1
Nyssa sinensis	GCA_008638375.1
Pistacia vera	GCA_008641045.1
Bassia scoparia	GCA_008642245.1
Neoporphyra haitanensis	GCA_008729055.1
Gossypium barbadense	GCA_008761655.1
Sporobolus alterniflorus	GCA_008808055.2

Nymphaea colorata	GCA_008831285.1
Aristotelia chilensis	GCA_008921755.1
Oenanthe javanica	GCA_008931105.1
Rhodoleia championii	GCA_008932045.1
Pyrus ussuriensis x *Pyrus communis*	GCA_008932095.1
Salix brachista	GCA_009078335.1
Chlorokybus atmophyticus	GCA_009103225.1
Mychonastes homosphaera	GCA_009193075.1
Mikania micrantha	GCA_009363875.1
Phoenix dactylifera	GCA_009389715.1
Spirogloea muscicola	GCA_009602725.1
Mesotaenium endlicherianum	GCA_009602735.1
Micromonas sp. SAG5	GCA_009618055.1
Bathycoccus sp. SAG4	GCA_009618065.1
Bathycoccus sp. SAG1	GCA_009618075.1
Bathycoccus sp. SAG3	GCA_009618085.1
Bathycoccus sp. SAG2	GCA_009618095.1
Micromonas sp. SAG6	GCA_009618155.1
Micromonas sp. SAG7	GCA_009618165.1
Micromonas sp. SAG8	GCA_009618195.1
Ostreococcus sp. SAG9	GCA_009618205.1
Ostreococcus sp. SAG10	GCA_009618215.1
Ostreococcus sp. SAG12	GCA_009618245.1
Ostreococcus sp. SAG11	GCA_009618255.1
Picochlorum sp. BH-2019	GCA_009650465.1
Actinidia chinensis	GCA_009663005.1
Chlorella vulgaris	GCA_009720205.1
Fragaria iinumae	GCA_009720345.1
Utricularia reniformis	GCA_009725065.1
Coix aquatica	GCA_009725075.1
Benincasa hispida	GCA_009727055.1
Dioscorea cayenensis subsp. *rotundata*	GCA_009730915.2
Mesostigma viride	GCA_009746045.1
Rhododendron williamsianum	GCA_009746105.1
Lepidium campestre	GCA_009757365.2
Oryza coarctata	GCA_009761635.1
Ficus carica	GCA_009761775.1
Coix lacryma-jobi var. *lacryma-jobi*	GCA_009763385.1
Silene noctiflora	GCA_009801015.1

Moringa oleifera	GCA_009801145.1
Oryza longistaminata	GCA_009805545.1
Andrographis paniculata	GCA_009805555.1
Zostera nigricaulis	GCA_009812395.1
Neopyropia yezoensis	GCA_009829735.1
Picea engelmannii	GCA_009831015.1
Ipomoea purpurea	GCA_009835305.1
Solanum chaucha	GCA_009849625.1
Solanum x *curtilobum*	GCA_009849645.1
Solanum x *juzepczukii*	GCA_009849685.1
Solanum phureja	GCA_009849755.1
Solanum ahanhuiri	GCA_009849805.1
Solanum bukasovii	GCA_009849815.1
Solanum stenotomum	GCA_009849865.1
Rafflesia leonardi	GCA_009866635.1
Ceratopteris richardii	GCA_009866685.1
Toxicodendron radicans	GCA_009867345.1
Solanum pinnatisectum	GCA_009887355.1
Apium graveolens	GCA_009905375.1
Chlorella sp. CH2018	GCA_009928355.1
Stevia rebaudiana	GCA_009936405.1
Ulmus americana	GCA_010015005.2
Triticum urartu	GCA_010110895.1
Fragaria nilgerrensis	GCA_010134655.1
Lupinus albus	GCA_010261695.1
Erigeron canadensis	GCA_010389155.1
Isatis tinctoria	GCA_010577795.1
Tetratostichococcus sp. P1	GCA_010646915.1
Linum usitatissimum	GCA_010665275.1
Linum usitatissimum	GCA_010665275.2
Linum bienne	GCA_010665285.1
Gossypium longicalyx	GCA_010883175.1
Anthoceros angustus	GCA_010909165.1
Picochlorum sp. *'celeri'*	GCA_010909725.1
Populus alba x *Populus glandulosa*	GCA_011022345.1
Carya cathayensis	GCA_011037825.1
Phyllostachys edulis	GCA_011038535.1
Vitis labrusca	GCA_011039315.1
Mangifera indica	GCA_011075055.1

Carex littledalei	GCA_011114355.1
Picochlorum costavermella	GCA_011316045.1
Zingiber officinale	GCA_011317585.1
Erysimum cheiranthoides	GCA_011420285.1
Quercus mongolica	GCA_011696235.1
Isoetes engelmannii	GCA_011763485.1
Nymphaea thermarum	GCA_011799765.1
Thinopyrum elongatum	GCA_011799875.1
Solanum clarkiae	GCA_011800125.1
Puccinellia tenuiflora	GCA_012064385.1
Morus alba	GCA_012066045.3
Sinapis alba	GCA_012274485.2
Galium porrigens var. tenue	GCA_012274505.1
Asparagus setaceus	GCA_012295165.1
Luffa acutangula	GCA_012295215.1
Ostreococcus mediterraneus	GCA_012295225.1
Primulina huaijiensis	GCA_012295235.1
Eragrostis nindensis	GCA_012490785.1
Litsea cubeba	GCA_012931725.1
Dipteryx alata	GCA_012978445.1
Hypericum perforatum	GCA_012979155.1
Trifolium occidentale	GCA_012979555.1
Forsythia suspensa	GCA_013103335.1
Gardenia jasminoides	GCA_013103745.1
Codonopsis lanceolata	GCA_013146195.2
Larix kaempferi	GCA_013171265.2
Boswellia sacra	GCA_013180625.1
Wolffia australiana	GCA_013350325.1
Chimonanthus salicifolius	GCA_013350335.1
Thalictrum thalictroides	GCA_013358455.1
Macadamia integrifolia	GCA_013358625.1
Chlorella sp. BAC 9706	GCA_013372505.1
Phaseolus lunatus	GCA_013389735.1
Hydrangea macrophylla	GCA_013391885.1
Tripterygium wilfordii	GCA_013401445.1
Glycine latifolia	GCA_013407115.1
Prasinococcaceae sp. CCMP 1430	GCA_013416795.1
Vigna mungo	GCA_013427195.1
Chlamydomonas sp. ICE-L	GCA_013435795.1

Gossypium trilobum	GCA_013467465.1
Gossypium lobatum	GCA_013467485.1
Gossypium gossypioides	GCA_013467495.1
Gossypium aridum	GCA_013487665.1
Gossypium laxum	GCA_013511315.1
Aeschynomene evenia	GCA_013621005.1
Petunia axillaris subsp. *parodii*	GCA_013625405.1
Gossypium klotzschianum	GCA_013677235.1
Gossypium davidsonii	GCA_013677245.1
Gossypium harknessii	GCA_013677255.1
Gossypium armourianum	GCA_013677265.1
Gossypium schwendimanii	GCA_013677275.1
Cyanidiococcus yangmingshanensis	GCA_013995675.1
Kingdonia uniflora	GCA_014058105.1
Dioscorea zingiberensis	GCA_014060945.1
Scenedesmus sp. NREL 46B-D3	GCA_014080715.1
Allium sativum	GCA_014155895.1
Allium sativum	GCA_014155895.2
Artemisia annua	GCA_014162995.1
Marchantia paleacea	GCA_014180765.2
Eucalyptus tenuipes	GCA_014182365.1
Eucalyptus virginea	GCA_014182375.1
Eucalyptus viminalis	GCA_014182385.1
Eucalyptus salubris	GCA_014182395.1
Eucalyptus sideroxylon	GCA_014182405.1
Eucalyptus microcorys	GCA_014182515.1
Eucalyptus globulus	GCA_014182545.1
Eucalyptus erythrocorys	GCA_014182555.1
Eucalyptus marginata	GCA_014182565.1
Eucalyptus decipiens	GCA_014182575.1
Eucalyptus coolabah	GCA_014182585.1
Eucalyptus albens	GCA_014182695.1
Eucalyptus camaldulensis	GCA_014182705.1
Eucalyptus cloeziana	GCA_014182715.1
Eucalyptus brandiana	GCA_014182725.1
Corymbia maculata	GCA_014182735.1
Corymbia calophylla	GCA_014182845.1
Eucalyptus regnans	GCA_014182855.1
Eucalyptus caleyi	GCA_014182885.2

Angophora floribunda	GCA_014182895.1
Castanea mollissima	GCA_014183005.1
Colocasia esculenta	GCA_014218235.1
Rhododendron simsii	GCA_014282245.1
Actinidia rufa	GCA_014362265.1
Fagus crenata	GCA_014362285.1
Bathycoccus sp. NIOZ-UU96	GCA_014466055.1
Vaccinium corymbosum	GCA_014504835.1
Cardamine resedifolia	GCA_014578085.1
Diospyros lotus	GCA_014633365.1
Centella asiatica	GCA_014636745.1
Arachis stenosperma	GCA_014773155.1
Diplotaxis tenuifolia	GCA_014822095.1
Arbutus unedo	GCA_014822125.1
Jatropha curcas	GCA_014843425.1
Euphrasia arctica	GCA_014843705.1
Amphicarpaea edgeworthii	GCA_014843725.1
Senna tora	GCA_014851425.1
Corymbia citriodora subsp. variegata	GCA_014858505.1
Cephalotus follicularis	GCA_014871385.1
Populus davidiana	GCA_014885075.1
Jacaranda copaia	GCA_014898725.1
Handroanthus guayacan	GCA_014898755.1
Phtheirospermum japonicum	GCA_014905375.1
Scenedesmus sp. PABB004	GCA_014905635.1
Solanum pimpinellifolium	GCA_014964335.1
Echinochloa crus-galli	GCA_015022175.1
Tetracentron sinense	GCA_015143295.1
Vitis rotundifolia	GCA_015341995.1
Digitaria exilis	GCA_015342445.1
Pycnococcus provasolii	GCA_015473125.1
Crambe hispanica	GCA_015476445.1
Urochloa ruziziensis	GCA_015476505.1
Brassica juncea	GCA_015484525.1
Gnetum montanum	GCA_015680685.1
Coptis chinensis	GCA_015680905.1
Cydonia oblonga	GCA_015708375.1
Populus ilicifolia	GCA_015708895.1
Parachlorella kessleri	GCA_015712045.1

Salix dunnii	GCA_015731905.1
Boechera retrofracta	GCA_015832515.1
Chrysopogon serrulatus	GCA_015844335.1
Hyparrhenia diplandra	GCA_015847255.1
Populus deltoides	GCA_015852605.1
Vitis amurensis	GCA_016071775.1
Rehmannia glutinosa	GCA_016081115.1
Eucalyptus sideroxylon x *Eucalyptus melliodora*	GCA_016097485.1
Eucalyptus victrix	GCA_016097545.1
Eucalyptus pumila	GCA_016097595.1
Eucalyptus guilfoylei	GCA_016097605.1
Eucalyptus dawsonii	GCA_016097615.1
Syntrichia caninervis	GCA_016097705.1
Secale cereale	GCA_016097815.1
Corylus heterophylla	GCA_016403345.1
Vanilla planifolia	GCA_016413895.1
Brassica nigra	GCA_016432835.1
Psidium guajava	GCA_016432845.1
Salvia miltiorrhiza	GCA_016432925.1
Eucalyptus grandis	GCA_016545825.1
Ophiorrhiza pumila	GCA_016586305.1
Pyrus pyrifolia	GCA_016587475.1
Dendrobium huoshanense	GCA_016618105.1
Chlamydomonas sp. UWO 241	GCA_016618255.1
Platycodon grandiflorus	GCA_016624345.1
Eucommia ulmoides	GCA_016647705.1
Gastrodia elata	GCA_016760335.1
Brassica carinata	GCA_016771965.1
Panax notoginseng	GCA_016801055.1
Leptodermis oblonga	GCA_016801395.1
Solanum sitiens	GCA_016801875.1
Suaeda aralocaspica	GCA_016808085.1
Tetrastigma voinierianum	GCA_016808115.1
Sapria himalayana	GCA_016808135.1
Carya illinoinensis	GCA_016808215.1
Panicum virgatum	GCA_016808335.1
Chlamydomonas schloesseri	GCA_016834595.1
Chlamydomonas incerta	GCA_016834605.1

Edaphochlamys debaryana	GCA_016858145.1
Macrotyloma uniflorum	GCA_016859785.1
Puya raimondii	GCA_016920855.1
Vaccinium myrtillus	GCA_016920895.1
Luffa aegyptiaca	GCA_017139565.1
Eucalyptus shirleyi	GCA_017140165.1
Eucalyptus polyanthemos subsp. *polyanthemos*	GCA_017140185.1
Eucalyptus paniculata subsp. *matutina*	GCA_017140255.1
Eucalyptus leucophloia subsp. *euroa*	GCA_017140325.1
Eucalyptus lansdowneana	GCA_017140395.1
Eucalyptus fibrosa	GCA_017140475.1
Eucalyptus curtisii	GCA_017140595.1
Eucalyptus cladocalyx	GCA_017140615.1
Hamamelis virginiana	GCA_017140935.1
Gossypium mustelinum	GCA_017165895.1
Dendrocalamus latiflorus	GCA_017311315.1
Salix suchowensis	GCA_017552425.1
Metroxylon sagu	GCA_017589505.1
Selenicereus undatus	GCA_017589665.1
Hemerocallis citrina	GCA_017893485.1
Akebia trifoliata	GCA_017979445.1
Bretschneidera sinensis	GCA_018105755.1
Datura stramonium	GCA_018107945.1
Rhododendron griersonianum	GCA_018127125.1
Boehmeria nivea var. *tenacissima*	GCA_018132145.1
Themeda triandra	GCA_018135685.1
Citrullus lanatus subsp. *cordophanus*	GCA_018142915.1
Gossypium tomentosum	GCA_018144435.1
Medicago ruthenica	GCA_018208015.1
Gillenia trifoliata	GCA_018257905.1
Solanum commersonii	GCA_018258275.1
Coelastrella sp. MACC-549	GCA_018290735.1
Petunia secreta	GCA_018292955.1
Flaveria bidentis	GCA_018326385.1
Flaveria brownii	GCA_018326445.1
Flaveria floridana	GCA_018326465.1
Flaveria robusta	GCA_018326485.1
Vicia sativa	GCA_018327625.1

Taxus wallichiana var. *yunnanensis*	GCA_018340775.1
Solanum appendiculatum	GCA_018342035.1
Citrus trifoliata	GCA_018350135.1
Boechera stricta	GCA_018361395.1
Asparagopsis taxiformis	GCA_018397955.1
Simmondsia chinensis	GCA_018398585.1
Persea americana	GCA_018408905.1
Arachis cardenasii	GCA_018493915.1
Thuja plicata	GCA_018584345.1
Cucurbita argyrosperma subsp. *sororia*	GCA_018691285.1
Pyramimonas parkeae	GCA_018697155.1
Prunus fruticosa	GCA_018703695.1
Brassica juncea	GCA_018703725.1
Populus tomentosa	GCA_018804465.1
Vigna umbellata	GCA_018835915.1
Jacaranda mimosifolia	GCA_018894105.1
Pugionium cornutum	GCA_018901935.1
Pugionium dolabratum	GCA_018901945.1
Silene uniflora	GCA_018983105.1
Gossypium barbadense	GCA_018997955.1
Fragaria x *ananassa*	GCA_019022445.1
Quercus aquifolioides	GCA_019022515.1
Acacia melanoxylon	GCA_019022615.1
Acacia acuminata	GCA_019022655.1
Chlorella desiccata (nom. nud.)	GCA_019044685.2
Pueraria montana var. *thomsonii*	GCA_019096045.1
Vigna mungo var. *mungo*	GCA_019096145.1
Fraxinus excelsior	GCA_019097785.1
Psidium friedrichsthalianum	GCA_019149425.1
Avicennia marina	GCA_019155195.1
Agarophyton vermiculophyllum	GCA_019155205.1
Lycium barbarum	GCA_019175385.1
Lolium multiflorum	GCA_019182485.1
Solanum stenotomum	GCA_019186545.1
Populus x *sibirica*	GCA_019202695.1
Actinidia eriantha	GCA_019202715.1
Arabidopsis thaliana x *Arabidopsis arenosa*	GCA_019202795.1
Arabidopsis suecica	GCA_019202805.1
Stipa capillata	GCA_019208055.1

Zizania palustris	GCA_019279435.1
Apostasia ramifera	GCA_019297695.1
Miscanthus floridulus	GCA_019320115.1
Paulownia fortunei	GCA_019321725.1
Pharus latifolius	GCA_019359835.1
Lolium perenne	GCA_019359855.1
Osmanthus fragrans	GCA_019395295.1
Amaranthus cruentus	GCA_019425755.1
Buddleja alternifolia	GCA_019426215.1
Zanthoxylum bungeanum	GCA_019454045.1
Gossypium anomalum	GCA_019455425.1
Perilla frutescens var. *frutescens*	GCA_019511825.2
Dendrobium officinale	GCA_019514585.1
Apocynum venetum	GCA_019593545.1
Elaeagnus angustifolia	GCA_019593565.1
Volvox africanus	GCA_019650175.1
Volvox reticuliferus	GCA_019650235.1
Xylocarpus granatum	GCA_019650275.1
Rhododendron kiyosumense	GCA_019656275.1
Rhododendron mucronatum var. *ripense*	GCA_019656295.1
Rhododendron ovatum	GCA_019656835.1
Hibiscus mutabilis	GCA_019671005.1
Asterochloris erici	GCA_019693375.1
Ettlia oleoabundans	GCA_019693395.1
Axilococcus clingmanii	GCA_019693495.1
Cyanidium caldarium	GCA_019693505.1
Prasinocladus sp. *malaysianus*	GCA_019702555.1
Raphanus raphanistrum x *Raphanus sativus*	GCA_019705995.1
Raphanus raphanistrum subsp. *raphanistrum*	GCA_019706035.1
Lithospermum erythrorhizon	GCA_019722365.1
Passiflora organensis	GCA_019739055.1
Wolffia australiana	GCA_019775775.1
Sarracenia alata	GCA_019775975.1
Brassica rapa x *Raphanus sativus*	GCA_019776045.1
Amaranthus palmeri	GCA_019776075.1
Eucalyptus melliodora	GCA_019776745.1
Bruguiera parviflora	GCA_019804595.1
Aristolochia fimbriata	GCA_019845555.1
Typha latifolia	GCA_019914945.1

Acanthochlamys bracteata	GCA_019914995.1
Eucalyptus camaldulensis	GCA_019915185.1
Eucalyptus rudis	GCA_019915205.1
Vitellaria paradoxa	GCA_019916065.1
Picea sitchensis	GCA_019925245.1
Litchi chinensis	GCA_019925255.1
Dendrobium chrysotoxum	GCA_019925795.1
Melaleuca alternifolia	GCA_019926035.1
Castanospermum australe	GCA_019955255.1
Chrysanthemum seticuspe	GCA_019973895.1
Taxus chinensis	GCA_019985065.1
Artocarpus heterophyllus	GCA_020010975.1
Gynochthodes officinalis	GCA_020080225.1
Tilia amurensis	GCA_020138205.1
Bruguiera sexangula	GCA_020141445.1
Panax japonicus	GCA_020205505.1
Panax stipuleanatus	GCA_020205555.1
Panax ginseng	GCA_020205605.1
Panax quinquefolius	GCA_020205615.1
Sindora glabra	GCA_020226215.1
Prunus davidiana	GCA_020226225.1
Prunus mira	GCA_020226265.1
Prunus salicina	GCA_020226455.1
Alliaria petiolata	GCA_020283515.1
Trifolium pratense	GCA_020283565.1
Ceratopteris richardii	GCA_020310875.1
Dimocarpus longan	GCA_020457875.1
Centrapalus pauciflorus	GCA_020465985.1
Echinochloa crus-galli	GCA_020466025.1
Gossypium stocksii	GCA_020496765.1
Forsythia suspensa	GCA_020510225.1
Prunus kanzakura	GCA_020521455.1
Shorea leprosula	GCA_020521535.1
Gynostemma pentaphyllum	GCA_020536105.1
Rhododendron henanense subsp. *lingbaoense*	GCA_020567845.1
Ceriops decandra	GCA_020615895.1
Ceriops zippeliana	GCA_020617695.1
Saccharum officinarum	GCA_020631735.1
Cenchrus americanus	GCA_020739535.1

Malus sieversii	GCA_020795835.1
Eperua falcata	GCA_900006205.1
Sonneratia caseolaris	GCA_900006705.1
Coffea canephora	GCA_900059795.1
Picea abies	GCA_900067695.1
Hordeum bulbosum	GCA_900070015.1
Arabidopsis halleri subsp. *gemmifera*	GCA_900078215.1
Secale cereale	GCA_900079665.1
Tetradesmus obliquus	GCA_900108755.1
Conringia planisiliqua	GCA_900108845.1
Euclidium syriacum	GCA_900116095.1
Bathycoccus sp. TOSAG39-1	GCA_900128745.1
Arabis alpina	GCA_900128785.1
Fraxinus excelsior	GCA_900149125.1
Rhizophora apiculata	GCA_900174605.1
Avicennia marina	GCA_900174615.1
Sonneratia alba	GCA_900174645.1
Betula pendula	GCA_900184695.1
Solanum verrucosum	GCA_900185275.1
Solanum americanum	GCA_900188915.1
Triticum turgidum subsp. *durum*	GCA_900231445.1
Geum urbanum	GCA_900236755.1
Quercus robur	GCA_900291515.1
Trifolium pratense	GCA_900292005.1
Kewa caespitosa	GCA_900322205.1
Simmondsia chinensis	GCA_900322235.1
Microtea debilis	GCA_900322245.1
Macarthuria australis	GCA_900322265.1
Pharnaceum exiguum	GCA_900322385.1
Cuscuta campestris	GCA_900332095.2
Odontarrhena argentea	GCA_900406245.1
Anastatica hierochuntica	GCA_900406275.1
Biscutella auriculata	GCA_900406285.1
Aurinia saxatilis	GCA_900406295.1
Cochlearia officinalis	GCA_900406305.1
Biscutella laevigata subsp. *laevigata*	GCA_900406315.1
Erucastrum elatum	GCA_900406325.1
Boechera puberula	GCA_900406335.1
Erysimum cheiri	GCA_900406345.1

Erysimum pusillum	GCA_900406355.1
Heliophila coronopifolia	GCA_900406365.1
Iberis amara	GCA_900406375.1
Kernera saxatilis	GCA_900406395.1
Lepidium africanum	GCA_900406405.1
Isatis lusitanica	GCA_900406415.1
Iberis pinnata	GCA_900406425.1
Lepidium aucheri	GCA_900406435.1
Nasturtium officinale	GCA_900406445.1
Macropodium nivale	GCA_900406455.1
Noccaea caerulescens	GCA_900406465.1
Noccaea goesingensis	GCA_900406475.1
Physaria acutifolia	GCA_900406485.1
Sisymbrium altissimum	GCA_900406495.1
Physaria ovalifolia	GCA_900406505.1
Physaria fendleri	GCA_900406525.1
Raparia bulbosa	GCA_900406535.1
Pseudoturritis turrita	GCA_900406555.1
Turritis glabra	GCA_900406565.1
Musa schizocarpa	GCA_900464855.1
Saccharum hybrid cultivar	GCA_900465005.1
Calamus simplicifolius	GCA_900491605.1
Spirodela polyrhiza	GCA_900492545.1
Vitis riparia x *Vitis cinerea*	GCA_900538005.1
Silphium perfoliatum	GCA_900538075.1
Ulva mutabilis	GCA_900538255.1
Cannabis sativa	GCA_900626175.2
Dioscorea sansibarensis	GCA_900631875.1
Rhodamnia argentea	GCA_900635035.1
Syzygium oleosum	GCA_900635055.1
Pisum sativum	GCA_900700895.2
Corrigiola litoralis	GCA_901111285.1
Limeum aethiopicum	GCA_901111325.1
Spergula arvensis	GCA_901111335.1
Zea mays	GCA_902167145.1
Prunus dulcis	GCA_902201215.1
Arabis nemorensis	GCA_902206195.3
Lathyrus sativus	GCA_902207245.1
Dioscorea dumetorum	GCA_902712375.1

Microthlaspi erraticum	GCA_902728155.2
Spirodela intermedia	GCA_902729315.2
Tetradesmus acuminatus	GCA_902809745.2
Fraxinus latifolia	GCA_902829145.1
Fraxinus dipetala	GCA_902829195.1
Fraxinus paxiana	GCA_902829205.1
Fraxinus velutina	GCA_902837555.1
Lactuca saligna	GCA_902860255.1
Fraxinus sieboldiana	GCA_902876705.1
Prunus armeniaca	GCA_903112645.1
Fraxinus mandshurica	GCA_903772985.1
Fraxinus americana	GCA_903798225.1
Fraxinus ornus	GCA_903798235.1
Fraxinus pennsylvanica	GCA_903798245.1
Fraxinus quadrangulata	GCA_903798255.1
Fraxinus angustifolia subsp. *syriaca*	GCA_903798275.1
Fraxinus gooddingii	GCA_903798285.1
Fraxinus nigra	GCA_903798295.1
Fraxinus griffithii	GCA_903798305.1
Fraxinus greggii	GCA_903798315.1
Fraxinus xanthoxyloides	GCA_903798325.1
Fraxinus albicans	GCA_903798335.1
Fraxinus anomala	GCA_903798345.1
Fraxinus baroniana	GCA_903798355.1
Fraxinus sp. 1973-6204	GCA_903798365.1
Fraxinus sp. D2006-0159	GCA_903798375.1
Fraxinus cuspidata	GCA_903798385.1
Fraxinus floribunda	GCA_903798395.1
Fraxinus platypoda	GCA_903798405.1
Mesua ferrea	GCA_904814375.1
Miscanthus lutarioriparius	GCA_904845875.1
Hordeum vulgare subsp. vulgare	GCA_904849725.1
Moricandia arvensis	GCA_905132765.1
Moricandia moricandioides	GCA_905132885.1
Ostreobium quekettii	GCA_905146915.1
Arabidopsis suecica	GCA_905175345.1
Arabidopsis arenosa subsp. *arenosa*	GCA_905175405.1
Ilex paraguariensis	GCA_905181385.1
Allium cepa	GCA_905187595.1

Impatiens glandulifera	GCA_907164915.1
Fagus sylvatica	GCA_907173295.1
Azolla filiculoides	GCA_910574055.2
Azolla mexicana	GCA_910574105.1
Azolla rubra	GCA_910574125.1
Azolla caroliniana	GCA_910574225.1
Azolla microphylla	GCA_910574305.1
Azolla nilotica	GCA_910574485.1
Oryza australiensis	GCA_911865555.1
Fraxinus pennsylvanica	GCA_912172775.1
Dunaliella primolecta	GCA_914767535.1
Arctostaphylos glauca	GCA_916048215.1
Arabidopsis thaliana	GCF_000001735.4
Physcomitrium patens	GCF_000002425.4
Chlamydomonas reinhardtii	GCF_000002595.2
Populus trichocarpa	GCF_000002775.4
Citrus sinensis	GCF_000003195.3
Sorghum bicolor	GCF_000003745.3
Gossypium raimondii	GCF_000004075.3
Vitis vinifera	GCF_000004255.2
Cucumis sativus	GCF_000004515.6
Arabidopsis lyrata subsp. *lyrata*	GCF_000005505.3
Cicer arietinum	GCF_000090985.2
Cyanidioschyzon merolae	GCF_000091205.1
Cajanus cajan	GCF_000092065.1
Glycine max	GCF_000143415.4
Prunus persica	GCF_000143455.1
Prunus mume	GCF_000147415.1
Brachypodium distachyon	GCF_000150535.2
Micromonas commoda	GCF_000151265.2
Ostreococcus lucimarinus CCE9901	GCF_000151685.1
Nelumbo nucifera	GCF_000184155.1
Capsella rubella	GCF_000188115.4
Selaginella moellendorffii	GCF_000208745.1
Volvox carteri f. *nagariensis*	GCF_000214015.3
Nicotiana tomentosiformis	GCF_000226075.1
Chlorella variabilis	GCF_000231095.2
Nicotiana sylvestris	GCF_000258705.1
Morus notabilis	GCF_000263155.2

Carica papaya	GCF_000309985.2
Elaeis guineensis	GCF_000313045.1
Tarenaya hassleriana	GCF_000313855.2
Amborella trichopoda	GCF_000315295.1
Eutrema salsugineum	GCF_000317415.1
Micromonas pusilla CCMP1545	GCF_000327365.1
Citrus clementina	GCF_000331145.1
Ricinus communis	GCF_000340665.1
Galdieria sulphuraria	GCF_000341285.1
Populus euphratica	GCF_000346465.2
Phaseolus vulgaris	GCF_000346735.1
Chondrus crispus	GCF_000350225.1
Fragaria vesca subsp. *vesca*	GCF_000365185.1
Erythranthe guttata	GCF_000375325.1
Beta vulgaris subsp. *vulgaris*	GCF_000390325.2
Sesamum indicum	GCF_000393655.1
Solanum lycopersicum	GCF_000414095.1
Monoraphidium neglectum	GCF_000442705.1
Gossypium arboreum	GCF_000463585.1
Theobroma cacao	GCF_000471905.2
Camelina sativa	GCF_000478725.1
Brassica napus	GCF_000493195.1
Brassica oleracea var. *oleracea*	GCF_000495115.1
Capsicum annuum	GCF_000499845.1
Nicotiana tabacum	GCF_000504015.1
Auxenochlorella protothecoides	GCF_000511025.2
Vigna radiata var. *radiata*	GCF_000512975.1
Raphanus sativus	GCF_000611645.1
Ostreococcus tauri	GCF_000612285.1
Arachis ipaensis	GCF_000633955.1
Arachis duranensis	GCF_000686985.2
Ziziphus jujuba	GCF_000695525.1
Vigna angularis	GCF_000710875.1
Phalaenopsis equestris	GCF_000715135.1
Solanum pennellii	GCF_000733215.1
Solanum tuberosum	GCF_000741045.1
Oryza brachyantha	GCF_000801105.1
Juglans regia	GCF_000816755.2
Oryza sativa Japonica Group	GCF_000817695.2

Cynara cardunculus var. *scolymus*	GCF_000826755.1
Ananas comosus	GCF_001190045.1
Dendrobium catenatum	GCF_001263595.1
Coccomyxa subellipsoidea C-169	GCF_001406875.1
Daucus carota subsp. *sativus*	GCF_001411555.2
Quercus lobata	GCF_001433935.1
Setaria italica	GCF_001531365.1
Hevea brasiliensis	GCF_001540865.1
Manihot esculenta	GCF_001605985.2
Chenopodium quinoa	GCF_001625215.1
Brassica rapa	GCF_001633185.2
Lupinus angustifolius	GCF_001654055.1
Asparagus officinalis	GCF_001659605.2
Nicotiana attenuata	GCF_001683475.1
Ipomoea nil	GCF_001865875.1
Momordica charantia	GCF_001876935.1
Spinacia oleracea	GCF_001879085.1
Malus domestica	GCF_001879475.1
Helianthus annuus	GCF_001995035.1
Triticum dicoccoides	GCF_002007265.1
Herrania umbratica	GCF_002114115.1
Prunus avium	GCF_002127325.2
Cucumis melo	GCF_002162155.1
Musa acuminata subsp. *malaccensis*	GCF_002168275.1
Panicum hallii	GCF_002207925.1
Pyrus x *bretschneideri*	GCF_002211085.1
Bathycoccus prasinos	GCF_002220235.1
Durio zibethinus	GCF_002303985.1
Cucurbita maxima	GCF_002738345.1
Cucurbita moschata	GCF_002738365.1
Olea europaea var. *sylvestris*	GCF_002742605.1
Cucurbita pepo subsp	GCF_002806865.1
Lactuca sativa	GCF_002870075.2
Quercus suber	GCF_002906115.1
Rosa chinensis	GCF_002994745.2
Arachis hypogaea	GCF_003086295.2
Medicago truncatula	GCF_003473485.1
Papaver somniferum	GCF_003573695.1
Ipomoea triloba	GCF_003576645.1

Coffea eugenioides	GCF_003713205.1
Coffea arabica	GCF_003713225.1
Abrus precatorius	GCF_003935025.1
Vigna unguiculata	GCF_004118075.1
Camellia sinensis	GCF_004153795.1
Glycine soja	GCF_004193775.1
Vitis riparia	GCF_004353265.1
Salvia splendens	GCF_004379255.1
Juglans microcarpa x *Juglans regia*	GCF_004785595.1
Prosopis alba	GCF_004799145.1
Populus alba	GCF_005239225.1
Setaria viridis	GCF_005286985.1
Hibiscus syriacus	GCF_006381635.1
Punica granatum	GCF_007655135.1
Gossypium hirsutum	GCF_007990345.1
Pistacia vera	GCF_008641045.1
Nymphaea colorata	GCF_008831285.1
Phoenix dactylifera	GCF_009389715.1
Benincasa hispida	GCF_009727055.1
Dioscorea cayenensis subsp. *rotundata*	GCF_009730915.1
Erigeron canadensis	GCF_010389155.1
Mangifera indica	GCF_011075055.1
Macadamia integrifolia	GCF_013358625.1
Tripterygium wilfordii	GCF_013401445.1
Jatropha curcas	GCF_014843425.1
Eucalyptus grandis	GCF_016545825.1
Panicum virgatum	GCF_016808335.1
Triticum aestivum	GCF_018294505.1
Zingiber officinal	GCF_018446385.1
Carya illinoinensis	GCF_018687715.1
Telopea speciosissima	GCF_018873765.1
Cannabis sativa	GCF_900626175.2
Rhodamnia argentea	GCF_900635035.1
Syzygium oleosum	GCF_900635055.1
Zea mays	GCF_902167145.1
Prunus dulcis	GCF_902201215.1
Hordeum vulgare	GCF_904849725.1
Astrephomene gubernaculifera	AP024562.1
Coccomyxa sp. Obi	AP025007.1

Phoenix roebelenii	BK059358.1
Picochlorum sp. *'soloecismus'* DOE 101	CM008992.1
Prototheca wickerhamii HMC1	CM009948.1
Avicennia marina subsp. *marina*	CM023205.1
Ziziphus jujuba var. *spinosa*	CM036902.1
Picocystis sp. L7	CM037205.1
Gracilaria domingensis	CM040115.1
Picochlorum sp. BPE23	CM046021.1
Murraya koenigii	CM046829.1
Hirschfeldia incana	CM048459.1
Dysphania ambrosioides	CM048684.1
Salvia hispanica	CM056166.1
Oryza sativa Indica Group	CP018169.1
Gracilariopsis andersonii	HQ586060.1
Cyanidioschyzon merolae 10D-T	LC519602.1
Closterium sp. NIES-68	LC704714.1
Closterium sp. NIES-67	LC704716.1
Olea europaea subsp. *europaea*	LR743801.1
Conocephalum conicum	MK230928.1
Monosolenium tenerum	MK230931.1
Lactuca sativa	MK642355.1
Marchantia paleacea	NC_001660.1
Physcomitrella patens	NC_007945.1
Megaceros aenigmaticus	NC_012651.1
Pleurozia purpurea	NC_013444.1
Phaeoceros laevis	NC_013765.1
Gracilariophila oryzoides	NC_014771.1/HQ586059.1
Anomodon rugelii	NC_016121.1
Treubia lacunosa	NC_016122.1
Liriodendron tulipifera	NC_021152.1/KC821969.1
Anomodon attenuatus	NC_021931.1
Tetraphis pellucida	NC_024290.1
Ptychomnion cygnisetum	NC_024514.1
Climacium americanum	NC_024515.1
Hypnum imponens	NC_024516.1
Buxbaumia aphylla	NC_024518.1
Bartramia pomiformis	NC_024519.1
Atrichum angustatum	NC_024520.1
Sphagnum palustre	NC_024521.1

Orthotrichum stellatum	NC_024522.1
Funaria hygrometrica	NC_024523.1
Codriophorus laevigatus	NC_025931.1
Orthotrichum speciosum	NC_026121.1
Leiosporoceros dussii	NC_039751.1
Wiesnerella denudata	NC_053538.1
Malus sylvestris	NC_065226.1
Theobroma grandiflorum	ON652864.1
Potentilla anserina	OW176988.1
Polygonum aviculare	OW204033.1
Misopates orontium	OX326954.1
Luzula sylvatica	OX326962.1
Medicago arabica	OX326972.1
Juncus effusus	OX326995.1
Ailanthus altissimus	OX327714.1
Chamaenerion angustifolium	OX328283.1
Scutellaria galericulata	OX335799.1
Ballota nigra	OX344731.1
Solanum dulcamara	OX381602.1
Pulicaria dysenterica	OX381670.1
Hedera helix	OX381723.1
Sherardia arvensis	OX415246.1
Linaria vulgaris	OX415255.1
Chenopodium album	OX419235.1
Crepidotus sp. BD-2015	GCA_001179765.1
Metschnikowia sp. 01-655c1	GCA_002370185.1
Metschnikowia proteae	GCA_002370515.1
Metschnikowia sp. 00-154.1	GCA_002370645.1
Gomphus sp. MG54	GCA_003314385.1
Metschnikowia sp. UFMG-CM-y6306	GCA_009756535.1
Metschnikowia sp. UWOPS 03-167b3	GCA_009756565.1
Tulosesus angulatus	GCA_013368325.1
Entomortierella lignicola	GCA_015500005.1
Linnemannia schmuckeri	GCA_015502505.1
Entomortierella beljakovae	GCA_015679585.1
Lunasporangiospora selenospora	GCA_015680635.1
Linnemannia zychae	GCA_015847235.1
Entomortierella chlamydospora	GCA_015849335.1
Linnemannia exigua	GCA_016097895.1

Blastomyces dermatitidis ER-3	GCA_000003525.2
Lachancea kluyveri	GCA_000149225.1
Rhizopus delemar RA 99-880	GCA_000149305.1
Trichophyton equinum CBS 127.97	GCA_000151175.1
Allomyces macrogynus ATCC 38327	GCA_000151295.1
Trichophyton tonsurans CBS 112818	GCA_000151455.1
Saccharomyces mikatae IFO 1815	GCA_000166975.1
Lachancea waltii NCYC 2644	GCA_000167115.1
Kluyveromyces wickerhamii UCD 54-210	GCA_000179415.1
Magnaporthiopsis poae ATCC 64411	GCA_000193285.1
Erysiphe pisi	GCA_000208805.1
Periglandula ipomoeae IasaF13	GCA_000222875.2
Epichloe gansuensis E7080	GCA_000222895.2
Epichloe brachyelytri E4804	GCA_000222915.1
Aciculosporium take MAFF-241224	GCA_000222935.2
Claviceps fusiformis PRL 1980	GCA_000223055.1
Epichloe amarillans E57	GCA_000223075.2
Zymoseptoria pseudotritici STIR04_4.3.1	GCA_000223685.2
Zymoseptoria ardabiliae STIR04_1.1.2	GCA_000223765.2
Zymoseptoria passerinii SP63	GCA_000223825.2
Epichloe glyceriae E277	GCA_000225285.2
Homolaphlyctis polyrhiza JEL 142	GCA_000235945.1
Mortierella alpina ATCC 32222	GCA_000240685.2
Cladosporium sphaerospermum UM 843	GCA_000261425.2
Pleosporales sp. UM 1110	GCA_000263175.2
Ganoderma lucidum G.260125-1	GCA_000271565.1
Fusarium oxysporum NRRL 32931	GCA_000271745.2
Sphaerulina populicola P02.02b	GCA_000291705.1
Omphalotus olearius VT 653.13	GCA_000296255.1
Serendipita indica DSM 11827	GCA_000313545.1
Fulvia fulva CBS 131901	GCA_000301015.1
Epichloe typhina E8	GCA_000308955.1
Epichloe gansuensis var. *inebrians* E818	GCA_000309355.1
Gelatoporia subvermispora B	GCA_000320605.2
Herpotrichiellaceae sp. UM238	GCA_000315175.1
Thermomyces lanuginosus SSBP	GCA_000315935.1
Metschnikowia fructicola 277	GCA_000317355.2
Ophiostoma novo-ulmi subsp. *novo-ulmi* H327	GCA_000317715.1

Ganoderma multipileum BCRC 37180	GCA_000338015.1
Saccharomyces cerevisiae x *Saccharomyces kudriavzevii* VIN7	GCA_000326105.1
Ascocoryne sarcoides NRRL 50072	GCA_000328965.1
Trichoderma hamatum GD12	GCA_000331835.2
Trichoderma longibrachiatum SMF2	GCA_000332775.1
Wolfiporia cocos MD-104 SS10	GCA_000344635.1
Dothistroma septosporum NZE10	GCA_000340195.1
Fomitopsis pinicola FP-58527 SS1	GCA_000344655.2
Volvariella volvacea V23	GCA_000349905.1
Candida maltosa Xu316	GCA_000344705.1
Penicillium oxalicum 114-2	GCA_000346795.1
Claviceps purpurea 20.1	GCA_000347355.1
Penicillium paxilli ATCC 26601	GCA_000347475.1
Cercospora canescens BHU	GCA_000347735.1
Leucoagaricus gongylophorus Ac12	GCA_000382605.1
Colletotrichum orbiculare MAFF 240422	GCA_000350065.2
Pyrenochaeta sp. UM 256	GCA_000359685.2
Amanita jacksonii TRTC168611	GCA_000497225.1
Ophiostoma piceae UAMH 11346	GCA_000410735.1
Pecoramyces ruminantium	GCA_000412615.1
Rozella allomycis CSF55	GCA_000442015.1
Gyalolechia flavorubescens KoLRI002931	GCA_000442125.1
Rhizoctonia solani AG-3 Rhs1AP	GCA_000524645.1
Zygosaccharomyces bailii CLIB 213	GCA_000442885.1
Cladonia macilenta KoLRI003786	GCA_000444155.1
Flammulina velutipes KACC42780	GCA_000633125.1
Galerina marginata CBS 339.88	GCA_000697645.1
Pyrenophora seminiperda CCB06	GCA_000465215.2
Hysterium pulicare CBS 123377	GCA_000467715.1
Rhytidhysteron rufulum CBS 306.38	GCA_000467735.1
Hirsutella thompsonii MTCC6686	GCA_000472125.2
Cladonia metacorallifera KoLRI002260	GCA_000482085.2
Jaapia argillacea MUCL 33604	GCA_000697665.1
Fusarium circinatum	GCA_000497325.3
Spathaspora arborariae UFMG-19.1A	GCA_000497715.1
Shiraia sp. slf14	GCA_000498155.1
Helminthosporium solani B-AC-16A	GCA_000498615.1
Botryobasidium botryosum FD-172 SS1	GCA_000697705.1

Lignosus rhinocerotis TM02	GCA_000743315.1
Sclerotinia borealis F-4128	GCA_000503235.1
Pseudocercospora pini-densiflorae CBS 125139	GCA_000504365.2
Exutisphaerella laricina CBS 326.52	GCA_000504385.2
Mycosphaerella sp. Ston1	GCA_000504405.2
Cryomyces antarcticus CCFEE 534	GCA_000504465.1
Trametes cinnabarina	GCA_000765035.1
Penicillium roqueforti FM164	GCA_000513255.1
Phlebopus portentosus	GCA_000766925.2
Taiwanofungus camphoratus	GCA_000766995.1
Drechslerella stenobrocha 248	GCA_000525045.1
Aspergillus sclerotiorum	GCA_000530345.1
Penicillium fuscoglaucum FM041	GCA_000576735.1
Penicillium carneum LCP05634	GCA_000577495.1
Penicillium paneum FM227	GCA_000577715.1
Penicillium biforme FM169	GCA_000577785.1
Hanseniaspora vineae	GCA_000585475.3
Fusarium virguliforme Mont-1	GCA_000585705.1
Mucor irregularis B50	GCA_000587855.1
Aspergillus ruber CBS 135680	GCA_000600275.1
Neurospora sublineolata FGSC 5508	GCA_000604185.2
Neurospora africana FGSC 1740	GCA_000604205.2
Neurospora pannonica FGSC 7221	GCA_000604225.2
Neurospora terricola FGSC 1889	GCA_000604245.2
Rhizomucor miehei CAU432	GCA_000611695.1
Ochroconis constricta UM 578	GCA_000611715.1
Umbilicaria muehlenbergii	GCA_000611775.1
Trichophyton soudanense CBS 452.61	GCA_000616865.1
Trichophyton interdigitale MR816	GCA_000622975.1
Lentinus polychrous	GCA_000787475.1
Diplodia sapinea CMW 190	GCA_000671355.1
Ustilaginoidea virens	GCA_000687475.1
Mucor velutinosus B5328	GCA_000696895.1
Apophysomyces trapeziformis B9324	GCA_000696975.1
Apophysomyces elegans B7760	GCA_000696995.1
Cunninghamella elegans B9769	GCA_000697015.1
Rhizopus stolonifer B9770	GCA_000697035.1
Saksenaea vasiformis B4078	GCA_000697055.1

Rhizopus arrhizus	GCA_000697195.1
Cunninghamella bertholletiae 175	GCA_000697215.1
Cokeromyces recurvatus B5483	GCA_000697235.1
Mucor racemosus B9645	GCA_000697255.1
Mucor indicus B7402	GCA_000697295.1
Conidiobolus incongruus B7586	GCA_000697335.1
Syncephalastrum monosporum B8922	GCA_000697355.1
Umbelopsis isabellina B7317	GCA_000697415.1
Basidiobolus heterosporus B8920	GCA_000697455.1
Lichtheimia corymbifera B2541	GCA_000697475.1
Saksenaea oblongispora B3353	GCA_000697495.1
Hydnomerulius pinastri MD-312	GCA_000827185.1
Laccaria amethystina LaAM-08-1	GCA_000827195.1
Plicaturopsis crispa FD-325 SS-3	GCA_000827205.1
Rhizopus arrhizus 99-892	GCA_000697725.1
Balansia obtecta B249	GCA_000709145.1
Penicillium chrysogenum	GCA_000710275.1
Eremothecium coryli CBS 5749	GCA_000710315.1
Sporothrix pallida	GCA_000710705.2
Huntiella moniliformis	GCA_000712465.1
Lichtheimia corymbifera JMRC:FSU:9682	GCA_000723665.1
Epichloe sp. AL9924	GCA_000729825.1
Atkinsonella hypoxylon	GCA_000729835.1
Epichloe baconii ATCC 200745	GCA_000729845.1
Epichloe aotearoae	GCA_000729855.1
Hypocrella siamensis	GCA_000731825.1
Verticillium tricorpus MUCL 9792	GCA_000732205.1
Stachybotrys chlorohalonata IBT 40285	GCA_000732775.1
Penicillium nordicum	GCA_000733025.2
Falciphora oryzae	GCA_000733355.1
Cordyceps farinosa	GCA_000733625.1
Beauveria rudraprayagi	GCA_000733645.1
Venturia pyrina ICMP 11032	GCA_000738655.1
Podila verticillata NRRL 6337	GCA_000739165.1
Sphaerobolus stellatus SS14	GCA_000827215.1
Curvularia lunata CX-3	GCA_000743335.1
Scytalidium sp. 3C	GCA_000743665.3
Tolypocladium sp. *Sup5* PDA-1	GCA_000750105.3
Tolypocladium sp. *Salcha* MEA-2	GCA_000750145.2

Pseudogymnoascus sp. VKM F-3557	GCA_000750665.1
Pseudogymnoascus sp. VKM F-3808	GCA_000750675.1
Pseudogymnoascus sp. VKM F-3775	GCA_000750715.1
Pseudogymnoascus sp. VKM F-4246	GCA_000750735.1
Pseudogymnoascus sp. VKM F-4281 (FW-2241)	GCA_000750745.1
Pseudogymnoascus sp. VKM F-4513 (FW-928)	GCA_000750755.1
Pseudogymnoascus sp. VKM F-4514 (FW-929)	GCA_000750795.1
Pseudogymnoascus sp. VKM F-4515 (FW-2607)	GCA_000750805.1
Pseudogymnoascus sp. VKM F-4516 (FW-969)	GCA_000750815.1
Pseudogymnoascus sp. VKM F-4517 (FW-2822)	GCA_000750875.1
Pseudogymnoascus sp. VKM F-103	GCA_000750895.1
Pseudogymnoascus sp. VKM F-4518 (FW-2643)	GCA_000750925.1
Pseudogymnoascus sp. VKM F-4519 (FW-2642)	GCA_000750935.1
Pseudogymnoascus sp. VKM F-4520 (FW-2644)	GCA_000750995.1
Daldinia eschscholtzii	GCA_000751375.2
Meyerozyma caribbica MG20W	GCA_000755205.1
Suillus luteus UH-Slu-Lm8-n1	GCA_000827255.1
Gymnopus luxurians FD-317 M1	GCA_000827265.1
Pisolithus microcarpus 441	GCA_000827275.1
Acremonium chrysogenum ATCC 11550	GCA_000769265.1
Fusarium avenaceum	GCA_000769295.1
Piloderma croceum F 1598	GCA_000827315.1
Evansstolkia leycettana	GCA_000787455.1
Thermomucor indicae-seudaticae	GCA_000787465.1
Pisolithus tinctorius Marx 270	GCA_000827335.1
Erysiphe necator	GCA_000798715.1
Diaporthe longicolla	GCA_000800745.1
Metarhizium album ARSEF 1941	GCA_000804445.1
Leptographium procerum	GCA_000806385.1
Scedosporium aurantiacum	GCA_000812075.1

Aspergillus ustus	GCA_000812125.1
Onygena corvina	GCA_000812245.1
Stagonosporopsis tanaceti	GCA_000812845.1
Lanzia echinophila	GCA_000812885.1
Rutstroemia sydowiana	GCA_000812895.1
Metarhizium guizhouense ARSEF 977	GCA_000814955.1
Magnusiomyces capitatus CNRMA 12.647	GCA_000817185.1
Curvularia papendorfii	GCA_000817285.1
Valsa mali	GCA_000818155.1
Hebeloma cylindrosporum h7	GCA_000827355.1
Sporothrix brasiliensis 5110	GCA_000820605.1
[Torrubiella] hemipterigena	GCA_000825705.1
Paxillus rubicundulus Ve08.2h10	GCA_000827395.1
Serendipita vermifera MAFF 305830	GCA_000827415.1
Scleroderma citrinum Foug A	GCA_000827425.1
Tulasnella calospora MUT 4182	GCA_000827465.1
Paxillus involutus ATCC 200175	GCA_000827475.1
Amanita muscaria Koide BX008	GCA_000827485.1
Hypholoma sublateritium FD-334 SS-4	GCA_000827495.1
Phlebiopsis gigantea 11061_1 CR5-6	GCA_000832265.1
Cylindrobasidium torrendii FP15055 ss-10	GCA_000934385.1
Oidiodendron maius Zn	GCA_000827325.1
Fistulina hepatica ATCC 64428	GCA_000934395.1
Schizopora paradoxa	GCA_001020605.1
Baeospora myosura	GCA_001179705.1
Macrocystidia cucumis	GCA_001179725.1
Clavaria fumosa	GCA_001179745.1
Termitomyces sp. J132	GCA_001263195.1
Trametes hirsuta	GCA_001302255.2
Trametes sp. AH28-2	GCA_001304625.1
Heterobasidion annosum	GCA_001457955.1
Talaromyces cellulolyticus	GCA_000829775.1
Moniliophthora roreri	GCA_001466705.1
Huntiella omanensis	GCA_000833645.1
Exophiala sideris	GCA_000835395.1
Phialophora americana	GCA_000835435.1
Sanghuangporus baumii	GCA_001481415.2
Lentinula edodes	GCA_001562095.1
Taphrina flavorubra JCM 22207	GCA_000836175.1

Taphrina populina JCM 22190	GCA_000836195.1
Leucoagaricus sp. *SymC.cos*	GCA_001563735.1
Hypoxylon sp. *E7406B*	GCA_000931505.1
Fibularhizoctonia sp. CBS 109695	GCA_001630335.1
Sistotremastrum niveocremeum HHB9708	GCA_001630475.1
Dactylonectria macrodidyma	GCA_000935225.1
Parasitella parasitica	GCA_000938895.1
Penicillium capsulatum	GCA_000943775.1
Mucor ambiguus	GCA_000950595.1
Daedalea quercina L-15889	GCA_001632345.1
Penicillium solitum	GCA_000952775.2
Hirsutella minnesotensis 3608	GCA_000956045.1
Fusarium sp. JS1030	GCA_000966855.1
Fusarium sp. JS626	GCA_000966865.1
Xylaria sp. JS573	GCA_000966885.1
Thielaviopsis punctulata	GCA_000968615.1
Penicillium verrucosum	GCA_000970515.2
Ceratocystis platani	GCA_000978885.1
Zymoseptoria brevis	GCA_000983655.1
Talaromyces islandicus	GCA_000985935.1
Aspergillus rambellii	GCA_000986645.1
Sistotremastrum suecicum HHB10207 ss-3	GCA_001632355.1
Candida sp. LDI48194	GCA_001005365.1
Starmerella apicola	GCA_001005415.1
Phaeomoniella chlamydospora	GCA_001006345.1
Laetiporus sulphureus 93-53	GCA_001632365.1
Exidia glandulosa HHB12029	GCA_001632375.1
Nigrograna mackinnonii	GCA_001007845.1
Atkinsonella texensis	GCA_001008035.1
Epichloe bromicola	GCA_001008065.1
Epichloe sylvatica	GCA_001008265.1
Neolentinus lepideus HHB14362 ss-1	GCA_001632425.1
Peniophora sp. CONT	GCA_001632445.1
Blastomyces silverae	GCA_001014755.1
Rhizopogon vinicolor AM-OR11-026	GCA_001658105.1
Stachybotrys chartarum	GCA_001021365.1
Nakaseomyces delphensis CBS 2170	GCA_001039675.1
Epichloe uncinata	GCA_001043855.1
Aspergillus sp. Z5	GCA_001044295.1

[Candida] *Nakaseomyces nivariensis* CBS 9983	GCA_001046915.1
[Candida] *Nakaseomyces castellii* CBS 4332	GCA_001046935.1
Nakaseomyces bacillisporus CBS 7720	GCA_001046975.1
Penicillium brasilianum	GCA_001048715.1
Trichoderma parareesei	GCA_001050175.1
Chrysoporthe austroafricana	GCA_001051155.2
[Candida] *Nakaseomyces bracarensis* CBS 10154	GCA_001077315.1
Aspergillus udagawae	GCA_001078395.1
Armillaria fuscipes	GCA_001679825.1
Grifola frondosa	GCA_001683735.1
Obba rivulosa	GCA_001687445.1
Tolypocladium ophioglossoides CBS 100239	GCA_001189435.1
Stemphylium lycopersici	GCA_001191545.1
Amanita polypyramis BW_CC	GCA_001691755.2
Ciborinia camelliae	GCA_001247705.1
Fusarium nygamai	GCA_001262555.1
Amanita inopinata Kibby_2008	GCA_001691775.3
Amanita brunnescens Koide BX004	GCA_001691785.2
Polyporus brumalis	GCA_001792895.1
Rhizopogon vesiculosus	GCA_001882365.1
Trametes pubescens	GCA_001895945.1
Phanerodontia chrysosporium	GCA_001910725.1
Hermanssonia centrifuga	GCA_001913855.2
Verticillium longisporum	GCA_001268145.1
Talaromyces purpureogenus	GCA_001270325.1
Ophiocordyceps unilateralis	GCA_001272575.2
Madurella mycetomatis	GCA_001275765.2
Chrysoporthe cubensis	GCA_001282315.2
Fusarium langsethiae	GCA_001292635.1
Mycosphaerella arachidis	GCA_001297265.1
Fomitopsis palustris	GCA_001937815.1
Trametes polyzona	GCA_001939255.1
Talaromyces verruculosus	GCA_001305275.1
Neonectria ditissima	GCA_001305505.1
Aspergillus westerdijkiae	GCA_001307345.1
Hymenoscyphus scutula	GCA_001399465.1
Saccharomyces boulardii (nom. inval.)	GCA_001413975.1

Hymenoscyphus infarciens	GCA_001414345.1
Hymenoscyphus salicellus	GCA_001414355.1
Hymenoscyphus laetus	GCA_001414375.1
Hymenoscyphus repandus	GCA_001414415.1
Hymenoscyphus fructigenus	GCA_001414455.1
Hymenoscyphus herbarum	GCA_001414485.1
Byssoonygena ceratinophila	GCA_001430925.1
Amauroascus mutatus	GCA_001430935.1
Amauroascus niger	GCA_001430945.1
Chrysosporium queenslandicum	GCA_001430955.1
Rosellinia necatrix	GCA_001445595.3
Diaporthe aspalathi	GCA_001447215.1
Leptographium lundbergii	GCA_001455505.1
Diplodia scrobiculata	GCA_001455585.1
Samsoniella hepiali	GCA_001455915.2
Lyophyllum decastes	GCA_001950495.1
Clarireedia homoeocarpa	GCA_001465935.1
Lyophyllum shimeji	GCA_001950515.1
Termitomyces sp. JCM 13351	GCA_001972325.1
Huntiella savannae	GCA_001483325.1
Colletotrichum falcatum	GCA_001484525.1
Knoxdaviesia proteae	GCA_001510565.1
Knoxdaviesia capensis	GCA_001510575.1
Aspergillus calidoustus	GCA_001511075.1
Davidsoniella virescens	GCA_001513805.1
Ceratocystis eucalypticola	GCA_001513815.1
Chrysoporthe deuterocubensis	GCA_001513825.2
Fusarium temperatum	GCA_001513835.1
Thielaviopsis musarum	GCA_001513885.1
Graphilbum fragrans	GCA_001513895.1
Penicillium freii	GCA_001513925.1
Amanita bisporigera	GCA_001983365.1
Preussia sp. BSL10	GCA_001553865.1
Reticulascus tulasneorum	GCA_001557505.1
Penicillium griseofulvum	GCA_001561935.1
Amanita phalloides	GCA_001983385.1
Colletotrichum salicis	GCA_001563125.1
Colletotrichum simmondsii	GCA_001563135.1
Irpex lacteus	GCA_001986395.2

Microdochium bolleyi	GCA_001566295.1
Conidiobolus coronatus NRRL 28638	GCA_001566745.1
Fusarium sambucinum	GCA_001567575.1
Talaromyces pinophilus	GCA_001571465.2
Gonapodya prolifera JEL478	GCA_001574975.1
Pseudocercospora musae	GCA_001578225.1
Pseudocercospora eumusae	GCA_001578235.1
Peltaster fructicola	GCA_001592805.2
Colletotrichum acutatum	GCA_001593745.1
Trametes coccinea BRFM310	GCA_002092935.1
Auricularia auricula-judae	GCA_002092955.1
Thielaviopsis ethacetica	GCA_001599055.1
Ambrosiozyma kashinagicola	GCA_001599075.1
Hyphopichia homilentoma	GCA_001599095.1
[Candida] sorboxylosa	GCA_001599115.1
Wickerhamia fluorescens	GCA_001599155.1
Pestalotiopsis sp. JCM 9685	GCA_001599175.1
Cyberlindnera fabianii	GCA_001599195.1
Meyerozyma carpophila	GCA_001599235.1
[Candida] succiphila	GCA_001599255.1
Wickerhamiella domercqiae	GCA_001599275.1
Sporopachydermia quercuum	GCA_001599295.1
Starmerella bombicola	GCA_001599315.1
[Candida] boidinii	GCA_001599335.1
Nakazawaea peltata	GCA_001599355.1
Phoma herbarum	GCA_001599375.1
Scheffersomyces lignosus	GCA_001599395.1
Chalaropsis thielavioides	GCA_001599435.1
Fusarium commune	GCA_001599515.1
Memnoniella echinata	GCA_001599555.1
Mucor circinelloides	GCA_001599575.1
Beverwykella pulmonaria	GCA_001599595.1
Thielaviopsis euricoi	GCA_001599615.1
Actinomucor elegans	GCA_001599635.1
Grammothele lineata	GCA_002150815.3
Basipetospora chlamydospora	GCA_001599675.1
Limonomyces culmigenus	GCA_002233555.1
Dentipellis sp. KUC8613	GCA_002286715.1
Auricularia heimuer	GCA_002287115.1

Gliomastix tumulicola	GCA_001599755.1
Exophiala alcalophila	GCA_001599775.1
Exophiala calicioides	GCA_001599795.1
Furcasterigmium furcatum	GCA_001599815.1
Thermoascus crustaceus	GCA_001599835.1
Aspergillus chevalieri	GCA_001599875.1
Priceomyces haplophilus	GCA_001599895.1
Symbiotaphrina buchneri	GCA_001599915.1
Pyrrhoderma noxium	GCA_002287475.2
Armillaria solidipes	GCA_002307675.1
Amanita thiersii Skay4041	GCA_002554575.1
Ambrosiozyma monospora	GCA_001599995.1
Pleurotus salmoneostramineus	GCA_002583695.1
Agaricales sp. HM26-F1	GCA_002718315.1
Ganoderma sinense ZZ0214-1	GCA_002760635.1
Phellinus lamaoensis	GCA_002794735.1
Porodaedalea pini	GCA_002794775.1
Yarrowia keelungensis	GCA_001600195.1
Coniferiporia sulphurascens	GCA_002794785.1
Ganoderma boninense	GCA_002900995.2
Panaeolus cyanescens	GCA_002938355.1
Psilocybe cyanescens	GCA_002938375.1
Gymnopilus dilepis	GCA_002938385.1
Athelia rolfsii	GCA_002940785.1
Wickerhamiella versatilis	GCA_001600375.1
Trametes villosa	GCA_002964805.1
Endocalyx cinctus	GCA_001600455.1
Yarrowia sp. JCM 30694	GCA_001600515.1
Yarrowia sp. JCM 30696	GCA_001600535.1
Albophoma yamanashiensis	GCA_001600555.1
Didymobotryum rigidum	GCA_001600575.1
Heterobasidion parviporum	GCA_002994785.1
Rhizopogon parksii	GCA_002994865.1
Rhizopogon hawkerae	GCA_002994995.1
Rhizopogon rudus	GCA_002995055.1
Millerozyma acaciae	GCA_001600675.1
Ascoidea asiatica	GCA_001600695.1
Rhizopogon sp. AM-2018a	GCA_002995095.1
Rhizopogon vulgaris	GCA_002995295.1

Ogataea methanolica	GCA_001600755.1
Rhizopogon villosulus	GCA_002995315.1
Rhizopogon fuscorubens	GCA_002995455.1
Alloascoidea hylecoeti	GCA_001600815.1
Rhizopogon salebrosus	GCA_002995475.1
Ganoderma tsugae	GCA_003057275.1
Pleurotus tuoliensis	GCA_003243755.1
Yarrowia sp. JCM 30695	GCA_001602355.1
Phyllosticta capitalensis	GCA_001604925.1
Phyllosticta citricarpa	GCA_001604955.1
Pleurotus eryngii	GCA_003243765.1
Colletotrichum tofieldiae	GCA_001618715.1
Termitomyces sp. MG145	GCA_003313055.1
Drechmeria coniospora	GCA_001625195.1
Colletotrichum incanum	GCA_001625285.1
Cairneyella variabilis	GCA_001625345.1
Sarcodon sp. MG97	GCA_003313065.1
Termitomyces sp. MG16	GCA_003313075.1
Diaporthe ampelina	GCA_001630405.1
Sporothrix globosa	GCA_001630435.1
Suillus spraguei	GCA_003313085.1
Pseudogymnoascus sp. 04NY16	GCA_001630575.1
Pseudogymnoascus sp. BL549	GCA_001630585.1
Pseudogymnoascus sp. BL308	GCA_001630595.1
Pseudogymnoascus pannorum	GCA_001630605.1
Boletus sp. MG95	GCA_003313155.1
Collybia sp. MG36	GCA_003313185.1
Tricholoma saponaceum	GCA_003313625.1
Suillus placidus	GCA_003313645.1
Tricholoma bakamatsutake	GCA_003313665.1
Termitomyces heimii	GCA_003313675.1
Russula abietina	GCA_003313715.1
Fusarium metavorans	GCA_001633045.1
Ophiocordyceps polyrhachis-furcata BCC 54312	GCA_001633055.2
Moelleriella libera RCEF 2490	GCA_001636675.1
Ascosphaera apis ARSEF 7405	GCA_001636715.1
Beauveria brongniartii RCEF 3172	GCA_001636735.1
Metarhizium rileyi RCEF 4871	GCA_001636745.1

Akanthomyces lecanii RCEF 1005	GCA_001636795.1
Sporothrix insectorum RCEF 264	GCA_001636815.1
Endoconidiophora laricicola	GCA_001640655.1
Ceratocystis adiposa	GCA_001640685.1
Stagonospora sp. SRC1lsM3a	GCA_001644525.1
Pyrenochaeta sp. DS3sAY3a	GCA_001644535.1
Russula sp. MG48	GCA_003313725.1
[Candida] ethanolica M2	GCA_001649435.1
Gongronella sp. w5	GCA_001650995.1
Linnemannia elongata AG-77	GCA_001651415.1
Trichophyton violaceum	GCA_001651435.1
Fusarium agapanthi	GCA_001654545.1
Spathaspora hagerdaliae	GCA_001655755.1
Spathaspora gorwiae	GCA_001655765.1
Spathaspora girioi	GCA_001657455.1
Talaromyces piceae	GCA_001657655.1
Pleurotus platypus	GCA_003313735.1
Emergomyces africanus	GCA_001660665.1
Leptoxyphium fumago	GCA_001660795.1
Pachysolen tannophilus NRRL Y-2460	GCA_001661245.1
Nadsonia fulvescens var. *elongata* DSM 6958	GCA_001661315.1
Lipomyces starkeyi NRRL Y-11557	GCA_001661325.1
[Candida] arabinofermentans NRRL YB-2248	GCA_001661425.1
Tortispora caseinolytica NRRL Y-17796	GCA_001661475.1
Capniomyces stellatus	GCA_001661515.1
Pseudogymnoascus sp. 23342-1-I1	GCA_001662575.1
Pseudogymnoascus sp. WSF 3629	GCA_001662585.1
Pseudogymnoascus sp. 24MN13	GCA_001662595.1
Pseudogymnoascus sp. 05NY08	GCA_001662605.1
Pseudogymnoascus sp. 03VT05	GCA_001662645.1
Colletotrichum godetiae	GCA_001663355.1
Hanseniaspora valbyensis NRRL Y-1626	GCA_001664025.1
Fusarium poae	GCA_001675295.1
Ceratocystiopsis minuta	GCA_001676865.1
Termitomyces sp. MG148	GCA_003313785.1
Fusarium cuneirostrum	GCA_001680505.1
Fusarium tucumaniae	GCA_001680535.1
Fusarium azukiicola	GCA_001680625.1

Fusarium brasiliense	GCA_001680685.1
Choanephora cucurbitarum	GCA_001683725.1
Tricholoma flavovirens	GCA_003313805.1
Sarcodon aspratus	GCA_003313825.1
Suillus sp. MG131	GCA_003313855.1
Russula aff. *compacta*	GCA_003313875.1
Boletus sp. MG55	GCA_003313885.1
Lactifluus pinguis	GCA_003313945.1
Lepidopterella palustris CBS 459.81	GCA_001692735.1
Cenococcum geophilum 1.58	GCA_001692895.1
Glonium stellatum	GCA_001692915.1
Colletotrichum lindemuthianum	GCA_001693025.2
Aspergillus cristatus	GCA_001693355.1
Aspergillus hancockii	GCA_001696595.2
Diaporthe helianthi	GCA_001702395.2
Komagataella pastoris	GCA_001708105.1
Fusarium asiaticum	GCA_001717845.1
Fusarium meridionale	GCA_001717855.1
Megacollybia marginata	GCA_003313965.1
Trichoderma pleuroti	GCA_001721665.1
Lactarius indigo	GCA_003313985.1
Hygrophorus pudorinus	GCA_003314045.1
Lactifluus hygrophoroides	GCA_003314055.1
Lactarius sp. MG50	GCA_003314065.1
Hanseniaspora osmophila	GCA_001747045.1
Hanseniaspora uvarum	GCA_001747055.1
Paraphoma sp. B47-9	GCA_001748405.1
Hymenopellis chiangmaiae	GCA_003314085.1
Hanseniaspora opuntiae	GCA_001749795.1
Penicillium sclerotiorum	GCA_001750025.1
Chaetomium cochliodes	GCA_001752565.1
Hygrophorus russula	GCA_003314125.1
Cantharellus cinnabarinus	GCA_003314235.1
Stropharia rugosoannulata	GCA_003314255.1
Chroogomphus rutilus	GCA_003314275.1
Brettanomyces anomalus	GCA_001754015.1
Cantharellus lutescens	GCA_003314295.1
Colletotrichum incanum	GCA_001855235.1
Endoconidiophora polonica	GCA_001856765.1

Coniochaeta ligniaria NRRL 30616	GCA_001879275.1
Calonectria pseudoreteaudii	GCA_001879505.1
Epicoccum sorghinum	GCA_001879705.1
Cantharellus appalachiensis	GCA_003314335.1
Emergomyces pasteurianus Ep9510	GCA_001883825.1
Albatrellus ellisii	GCA_003314395.1
Aspergillus brasiliensis CBS 101740	GCA_001889945.1
Aspergillus versicolor CBS 583.65	GCA_001890125.1
Aspergillus luchuensis CBS 106.47	GCA_001890685.1
Aspergillus sydowii CBS 593.65	GCA_001890705.1
Aspergillus wentii DTO 134E9	GCA_001890725.1
Ramaria sp. MG151	GCA_003314545.1
Talaromyces amestolkiae	GCA_001896365.1
Penicillium subrubescens	GCA_001908125.1
Pleurotus citrinopileatus	GCA_003314595.1
Ilyonectria destructans	GCA_001913115.1
Pholiota microspora	GCA_003314615.1
Aureobasidium sp. FSWF8-4	GCA_001914275.1
Tricholoma sp. MG77	GCA_003314665.1
Neolecta irregularis DAH-3	GCA_001929475.1
Scedosporium sp. IMV 00882	GCA_001931805.1
Beauveria sp. IMV 00265	GCA_001931865.2
Byssochlamys sp. IMV 00236	GCA_001931875.2
Cladosporiaceae sp. IMV 00045	GCA_001931905.2
Aspergillus aff. *floccosus* IMV 01167	GCA_001931935.1
Trichoderma sp. IMV 00454	GCA_001931985.1
Magnaporthe sp. MG03	GCA_001936055.1
Magnaporthe sp. MG05	GCA_001936065.1
Saccharomycopsis fibuligera x Saccharomycopsis cf. *fibuligera*	GCA_001936135.1
Saccharomycopsis fibuligera	GCA_001936155.1
Saccharomycopsis fibuligera	GCA_001936275.1
Magnaporthe sp. MG07	GCA_001936455.1
Magnaporthe sp. MG08	GCA_001936555.1
Magnaporthe sp. MG12	GCA_001936955.1
Colletotrichum chlorophyti	GCA_001937105.1
Albatrellus sp. MG142	GCA_003314695.1
Sugiyamaella xylanicola (nom. inval.)	GCA_001939105.2
Talaromyces wortmannii	GCA_001939245.1

Lactifluus volemus	GCA_003315835.1
Alternaria consortialis	GCA_001950455.1
Trichoderma koningii	GCA_001950475.1
Lactarius trivialis	GCA_003315845.1
Lactifluus piperatus	GCA_003315875.1
Penicillium citrinum	GCA_001950535.1
Candida sp. JCM 15000	GCA_001950555.1
Colletotrichum sublineola	GCA_001951195.1
Smittium mucronatum	GCA_001953115.1
Ramalina peruviana	GCA_001956345.1
Zancudomyces culisetae	GCA_001969505.1
Smittium culicis	GCA_001970855.1
Sarocladium oryzae	GCA_001972265.1
Lactifluus rugatus	GCA_003315895.1
Macrolepiota dolichaula	GCA_003315915.1
Diplodia seriata	GCA_001975905.1
Cyberlindnera fabianii	GCA_001983305.1
Lactarius sp. MG121	GCA_003315925.1
Lactarius hatsudake	GCA_003315955.1
Zygosaccharomyces parabailii	GCA_001984395.2
Lactifluus echinatus	GCA_003315975.1
Butyriboletus roseoflavus	GCA_003315995.1
Aspergillus carbonarius ITEM 5010	GCA_001990825.1
Penicillium sp. HKF2	GCA_002000375.1
Batrachochytrium salamandrivorans	GCA_002006685.1
Bretziella fagacearum	GCA_002018255.1
Ceratocystis harringtonii	GCA_002018265.1
Huntiella bhutanensis	GCA_002018275.1
Trichoderma guizhouense	GCA_002022785.1
[Candida] aaseri	GCA_002068075.1
Penicillium decumbens	GCA_002072245.1
Penicillium vulpinum	GCA_002072255.1
Penicillium antarcticum	GCA_002072345.1
Penicillium flavigenum	GCA_002072365.1
Penicillium steckii	GCA_002072375.1
Penicillium coprophilum	GCA_002072405.1
Penicillium nalgiovense	GCA_002072425.1
Metschnikowia australis	GCA_002073855.1
Rachicladosporium sp. CCFEE 5018	GCA_002077045.2

Rachicladosporium antarcticum	GCA_002077065.1
Kretzschmaria deusta	GCA_002081935.3
Coprinus comatus	GCA_003316025.1
Boletus subvelutipes	GCA_003316035.1
Fusarium praegraminearum	GCA_002093855.1
Spathaspora boniae	GCA_002094185.1
Phialophora verrucosa	GCA_002099365.1
Catenaria anguillulae PL171	GCA_002102555.1
Anaeromyces robustus	GCA_002104895.1
Basidiobolus meristosporus CBS 931.73	GCA_002104905.1
Hesseltinella vesiculosa	GCA_002104935.1
Piromyces finnis	GCA_002104945.1
Neocallimastix californiae	GCA_002104975.1
Rhizoclosmatium globosum	GCA_002104985.1
Linderina pennispora	GCA_002104995.1
Clohesyomyces aquaticus	GCA_002105025.1
Boletus speciosus	GCA_003316055.1
Pseudomassariella vexata	GCA_002105095.1
Protomyces lactucae-debilis	GCA_002105105.1
Syncephalastrum racemosum	GCA_002105135.1
Absidia repens	GCA_002105175.1
Spathaspora xylofermentans	GCA_002105455.1
Yamadazyma laniorum	GCA_002109505.1
Emergomyces orientalis	GCA_002110485.1
Penicillium italicum	GCA_002116305.1
Epicoccum nigrum	GCA_002116315.1
Mycosphaerella sp. PB-2012b Mex 2-1-2	GCA_002116345.1
Dothistroma pini CBS 116487	GCA_002116355.1
Nothophaeocryptopus gaeumannii CBS 267.37	GCA_002116385.1
Ramularia endophylla CBS 113265	GCA_002116395.1
Scheffersomyces shehatae	GCA_002118035.1
Hypoxylon sp. CO27-5	GCA_002120305.1
Hypoxylon sp. CI-4A	GCA_002120315.1
Daldinia sp. EC12	GCA_002120325.1
Hypoxylon sp. EC38	GCA_002120335.1
Retiboletus ornatipes	GCA_003316065.1
Mycosphaerella populi pn0402	GCA_002153405.1
Aureobasidium melanogenum	GCA_002156615.1

Caloboletus calopus	GCA_003316085.1
Auricularia polytricha	GCA_003316125.1
Neoboletus magnificus	GCA_003316145.1
Neoboletus brunneissimus	GCA_003316195.1
Boletus bicolor	GCA_003316205.1
Piromyces sp. E2	GCA_002157105.1
Curvularia sp. IFB-Z10	GCA_002161795.1
Myriosclerotinia curreyana	GCA_002162495.1
Myriosclerotinia scirpicola	GCA_002162505.1
Monilinia aucupariae	GCA_002162555.1
Sclerotinia glacialis	GCA_002162575.1
Myriosclerotinia duriaeana	GCA_002162615.1
Fusarium pininemorale	GCA_002165215.1
Ophidiomyces ophidiicola	GCA_002167195.1
Fusarium euwallaceae	GCA_002168265.2
Calonectria leucothoes	GCA_002179835.1
Fusarium udum	GCA_002194535.1
Millerozyma farinosa	GCA_002196765.1
Gaeumannomyces sp. JS-464	GCA_002197995.1
Marssonina coronariae	GCA_002204255.1
Kazachstania servazzii	GCA_002214935.1
Rhizophlyctis rosea	GCA_002214945.1
Fusarium solani	GCA_002215905.1
Aspergillus persii	GCA_002215965.1
Dryadomyces quercus-mongolicae	GCA_002215975.1
Scedosporium boydii	GCA_002221725.1
Xerocomus impolitus	GCA_003316335.1
Dipodascus fermentans	GCA_002233575.1
Fusarium hostae	GCA_002234235.1
Fusarium sp. Na10	GCA_002234255.1
Aspergillus turcosus	GCA_002234965.2
Dothistroma septosporum	GCA_002236755.2
Elaphomyces granulatus	GCA_002240705.1
Saccharomyces uvarum	GCA_002242645.1
Lecanicillium sp. LEC01	GCA_002242745.2
Byssochlamys sp. BYSS01	GCA_002242795.1
Clarireedia sp. CPB17	GCA_002242865.1
Clarireedia jacksonii	GCA_002242905.1
Clarireedia monteithiana	GCA_002242955.1

Clarireedia sp. SE16F4	GCA_002242985.1
Scheffersomyces stambukii	GCA_002245345.1
Trichoderma koningiopsis	GCA_002246955.1
Colletotrichum coccodes	GCA_002249775.1
Bifiguratus adelaidae	GCA_002261195.1
Starmerella bacillaris	GCA_002270425.1
Stenocarpella maydis	GCA_002270565.1
Lomentospora prolificans	GCA_002276285.1
Chlorociboria aeruginascens	GCA_002276475.2
Tricholoma terreum	GCA_003316345.1
Bipolaris cookei	GCA_002286855.1
Tylopilus plumbeoviolaceoides	GCA_003316375.1
Thelephora aurantiotincta	GCA_003316405.1
Xylaria sp. MSU_SB201401	GCA_002288965.1
Russula lepida	GCA_003316425.1
Knufia petricola	GCA_002319055.1
Russula virescens	GCA_003316435.1
Ramaria cf. *rubripermanens*	GCA_003316465.1
Chiua virens	GCA_003316485.1
Suillus alpinus	GCA_003316505.1
Ramichloridium luteum	GCA_002368545.1
Penicillium janthinellum	GCA_002369805.1
Metschnikowia kipukae	GCA_002370135.1
[Candida] ipomoeae	GCA_002370145.1
Metschnikowia colocasiae	GCA_002370175.1
Metschnikowia sp. 04-226.1	GCA_002370245.1
Metschnikowia bowlesiae	GCA_002370295.1
Metschnikowia hawaiiensis	GCA_002370325.1
Metschnikowia drakensbergensis	GCA_002370475.1
Metschnikowia sp. M2Y3	GCA_002370575.1
Metschnikowia aberdeeniae	GCA_002370615.1
Metschnikowia cerradonensis	GCA_002370635.1
Metschnikowia matae var. maris	GCA_002370695.1
Metschnikowia similis	GCA_002370765.1
Metschnikowia sp. 03-147.1	GCA_002370795.1
Metschnikowia hamakuensis	GCA_002370815.1
Metschnikowia continentalis	GCA_002370835.1
Metschnikowia arizonensis	GCA_002370875.1
Metschnikowia lochheadii	GCA_002370915.1

Metschnikowia borealis	GCA_002374385.1
Metschnikowia cubensis	GCA_002374405.1
Metschnikowia santaceciliae	GCA_002374485.1
Metschnikowia kamakouana	GCA_002374535.1
Metschnikowia mauinuiana	GCA_002374555.1
Metschnikowia sp. 04-218.3	GCA_002374635.1
Metschnikowia shivogae	GCA_002374645.1
Metschnikowia hibisci	GCA_002374725.1
Metschnikowia sp. 13-106.1	GCA_002374735.1
Saccharomyces sp. M14	GCA_002375215.1
Penicillium occitanis (nom. inval.)	GCA_002382855.1
Lecanosticta acicola	GCA_002441625.1
Termitomyces eurrhizus	GCA_003316525.1
Helotiales sp. F229	GCA_002554605.1
Saccharomycopsis fodiens	GCA_002564235.1
Saccharomycopsis fermentans	GCA_002564245.1
Emmonsia crescens	GCA_002572855.1
Blastomyces parvus	GCA_002572885.1
Helicocarpus griseus UAMH5409	GCA_002573585.1
Polytolypa hystricis UAMH7299	GCA_002573605.1
Pulveroboletus ravenelii	GCA_003316545.1
Cordyceps sp. RAO-2017	GCA_002591385.1
Ophiocordyceps camponoti-rufipedis	GCA_002591395.1
Ophiocordyceps australis	GCA_002591415.1
Epichloe elymi	GCA_002591845.1
Nectria sp. B-13	GCA_002682825.1
Coemansia reversa NRRL 1564	GCA_002705745.1
Russula foetens	GCA_003316565.1
Ceratocystis albifundus	GCA_002742255.2
Apophysomyces variabilis	GCA_002749535.1
Xylaria striata	GCA_002749545.1
Colletotrichum sansevieriae	GCA_002749775.1
Gomphus bonarii	GCA_003316585.1
Talaromyces adpressus	GCA_002775195.1
Ceratocystis cacaofunesta	GCA_002776505.1
Raffaelea sp. RL272	GCA_002777955.1
Ambrosiella xylebori	GCA_002778035.1
Dryadomyces sulphureus	GCA_002778055.1
Grosmannia penicillata	GCA_002778075.1

Fragosphaeria purpurea	GCA_002778095.1
Ceratocystiopsis brevicomis	GCA_002778105.1
Dryadomyces quercivorus	GCA_002778125.1
Raffaelea arxii	GCA_002778165.1
Raffaelea ambrosiae	GCA_002778195.1
Esteya vermicola	GCA_002778215.1
Raffaelea albimanens	GCA_002778245.1
Microcyclosporella mali	GCA_002785985.1
Mycosphaerelloides madeirae	GCA_002785995.1
Zasmidium citrigriseum	GCA_002786025.1
Zasmidium angulare	GCA_002786045.1
Microcyclospora pomicola	GCA_002786065.1
Amanita pseudoporphyria	GCA_003316615.1
Tricholoma sp. MG99	GCA_003521275.1
Cantharellus cibarius	GCA_003521295.1
Alternaria brassicicola	GCA_002796735.1
Lecanicillium psalliotae	GCA_002796755.1
Coniochaeta hoffmannii	GCA_002798055.1
Gamarada debralockiae	GCA_002803225.1
Hericium coralloides	GCA_003675405.1
Scedosporium dehoogii	GCA_002812735.1
Scytalidium lignicola	GCA_002812745.2
Colletotrichum musae	GCA_002814275.1
Lentinus tigrinus ALCF2SS1-7	GCA_003813185.1
Cercospora zeina	GCA_002844615.1
Aspergillus ochraceoroseus IBT 24754	GCA_002846915.2
Aspergillus taichungensis	GCA_002850765.1
Verticillium nubilum	GCA_002851675.1
Verticillium albo-atrum	GCA_002851705.1
Verticillium klebahnii	GCA_002851715.1
Verticillium zaregamsianum	GCA_002851755.1
Verticillium isaacii	GCA_002851775.1
Hyaloscypha hepaticicola	GCA_002865625.1
Hyaloscypha variabilis F	GCA_002865655.1
Neonothopanus nambi	GCA_003987895.1
Metschnikowia hawaiiana	GCA_002893645.1
Metschnikowia orientalis	GCA_002893665.1
Metschnikowia drosophilae	GCA_002893705.1
Metschnikowia torresii	GCA_002893725.1

Metschnikowia drosophilae	GCA_002893735.1
Metschnikowia sp. AWRI3582	GCA_002894445.1
Aspergillus sp. ATCC 12892	GCA_002894705.1
Saccharomycopsis sp. UWO(PS) 91-127.1	GCA_002895615.1
Sphaceloma murrayae	GCA_002895985.1
Lachancea quebecensis	GCA_002900925.1
Mycena citricolor	GCA_003987915.1
Cladosporium cladosporioides	GCA_002901145.1
Tolypocladium capitatum	GCA_002901185.1
Monilinia polystroma	GCA_002909645.1
Spathaspora sp. UFMG-CM-Y6060	GCA_002911495.1
Byssochlamys sp. AF001	GCA_002914405.1
Talaromyces borbonicus	GCA_002916415.1
Penicillium sp. CF05	GCA_002916455.1
Tolypocladium paradoxum	GCA_002916505.1
Eurotiomycetes sp.	GCA_002917005.1
Ophiostoma ips	GCA_002917055.1
Hawksworthiomyces lignivorus	GCA_002917075.1
Candolleomyces aberdarensis	GCA_004126415.1
Lepiota venenata	GCA_004296355.1
Erysiphe pulchra	GCA_002918395.1
Cladosporium sp. SL-16	GCA_002921095.1
Steccherinum ochraceum	GCA_004332605.1
Aspergillus pseudoterreus	GCA_002927005.1
Cladonia uncialis	GCA_002927785.1
Colletotrichum fioriniae	GCA_002930455.1
Cercospora berteroae	GCA_002933655.1
Gloeostereum incarnatum	GCA_004338095.1
Chondrostereum purpureum	GCA_004354395.1
Rickenella mellea	GCA_004355085.1
Heliocybe sulcata	GCA_004369045.1
Rutstroemia sp. NJR-2017a WRK4	GCA_002946385.1
Rutstroemia sp. NJR-2017a BVV2	GCA_002946415.1
Rutstroemia sp. NJR-2017a BBW	GCA_002946425.1
Alternaria solani	GCA_002952155.1
Polyporus arcularius HHB13444	GCA_004369055.1
Cordyceps cicadae	GCA_002968875.1
Pluteus cervinus	GCA_004369065.1
Monascus ruber	GCA_002976275.1

Fusarium beomiforme	GCA_002980475.2
Fusarium burgessii	GCA_002980515.1
Fusarium algeriense	GCA_002982035.1
Arthonia radiata	GCA_002989075.1
Ilyonectria mors-panacis	GCA_002991585.1
Coprinopsis marcescibilis	GCA_004369085.1
Cercospora nicotianae	GCA_002994015.1
Pterula gracilis	GCA_004369125.1
Dendrothele bispora CBS 962.96	GCA_004369135.1
Coprinellus micaceus	GCA_004369175.1
Crucibulum laeve	GCA_004379715.1
Rhodofomes roseus	GCA_004679265.1
Dentipellis fragilis	GCA_004679275.1
Hericium alpestre	GCA_004681135.1
Russula griseocarnosa	GCA_004801975.1
Phellinidium pouzarii	GCA_004802695.1
Bondarzewia mesenterica	GCA_004802705.1
Eurotiomycetes sp. MA 6039	GCA_003004485.1
Eurotiomycetes sp. MA 6038	GCA_003004525.2
Antrodiella citrinella	GCA_004802725.1
Pleurotus ostreatoroseus	GCA_005298045.1
Hericium erinaceus	GCA_006506795.2
Pezicula radicicola	GCA_003008705.1
Lactarius deliciosus	GCA_006680135.1
Auriculariopsis ampla	GCA_007026445.1
Trichoderma brevicompactum	GCA_003012085.1
Trichoderma arundinaceum	GCA_003012105.1
Trichothecium sympodiale	GCA_003012115.1
Paramyrothecium roridum	GCA_003012165.1
Trichothecium roseum	GCA_003012185.1
Trichothecium ovalisporum	GCA_003012195.1
Microcyclospora tardicrescens	GCA_003012245.1
Fusarium sporotrichioides	GCA_003012315.1
Juglanconis oblonga	GCA_003012965.1
Juglanconis juglandina	GCA_003012975.1
Juglanconis sp. DMW523	GCA_003013055.1
Corynespora cassiicola Philippines	GCA_003016335.1
Coniella lustricola	GCA_003019895.1
Hanseniaspora gamundiae	GCA_003020785.1

Trichoderma longibrachiatum ATCC 18648	GCA_003025155.1
Cordyceps pruinosa	GCA_003025255.1
Cordyceps farinosa	GCA_003025275.1
Cordyceps tenuipes	GCA_003025305.1
Calonectria naviculata	GCA_003031705.1
Huntiella decipiens	GCA_003032515.1
Starmerella magnoliae	GCA_003033435.1
Wickerhamiella galacta	GCA_003045245.1
Pseudohalonectria lignicola	GCA_003049395.1
Magnaporthiopsis incrustans	GCA_003049425.1
Nakataea oryzae	GCA_003049435.1
Magnaporthiopsis rhizophila	GCA_003049465.1
Ophioceras dolichostomum	GCA_003049485.1
Coriolopsis trogii	GCA_007896425.1
Escovopsis weberi	GCA_003055145.1
Escovopsis sp. Ae724	GCA_003055165.1
Escovopsis sp. TC	GCA_003055185.1
Auricularia cornea	GCA_008368385.1
Escovopsis sp. Ae720	GCA_003055925.1
Escovopsis sp. Ae733	GCA_003055945.1
Escovopsis sp. AC	GCA_003055955.1
Lecanicillium sp. MT-2017a	GCA_003056605.1
Ganoderma sp. BRIUMSc	GCA_008694245.1
Tuber borchii	GCA_003070745.1
Ramalina intermedia	GCA_003073195.1
Periconia macrospinosa	GCA_003073855.1
Cadophora sp. DSE1049	GCA_003073865.1
Smittium megazygosporum	GCA_003086715.1
Furculomyces boomerangus	GCA_003086725.1
Smittium simulii	GCA_003086735.1
Smittium angustum	GCA_003097675.1
Paecilomyces niveus	GCA_003116535.1
Aspergillus fumigatus	GCA_003116565.1
Trametes sanguinea	GCA_008973685.1
Ceratobasidium theobromae	GCA_009078325.1
Gymnopus androsaceus JB14	GCA_009733575.1
Floccularia luteovirens	GCA_009739215.1
Colletotrichum sp. JS-367	GCA_003122705.1
Nadsonia starkeyi-henricii	GCA_003123035.1

Thermoascaceae sp. COH1141	GCA_003123655.1
Aspergillus sp. MA 6041	GCA_003138005.1
Penicillium sp. MA 6040	GCA_003138025.1
Aspergillus sp. MA 6037	GCA_003138035.1
Penicillium sp. MA 6036	GCA_003138045.1
Sarcomyxa edulis	GCA_009761415.1
Sanghuangporus sanghuang	GCA_009806525.1
Tuber magnatum	GCA_003182015.1
Monascus purpureus	GCA_003184285.1
Pseudevernia furfuracea	GCA_003184345.1
Evernia prunastri	GCA_003184365.1
Aspergillus sclerotiicarbonarius CBS 121057	GCA_003184635.1
Aspergillus ellipticus CBS 707.79	GCA_003184645.1
Aspergillus indologenus CBS 114.80	GCA_003184685.1
Aspergillus violaceofuscus CBS 115571	GCA_003184705.1
Aspergillus fijiensis CBS 313.89	GCA_003184825.1
Blastomyces percursus	GCA_003206225.1
Blastomyces emzantsi	GCA_003206725.1
Nakazawaea wickerhamii	GCA_003243035.1
Saturnispora dispora	GCA_003243065.1
Hanseniaspora uvarum	GCA_003243715.1
Sparassis latifolia	GCA_009812315.1
Pholiota adiposa	GCA_009935795.1
[Candida] sake	GCA_003243815.1
Colletotrichum gloeosporioides	GCA_003243855.1
Lasallia hispanica	GCA_003254425.1
Monilinia fructigena	GCA_003260565.1
Beauveria pseudobassiana	GCA_003267905.1
Stemphylium lycopersici	GCA_003268315.1
Diutina catenulata	GCA_003285555.1
Lepista sordida	GCA_010725545.1
Phlebia tremellosa	GCA_011032875.1
Macrocybe gigantea	GCA_011319805.1
Flammulina velutipes	GCA_011800155.1
Suillus brevipes	GCA_011800875.1
Fusarium equiseti	GCA_003313175.1
Armillaria gallica	GCA_012064365.1
Pseudopyrenochaeta lycopersici	GCA_003313425.1
Tuber umbilicatum	GCA_003313605.1

Ganoderma lucidum	GCA_012655175.1
Amylostereum areolatum	GCA_012932865.1
Pleurotus pulmonarius	GCA_012980535.1
Agrocybe pediades	GCA_013053245.1
Psilocybe cf. *subviscida*	GCA_013368295.1
Tetrapyrgos nigripes	GCA_013368315.1
Tricholomella constricta	GCA_013368375.1
Morchella septimelata	GCA_003313775.1
Leucoagaricus leucothites	GCA_013368445.1
Gymnopus confluens	GCA_013368555.1
Agrocybe cylindracea	GCA_013376435.1
Panellus pusillus	GCA_013387415.1
Leucocalocybe mongolica	GCA_013420905.1
Armillaria borealis	GCA_013427175.2
Hypsizygus marmoreus	GCA_013433165.1
Pleurotus tuber-regium	GCA_014058305.1
Mycena chlorophos	GCA_014461115.1
Mycena kentingensis (nom. inval.)	GCA_014461125.1
Mycena venus	GCA_014462655.1
Mycena sanguinolenta	GCA_014462675.1
Athelia sp. TMB	GCA_014898675.1
Paxillus ammoniavirescens	GCA_014904835.1
Thelephora ganbajun	GCA_014904855.1
Tricholoma matsutake 945	GCA_014904895.1
Melanogaster broomeanus	GCA_014904935.1
Gyrodon lividus	GCA_014904945.1
Annulohypoxylon stygium	GCA_003314315.1
Marasmius fiardii PR-910	GCA_014904975.1
Hydnum rufescens UP504	GCA_014904985.1
Lyophyllum atratum	GCA_014905825.1
Mycena galopus ATCC 62051	GCA_014925785.1
Pholiota molesta	GCA_014925825.1
Morchella eximia	GCA_003314645.1
Crassisporium funariophilum	GCA_014925845.1
Lactarius quietus	GCA_015025155.1
Clavulina sp. PMI_390	GCA_015039315.1
Ramaria rubella	GCA_015039355.1
Amanita rubescens	GCA_015039365.1
Ceratobasidium sp. AG-I	GCA_015039395.1

Imleria badia	GCA_015039435.1
Cortinarius glaucopus	GCA_015039465.1
Russula emetica	GCA_015178925.1
Russula ochroleuca	GCA_015178965.1
Hysterangium stoloniferum	GCA_015178975.1
Boletus edulis BED1	GCA_015179015.1
Gautieria morchelliformis	GCA_015179125.1
Flammulina filiformis	GCA_015342475.1
Pholiota conissans	GCA_015484465.1
Flammula alnicola	GCA_015499995.1
Mucidula mucida	GCA_015501055.1
Infundibulicybe gibba	GCA_015501065.1
Gymnopilus junonius	GCA_015501075.1
Cyathus striatus	GCA_015501535.1
Rhodocollybia butyracea	GCA_015501585.1
Hymenopellis radicata	GCA_015501595.1
Tuber calosporum	GCA_003316355.1
Panaeolus papilionaceus	GCA_015501605.1
Lepista nuda	GCA_015584075.1
Macrolepiota fuliginosa MF-IS2	GCA_015584085.1
Crepidotus variabilis	GCA_015657495.1
Clitopilus hobsonii	GCA_015708445.1
Thelephora terrestris	GCA_015956445.1
Suillus ampliporus	GCA_016584345.1
Sanghuangporus lonicericola	GCA_016618145.1
Astraeus odoratus	GCA_016618355.1
Suillus lakei	GCA_016628065.1
Suillus paluster	GCA_016628075.1
Suillus occidentalis	GCA_016628085.1
Rhizopus azygosporus	GCA_003325435.1
Saccharomyces kudriavzevii	GCA_003327635.1
Pichia kluyveri	GCA_003327685.1
Pichia fermentans	GCA_003339355.1
Ophiocordyceps camponoti-saundersi (nom. inval.)	GCA_003339415.1
Ophiocordyceps camponoti-leonardi (nom. inval.)	GCA_003339455.1
Aspergillus phoenicis ATCC 13157	GCA_003344505.1
Suillus placidus	GCA_016628565.1

Fusarium fracticaudum	GCA_003353625.1
Coleophoma crateriformis	GCA_003369635.1
Coleophoma cylindrospora	GCA_003369665.1
Corinectria fuckeliana	GCA_003385255.1
Neonectria hederae	GCA_003385265.1
Neonectria punicea	GCA_003385315.1
Colletotrichum lentis	GCA_003386485.1
Metschnikowia reukaufii	GCA_003401635.1
Aquanectria penicillioides	GCA_003415625.1
Articulospora tetracladia	GCA_003415645.1
Xylaria polymorpha	GCA_003426235.1
Xylaria longipes	GCA_003426265.1
Trichoderma atrobrunneum	GCA_003439915.1
Morchella importuna	GCA_003444635.1
Ceratocystis smalleyi	GCA_003449175.1
Phaeosphaeria sp. A1 3.1a	GCA_003501895.1
Phaeosphaeria sp. H6.2b	GCA_003503105.1
Parastagonospora avenae f. sp. *tritici*	GCA_003503115.1
Tuber microsphaerosporum	GCA_003521225.1
Cetradonia linearis	GCA_003521265.1
Suillus subalutaceus	GCA_016647625.1
Suillus subaureus	GCA_016647635.1
Acidomyces sp. *'richmondensis'*	GCA_003545705.1
Diversispora epigaea	GCA_003547095.1
Rhizophagus sp. MUCL 43196	GCA_003549995.1
Glomus cerebriforme	GCA_003550305.1
Gigaspora rosea	GCA_003550325.1
Protomyces sp. C29	GCA_003568695.1
Alternaria sp. MG1	GCA_003574525.1
Aspergillus spinulosporus	GCA_003574815.1
Aspergillus sclerotialis	GCA_003589665.1
Erysiphe neolycopersici	GCA_003610855.1
Golovinomyces cichoracearum	GCA_003611235.1
Piptocephalis cylindrospora	GCA_003614145.1
Dimargaris cristalligena	GCA_003614675.1
Blyttiomyces helicus	GCA_003614705.1
Thamnocephalis sphaerospora	GCA_003614735.1
Syncephalis pseudoplumigaleata	GCA_003614755.1
Arthrocladium fulminans	GCA_003614865.1

Cladosporium phlei	GCA_003614995.1
Caulochytrium protostelioides	GCA_003615045.1
Coniochaeta pulveracea	GCA_003635345.1
Trichophyton mentagrophytes	GCA_003664465.1
Suillus variegatus	GCA_016647645.1
Pseudocercospora crystallina	GCA_003666085.1
Fusarium subtropicale	GCA_003670145.1
Kluyveromyces nonfermentans	GCA_003670155.1
Berkeleyomyces basicola	GCA_003671435.1
Ophiognomonia clavigignenti-juglandacearum	GCA_003671545.1
Zygotorulaspora florentina	GCA_003671575.2
Komagataella pseudopastoris	GCA_003671595.2
Monilinia fructigena	GCA_003671625.1
Suillus tomentosus	GCA_016647685.1
Meyerozyma sp. JA9	GCA_003676015.1
Spathaspora sp. JA1	GCA_003676035.1
Venturia inaequalis	GCA_003689225.1
Venturia asperata	GCA_003689335.1
Venturia aucupariae	GCA_003693225.1
Pseudonectria foliicola	GCA_003693505.1
Pseudonectria buxi	GCA_003693545.1
Coccinonectria pachysandricola	GCA_003693555.1
Chaetothyriales sp. CBS 134920	GCA_003693665.1
Aspergillus sp. HF37	GCA_003698115.1
Fusarium kuroshium	GCA_003698175.1
Ogataea henricii	GCA_003705115.1
Citeromyces matritensis	GCA_003705165.1
Torulaspora franciscae	GCA_003705175.2
Ambrosiozyma ambrosiae	GCA_003705185.1
Ambrosiozyma vanderkliftii	GCA_003705225.1
Komagataella populi	GCA_003705255.2
Starmera quercuum	GCA_003705275.1
Sporopachydermia lactativora	GCA_003705295.1
Spencermartinsiella europaea	GCA_003705305.2
Priceomyces medius	GCA_003705335.1
Saturnispora mendoncae	GCA_003705365.1
Saccharomycopsis capsularis	GCA_003705375.1
Saturnispora serradocipensis	GCA_003705415.1

Saturnispora zaruensis	GCA_003705435.1
Pichia occidentalis	GCA_003705455.2
Pichia norvegensis	GCA_003705465.1
Pichia nakasei	GCA_003705495.1
Pichia heedii	GCA_003705525.2
Martiniozyma abietophila	GCA_003705535.2
Meyerozyma athensensis	GCA_003705555.1
Wickerhamiella cacticola	GCA_003705615.1
Ogataea nitratoaversa	GCA_003705625.1
Diddensiella caesifluorescens	GCA_003705635.1
Kodamaea laetipori	GCA_003705675.1
Blastobotrys serpentis	GCA_003705695.1
Blastobotrys raffinosifermentans	GCA_003705705.1
Blastobotrys peoriensis	GCA_003705735.2
Blastobotrys muscicola	GCA_003705745.2
Blastobotrys mokoenaii	GCA_003705765.2
Blastobotrys americana	GCA_003705795.2
Kluyveromyces dobzhanskii	GCA_003705805.2
Kockiozyma suomiensis	GCA_003705835.2
Lipomyces mesembrius	GCA_003705845.2
Lipomyces doorenjongii	GCA_003705865.1
Lipomyces japonicus	GCA_003705895.1
Lipomyces sp. NRRL Y-11553	GCA_003705905.1
Lipomyces lipofer	GCA_003705915.1
Zygosaccharomyces kombuchaensis	GCA_003705955.1
Tetrapisispora iriomotensis	GCA_003705975.1
Tetrapisispora namnaoensis	GCA_003705985.1
Peterozyma xylosa	GCA_003706035.1
Peterozyma toletana	GCA_003706045.1
Ogataea trehaloabstinens	GCA_003706095.1
Ogataea ramenticola	GCA_003706105.2
Ogataea philodendri	GCA_003706115.2
Ogataea glucozyma	GCA_003706155.1
Ogataea kodamae	GCA_003706165.2
Ogataea minuta	GCA_003706195.1
Ogataea methylovora	GCA_003706205.1
Ogataea naganishii	GCA_003706235.1
Ogataea nonfermentans	GCA_003706255.1
Nakazawaea holstii	GCA_003706265.1

Kuraishia ogatae	GCA_003706285.2
Kuraishia molischiana	GCA_003706305.2
Ambrosiozyma oregonensis (nom. inval.)	GCA_003706315.2
Citeromyces hawaiiensis	GCA_003706345.1
Citeromyces siamensis	GCA_003706365.1
Hanseniaspora clermontiae	GCA_003706385.1
Danielozyma ontarioensis	GCA_003706395.1
Deakozyma indianensis	GCA_003706415.2
Cyberlindnera mrakii	GCA_003706445.2
Candida corydali	GCA_003706475.2
Cephaloascus albidus	GCA_003706495.1
Suhomyces pyralidae	GCA_003706505.2
Suhomyces canberraensis	GCA_003706525.1
Teunomyces kruisii	GCA_003706535.1
Teunomyces gatunensis	GCA_003706565.2
Teunomyces cretensis	GCA_003706575.1
[Candida] montana	GCA_003706615.2
Ambrosiozyma maleeae (nom. inval.)	GCA_003706635.1
Barnettozyma californica	GCA_003706655.2
Barnettozyma hawaiiensis	GCA_003706665.1
[Candida] incommunis	GCA_003706695.2
Barnettozyma populi	GCA_003706705.2
Yamadazyma nakazawae	GCA_003706715.1
[Candida] ponderosae	GCA_003706755.2
Wickerhamomyces hampshirensis	GCA_003706765.2
Tortispora starmeri	GCA_003706775.2
Trigonopsis vinaria	GCA_003706805.2
Wickerhamomyces sp. NRRL YB-2243	GCA_003706815.2
Priceomyces castillae	GCA_003706855.2
Pichia terricola	GCA_003706875.2
Kurtzmaniella fragi	GCA_003706895.1
Cyberlindnera petersonii	GCA_003706915.2
Hyphopichia heimii	GCA_003706925.2
[Candida] blattae	GCA_003706955.2
Yamadazyma philogaea	GCA_003706995.2
Wickerhamomyces canadensis	GCA_003707015.2
Yamadazyma scolyti	GCA_003707025.1
Trigonopsis variabilis	GCA_003707065.2
Tortispora ganteri	GCA_003707115.1

Kurtzmaniella cleridarum	GCA_003707135.1
Phaffomyces opuntiae	GCA_003707165.1
Phaffomyces antillensis	GCA_003707195.1
Kregervanrija delftensis	GCA_003707205.2
Phaffomyces thermotolerans	GCA_003707215.1
Saturnispora hagleri	GCA_003707255.1
Starmera stellimalicola	GCA_003707265.1
Kregervanrija fluxuum	GCA_003707275.2
Saturnispora saitoi	GCA_003707305.1
Saturnispora silvae	GCA_003707315.2
Pichia exigua	GCA_003707355.1
Ogataea populi-albae	GCA_003707375.2
[Candida] schatavii	GCA_003707405.1
[Candida] restingae	GCA_003707425.2
Aciculoconidium aculeatum	GCA_003707435.1
Botryozyma nematodophila	GCA_003707445.1
Dipodascus albidus	GCA_003707475.1
Blastobotrys proliferans	GCA_003707485.2
Blastobotrys niveus	GCA_003707525.2
Lipomyces oligophaga	GCA_003707545.2
Kluyveromyces aestuarii	GCA_003707555.1
Lipomyces arxii	GCA_003707585.2
Zygosaccharomyces bisporus	GCA_003707595.1
Tetrapisispora fleetii	GCA_003707605.1
Ogataea trehalophila	GCA_003707645.1
Ogataea pini	GCA_003707665.1
Ambrosiozyma philentoma	GCA_003707675.2
Ogataea pilisensis	GCA_003707685.1
[Candida] freyschussii	GCA_003707735.1
Cyberlindnera misumaiensis	GCA_003707745.1
[Candida] oregonensis	GCA_003707785.2
Clavispora fructus	GCA_003707795.1
Cephaloascus fragrans	GCA_003707825.2
Suhomyces emberorum	GCA_003707835.2
Barnettozyma pratensis	GCA_003707865.1
Barnettozyma salicaria	GCA_003707885.1
Ambrosiozyma pseudovanderkliftii	GCA_003707895.2
Zygoascus ofunaensis	GCA_003707925.2
Zygoascus meyerae	GCA_003707935.2

Wickerhamomyces bovis	GCA_003707945.2
Wickerhamomyces alni	GCA_003707985.2
Yueomyces sinensis	GCA_003707995.1
Torulaspora maleeae	GCA_003708055.2
Starmera amethionina	GCA_003708085.1
Saturnispora dispora	GCA_003708095.2
Middelhovenomyces tepae	GCA_003708105.2
[Candida] orba	GCA_003708145.2
Hyphopichia rhagii	GCA_003708185.2
Hyphopichia gotoi	GCA_003708205.1
Cyberlindnera suaveolens	GCA_003708225.2
Cyberlindnera xylosilytica	GCA_003708285.1
Cyberlindnera mycetangii	GCA_003708295.2
Groenewaldozyma salmanticensis	GCA_003708315.2
Hanseniaspora pseudoguilliermondii	GCA_003708335.1
Cyberlindnera maclurae	GCA_003708355.2
Hanseniaspora singularis (nom. inval.)	GCA_003708365.1
[Candida] heveicola	GCA_003708405.1
Kazachstania rosinii	GCA_003708425.2
Grigorovia transvaalensis	GCA_003708445.2
Kazachstania viticola	GCA_003708455.1
Kazachstania kunashirensis	GCA_003708465.1
Kazachstania aerobia	GCA_003708495.1
Kazachstania unispora	GCA_003708525.2
Kazachstania bromeliacearum	GCA_003708535.2
Kazachstania turicensis	GCA_003708545.1
Debaryomyces nepalensis	GCA_003708585.1
Debaryomyces maramus	GCA_003708605.2
Magnusiomyces tetraspermus NRRL Y-7288	GCA_003708635.2
Dipodascus geniculatus	GCA_003708655.1
[Candida] tammaniensis	GCA_003708705.1
[Candida] wancherniae	GCA_003708715.2
[Candida] ascalaphidarum	GCA_003708745.2
[Candida] golubevii	GCA_003708755.1
Hanseniaspora hatyaiensis (nom. inval.)	GCA_003708765.1
Cyberlindnera americana	GCA_003708795.2
Kazachstania spencerorum	GCA_003708825.2
Kazachstania solicola	GCA_003708835.2
Kazachstania intestinalis	GCA_003708845.2

Kazachstania taianensis	GCA_003708865.1
Kazachstania siamensis	GCA_003708905.2
Kazachstania martiniae	GCA_003708925.2
Debaryomyces prosopidis	GCA_003708935.1
Debaryomyces subglobosus	GCA_003708965.1
[Candida] gorgasii	GCA_003708985.2
Magnaporthales sp. P1609	GCA_003709005.1
Ogataea zsoltii	GCA_003709205.2
Priceomyces carsonii	GCA_003709225.2
Cyberlindnera saturnus	GCA_003709245.2
Grigorovia yakushimaensis	GCA_003709265.1
Chaetothyriales sp. CBS 135597	GCA_003709825.1
Chaetothyriales sp. CBS 134916	GCA_003709845.1
Chaetothyriales sp. CBS 132003	GCA_003709865.1
Protomyces inouyei	GCA_003717155.1
Protomyces inundatus	GCA_003717165.1
Protomyces macrosporus	GCA_003717175.1
Protomyces gravidus	GCA_003717255.1
Protomyces pachydermus	GCA_003717275.1
Aspergillus incahuasiensis	GCA_003719405.1
Aspergillus olivimuriae	GCA_003719415.1
Ascobolus immersus RN42	GCA_003788565.2
Choiromyces venosus 120613-1	GCA_003788595.2
Terfezia boudieri ATCC MYA-4762	GCA_003788615.2
Valsa sordida	GCA_003795275.1
Cytospora leucostoma	GCA_003795295.1
Valsa malicola	GCA_003795315.1
Penicillium sp. SPG-F15	GCA_003800485.1
Penicillium sp. SPG-F1	GCA_003800495.1
Suillus plorans	GCA_016647745.1
Epichloe festucae Fl1	GCA_003814445.1
Oehlia diaphana	GCA_003833135.1
Aspergillus awamori	GCA_003850985.1
Penicillium sp. MT2 MMC-2018	GCA_003852855.1
Hyphopichia pseudoburtonii	GCA_003856775.1
Pseudophaeomoniella oleicola	GCA_003868215.1
Neonectria sp. DH2	GCA_003934905.1
Suillus subluteus	GCA_016647755.1
Tolypocladium inflatum	GCA_003945565.1

Fusarium duplospermum	GCA_003946985.1
Fusarium oligoseptatum	GCA_003946995.1
Fusarium floridanum	GCA_003947005.1
Fusarium sp. AF-6	GCA_003947015.1
Fusarium ambrosium	GCA_003947045.1
Jimgerdemannia lactiflua	GCA_003951145.1
Exophiala lecanii-corni	GCA_003955835.1
Oidium heveae	GCA_003957845.1
Suillus fuscotomentosus	GCA_016647785.1
Suillus americanus	GCA_016695465.1
Suillus decipiens	GCA_016695475.1
Proliferodiscus dingleyae	GCA_003988795.1
Ploettnerulaceae sp. D365	GCA_003988805.1
Chlorencoelia torta	GCA_003988815.1
Arachnopeziza araneosa	GCA_003988855.1
Phialocephala sp. D728	GCA_003988865.1
Lachnum nothofagi	GCA_003988875.1
Hyphodiscus sp. D1413	GCA_003988895.1
Hymenotorrendiella dingleyae	GCA_003988905.1
Pirottaea palmicola	GCA_003988945.1
Neobulgaria alba	GCA_003988965.1
Jimgerdemannia flammicorona	GCA_003990745.1
Endogone sp. FLAS-F59071	GCA_003990785.1
Aureobasidium sp. P6	GCA_003992365.1
Arthrobotrys flagrans	GCA_004000055.1
Taphrina sp. SM11	GCA_004000165.1
Davidsoniella eucalypti	GCA_004009845.1
Ascochyta rabiei	GCA_004011695.1
Ascochyta lentis	GCA_004011705.1
Saccharomycopsis malanga	GCA_004014935.1
Claviceps quebecensis	GCA_004016085.1
Claviceps humidiphila	GCA_004016155.1
Claviceps ripicola	GCA_004016175.1
Suillus hirtellus	GCA_016695485.1
Botryosphaeria kuwatsukai	GCA_004016305.1
Claviceps arundinis	GCA_004016465.1
Claviceps perihumidiphila	GCA_004016475.1
Verruconis sp.	GCA_004026245.1
Suillus discolor	GCA_016758755.1

Exophiala sp.	GCA_004026505.1
Grosmannia galeiformis	GCA_004028395.1
Zoopage sp. CT-All	GCA_004114245.1
Acaulopage tetraceros	GCA_004114255.1
Cochlonema odontosperma	GCA_004114315.1
Zoophagus insidians	GCA_004114325.1
Amphirosellinia nigrospora	GCA_004123355.1
Starmerella kuoi	GCA_004124915.1
Starmerella riodocensis	GCA_004124955.1
Starmerella ratchasimensis	GCA_004124975.1
Starmerella davenportii	GCA_004124985.1
Starmerella sorbosivorans	GCA_004125005.1
Starmerella tilneyi (nom. inval.)	GCA_004125055.1
Starmerella gropengiesseri	GCA_004125075.1
Wickerhamiella occidentalis	GCA_004125095.1
Wickerhamiella hasegawae	GCA_004125105.1
Wickerhamiella infanticola	GCA_004125145.1
Starmerella geochares	GCA_004125165.1
Starmerella vaccinii	GCA_004125185.1
Wickerhamiella pararugosa	GCA_004125235.1
Suillus cothurnatus	GCA_016758765.1
Suillus clintonianus	GCA_016758775.1
Hirsutella rhossiliensis	GCA_004142005.1
Ascochyta koolunga	GCA_004151165.1
Didymella pinodes	GCA_004151525.1
Monosporascus ibericus	GCA_004154915.1
Monosporascus cannonballus	GCA_004154925.1
Monosporascus sp. MC13-8B	GCA_004154975.1
Monosporascus sp. 5C6A	GCA_004155035.1
Monosporascus sp. CRB-8-3	GCA_004155055.1
Monosporascus sp. CRB-9-2	GCA_004155915.1
Monosporascus sp. MG133	GCA_004155925.1
Monosporascus sp. GIB2	GCA_004155935.1
Monosporascus sp. mg162	GCA_004155945.1
Alternaria gaisen	GCA_004156025.2
Alternaria tenuissima	GCA_004156035.1
Aeminium ludgeri	GCA_004216415.1
Metschnikowia aff. *pulcherrima*	GCA_004217705.1
Suillus bovinus	GCA_016758785.1

Talaromyces funiculosus	GCA_004299765.1
Cercospora sojina	GCA_004299825.1
Cladobotryum protrusum	GCA_004303015.1
Suillus weaverae	GCA_016767475.1
Suillus spraguei	GCA_016800925.1
Ascochyta viciae	GCA_004335155.1
Phoma sp. RAV-16-625	GCA_004335185.1
Ascochyta viciae-villosae	GCA_004335205.1
Didymella lethalis	GCA_004335245.1
Ascochyta fabae	GCA_004335285.1
Pyricularia sp. CBS 133598	GCA_004337975.1
Hypholoma fasciculare	GCA_016801325.1
Rhizoctonia solani	GCA_016906535.1
Xylaria grammica	GCA_004353285.1
Ceratobasidium sp. AG-Ba	GCA_016906575.1
Pleurotus citrinopileatus	GCA_017312325.1
Colletotrichum spinosum	GCA_004366825.1
Fusarium humuli	GCA_004366955.1
Fusarium incarnatum	GCA_004367075.1
Fusarium irregulare	GCA_004367085.1
Fusarium nanum	GCA_004367095.1
Fusarium clavum	GCA_004367155.1
Fusarium flagelliforme	GCA_004367175.1
Colletotrichum trifolii	GCA_004367215.1
Fusarium camptoceras	GCA_004367475.1
Fusarium citri	GCA_004367485.1
Fusarium scirpi	GCA_004367495.1
Colletotrichum sidae	GCA_004367935.1
Aspergillus viridinutans	GCA_004368095.1
Amauroderma rugosum	GCA_017499575.1
Psilocybe cubensis	GCA_017499595.1
Termitomyces bulborhizus	GCA_017580835.1
Termitomyces sp. Mn162	GCA_017607575.1
Termitomyces sp. T70a	GCA_017657195.1
Termitomyces sp. T69sscA	GCA_017657225.1
Termitomyces sp. T73sscA	GCA_017657235.1
Botryotinia calthae	GCA_004379285.1
Termitomyces sp. T108	GCA_017657275.1
Stemphylium vesicarium	GCA_004380135.1

Calonectria pseudonaviculata	GCA_004380915.1
Calonectria henricotiae	GCA_004380935.1
Didymella segeticola	GCA_004522025.1
Venturia nashicola	GCA_004522655.2
Venturia nashicola	GCA_004522665.1
Coniothyrium glycines	GCA_004523985.2
Alternaria atra	GCA_004634305.1
Termitomyces sp. DKA64	GCA_017657295.1
Termitomyces sp. K1Ag	GCA_017657315.1
Termitomyces titanicus	GCA_017657335.1
Aspergillus cejpii	GCA_004769165.1
Ascodesmis nigricans	GCA_004786065.1
Botrytis tulipae	GCA_004786125.1
Botrytis paeoniae	GCA_004786145.1
Botryotinia narcissicola	GCA_004786225.1
Botrytis hyacinthi	GCA_004786245.1
Botryotinia convoluta	GCA_004786275.1
Termitomyces sp. K1Ac	GCA_017657355.1
Cryphonectria macrospora	GCA_004802535.1
Amesia nigricolor	GCA_004802645.1
Termitomyces sp. T27	GCA_017657375.1
Megacollybia platyphylla	GCA_900068915.1
Entoloma clypeatum	GCA_900068945.1
Phoma sp. XZ068	GCA_004835665.1
Aspergillus ochraceus	GCA_004849945.1
Botrytis galanthina	GCA_004916875.1
Clitocybe nebularis	GCA_900068955.1
Kodamaea ohmeri	GCA_004919595.1
Hanseniaspora lachancei	GCA_004919765.1
Hanseniaspora guilliermondii	GCA_004919775.1
Hanseniaspora jakobsenii	GCA_004919785.1
Hanseniaspora sp. CRUB 1602	GCA_004919795.1
Hanseniaspora meyeri	GCA_004919825.1
Hanseniaspora nectarophila	GCA_004919845.1
Hanseniaspora occidentalis var. *citrica*	GCA_004919885.1
Hanseniaspora thailandica (nom. inval.)	GCA_004919915.1
[Candida] inconspicua	GCA_004931855.1
Alternaria brassicae	GCA_004936725.1
Penicillium sp. W3 MMC-2018	GCA_004959885.1

Cryomyces minteri	GCA_005059845.1
Friedmanniomyces endolithicus	GCA_005059855.1
Friedmanniomyces simplex	GCA_005059865.1
Calocybe gambosa	GCA_900068965.1
Salinomyces thailandica	GCA_005059885.1
Stylopage hadra	GCA_005111325.1
Xylaria sp. BCC 1067	GCA_005188305.1
Curvularia lunata	GCA_005212705.1
Penicillium sp. CF01	GCA_005250745.1
Taphrina wiesneri	GCA_005281515.1
Taphrina communis	GCA_005281525.1
Taphrina confusa	GCA_005281535.1
Taphrina deformans	GCA_005281575.1
Taphrina pruni	GCA_005281585.1
Hygrocybe conica	GCA_900068975.1
Pterula multifida	GCA_900068985.1
Colletotrichum tanaceti	GCA_005350895.1
Cercospora cf. *sigesbeckiae*	GCA_005356805.1
Cercospora cf. *flagellaris*	GCA_005356885.1
Metschnikowia sp. JCM 33374	GCA_005406065.1
Pichia manshurica	GCA_005406165.1
Penicillium oxalicum	GCA_005546515.1
[Paecilomyces] penicillatus	GCA_005765155.1
Elsinoe ampelina	GCA_005959805.1
Tuber indicum	GCA_006112555.1
Cladonia rangiferina	GCA_006146055.1
Tubaria furfuracea	GCA_900069095.1
Tinctoporellus epimiltinus	GCA_900155495.1
Ceratocystis manginecans	GCA_006408425.1
Cryphonectria nitschkei	GCA_006503525.1
Arthrinium phaeospermum	GCA_006503535.1
Coprinopsis strossmayeri	GCA_900156845.1
Apiospora malaysiana	GCA_006508115.1
Fusarium neocosmosporiellum	GCA_006518225.1
Synchytrium endobioticum	GCA_006535955.1
Spizellomyces sp. 'palustris'	GCA_006535965.1
Chytriomyces confervae	GCA_006535975.1
Powellomyces hirtus	GCA_006536005.1
Hansfordia pulvinata	GCA_006538405.1

Armillaria cepistipes	GCA_900157415.1
Colletotrichum shisoi	GCA_006783085.1
Massospora cicadina	GCA_006912075.1
Massospora platypediae	GCA_006912095.1
Golovinomyces magnicellulatus	GCA_006912115.1
Cordyceps javanica	GCA_006981975.1
Armillaria ostoyae	GCA_900157425.1
Peniophora sp. CBMAI 1063	GCA_900536885.1
Teratosphaeria zuluensis	GCA_007113905.1
Teratosphaeria gauchensis	GCA_007113925.1
Xylaria flabelliformis	GCA_007182795.1
Coniochaeta prunicola	GCA_007388105.1
Coniochaeta sp. IL0111	GCA_007388115.1
Coniochaeta sp. YLH0009	GCA_007388125.1
Coniochaeta sp. NC1642	GCA_007388135.1
Coniochaeta sp. AEA 9094	GCA_007388145.1
Coniochaeta sp. AEA 9055	GCA_007388195.1
Zygosaccharomyces mellis	GCA_007556495.1
Elsinoe australis	GCA_007556505.1
Elsinoe fawcettii	GCA_007556565.1
Margaritispora aquatica	GCA_007644065.1
Venturia effusa	GCA_007735645.1
Lachnellula arida	GCA_007821475.1
Lachnellula occidentalis	GCA_007821535.1
Lachnellula subtilissima	GCA_007821545.1
Lachnellula cervina	GCA_007825325.1
Lachnellula suecica	GCA_007825345.1
Lachnellula willkommii	GCA_007825375.1
Cyclocybe aegerita	GCA_902728275.1
Schizophyllum commune	GCF_000143185.1
Trichoderma viride	GCA_007896495.1
Metschnikowia caudata	GCA_008065175.1
Metschnikowia amazonensis	GCA_008065195.1
Metschnikowia agaves	GCA_008065255.1
Laccaria bicolor	GCF_000143565.1
Morchella conica	GCA_008079325.1
Pyrenophora teres f. teres	GCA_008086845.1
Ciboria shiraiana	GCA_008122225.1
Sarocladium brachiariae	GCA_008271525.1

Aspergillus sojae	GCA_008274985.1
Coprinopsis cinerea	GCF_000182895.1
Hypomyces perniciosus	GCA_008477525.1
Lasallia pustulata	GCA_008636195.1
Monilinia fructicola	GCA_008692225.1
Moniliophthora perniciosa	GCF_000183025.1
Sphaerosporella brunnea	GCA_008704415.1
Trichomonascus ciferrii	GCA_008704605.1
Fusarium xyrophilum	GCA_008711595.1
Lichtheimia ramosa	GCA_008728235.1
Macrophomina phaseolina	GCA_008729105.1
Taphrina betulina	GCA_008802775.1
Serpula lacrymans	GCF_000218685.1
Penicillium sp. BW_12	GCA_008931925.1
Penicillium sp. BW_MB	GCA_008931935.1
Penicillium sp. BW_162_3FA	GCA_008931945.1
Kluyveromyces starmeri	GCA_008973615.1
Stereum hirsutum FP-91666 SS1	GCF_000264905.1
Punctularia strigosozonata HHB-11173 SS5	GCF_000264995.1
Aspergillus leporis	GCA_009176345.1
Aspergillus novoparasiticus	GCA_009176405.1
Aspergillus minisclerotigenes	GCA_009176455.1
Morchella crassipes	GCA_009192285.1
Cercospora kikuchii	GCA_009193115.1
Phyllosticta citriasiana	GCA_009193405.1
Aspergillus avenaceus	GCA_009193465.1
Aspergillus tamarii	GCA_009193485.1
Aspergillus transmontanensis	GCA_009193505.1
Aspergillus sergii	GCA_009193525.1
Aspergillus arachidicola	GCA_009193545.1
Aspergillus coremiiformis	GCA_009193565.1
Aspergillus bertholletiae	GCA_009193595.1
Aspergillus pseudocaelatus	GCA_009193665.1
Coniochaeta sp. 2T2.1	GCA_009194965.1
Alternaria gansuensis	GCA_009289805.1
Monilinia laxa	GCA_009299455.1
Kazachstania telluris	GCA_009394695.1
Auricularia subglabra TFB-10046 SS5	GCF_000265015.1
Trametes versicolor FP-101664 SS1	GCF_000271585.1

Blumeriella jaapii	GCA_009599575.1
Helotiales sp. DMI_Dod_QoI	GCA_009613015.1
Fusarium cortaderiae	GCA_009617495.1
Fusarium austroamericanum	GCA_009617525.1
Fomitiporia mediterranea MF3/22	GCF_000271605.1
Saccharomyces cerevisiae x *Saccharomyces eubayanus*	GCA_009665555.1
Saccharomyces cerevisiae x *Saccharomyces kudriavzevii*	GCA_009665985.1
Saccharomyces cerevisiae x *Saccharomyces eubayanus x Saccharomyces uvarum*	GCA_009666275.1
Saccharomyces eubayanus x *Saccharomyces uvarum*	GCA_009666385.1
Saccharomyces cerevisiae x *Saccharomyces kudriavzevii x Saccharomyces eubayanus*	GCA_009666465.1
Saccharomyces cerevisiae x *Saccharomyces eubayanus*	GCA_009666655.1
Saccharomyces cerevisiae x *Saccharomyces eubayanus x Saccharomyces kudriavzevii* x *Saccharomyces uvarum*	GCA_009667055.1
Chalara longipes BDJ	GCA_009732865.1
Coniophora puteana RWD-64-598 SS2	GCF_000271625.1
Herpomyces periplanetae	GCA_009733715.1
Alectoria sarmentosa	GCA_009733775.1
Dichomitus squalens LYAD-421 SS1	GCF_000275845.1
Morchella sextelata	GCA_009741755.1
Metschnikowia citriensis (nom. inval.)	GCA_009746055.1
Metschnikowia dekortorum	GCA_009756545.1
Metschnikowia sp. UWOPS 12.619.2	GCA_009756645.1
Agaricus bisporus var. *bisporus* H97	GCF_000300575.1
Colletotrichum sp. COLG31	GCA_009800995.1
Lasiodiplodia sp. COLG20	GCA_009801085.1
Colletotrichum sp. COLG25	GCA_009801095.1
Lasiodiplodia sp. COLG96	GCA_009801105.1
Neurospora sp. CHS-2018a	GCA_009802275.1
Neurospora sp. FGSC 26638	GCA_009804825.1
Neurospora sp. FGSC 26637	GCA_009804845.1
Neurospora sp. FGSC 26633	GCA_009804945.1
Neurospora sp. FGSC 26636	GCA_009804955.1

Neurospora sp. FGSC 26632	GCA_009804965.1
Neurospora sp. FGSC 26635	GCA_009804975.1
Neurospora sp. FGSC 26634	GCA_009804985.1
Neurospora sp. FGSC 26630	GCA_009805085.1
Neurospora sp. FGSC 26631	GCA_009805105.1
Neurospora sp. LNF1-2	GCA_009805125.1
Neurospora sp. FGSC 26629	GCA_009805155.1
Neurospora discreta FGSC 8579	GCA_009805215.1
Neurospora sp. LNF1-1	GCA_009805225.1
Neurospora pannonica FGSC 7221	GCA_009805235.1
Neurospora terricola FGSC 1889	GCA_009805285.1
Neurospora sp. FGSC 6877	GCA_009805295.1
Neurospora sp. FGSC 26628	GCA_009805345.1
Neurospora sp. FGSC 26627	GCA_009805365.1
Neurospora sp. FGSC 26626	GCA_009805385.1
Neurospora sp. FGSC 26625	GCA_009805435.1
Talaromyces cellulolyticus	GCA_009805475.1
Neurospora sp. FGSC 26624	GCA_009805485.1
Pezicula neosporulosa	GCA_009805495.1
Laburnicola sp. JP-R-44	GCA_009805535.1
Neurospora sp. FGSC 26623	GCA_009805935.1
Neurospora sp. CHS-2018b	GCA_009806015.1
Neurospora cerealis	GCA_009806085.1
Neurospora tetraspora	GCA_009806155.1
Neurospora sp. CHS-2018c	GCA_009806235.1
Davidsoniella neocaledoniae	GCA_009806295.1
Phanerochaete carnosa HHB-10118-sp	GCF_000300595.1
Davidsoniella australis	GCA_009806335.1
Colletotrichum asianum	GCA_009806415.1
Fibroporia radiculosa	GCF_000313525.1
Gigaspora margarita	GCA_009809945.1
Heterobasidion irregulare	GCF_000320585.1
Aspergillus tritici	GCA_009812425.1
Aspergillus amoenus	GCA_009812435.1
Calcarisporium arbuscula	GCA_009828645.1
Neofusicoccum umdonicola	GCA_009829365.1
Neofusicoccum ribis	GCA_009829435.1
Lasiodiplodia gonubiensis	GCA_009829795.1
Lasiodiplodia pseudotheobromae	GCA_009829805.1

Neofusicoccum kwambonambiense	GCA_009829855.1
Neofusicoccum cordaticola	GCA_009830905.1
Colletotrichum destructivum	GCA_009900065.1
Ceratocystis fimbriata	GCA_009914735.1
Metschnikowia pulcherrima	GCA_009932455.1
Gloeophyllum trabeum ATCC 11539	GCF_000344685.1
Postia placenta MAD-698-R-SB12	GCF_002117355.1
Delphinella strobiligena	GCA_009982845.1
Lizonia empirigonia	GCA_009982855.1
Trichoderma sp. TW21990_1	GCA_010015515.1
Decorospora gaudefroyi	GCA_010015605.1
Cucurbitaria berberidis CBS 394.84	GCA_010015615.1
Clathrospora elynae	GCA_010015635.1
Bimuria novae-zelandiae CBS 107.79	GCA_010015655.1
Byssothecium circinans	GCA_010015675.1
Aulographum hederae CBS 113979	GCA_010015705.1
Amniculicola lignicola CBS 123094	GCA_010015725.1
Setomelanomma holmii	GCA_010015745.1
Saccharata proteae CBS 121410	GCA_010015785.1
Rhizodiscina lignyota	GCA_010015805.1
Lophiotrema nucula	GCA_010015825.1
Podosphaera xanthii	GCA_010015925.1
Karstenula rhodostoma CBS 690.94	GCA_010093485.1
Lineolata rhizophorae	GCA_010093515.1
Melanomma pulvis-pyrius CBS 109.77	GCA_010093585.1
Lophium mytilinum	GCA_010093605.1
Massarina eburnea CBS 473.64	GCA_010093635.1
Ophiobolus disseminans	GCA_010093685.1
Plenodomus tracheiphilus IPT5	GCA_010093695.1
Patellaria atrata CBS 101060	GCA_010093705.1
Pleomassaria siparia CBS 279.74	GCA_010093715.1
Piedraia hortae CBS 480.64	GCA_010093745.1
Polychaeton citri CBS 116435	GCA_010093785.1
Sporormia fimetaria CBS 119925	GCA_010093795.1
Polyplosphaeria fusca	GCA_010093805.1
Teratosphaeria nubilosa	GCA_010093825.1
Myriangium duriaei CBS 260.36	GCA_010093895.1
Zopfia rhizophila CBS 207.26	GCA_010093925.1
Delitschia confertaspora ATCC 74209	GCA_010093945.1

Cercospora zeae-maydis SCOH1-5	GCA_010093985.1
Viridothelium virens	GCA_010094025.1
Didymosphaeria enalia	GCA_010094045.1
Ampelomyces quisqualis	GCA_010094095.1
Mucor lusitanicus	GCA_010203745.1
Trichodelitschia bisporula	GCA_010356995.1
Teratosphaeria destructans	GCA_010367425.1
Lophiostoma macrostomum CBS 122681	GCA_010405375.1
Microthyrium microscopicum	GCA_010405405.1
Lentithecium fluviatile CBS 122367	GCA_010405425.1
Sparassis crispa	GCF_003851025.1
Tothia fuscella	GCA_010583015.1
Antarctomyces pellizariae	GCA_010623925.1
Mycena indigotica	GCF_014461135.1
Pleurotus ostreatus	GCF_014466165.1
Exophiala phaeomuriformis	GCA_010883475.1
Saccharomycopsis schoenii	GCA_010994365.1
Saccharomycopsis crataegensis	GCA_010994405.1
Saccharomyces pastorianus	GCA_011022315.1
Massariosphaeria phaeospora	GCA_011032825.1
Cystobasidiopsis lactophilus	GCA_001599975.1
Fusarium cf. *solani*	GCA_011033385.1
Fusarium cf. *falciforme*	GCA_011033785.1
Fusarium cf. *aywerte*	GCA_011034095.1
Fusarium cf. *verticillioides*	GCA_011035275.1
Fusarium cf. *hostae*	GCA_011035825.1
Fusarium cf. *nygamai*	GCA_011036685.1
Ascochyta rabiei	GCA_011037155.1
Xylaria multiplex	GCA_011057905.1
Didymella keratinophila	GCA_011058865.1
Neopestalotiopsis sp. 37M	GCA_011058875.1
Didymella heteroderae	GCA_011058895.1
Curvularia kusanoi	GCA_011058905.1
Trichoderma lentiforme	GCA_011066345.1
Brettanomyces nanus	GCA_011074865.2
Brettanomyces bruxellensis	GCA_011074885.2
[Humicola] grisea var. thermoidea	GCA_011316235.1
Rusavskia elegans	GCA_011316305.1
Coniella vitis	GCA_011317545.1

Monascus purpureus	GCA_011319195.1
Cystobasidium pallidum	GCA_001599955.1
Fusarium protoensiforme	GCA_011320165.1
Diplocarpon rosae	GCA_011320175.1
Penicillium rolfsii	GCA_011392555.1
Dactylonectria torresensis	GCA_011426275.1
Botryosphaeria dothidea	GCA_011503125.2
Mortierella sp. BCC40632	GCA_011634665.1
Cryphonectria parasitica EP155	GCA_011745365.1
Cladosporium sp. TM138-S3	GCA_011745625.1
Penicillium sp. OUCMDZ-019	GCA_011750695.1
Diplocarpon rosae	GCA_011750715.1
Erythrobasidium yunnanense	GCA_001600175.1
Tortispora sangerardonensis	GCA_011751505.1
Hypomyces rosellus	GCA_011799845.1
Erythrobasidium hasegawianum	GCA_001972285.1
Fonsecaea pugnacius	GCA_011800825.1
Cystobasidiaceae sp. HBUAS51001	GCA_003351005.1
Phyllachora maydis	GCA_011801745.1
Colletotrichum karsti	GCA_011947395.2
Colletotrichum camelliae	GCA_011947485.1
Symmetrospora coprosmae	GCA_008802785.1
Dactylella cylindrospora	GCA_012184295.1
Drechslerella brochopaga	GCA_012184305.1
Arthrobotrys entomopaga	GCA_012184315.1
Dactylellina cionopaga	GCA_012184355.1
Xenoacremonium recifei	GCA_012184525.1
Trichophyton kuryangei	GCA_012184535.1
Aspergillus floridensis	GCA_012184565.1
Trichophyton yaoundei	GCA_012184575.1
Simplicillium aogashimaense	GCA_012273805.1
Sclerotiophoma versabilis	GCA_012274445.1
Brettanomyces custersianus	GCA_012295335.1
Brettanomyces naardenensis	GCA_012295355.1
Pyrenophora graminea	GCA_012365135.1
Fusarium cerealis	GCA_012600195.1
Dacryopinax primogenitus	GCA_000292625.1
Aspergillus fumigatiaffinis	GCA_012656285.1
Torulaspora pretoriensis	GCA_012851205.1

Aspergillus flavus	GCA_012897115.1
Calocera viscosa TUFC12733	GCA_001630345.1
Calocera cornea HHB12733	GCA_001632435.1
Tilletia horrida	GCA_001006505.1
Fusarium albosuccineum	GCA_012931995.1
Fusarium acutatum	GCA_012932015.1
Fusarium austroafricanum	GCA_012932025.1
Golubevia pallescens	GCA_001599655.1
Ophiocordyceps sinensis	GCA_012934285.1
Meira nashicola	GCA_001600355.1
Tilletia controversa	GCA_001645045.2
Tilletia indica	GCA_001689995.1
Fusarium tanahbumbuense	GCA_012977735.1
Fusarium anguioides	GCA_012977745.1
Fusarium nurragi	GCA_012977755.1
Fusarium babinda	GCA_012977765.1
Pseudocercospora macadamiae	GCA_012978405.1
Fusarium sp. NRRL 62957	GCA_012978535.1
Fusarium drepaniforme	GCA_012978555.1
Ophiocordyceps camponoti-floridani	GCA_012980515.1
Salmacisia buchloeana	GCA_001990185.1
Fusarium sp. NRRL 22101	GCA_013010345.1
Alternaria burnsii	GCA_013036055.1
Quambalaria eucalypti	GCA_004016185.1
Cudoniella acicularis	GCA_013054445.1
Tilletia caries	GCA_004334575.1
Penicillium sp. str. #12	GCA_013138035.1
Podosphaera leucotricha	GCA_013170925.1
Venturia oleaginea	GCA_013176395.1
Tilletia laevis	GCA_009428275.1
Candida sojae	GCA_013177575.1
Saccharomyces bayanus NBRC1948	GCA_013180125.1
Saccharomyces cerevisiae x *Saccharomyces uvarum*	GCA_013180185.1
Saccharomyces cerevisiae x *Saccharomyces kudriavzevii* x *Saccharomyces uvarum*	GCA_013180725.1
Fusarium xylarioides	GCA_013183765.1
Fusarium brasilicum	GCA_013184295.1
Fusarium kyushuense	GCA_013184315.1

Fusarium luffae	GCA_013184325.1
Fusarium newnesense	GCA_013184375.1
Fusarium concolor	GCA_013184415.1
Fusarium sp. NRRL 53497	GCA_013184445.1
Fusarium continuum	GCA_013184455.1
Fusarium aywerte	GCA_013186375.1
Fusarium papillatum	GCA_013186395.1
Fusarium sp. NRRL 66088	GCA_013186415.1
Fusarium sp. NRRL 62610	GCA_013186425.2
Fusarium begoniae	GCA_013186755.1
Fusarium dlaminii	GCA_013186775.1
Fusarium pseudonygamai	GCA_013186785.1
Fusarium guttiforme	GCA_013186795.1
Fusarium sterilihyphosum	GCA_013186845.1
Fusarium ramigenum	GCA_013186855.1
Fusarium udum	GCA_013186905.1
Fusarium succisae	GCA_013186925.1
Fusarium thapsinum	GCA_013186935.1
Colletotrichum viniferum	GCA_013201765.1
Colletotrichum tropicale	GCA_013201785.1
Passalora sequoiae	GCA_013248845.1
Fusarium graminum	GCA_013266165.1
Fusarium gaditjirri	GCA_013266175.1
Fusarium sarcochroum	GCA_013266185.1
Fusarium zealandicum	GCA_013266195.1
Fusarium decemcellulare	GCA_013266205.1
Fusarium sp. NRRL 66182	GCA_013266265.1
Metarhizium anisopliae	GCA_013305495.1
Aspergillus quadrilineatus	GCA_013305525.1
Galactomyces reessii	GCA_013305925.1
Aspergillus latus	GCA_013306195.1
Dirinaria sp. GBRC AP01	GCA_013315955.1
Fusarium falciforme	GCA_013363125.1
Fusarium brevicatenulatum	GCA_013363135.1
Fusarium secorum	GCA_013363185.1
Fusarium tuaranense	GCA_013363205.1
Fusarium acuminatum	GCA_013363215.1
Diaporthe capsici	GCA_013364905.1
Fusarium phaseoli	GCA_013364925.1

Fusarium anthophilum	GCA_013364935.1
Fusarium tupiense	GCA_013364945.1
Geotrichum candidum	GCA_013365045.1
Trichoderma erinaceum	GCA_013365115.1
Cercospora citrullina	GCA_013365195.1
Pseudocercospora cruenta	GCA_013365205.1
Cercospora sesami	GCA_013365235.1
Cercospora brassicicola	GCA_013365245.1
Tilletia walkeri	GCA_009428295.1
Golubevia sp. BC0902	GCA_012976205.1
Golubevia sp. BC0812	GCA_012976215.1
Golubevia sp. BC0850	GCA_012976225.1
Candida theae	GCA_013368535.1
Ceraceosorus bombacis	GCA_900000165.1
Elsinoe arachidis	GCA_013372555.1
Tilletiaria anomala UBC 951	GCF_000711695.1
Tilletiopsis washingtonensis	GCF_003144115.1
Fusarium pseudoanthophilum	GCA_013395995.1
Fusarium napiforme	GCA_013396005.1
Fusarium mexicanum	GCA_013396015.1
Fusarium phyllophilum	GCA_013396025.1
Fusarium pseudocircinatum	GCA_013396035.1
Fusarium sp. NRRL 52700	GCA_013396095.1
Fusarium globosum	GCA_013396165.1
Fusarium denticulatum	GCA_013396175.1
Fusarium mundagurra	GCA_013396205.1
Fusarium sp. NRRL 25303	GCA_013396255.1
Fusarium heterosporum	GCA_013396295.1
Teratosphaeria pseudoeucalypti	GCA_013403795.1
Calonectria pseudoturangicola	GCA_013403825.1
Calonectria honghensis	GCA_013403855.1
Calonectria fujianensis	GCA_013406965.1
Calonectria crousiana	GCA_013406985.1
Calonectria aciculata	GCA_013406995.1
Morchella sp. a71	GCA_013407065.1
Fusarium sp. QHM	GCA_013416755.1
Fusarium sp	GCA_013416785.1
Thelonectria rubi	GCA_013420875.1
Pseudomicrostroma glucosiphilum	GCF_003144135.1

Aspergillus burnettii	GCA_013421405.1
Usnea hakonensis	GCA_013423325.1
Ceraceosorus guamensis	GCF_003144195.1
Zygosaccharomyces siamensis	GCA_013423405.1
Meira miltonrushii	GCF_003144205.1
Jaminaea rosea	GCF_003144245.1
Claviceps paspali	GCA_013435705.1
Diaporthe sp. HANT25	GCA_013435955.1
Ramularia coccinea	GCA_013461505.1
Acaromyces ingoldii	GCF_003144295.1
Syncephalastrum contaminatum	GCA_013461545.1
Penicillium polonicum	GCA_013466175.1
Fusarium albidum	GCA_013618265.1
Fusarium armeniacum	GCA_013618295.1
Fusarium sp. FSAMSC_23	GCA_013618385.1
Fusarium domesticum	GCA_013618395.1
Fusarium hainanense	GCA_013618405.1
Fusarium longipes	GCA_013618495.1
Clavariopsis aquatica	GCA_013620735.1
Fusarium musae	GCA_013623345.1
Fusarium bactridioides	GCA_013623355.1
Fusarium illudens	GCA_013623515.1
Fusarium dimerum	GCA_013623525.1
Fusarium penzigii	GCA_013623535.1
Fusarium nisikadoi	GCA_013623555.1
Fusarium nematophilum	GCA_013623595.1
Fusarium setosum	GCA_013623625.1
Fusarium sublunatum	GCA_013623665.1
Fusarium transvaalense	GCA_013623685.1
Fusarium verrucosum	GCA_013623715.1
Fusarium robinianum	GCA_013623725.1
Fusarium zanthoxyli	GCA_013623745.1
Fusarium foetens	GCA_013623845.1
Fusarium torulosum	GCA_013623875.1
Fusarium caatingaense	GCA_013624355.1
Fusarium sp. KOD 1611	GCA_013624395.1
Torulaspora sp. CBS 2947	GCA_013694445.1
Fusarium sp. NRRL 25184	GCA_013755755.1
Cylindrocarpon cylindroides	GCA_013756995.1

Neonectria coccinea	GCA_013757005.1
Fusarium proliferatum	GCA_013758875.1
Fusarium bulbicola	GCA_013758895.1
Fusarium sacchari	GCA_013759005.1
Neonectria galligena	GCA_013759035.1
Fusarium sp. NRRL 29148	GCA_013759095.1
Fusarium sp. NRRL 47473	GCA_013759115.1
Fusarium sp. NRRL 53293	GCA_013759125.1
Fusarium coicis	GCA_013781345.1
Diaporthe sp. NJD1	GCA_013842865.1
Tuber brumale	GCA_014065205.1
Talaromyces stollii	GCA_014065225.1
Raffaelea lauricola	GCA_014183025.1
Harringtonia aguacate	GCA_014183095.1
Colletotrichum musicola	GCA_014235935.1
Colletotrichum plurivorum	GCA_014235945.1
Colletotrichum sojae	GCA_014235955.1
Aspergillus oerlinghausenensis	GCA_014250555.1
Laburnicola sp. R22_1	GCA_014281115.1
Aspergillus hiratsukae	GCA_014281905.1
Neospermospora avenae	GCA_014282315.1
Pseudocercospora fuligena	GCA_014298035.1
Pyrenophora teres f. *maculata*	GCA_014334815.1
Malassezia caprae	GCA_001264625.1
Malassezia cuniculi	GCA_001264635.1
Malassezia equina	GCA_001264685.1
Malassezia yamatoensis	GCA_001264885.1
Trichoderma lixii	GCA_014468695.1
Diaporthe citri	GCA_014595645.1
Penicillium crustosum	GCA_014621375.1
Stylonectria norvegica	GCA_014621405.1
Cylindrodendrum hubeiense	GCA_014621425.1
Neopestalotiopsis clavispora	GCA_014621435.1
Calonectria ilicicola	GCA_014622005.1
Plectosphaerella sp. P0831	GCA_014635975.1
Plectosphaerella cucumerina	GCA_014636675.1
Colletotrichum theobromicola	GCA_014705045.1
Colletotrichum nymphaeae	GCA_014705085.1
Colletotrichum sp. HC278	GCA_014705495.1

Colletotrichum sp. HC292	GCA_014705525.1
Colletotrichum australisinense (nom. inval.)	GCA_014706365.1
Malassezia obtusa	GCA_001264985.1
Fusarium sp. DS 682	GCA_014764975.1
Aspergillus flavus	GCA_014784225.1
Spiromastix sp. SCSIO F190	GCA_014805645.1
Malassezia dermatis	GCA_001600775.1
Fusarium acaciae-mearnsii	GCA_014822065.1
Fusarium buharicum	GCA_014822075.1
Fusarium stilboides	GCA_014822085.1
Fusarium sp. NRRL 66894	GCA_014824365.1
Fusarium cyanostomum	GCA_014824385.1
Fusarium sp. NRRL 66896	GCA_014824405.1
Fusarium concentricum	GCA_014824425.1
Fusarium torreyae	GCA_014824505.1
Malassezia japonica	GCA_001600795.1
Malassezia nana	GCA_001600835.1
Malassezia vespertilionis	GCA_002818225.1
Malassezia sp.	GCA_004026415.1
Malassezia furfur	GCA_009938135.1
Malassezia slooffiae	GCA_010577765.1
Malassezia globosa	GCF_000181695.1
Malassezia sympodialis	GCF_000349305.1
Penicillium ucsense	GCA_014839625.1
Penicillium ucsense	GCA_014839855.1
Apophysomyces ossiformis	GCA_014839865.1
Chrysosporium tropicum	GCA_014839905.1
Entomophthora muscae	GCA_014839935.1
Trichophyton simii	GCA_014839955.1
Penicillium camemberti	GCA_014839975.1
Hortaea werneckii	GCA_014843535.1
Cryphonectria radicalis	GCA_014849355.1
Cryphonectria carpinicola	GCA_014849695.1
Cryphonectria carpinicola	GCA_014849955.1
Cryphonectria naterciae	GCA_014850475.1
Cryphonectria japonica	GCA_014851275.1
Venturia carpophila	GCA_014858625.1
Diaporthe citriasiana	GCA_014872975.1
Diaporthe citrichinensis	GCA_014872995.1

Fusarium sp. 190-020-2	GCA_014884825.1
Fusarium sp. 'flavolapis'	GCA_014885045.1
Botrytis aclada	GCA_014898285.1
Botrytis byssoidea	GCA_014898295.1
Stromatinia cepivora	GCA_014898415.1
Botryotinia globosa	GCA_014898425.1
Botrytis sinoallii	GCA_014898435.1
Botrytis porri	GCA_014898465.1
Botrytis squamosa	GCA_014898485.1
Botrytis deweyae	GCA_014898535.1
Botrytis elliptica	GCA_014898555.1
Malassezia pachydermatis	GCF_001278385.1
Fusarium lateritium	GCA_014898835.1
Fusarium miscanthi	GCA_014898875.1
Fusarium lyarnte (nom. inval.)	GCA_014898885.1
Fusarium chlamydosporum	GCA_014898915.1
Fusarium nelsonii	GCA_014898925.1
Fusarium nodosum	GCA_014898975.1
Fusarium sibiricum	GCA_014898995.1
Fusarium palustre	GCA_014899045.1
Fusarium goolgardi	GCA_014899075.1
Fusarium redolens	GCA_014899085.1
Fusarium buxicola	GCA_014899095.1
Malassezia restricta	GCF_003290485.1
Acephala macrosclerotiorum	GCA_014904845.1
Microbotryum lychnidis-dioicae	GCA_001244265.1
Rhodotorula sp. JG-1b	GCA_001541205.1
Wilcoxina mikolae CBS 423.85	GCA_014904905.1
Leucosporidium creatinivorum	GCA_001600635.1
Rhodotorula sp. FNED7-22	GCA_001914285.1
Rhodotorula mucilaginosa	GCA_002806785.1
Glaciozyma antarctica PI12	GCA_002917775.1
Racodium therryanum	GCA_014905255.1
Metschnikowia persimmonesis	GCA_014905795.1
Rhodotorula kratochvilovae	GCA_002917965.1
Rhodotorula taiwanensis	GCA_002922495.1
Leucosporidium scottii	GCA_003054985.1
Rhodotorula mucilaginosa	GCA_003055205.1
Nannizziopsis barbatae	GCA_014964245.1

Microbotryum silenes-acaulis	GCA_003665825.1
Rhodotorula sp. CCFEE 5036	GCA_005059875.1
Rhodotorula paludigena	GCA_005281665.1
Rhodotorula diobovata	GCA_006352295.1
Rhodotorula sp. ZM1	GCA_009806315.1
Sporidiobolus pararoseus	GCA_010758995.1
Microbotryum silenes-dioicae	GCA_014805725.1
Botrytis fabae	GCA_015147945.1
Rhodotorula glutinis	GCA_015501985.1
Sporobolomyces roseus	GCA_016617785.1
Microbotryum violaceum	GCA_900015485.1
Trichophaea hybrida	GCA_015178995.1
Microbotryum intermedium	GCA_900096595.1
Terfezia claveryi	GCA_015179025.1
Tirmania nivea	GCA_015179035.1
Kalaharituber pfeilii	GCA_015179045.1
Microbotryum saponariae	GCA_900102585.1
Trichoderma oligosporum	GCA_015266385.1
Epicoccum latusicollum	GCA_015266435.1
Exophiala sp. BO6	GCA_015295625.1
Rhodotorula toruloides	GCF_000320785.1
Aotearoamyces nothofagi	GCA_015345905.1
Rhodotorula graminis WP1	GCF_001329695.1
Dissophora ornata	GCA_015499975.1
Haplosporangium gracile	GCA_015499985.1
Mixia osmundae IAM 14324	GCF_000708205.1
Haplosporangium bisporale	GCA_015500155.1
Melampsora pinitorqua Mpini7	GCA_000464645.1
Cronartium comandrae C4	GCA_000464975.1
Cronartium ribicola 11-2	GCA_000500245.1
Endocronartium harknessii PhW48OC	GCA_000500795.1
Uromyces viciae-fabae	GCA_000785685.1
Puccinia arachidis	GCA_001013415.1
Puccinia sorghi	GCA_001263375.1
Puccinia horiana	GCA_001624995.1
Mortierella sp. GBA35	GCA_015502445.1
Mortierella sp. AM989	GCA_015502455.1
Mortierella sp. 14UC	GCA_015502465.1
Mortierella sp. AD031	GCA_015502475.1

Mortierella sp. GBA39	GCA_015502485.1
Mortierella sp. AD010	GCA_015502575.1
Haplosporangium sp. Z 11	GCA_015529505.1
Haplosporangium sp. Z 767	GCA_015529515.1
Haplosporangium sp. Z 27	GCA_015529795.1
Penicillium roqueforti	GCA_015533775.1
Melampsora allii-populina	GCA_002157005.1
Melampsora aecidioides	GCA_002157015.1
Penicillium dipodomyicola	GCA_015585785.1
Melampsora abietis-canadensis	GCA_002157025.1
Penicillium griseoroseum	GCA_015586035.1
Aspergillus unguis	GCA_015586375.1
Melampsora medusae f. sp. *deltoidis*	GCA_002157035.1
Chrysoporthe sp. MAF-2017a	GCA_015678955.1
Mortierella sp. NVP85	GCA_015679515.1
Podila horticola	GCA_015679535.1
Mortierella sp. AD094	GCA_015679545.1
Podila epigama	GCA_015679605.1
Mortierella sp. AD011	GCA_015679695.1
Mortierella hygrophila	GCA_015680625.1
Rhizophagus clarus	GCA_015698045.1
Melampsora occidentalis	GCA_002157085.1
[Candida] mesenterica	GCA_015708695.1
Puccinia coronata f. sp. *avenae*	GCA_002873125.1
Kodamaea anthophila	GCA_015708815.1
Paraphaeosphaeria minitans	GCA_015832175.1
Clonostachys rosea	GCA_015832225.1
Colletotrichum liriopes	GCA_015832465.1
Aspergillus nanangensis	GCA_015844325.1
Modicella reniformis	GCA_015849295.1
Mortierella antarctica	GCA_015849325.1
Podila clonocystis	GCA_015852385.1
Gryganskiella cystojenkinii	GCA_015852425.1
Podila epicladia	GCA_015852495.1
Pseudopestalotiopsis theae	GCA_015881745.1
Uromyces transversalis	GCA_002994575.1
Hemileia vastatrix	GCA_004125335.1
Sporothrix phasma	GCA_016097075.1
Sporothrix curviconia	GCA_016097085.1

Sporothrix variecibatus	GCA_016097105.1
Sporothrix protearum	GCA_016097115.2
Aspergillus elegans	GCA_016097275.1
Aspergillus melleus	GCA_016097325.1
Apophysomyces sp. BC1015	GCA_016097715.1
Apophysomyces sp. BC1021	GCA_016097735.1
Apophysomyces sp. BC1034	GCA_016097755.1
Linnemannia gamsii	GCA_016097855.1
Dissophora globulifera	GCA_016097935.1
Podila humilis	GCA_016097945.1
Podila minutissima	GCA_016098005.1
Gamsiella multidivaricata	GCA_016098015.1
Mortierella sp. AD032	GCA_016098025.1
Mortierella sp. GBA30	GCA_016098035.1
Mortierella sp. GBA43	GCA_016098045.1
Mortierella sp. NVP41	GCA_016098085.1
Actinomortierella wolfii	GCA_016098105.1
Actinomortierella ambigua	GCA_016098155.1
Mortierella polycephala	GCA_016098165.1
Phialophora tarda	GCA_016109495.1
Phialophora chinensis	GCA_016109575.1
Phialophora expanda	GCA_016109585.1
Phialophora macrospora	GCA_016109925.1
Aspergillus sp. A31	GCA_016162245.1
Blastobotrys adeninivorans	GCA_016162255.1
Curvularia geniculata	GCA_016162275.1
Pseudocercospora ulei	GCA_016165855.1
Aspergillus felis	GCA_016413765.1
Beauveria felina	GCA_016490725.1
Trichoderma afroharzianum	GCA_016490745.1
Cytospora piceae	GCA_016508685.1
Hyphodiscus hymeniophilus	GCA_016508695.1
Kazachstania exigua	GCA_016584175.1
[Candida] californica	GCA_016584195.1
Puccinia novopanici	GCA_004348175.1
Celoporthe dispersa	GCA_016584495.1
Neocamarosporium betae	GCA_016612005.1
Puccinia hordei	GCA_007896445.1
Colletotrichum echinochloae	GCA_016618095.1

Puccinia striiformis f. sp. *tritici*	GCA_011750755.1
Puccinia triticina	GCA_013090125.1
Cronartium quercuum f. sp. *fusiforme* G11	GCA_015951145.1
Austropuccinia psidii	GCA_902702905.1
Puccinia graminis	GCF_000149925.1
Melampsora larici-populina	GCF_000204055.1
Capillidium heterosporum	GCA_016630905.1
Didymella arachidicola	GCA_016630955.1
Papiliotrema flavescens NRRL Y-50378	GCA_000442785.1
Kwoniella heveanensis BCC8398	GCA_000507405.3
Cryptococcus gattii VGIV IND107	GCA_000835755.1
Cryptococcus gattii VGII 2001/935-1	GCA_000835815.1
Cryptococcus gattii CA1873	GCA_000855695.1
Mrakia blollopis	GCA_000950635.1
Tremella fuciformis	GCA_000987905.1
Phaffia rhodozyma	GCA_001007165.2
Apiotrichum montevideense	GCA_001598995.1
Apiotrichum domesticum	GCA_001599015.1
Geosiphon pyriformis	GCA_016748015.1
Phaeotremella skinneri	GCA_001599695.1
Phaeotremella fagi	GCA_001599715.1
Naganishia albida	GCA_001599735.1
Holtermannia corniformis	GCA_001599935.1
Nigrospora oryzae	GCA_016758845.1
Umbelopsis vinacea	GCA_016758895.1
Umbelopsis isabellina	GCA_016758905.1
Thamnidium elegans	GCA_016758935.1
Mucor plumbeus	GCA_016758945.1
Circinella minor	GCA_016758965.1
Mucor saturninus	GCA_016758985.1
Stagonosporopsis rhizophila	GCA_016767195.1
Solicoccozyma phenolica	GCA_001600015.1
Holtermanniella nyarrowii	GCA_001600035.1
Filobasidium wieringae	GCA_001600055.1
Ustilaginoidea virens	GCA_016803955.1
Saccharomycodes ludwigii	GCA_016806925.1
Aureobasidium melanogenum	GCA_016807825.1
Colletotrichum eleusines	GCA_016807845.1
Valsa sordida	GCA_016808195.1

Thielaviopsis cerberus	GCA_016859225.1
Diaporthe destruens	GCA_016859255.1
Aspergillus luchuensis	GCA_016861625.1
Aspergillus chevalieri	GCA_016861735.1
Aspergillus puulaauensis	GCA_016861865.1
Aspergillus sp. 2663	GCA_016880755.1
Phymatotrichopsis omnivora	GCA_016880775.1
Novakomyces olei	GCA_016906335.1
Corynespora cassiicola	GCA_016906425.1
Bullera alba	GCA_001600095.1
Vanrija humicola	GCA_001600235.1
Brettanomyces sp. HC-2020a	GCA_016906875.1
Erysiphe necator	GCA_016906895.1
Stagonosporopsis cucurbitacearum	GCA_016920805.1
Neocallimastix sp. JGI-2020a	GCA_016946835.1
Fusarium culmorum	GCA_016952355.1
Trichocladium uniseriatum	GCA_017139535.1
Ambrosiella cleistominuta	GCA_017139545.1
Fusarium devonianum	GCA_017140155.1
Fusarium staphyleae	GCA_017140175.1
Calonectria hongkongensis	GCA_017140755.1
Calonectria pauciramosa	GCA_017140785.1
Arthrinium sp. KUC21332	GCA_017163955.1
Fusarium sacchari	GCA_017165645.1
Pneumocystis wakefieldiae	GCA_017301755.1
[Candida] nivariensis	GCA_017309295.1
Elsinoe batatas	GCA_017309325.1
Trichoderma brevicrassum	GCA_017311225.1
Pneumocystis canis	GCA_017311265.1
Pneumocystis oryctolagi	GCA_017311285.1
Cutaneotrichosporon curvatum	GCA_001600275.1
Kazachstania slooffiae	GCA_017347545.1
Podospora comata	GCA_017354895.1
Monilinia vaccinii-corymbosi	GCA_017357885.1
Cadophora malorum	GCA_017365405.1
Cadophora sp. M221	GCA_017366145.1
Kluyveromyces sp. PCH397	GCA_017427045.1
Apiotrichum brassicae	GCA_001600295.1
Apiotrichum gamsii	GCA_001600315.1

Fusarium redolens	GCA_017562085.1
Fusarium sororula	GCA_017579625.1
Aureobasidium zeae	GCA_017580825.1
Apiotrichum gracile	GCA_001600335.1
Mrakia frigida	GCA_001600395.1
Yarrowia sp. B02	GCA_017635955.1
Yarrowia sp. E02	GCA_017639365.1
Yarrowia sp. C11	GCA_017639385.1
Candida metapsilosis	GCA_017655625.1
Incumbomyces delicatus	GCA_017655985.1
Incumbomyces lentus	GCA_017656005.1
Fusarium vorosii	GCA_017656575.1
Fusarium sp. CBS 123663	GCA_017656595.1
Fusarium nepalense	GCA_017656675.1
Fusarium ussurianum	GCA_017656695.1
Fusarium mesoamericanum	GCA_017656745.1
Fusarium louisianense	GCA_017656775.1
Fusarium meridionale	GCA_017656785.1
Fusarium meridionale x *Fusarium asiaticum*	GCA_017656815.1
Fusarium gerlachii	GCA_017656835.1
Fusarium cortaderiae	GCA_017656915.1
Fusarium brasilicum	GCA_017656955.1
Fusarium boothii	GCA_017656985.1
Fusarium austroamericanum	GCA_017657035.1
Fusarium aethiopicum	GCA_017657045.1
Fusarium acaciae-mearnsii	GCA_017657105.1
Scopulariopsis brevicaulis	GCA_017657125.1
Apiotrichum veenhuisii	GCA_001600595.1
Dioszegia crocea	GCA_001600615.1
Dioszegia aurantiaca	GCA_001600655.1
Cutaneotrichosporon cutaneum	GCA_001600715.1
Apiotrichum laibachii	GCA_001600735.1
Saitozyma sp. JCM 24511	GCA_001600855.1
Solicoccozyma terricola	GCA_001600875.1
Cutaneotrichosporon cutaneum	GCA_001613755.1
Cryptococcus depauperatus CBS 7855	GCA_001720245.1
Cunninghamella blakesleeana	GCA_017871115.1
Trichosporon faecale	GCA_001752585.1
Xeromyces bisporus	GCA_900006255.1

Trichosporon coremiiforme	GCA_001752605.1
Fusarium mangiferae	GCA_900044065.1
Trichosporon inkin	GCA_001752625.1
Trichosporon ovoides	GCA_001752645.1
Mrakia psychrophila	GCA_001889225.1
Naematelia encephala	GCA_002105065.1
Cutaneotrichosporon arboriforme	GCA_002335565.1
Cutaneotrichosporon daszewskae	GCA_002335585.1
Prillingera fragicola	GCA_002335605.1
Candida metapsilosis	GCA_900069165.1
Phialocephala subalpina	GCA_900073065.1
Lachancea meyersii CBS 8951	GCA_900074715.1
Lachancea dasiensis	GCA_900074725.1
Lachancea sp. CBS 6924	GCA_900074735.1
Lachancea mirantina	GCA_900074745.1
Lachancea nothofagi CBS 11611	GCA_900074755.1
Lachancea fermentati	GCA_900074765.1
Fusarium culmorum	GCA_900074845.1
Rhynchosporium commune	GCA_900074885.1
Rhynchosporium secalis	GCA_900074895.1
Rhynchosporium agropyri	GCA_900074905.1
Absidia glauca	GCA_900079185.1
Cutaneotrichosporon cyanovorans	GCA_002335625.1
Neoscytalidium dimidiatum	GCA_900092665.1
Apiotrichum akiyoshidainum	GCA_002973495.1
Cryptococcus gattii VGII	GCA_003011995.1
[Candida] intermedia	GCA_900106115.1
Malbranchea cinnamomea	GCA_900128795.2
Takashimella koratensis	GCA_003116875.1
Cutaneotrichosporon dermatis	GCA_003116895.1
Takashimella tepidaria	GCA_003116915.1
Cutaneotrichosporon mucoides	GCA_003116955.1
Zygosaccharomyces bailii	GCA_900162805.1
Saitozyma podzolica	GCA_003942215.1
Lecanicillium fungicola 150-1	GCA_900169235.1
Rhizomucor pusillus	GCA_900175165.2
Kazachstania saulgeensis	GCA_900180425.1
Hymenoscyphus fraxineus	GCA_900184765.1
Torulaspora microellipsoides	GCA_900186055.1

[Candida] psychrophila	GCA_900186205.1
Geosmithia flava	GCA_900188565.1
Geosmithia putterillii	GCA_900188575.1
Blumeria hordei DH14	GCA_900239735.1
Saccharomyces jurei	GCA_900290405.1
Podospora comata	GCA_900290415.1
Sarocladium strictum	GCA_900290465.1
Moniliella sp. *'wahieum'*	GCA_003971905.1
Cryptococcus floricola	GCA_006352305.1
Fusarium tricinctum	GCA_900382705.2
Zygosaccharomyces pseudobailii	GCA_900408955.1
Leptosphaeria biglobosa 'brassicae' group	GCA_900465125.1
Zygosaccharomyces sapae	GCA_900465325.1
[Candida] vartiovaarae	GCA_900465365.1
Saccharomycodes ludwigii	GCA_900491785.1
Brettanomyces bruxellensis	GCA_900496985.1
Magnusiomyces ingens NRRL Y-17630	GCA_900497715.1
Magnusiomyces capitatus NRRL Y-17686	GCA_900497725.1
Yarrowia alimentaria	GCA_900518985.1
Yarrowia yakushimensis	GCA_900518995.1
Yarrowia phangngaensis	GCA_900519005.1
Yarrowia osloensis	GCA_900519015.1
Yarrowia porcina	GCA_900519025.1
Yarrowia divulgata	GCA_900519045.1
Yarrowia galli	GCA_900519055.1
Yarrowia hollandica	GCA_900519065.1
Yarrowia bubula	GCA_900519075.1
Yarrowia deformans	GCA_900519085.1
Blumeria graminis f. sp. *tritici*	GCA_900519115.1
Cryptococcus neoformans AD hybrid	GCA_006992865.1
[Candida] hispaniensis	GCA_900535975.1
Hymenoscyphus linearis	GCA_900536425.1
Hymenoscyphus occultus	GCA_900536445.1
Vanrija humicola	GCA_008065275.1
Cyanodermella asteris	GCA_900618795.1
Paradendryphiella salina	GCA_900634815.1
Saprochaete suaveolens	GCA_900642975.1
Saprochaete fungicola	GCA_900654225.1
Hymenoscyphus koreanus	GCA_902652825.1

Cryptococcus cf. *gattii*	GCA_009650685.1
Papiliotrema laurentii	GCA_012922615.1
Daldinia concentrica	GCA_902805455.1
Annulohypoxylon truncatum	GCA_902805465.1
Entonaema liquescens	GCA_902805475.1
Hypomontagnella submonticulosa	GCA_902806495.1
Hypoxylon lienhwacheense	GCA_902806505.1
Hypoxylon fragiforme	GCA_902806515.1
Hypoxylon pulicicidum	GCA_902806525.1
Hypoxylon rickii	GCA_902806535.1
Hypoxylon rubiginosum	GCA_902806565.1
Jackrogersella multiformis	GCA_902806575.1
Xylaria hypoxylon	GCA_902806585.1
Pyrenopolyporus hunteri	GCA_902806595.1
Myriosclerotinia sulcatula	GCA_902810775.1
Hypomontagnella monticulosa	GCA_902825475.1
Kazachstania barnettii	GCA_903064755.1
Sclerotinia trifoliorum	GCA_905066765.1
Talaromyces marneffei	GCF_000001985.1
Pyricularia oryzae	GCF_000002495.2
Kluyveromyces lactis	GCF_000002515.2
Yarrowia lipolytica	GCF_000002525.2
Candida glabrata	GCF_000002545.3
Aspergillus fumigatus	GCF_000002655.1
Aspergillus clavatus	GCF_000002715.2
Aspergillus niger	GCF_000002855.3
Schizosaccharomyces pombe	GCF_000002945.1
Talaromyces stipitatus	GCF_000003125.1
Uncinocarpus reesii	GCF_000003515.1
Clavispora lusitaniae	GCF_000003835.1
Blastomyces gilchristii	GCF_000003855.2
Schizosaccharomyces cryophilus	GCF_000004155.1
Candida tropicalis	GCF_000006335.3
Debaryomyces hansenii	GCF_000006445.2
Zygosaccharomyces rouxii	GCF_000026365.1
Candida dubliniensis	GCF_000026945.1
Komagataella phaffii	GCF_000027005.1
Eremothecium gossypii	GCF_000091025.4
Naganishia sp. IF7SW-B1	GCA_012922685.1

Lachancea thermotolerans	GCF_000142805.1
Grosmannia clavigera	GCF_000143105.1
Naganishia adeliensis	GCA_012922715.1
Chaetomium globosum	GCF_000143365.1
Botrytis cinerea	GCF_000143535.2
Apiotrichum mycotoxinovorans	GCA_013177335.1
Gaeumannomyces tritici	GCF_000145635.1
Parastagonospora nodorum	GCF_000146915.1
Sclerotinia sclerotiorum	GCF_000146945.2
Colletotrichum graminicola	GCF_000149035.1
Aspergillus nidulans	GCF_000149205.2
Coccidioides immitis	GCF_000149335.2
Meyerozyma guilliermondii	GCF_000149425.1
Fusarium verticillioides	GCF_000149555.1
Histoplasma mississippiense	GCF_000149585.1
Aspergillus terreus?	GCF_000149615.1
Aspergillus fischeri	GCF_000149645.2
Lodderomyces elongisporus	GCF_000149685.1
Schizosaccharomyces japonicus	GCF_000149845.2
Naganishia liquefaciens	GCA_013423385.1
Fusarium oxysporum	GCF_000149955.1
Pyrenophora tritici-repentis	GCF_000149985.1
Vanderwaltozyma polyspora	GCF_000150035.1
Schizosaccharomyces octosporus	GCF_000150505.1
Verticillium dahliae	GCF_000150675.1
Paracoccidioides lutzii	GCF_000150705.2
Paracoccidioides brasiliensis	GCF_000150735.1
Verticillium alfalfae	GCF_000150825.1
Nannizzia gypsea	GCF_000150975.2
Trichophyton benhamiae	GCF_000151125.1
Microsporum canis	GCF_000151145.1
Coccidioides posadasii	GCF_000151335.2
Fusarium vanettenii	GCF_000151355.1
Trichophyton rubrum	GCF_000151425.1
Trichophyton verrucosum	GCF_000151505.1
Tuber melanosporum	GCF_000151645.1
Trichoderma reesei	GCF_000167675.1
Trichoderma virens	GCF_000170995.1
Trichoderma atroviride	GCF_000171015.1

Naganishia randhawae	GCA_013461525.1
Spizellomyces punctatus	GCF_000182565.1
Candida parapsilosis	GCF_000182765.1
Sordaria macrospora	GCF_000182805.2
Phaffia rhodozyma	GCA_014706385.1
Neurospora crassa	GCF_000182925.2
Sordaria macrospora	GCF_000182965.3
Cystofilobasidium bisporidii	GCA_014825535.1
Aspergillus oryzae	GCF_000184455.2
Cystofilobasidium capitatum	GCA_014825545.1
Ogataea parapolymorpha	GCF_000187245.1
Metarhizium acridum	GCF_000187405.1
Metarhizium robertsii	GCF_000187425.2
Batrachochytrium dendrobatidis	GCF_000203795.1
Phaffia tasmanica	GCA_014825565.1
Scheffersomyces stipitis	GCF_000209165.1
Neurospora tetrasperma	GCF_000213175.1
Phaffia australis	GCA_014825575.1
Zymoseptoria tritici	GCF_000219625.1
Thermochaetoides thermophila	GCF_000221225.1
Yamadazyma tenuis	GCF_000223465.1
Spathaspora passalidarum NRRL Y-27907	GCF_000223485.1
Orbilia oligospora ATCC 24927	GCF_000225545.1
Cordyceps militaris CM01	GCF_000225605.1
Thermothelomyces thermophilus ATCC 42464	GCF_000226095.1
Thermothielavioides terrestris NRRL 8126	GCF_000226115.1
Penicillium rubens Wisconsin 54-1255	GCF_000226395.1
Podospora anserina S mat+	GCF_000226545.1
Naumovozyma dairenensis CBS 421	GCF_000227115.2
Leptosphaeria maculans JN3	GCF_000230375.1
Exophiala dermatitidis NIH/UT8656	GCF_000230625.1
Eremothecium cymbalariae DBVPG#7215	GCF_000235365.1
Tetrapisispora phaffii CBS 4417	GCF_000236905.1
Naumovozyma castellii CBS 4309	GCF_000237345.1
Fusarium graminearum PH-1	GCF_000240135.3
Torulaspora delbrueckii	GCF_000243375.1
Fusarium odoratissimum	GCF_000260195.1
Cystofilobasidium ferigula	GCA_014825675.1

Cystofilobasidium macerans	GCA_014825765.1
Krasilnikovozyma curviuscula	GCA_014825775.1
Papiliotrema laurentii	GCA_015585935.1
Naganishia vishniacii ANT03-052	GCA_015708705.1
Cryptococcus neoformans	GCF_000091045.1
Cryptococcus gattii	GCF_000185945.1
Tremella mesenterica DSM 1558	GCF_000271645.1
Trichosporon asahii var. *asahii* CBS 2479	GCF_000293215.1
Beauveria bassiana ARSEF 2860	GCF_000280675.1
Coniosporium apollinis CBS 100218	GCF_000281105.1
Saccharomyces arboricola H-6	GCF_000292725.1
Kwoniella mangroviensis	GCF_000507465.1
Drepanopeziza brunnea f. sp. *'multigermtubi' MB_m1*	GCF_000298775.1
Kwoniella dejecticola	GCF_000512565.1
Kwoniella bestiolae	GCF_000512585.1
Fusarium pseudograminearum CS3096	GCF_000303195.2
Kazachstania africana CBS 2517	GCF_000304475.1
Wickerhamomyces ciferrii	GCF_000313485.1
Kwoniella pini	GCF_000512605.1
Penicillium digitatum	GCF_000315645.1
Candida orthopsilosis Co 90-125	GCF_000315875.1
Tetrapisispora blattae CBS 6284	GCF_000315915.1
Sphaerulina musiva	GCF_000320565.1
Cutaneotrichosporon oleaginosum	GCF_001027345.1
Cryptococcus wingfieldii CBS 7118	GCF_001720155.1
Cryptococcus amylolentus CBS 6039	GCF_001720205.1
Baudoinia panamericana UAMH 10762	GCF_000338955.1
Bipolaris sorokiniana ND90Pr	GCF_000338995.1
Pseudocercospora fijiensis CIRAD86	GCF_000340215.1
Kockovaella imperatae	GCF_002102565.1
Kazachstania naganishii CBS 8797	GCF_000348985.1
Pneumocystis murina B123	GCF_000349005.2
Apiotrichum porosum	GCF_003942205.1
Eutypa lata UCREL1	GCF_000349385.1
Bipolaris maydis ATCC 48331	GCF_000354255.1
Exserohilum turcica Et28A	GCF_000359705.1
Cyphellophora europaea	GCF_000365145.1
Cladophialophora carrionii	GCF_000365165.1

Neofusicoccum parvum UCRNP2	GCF_000385595.1
Phaeoacremonium minimum UCRPA7	GCF_000392275.1
Kwoniella shandongensis	GCF_008629635.1
Moesziomyces aphidis	GCA_000517465.1
Glarea lozoyensis ATCC 20868	GCF_000409485.1
Ustilago esculenta	GCA_000819925.1
Rhizophagus irregularis	GCF_000439145.1
Dactylellina haptotyla CBS 200.50	GCF_000441935.1
Endocarpon pusillum Z07020	GCF_000464535.1
Sporisorium scitamineum	GCA_001010845.1
Ustilago vetiveriae	GCA_001735935.1
Ustilago xerochloae	GCA_001736075.1
Pseudozyma tsukubaensis	GCA_001736125.1
Ustilago cynodontis	GCA_001736195.1
Pestalotiopsis fici W106-1	GCF_000516985.1
Bipolaris zeicola 26-R-13	GCF_000523435.1
Bipolaris oryzae ATCC 44560	GCF_000523455.1
Bipolaris victoriae FI3	GCF_000527765.1
Kuraishia capsulata CBS 1993	GCF_000576695.1
Cladophialophora yegresii CBS 114405	GCF_000585515.1
Cladophialophora psammophila CBS 110553	GCF_000585535.1
Capronia epimyces CBS 606.96	GCF_000585565.1
Capronia coronata CBS 617.96	GCF_000585585.1
Sporisorium iseilematis-ciliati	GCA_001748505.1
Exophiala aquamarina CBS 119918	GCF_000709125.1
Ustilago tritici	GCA_002993085.1
Aureobasidium subglaciale EXF-2481	GCF_000721755.1
Aureobasidium namibiae CBS 147.97	GCF_000721765.1
Aureobasidium pullulans EXF-150	GCF_000721785.1
Scedosporium apiospermum	GCF_000732125.1
Pseudozyma sp. F8B2	GCA_003004685.1
Penicillium expansum	GCF_000769745.1
Metarhizium majus ARSEF 297	GCF_000814945.1
Metarhizium brunneum ARSEF 3297	GCF_000814965.1
Fonsecaea pedrosoi CBS 271.37	GCF_000835455.1
Cladophialophora bantiana CBS 173.52	GCF_000835475.1
Cladophialophora immunda	GCF_000835495.1
Exophiala xenobiotica	GCF_000835505.1
Exophiala oligosperma	GCF_000835515.1

Rhinocladiella mackenziei CBS 650.93	GCF_000835555.1
Exophiala spinifera	GCF_000836115.1
Exophiala mesophila	GCF_000836275.1
Verruconis gallopava	GCF_000836295.1
Fonsecaea multimorphosa CBS 102226	GCF_000836435.1
Lachancea lanzarotensis	GCF_000938715.1
Sporothrix schenckii 1099-18	GCF_000961545.1
Rasamsonia emersonii CBS 393.64	GCF_000968595.1
Moesziomyces sp. F5C1	GCA_003004715.1
Aspergillus nomiae NRRL 13137	GCF_001204775.2
Moesziomyces sp. F16C1	GCA_003004725.1
Saccharomyces eubayanus	GCF_001298625.1
Phialophora attinorum	GCF_001299255.1
Ustilago hordei	GCA_003012045.1
Kluyveromyces marxianus DMKU3-1042	GCF_001417885.1
Debaryomyces fabryi	GCF_001447935.2
Pneumocystis jirovecii RU7	GCF_001477535.1
Pneumocystis carinii B80	GCF_001477545.1
Trichoderma gamsii	GCF_001481775.2
Mollisia scopiformis	GCF_001500285.1
Xylona heveae TC161	GCF_001619985.1
Cordyceps fumosorosea ARSEF 2679	GCF_001636725.1
Phycomyces blakesleeanus	GCF_001638985.1
Sugiyamaella lignohabitans	GCF_001640025.1
Pseudogymnoascus destructans	GCF_001641265.1
Paraphaeosphaeria sporulosa	GCF_001642045.1
Alternaria alternata	GCF_001642055.1
Fonsecaea monophora	GCF_001642475.1
Fonsecaea nubica	GCF_001646965.1
Fonsecaea erecta	GCF_001651985.1
Pochonia chlamydosporia 170	GCF_001653235.2
Purpureocillium lilacinum	GCF_001653265.1
Pichia membranifaciens NRRL Y-2026	GCF_001661235.1
Wickerhamomyces anomalus NRRL Y-366-8	GCF_001661255.1
Saitoella complicata NRRL Y-17804	GCF_001661265.1
Babjeviella inositovora NRRL Y-12698	GCF_001661335.1
Ascoidea rubescens DSM 1968	GCF_001661345.1
Hyphopichia burtonii NRRL Y-1933	GCF_001661395.1
Cyberlindnera jadinii NRRL Y-1542	GCF_001661405.1

Suhomyces tanzawaensis NRRL Y-17324	GCF_001661415.1
Pseudogymnoascus verrucosus	GCF_001662655.1
Metschnikowia bicuspidata var. *bicuspidata* NRRL YB-4993	GCF_001664035.1
Ogataea polymorpha	GCF_001664045.1
Colletotrichum higginsianum IMI 349063	GCF_001672515.1
Testicularia cyperi	GCA_003144125.1
Violaceomyces palustris	GCA_003144235.1
Penicillium arizonense	GCF_001773325.1
Aspergillus bombycis	GCF_001792695.1
Colletotrichum orchidophilum	GCF_001831195.1
Diplodia corticola	GCF_001883845.1
Penicilliopsis zonata CBS 506.65	GCF_001890105.1
Aspergillus glaucus CBS 516.65	GCF_001890805.1
Aspergillus aculeatus ATCC 16872	GCF_001890905.1
Talaromyces atroroseus	GCF_001907595.1
Saccharomyces paradoxus	GCF_002079055.1
Anthracocystis panici-leucophaei	GCA_014826065.1
Lobosporangium transversale	GCF_002105155.1
Ustilago bromivora	GCA_900080155.1
Aspergillus thermomutatus	GCF_002237265.1
Wickerhamiella sorbophila	GCF_002251995.1
Rhizopus microsporus ATCC 52813	GCF_002708625.1
Cercospora beticola	GCF_002742065.1
[Candida] auris	GCF_002775015.1
Aspergillus candidus	GCF_002847045.1
Aspergillus novofumigatus IBT 16806	GCF_002847465.1
Aspergillus campestris IBT 28561	GCF_002847485.1
Aspergillus steynii IBT 23096	GCF_002849105.1
Hyaloscypha bicolor E	GCF_002865645.1
Candida haemuloni	GCF_002926055.2
Candida duobushaemulonis	GCF_002926085.2
[Candida] pseudohaemulonii	GCF_003013735.1
Amorphotheca resinae ATCC 22711	GCF_003019875.1
Trichoderma harzianum CBS 226.95	GCF_003025095.1
Trichoderma asperellum CBS 433.97	GCF_003025105.1
Trichoderma citrinoviride	GCF_003025115.1
Pichia kudriavzevii	GCF_003054445.1
Sporisorium reilianum f. sp. *reilianum*	GCA_900162835.1

Thecaphora thlaspeos	GCA_900291925.1
Ustilago trichophora	GCA_900323505.1
Ustilago hordei	GCA_900519145.1
Ustilago maydis 521	GCF_000328475.2
Pseudozyma hubeiensis SY62	GCF_000403515.1
Aspergillus sclerotioniger CBS 115572	GCF_003184525.1
Aspergillus eucalypticola CBS 122712	GCF_003184535.1
Aspergillus heteromorphus CBS 117.55	GCF_003184545.1
Aspergillus saccharolyticus JOP 1030-1	GCF_003184585.1
Aspergillus neoniger CBS 115656	GCF_003184625.1
Aspergillus brunneoviolaceus CBS 621.78	GCF_003184695.1
Aspergillus uvarum CBS 121591	GCF_003184745.1
Aspergillus piperis CBS 112811	GCF_003184755.1
Aspergillus aculeatinus CBS 121060	GCF_003184765.1
Aspergillus japonicus CBS 114.51	GCF_003184785.1
Aspergillus costaricaensis CBS 115574	GCF_003184835.1
Aspergillus ibericus CBS 121593	GCF_003184845.1
Aspergillus homomorphus CBS 101889	GCF_003184865.1
Aspergillus vadensis CBS 113365	GCF_003184925.1
Pseudozyma flocculosa PF-1	GCF_000417875.1
Fusarium coffeatum	GCF_003316985.1
Candida viswanathii	GCF_003327735.1
Aspergillus welwitschiae	GCF_003344945.1
Venustampulla echinocandica	GCF_003357145.1
Aspergillus mulundensis	GCF_003369625.1
Aspergillus tanneri	GCF_003426965.1
Sodiomyces alkalinus F11	GCF_003711515.1
Verticillium nonalfalfae	GCF_003724135.2
Kalmanozyma brasiliensis GHG001	GCF_000497045.1
Moesziomyces antarcticus	GCF_000747765.1
Paecilomyces variotii	GCF_004022145.1
Alternaria arborescens	GCF_004154835.1
Pyricularia pennisetigena	GCF_004337985.1
Thyridium curvatum	GCF_004353045.1
Pyricularia grisea	GCF_004355905.1
Sporisorium graminicola	GCF_005498985.1
Synchytrium microbalum	GCF_006535985.1
Lachnellula hyalina	GCF_007821495.1
Wallemia hederae	GCA_004918325.1

Daldinia childiae	GCF_008694065.1
Diutina rugosa	GCF_008704595.1
Aspergillus alliaceus	GCF_009176365.1
Aspergillus pseudotamarii	GCF_009193445.1
Aspergillus caelatus	GCF_009193585.1
Aspergillus pseudonomiae	GCF_009193645.1
Colletotrichum fructicola	GCF_009771025.1
Dissoconium aciculare CBS 342.82	GCF_010015565.1
Eremomyces bilateralis CBS 781.70	GCF_010015585.1
Aaosphaeria arxii CBS 175.79	GCF_010015735.1
Dothidotthia symphoricarpi CBS 119687	GCF_010015815.1
Neohortaea acidophila	GCF_010093505.1
Lindgomyces ingoldianus	GCF_010093535.1
Mytilinidion resinicola	GCF_010093595.1
Macroventuria anomochaeta	GCF_010093625.1
Pseudovirgaria hyperparasitica	GCF_010093815.1
Aplosporella prunicola CBS 121167	GCF_010093885.1
Zasmidium cellare ATCC 36951	GCF_010093935.1
Trematosphaeria pertusa	GCF_010094035.1
Westerdykella ornata	GCF_010094085.1
Didymella exigua CBS 183.55	GCF_010094145.1
Aspergillus lentulus	GCF_010724455.1
Colletotrichum scovillei	GCF_011075155.1
Arthroderma uncinatum	GCF_011692745.1
Geosmithia morbida	GCF_012550715.1
Lasiodiplodia theobromae	GCF_012971845.1
Aspergillus tubingensis	GCF_013340325.1
Talaromyces rugulosus	GCF_013368755.1
Colletotrichum aenigma	GCF_013390185.1
Colletotrichum siamense	GCF_013390195.1
Fusarium subglutinans	GCF_013396075.1
Fusarium tjaetaba	GCF_013396195.1
Zygotorulaspora mrakii	GCF_013402915.1
Botrytis fragariae	GCF_013461495.1
Letharia columbiana	GCF_014066305.1
Letharia lupina	GCF_014066315.1
Torulaspora globosa	GCF_014133895.1
Colletotrichum truncatum	GCF_014235925.1
Wallemia mellicola CBS 633.66	GCF_000263375.1

Wallemia ichthyophaga EXF-994	GCF_000400465.1
Fusarium venenatum	GCF_900007375.1
Fusarium proliferatum ET1	GCF_900067095.1
Ramularia collo-cygni	GCF_900074925.1
Fusarium fujikuroi IMI 58289	GCF_900079805.1
Saprochaete ingens	GCF_902498895.1
Dictyocoela roeselum	GCA_016255985.1
Dictyocoela muelleri	GCA_016256075.1
Acremonium chrysogenum	JPKY01000541.1
Allantophomopsis cytisporea	CP103038.1
Allantophomopsis lycopodina	CP103019.1
Aspergillus parasiticus	CM023211.1
Aspergillus pseudoviridinutans	AP024468.1
Beauveria bassiana	CM020964.1
Blastobotrys aristatus	OX291664.1
Calonectria hawksworthii	CM037207.1
Candida albicans	CP025166.1
Candida jiufengensis	NC_014693.1
Candida margitis	OM763983.1
Candida oxycetoniae	NC_022162.1
Candida pseudojiufengensi	NC_022156.1
Cercospora sp. J1 LZ-2022	CP095198.1
Chrysoporthe puriensis	CP064901.1
Cladonia squamosa	OX392398.1
Coccidioides posadasii str. *Silveira*	CP075680.2
Colletotrichum gigasporum	CP077946.1
Colletotrichum lupini	NC_029213.1/CP019482.1
Cyberlindnera sargentensis	CP083471.1
Emydomyces testavorans	CP120632.1
Epichloe amarillans	CP099640.1
Epichloe baconii	CP097909.1
Epichloe brachyelytri	CP097982.1
Epichloe bromicola Nfe1	CP098272.1
Epichloe festucae	CP031392.1
Epichloe gansuensis	CP098288.1
Epichloe glyceriae	CP098296.1
Epichloe scottii	CP083252.1
Epichloe typhina	CP098312.1
Epichloe typhina subsp. *clarkii* 1605_22	CP064811.1

Epichloe typhina subsp. *poae* Pn1703_3	CP098304.1
Epichloe typhina subsp. *typhina* 1756	CP064803.1
Eremothecium sinecaudum	CP014249.1
Exophiala dermatitidis	CM021337.1
Exophiala sp. LBMH1013	CM045182.1
Fusarium graminearum	HG970331.1
Fusarium oxysporum f. sp. *apii*	CM026409.1/CM026407.1/CM026406.1
Fusarium oxysporum f. sp. *coriandrii*	CM026408.1/CM026405.1
Fusarium oxysporum f. sp. *fragariae* GL1080	CM029250.1
Fusarium oxysporum f. sp. *fragariae* GL1381	CM029251.1
Fusarium oxysporum f. sp. *lini* 39	CM022410.1
Fusarium oxysporum f. sp. *lini* 456	CM033783.1
Fusarium oxysporum f. sp. *lini* 476	CM033757.1
Fusarium oxysporum f. sp. *lini* 482	CM033784.1
Fusarium oxysporum f. sp. *lini* 483	CM033759.1
Fusarium oxysporum f. sp. *lini* 525	CM033758.1
Fusarium oxysporum f. sp. *lycopersici* 4287	CM010346.1
Fusarium oxysporum f. sp. *lycopersici* race 3	CM012197.1
Fusarium oxysporum f. sp. *matthiolae* PHW726	CM019668.1
Fusarium oxysporum f. sp. *radicis-cucumerinum* Forc016	CM008299.1
Fusarium oxysporum f. sp. *spinaciae*	CM056032.1/CM056034.1
Fusarium venenatum A3/5	NW_020311997.1/LN649234.1/CM033364.1
Glarea lozoyensis	KF169905.1
Histoplasma capsulatum	CP069113.1/CM029964.1/CP069123.1/CP069108.1
Histoplasma ohiense (nom. inval.) G217B	CM029948.1
Immersiporthe knoxdaviesiana	CP088216.1
Kluyveromyces marxianus	AP014607.1/CP118244.1/NC_036023.1/AP012221.1
Komagataella kurtzmanii	CM039693.1
Komagataella phaffii	FR839632.1
Madurella fahalii	CM056961.1
Metarhizium brunneum	CP058939.1
Metschnikowia zobellii	OW618033.1
Microsporum audouinii	CM039285.1
Nakaseomyces glabratus	NC_004691.1/CM008094.1/CM008107.1
Nakaseomyces nivariensis	CM029591.1
Nannizziopsis arthrosporioides	CP098264.1
Nigrospora osmanthi	CP120502.1

Ogataea degrootiae	CM033062.1
Ophiocordyceps xuefengensis	CM037206.1
Penicillium roqueforti UASWS P1	KR952335.1/NC_027416.1
Penicillium solitum	NC_016187.1
Podospora anserina	CM030339.1
Pseudopithomyces maydicus	CM049194.1
Purpureocillium lavendulum	CM054929.1
Pyricularia oryzae B71	CP060338.1
Pyronema omphalodes CBS 100304	NC_029745.1/KU707476.1
Saccharomyces arboricola	CM001579.1
Saccharomyces bayanus	CP080221.1
Saccharomyces cerevisiae	CP011826.1
Saccharomyces mikatae	CM057025.1
Sclerotinia nivalis	CM055954.1
Septoria linicola	CP099434.1/CM044304.1
Sugiyamaella lignohabitans	CP014499.1
Talaromyces trachyspermus	CM035349.1
Tolypocladium tundrense	CM044411.1
Torulaspora quercuum	OX291449.1
Trichoderma asperellum	KR952346.1/CP084950.1
Trichoderma atroviride	CP084942.1
Trichoderma pseudokoningii	OW971927.1
Venturia effusa albino	CP042205.1
Xeromyces bisporus FRR 0525	HG983520.1
Zygosaccharomyces parabailii ATCC 60483	CP019506.1
Zygotorulaspora mrakii NRRL Y-6702	NW_023500906.1/CP058612.1
Agaricus bisporus	OW971919.1
Agaricus bisporus var. *bisporus* ARP23	CM017010.1
Agaricus subrufescens	CM057070.1
Anthracocystis panici-leucophaei SPL10	CP060321.1
Antrodia cinnamomea	CM040236.1/CM040235.1/CM040234.1/CM040237.1
Cryptococcus neoformans var. *grubii*	CP022335.1
Cryptococcus wingfieldii CBS7118	CP034275.1
Exobasidium cylindrosporum YG638	CP096881.1
Exobasidium rhododendri	CP110633.1
Inonotus obliquus NBRC113408	LC497415.1
Inosperma sp. D86	CM048062.1
Malassezia globosa CBS7966	CP046440.1

Malassezia sympodialis ATCC 42132	LT671829.1
Marasmius oreades	NW_025064824.1/CM032192.1
Phanerochaete sordida	LC707859.1
Pleurotus ostreatus P51	NC_009905.1/OX344747.1/OX344748.1
Psilocybe cubensis MGC-MH-2018	NW_025952838.1/CM029846.1
Puccinia striiformis	CM009486.1/CM009485.1
Pyrrhoderma noxium FFPRI411160	CM008263.2
Rhizoctonia solani AG-1 IB isolate 7/3/14	HF546977.1
Schizophyllum commune IUM1114-SS01	CM026448.1
Sporisorium reilianum SRZ2	FQ311469.1
Sporisorium scitamineum SSC39B	CP010939.1
Symmetrospora coprosmae UCD350	CM018325.1
Taiwanofungus camphoratus M8	CP021234.1
Trichosporon asahii var. *asahii*	NW_014040924.1/CM001777.1/JH925097.1
Ustilago bromivora UB2112	LT558140.1
Vanrija pseudolonga	CP086722.1
Paramicrosporidium saccamoebae KSL3	CM008827.1
Entomortierella parvispora E1425	LC659289.1
Mucor piriformis	OW971873.1
Glomus cerebriforme	NC_022144.1
Gigaspora rosea	NC_016985.1
Lichtheimia ramosa	NC_024200.1
Gigaspora margarita	NC_016684.1
Rhizophagus clarus	LC506577.1
Absidia glauca	NC_036158.1
Rhizophagus irregularis	OP796669.1
Zancudomyces culisetae	NC_006837.1
Synchytrium endobioticum	NC_042370.1
Chytriomyces confervae	NC_042880.1
Synchytrium microbalum	NC_042878.1
Notamacropus eugenii	GCA_000004035.1
Mchenga conophoros	GCA_000150855.1
Labeotropheus fuelleborni	GCA_000150875.1
Melanochromis auratus	GCA_000150895.1
Rhamphochromis esox	GCA_000150935.1
Choloepus hoffmanni	GCA_000164785.2
Tetraodon nigroviridis	GCA_000180735.1
Tupaia belangeri	GCA_000181375.1
Leucoraja erinacea	GCA_000238235.1

Amazona vittata	GCA_000332375.2
Apalone spinifera	GCA_000385615.1
Ara macao	GCA_000400695.1
Eidolon helvum	GCA_000465285.1
Pteronotus parnellii	GCA_000465405.1
Lethenteron camtschaticum	GCA_000466285.1
Sebastes rubrivinctus	GCA_000475215.1
Sebastes nigrocinctus	GCA_000475235.3
Anoplopoma fimbria	GCA_000499045.2
Ophiophagus hannah	GCA_000516915.1
Lyrurus tetrix tetrix	GCA_000586395.1
Cavia aperea	GCA_000688575.1
Dicentrarchus labrax	GCA_000689215.1
Pampus argenteus	GCA_000697985.1
Podiceps cristatus	GCA_000699545.1
Cathartes aura	GCA_000699945.1
Crotalus pyrrhus	GCA_000737285.1
Capreolus capreolus	GCA_000751575.1
Nasalis larvatus	GCA_000772465.1
Gavialis gangeticus	GCA_000775435.1
Cyprinodon nevadensis pectoralis	GCA_000776015.1
Periophthalmodon schlosseri	GCA_000787095.1
Scartelaos histophorus	GCA_000787155.1
Pseudopleuronectes yokohamae	GCA_000787555.1
Vipera berus berus	GCA_000800605.1
Capra aegagrus	GCA_000978405.1
Balaenoptera bonaerensis	GCA_000978805.1
Eulemur macaco	GCA_001262655.1
Eulemur flavifrons	GCA_001262665.1
Zosterops lateralis melanops	GCA_001281735.1
Apodemus sylvaticus	GCA_001305905.1
Amazona aestiva	GCA_001420675.1
Cottus rhenanus	GCA_001455555.1
Miichthys miiuy	GCA_001593715.1
Anguilla rostrata	GCA_001606085.1
Mus spretus	GCA_001624865.1
Crotalus horridus	GCA_001625485.1
Phylloscopus trochiloides viridanus	GCA_001655095.1
Phylloscopus plumbeitarsus	GCA_001655115.1

Okapia johnstoni	GCA_001660835.1
Neotoma lepida	GCA_001675575.1
Ellobius lutescens	GCA_001685075.1
Ellobius talpinus	GCA_001685095.1
Mola mola	GCA_001698575.1
Passer domesticus	GCA_001700915.1
Phodopus sungorus	GCA_001707965.1
Malaclemys terrapin terrapin	GCA_001728815.2
Setophaga coronata coronata	GCA_001746935.2
Tympanuchus cupido pinnatus	GCA_001870855.1
Lycaon pictus	GCA_001887905.1
Sebastes minor	GCA_001910765.2
Sebastes steindachneri	GCA_001910785.2
Sebastes aleutianus	GCA_001910805.2
Ciconia boyciana	GCA_002002965.1
Grus japonensis	GCA_002002985.1
Hypotaenidia okinawae	GCA_002003005.1
Ailurus styani	GCA_002007465.1
Patagioenas fasciata monilis	GCA_002029285.1
Maccullochella peelii	GCA_002120245.1
Sporophila hypoxantha	GCA_002167245.1
Phalacrocorax pelagicus	GCA_002173435.1
Phalacrocorax auritus	GCA_002173455.1
Phalacrocorax harrisi	GCA_002173475.1
Phalacrocorax brasilianus	GCA_002174335.1
Eschrichtius robustus	GCA_002189225.1
Cervus elaphus hippelaphus	GCA_002197005.1
Ammotragus lervia	GCA_002201775.1
Psammomys obesus	GCA_002215935.2
Seriola quinqueradiata	GCA_002217815.1
Callipepla squamata	GCA_002218305.1
Anas zonorhyncha	GCA_002224875.1
Lithobates catesbeianus	GCA_002284835.2
Uria lomvia	GCA_002289315.1
Apodemus speciosus	GCA_002335545.1
Strix occidentalis	GCA_002372975.1
Spermophilus dauricus	GCA_002406435.1
Elaphurus davidianus	GCA_002443075.1
Tympanoctomys barrerae	GCA_002564285.1

Octomys mimax	GCA_002564305.1
Anser brachyrhynchus	GCA_002592135.1
Micropterus floridanus	GCA_002592385.1
Agapornis roseicollis	GCA_002631895.1
Symphodus melops	GCA_002819105.1
Limosa lapponica baueri	GCA_002844005.1
Acridotheres javanicus	GCA_002849675.1
Psittacula krameri	GCA_002870145.1
Coryphaenoides rupestris	GCA_002895965.1
Gopherus agassizii	GCA_002896415.1
Pagrus major	GCA_002897255.1
Bambusicola thoracicus	GCA_002909625.1
Ambystoma mexicanum	GCA_002915635.2
Seriola rivoliana	GCA_002994505.1
Amphiprion percula	GCA_003047355.2
Phocoena phocoena	GCA_003071005.2
Gambusia affinis	GCA_003097735.1
Sphenodon punctatus	GCA_003113815.1
Macaca fuscata fuscata	GCA_003118495.1
Paroedura picta	GCA_003118565.1
Ovis ammon	GCA_003121645.1
Pseudois nayaur	GCA_003182575.1
Capra sibirica	GCA_003182615.2
Tursiops aduncus	GCA_003227395.1
Thunnus thynnus	GCA_003231725.1
Prolemur simus	GCA_003258685.1
Pygoscelis papua	GCA_003264615.1
Spheniscus mendiculus	GCA_003264655.1
Chlorodrepanis virens	GCA_003286495.1
Hucho hucho	GCA_003317085.1
Mus spicilegus	GCA_003336285.1
Rhea pennata	GCA_003342835.1
Crypturellus cinnamomeus	GCA_003342915.1
Apteryx owenii	GCA_003342965.1
Apteryx haastii	GCA_003342985.1
Rhea americana	GCA_003343005.1
Ursus americanus	GCA_003344425.1
Ageneiosus marmoratus	GCA_003347165.1
Chlamydotis undulata undulata	GCA_003400225.2

Crotalus viridis viridis	GCA_003400415.2
Protobothrops flavoviridis	GCA_003402635.1
Corvus hawaiiensis	GCA_003402825.1
Chrysolophus pictus	GCA_003413605.1
Oplegnathus fasciatus	GCA_003416845.1
Eopsaltria australis	GCA_003426825.1
Chiloscyllium punctatum	GCA_003427335.1
Scyliorhinus torazame	GCA_003427355.1
Syrmaticus mikado	GCA_003435085.1
Thermophis baileyi	GCA_003457575.1
Eonycteris spelaea	GCA_003508835.1
Mixornis gularis	GCA_003546035.1
Oxygymnocypris stewartii	GCA_003573665.1
Salvator merianae	GCA_003586115.2
Anguilla japonica	GCA_003597225.1
Morone chrysops	GCA_003610055.1
Chloebia gouldiae	GCA_003676055.1
Calidris pygmaea	GCA_003697955.1
Taxidea taxus jeffersonii	GCA_003697995.1
Peromyscus polionotus subgriseus	GCA_003704135.2
Takifugu flavidus	GCA_003711565.2
Paradisaea rubra	GCA_003713215.1
Paradisaea raggiana	GCA_003713265.1
Diphyllodes magnificus	GCA_003713285.1
Parotia lawesii	GCA_003713295.1
Cicinnurus regius	GCA_003713305.1
Anabarilius grahami	GCA_003731715.1
Axis porcinus	GCA_003798545.1
Junco hyemalis	GCA_003829775.1
Cuora mccordi	GCA_003846335.1
Platysternon megacephalum	GCA_003942145.1
Oryx gazella	GCA_003945745.1
Amazona collaria	GCA_003947215.1
Clarias batrachus	GCA_003987875.1
Himantopus leucocephalus	GCA_003993805.1
Oryzias javanicus	GCA_003999625.1
Chiloscyllium plagiosum	GCA_004010195.1
Lateolabrax maculatus	GCA_004023545.1
Hydrophis cyanocinctus	GCA_004023725.1

Recurvirostra avosetta	GCA_004023745.1
Hydrophis hardwickii	GCA_004023765.1
Mungos mungo	GCA_004023785.1
Panthera onca	GCA_004023805.1
Vulpes lagopus	GCA_004023825.1
Helogale parvula	GCA_004023845.1
Mirounga angustirostris	GCA_004023865.1
Cryptoprocta ferox	GCA_004023885.1
Felis nigripes	GCA_004023925.1
Spilogale gracilis	GCA_004023965.1
Paradoxurus hermaphroditus	GCA_004024585.1
Pteronura brasiliensis	GCA_004024605.1
Mellivora capensis	GCA_004024625.1
Mirza coquereli	GCA_004024645.1
Lemur catta	GCA_004024665.1
Dipodomys stephensi	GCA_004024685.1
Moschus moschiferus	GCA_004024705.2
Catagonus wagneri	GCA_004024745.2
Zapus hudsonius	GCA_004024765.1
Ateles geoffroyi	GCA_004024785.1
Xerus inauris	GCA_004024805.1
Pygathrix nemaeus	GCA_004024825.1
Tonatia saurophila	GCA_004024845.1
Saguinus imperator	GCA_004024885.1
Tapirus indicus	GCA_004024905.1
Scalopus aquaticus	GCA_004024925.1
Uropsilus gracilis	GCA_004024945.1
Tragulus javanicus	GCA_004024965.2
Saiga tatarica	GCA_004024985.1
Tadarida brasiliensis	GCA_004025005.1
Tapirus terrestris	GCA_004025025.1
Sigmodon hispidus	GCA_004025045.1
Semnopithecus entellus	GCA_004025065.1
Thryonomys swinderianus	GCA_004025085.1
Tamandua tetradactyla	GCA_004025105.1
Tolypeutes matacus	GCA_004025125.1
Takifugu bimaculatus	GCA_004026145.2
Miniopterus schreibersii	GCA_004026525.1
Mormoops blainvillei	GCA_004026545.1

Noctilio leporinus	GCA_004026585.1
Ondatra zibethicus	GCA_004026605.1
Pithecia pithecia	GCA_004026645.1
Murina aurata feae	GCA_004026665.1
Nesogale talazaci	GCA_004026705.1
Myrmecophaga tridactyla	GCA_004026745.1
Micronycteris hirsuta	GCA_004026765.1
Lasiurus borealis	GCA_004026805.1
Hemitragus hylocrius	GCA_004026825.1
Heterohyrax brucei	GCA_004026845.1
Lepus americanus	GCA_004026855.1
Megaderma lyra	GCA_004026885.1
Hystrix cristata	GCA_004026905.1
Procavia capensis	GCA_004026925.2
Ovis canadensis	GCA_004026945.1
Petromus typicus	GCA_004026965.1
Muscardinus avellanarius	GCA_004027005.1
Myocastor coypus	GCA_004027025.1
Hippopotamus amphibius	GCA_004027065.2
Mesoplodon bidens	GCA_004027085.1
Daubentonia madagascariensis	GCA_004027145.1
Ctenomys sociabilis	GCA_004027165.1
Glis glis	GCA_004027185.1
Ctenodactylus gundi	GCA_004027205.1
Eulemur fulvus	GCA_004027275.1
Dolichotis patagonum	GCA_004027295.1
Erythrocebus patas	GCA_004027335.1
Macroglossus sobrinus	GCA_004027375.1
Hipposideros galeritus	GCA_004027415.1
Anoura caudifer	GCA_004027475.1
Beatragus hunteri	GCA_004027495.1
Antilocapra americana	GCA_004027515.2
Acomys cahirinus	GCA_004027535.1
Craseonycteris thonglongyai	GCA_004027555.1
Cricetomys gambianus	GCA_004027575.1
Dinomys branickii	GCA_004027595.1
Cercopithecus neglectus	GCA_004027615.1
Crocidura indochinensis	GCA_004027635.1
Graphiurus murinus	GCA_004027655.1

Cavia tschudii	GCA_004027695.1
Plecturocebus donacophilus	GCA_004027715.1
Carollia perspicillata	GCA_004027735.1
Cebus albifrons	GCA_004027755.1
Bradypus variegatus	GCA_004027775.1
Nycticebus coucang	GCA_004027815.1
Alouatta palliata	GCA_004027835.1
Aplodontia rufa	GCA_004027875.1
Orientallactaga bullata	GCA_004027895.1
Capromys pilorides	GCA_004027915.1
Chaetophractus vellerosus	GCA_004027955.1
Cirrhinus molitorella	GCA_004028445.1
Cuora amboinensis	GCA_004028625.2
Odocoileus hemionus hemionus	GCA_004115125.1
Collichthys lucidus	GCA_004119915.1
Poropuntius huangchuchieni	GCA_004124795.1
Hypophthalmichthys nobilis	GCA_004193235.1
Emydocephalus ijimae	GCA_004319985.1
Hydrophis melanocephalus	GCA_004320005.1
Laticauda laticaudata	GCA_004320025.1
Scolopax mira	GCA_004320125.1
Accipiter nisus	GCA_004320145.1
Dendrocopos noguchii	GCA_004320165.1
Lagopus muta japonica	GCA_004320205.1
Bubo blakistoni	GCA_004320225.1
Megaptera novaeangliae	GCA_004329385.1
Sebastes koreanus	GCA_004335335.1
Sebastes nudus	GCA_004335365.1
Pseudoliparis sp. *Yap Trench*	GCA_004335475.1
Thymallus thymallus	GCA_004348285.1
Ophiodon elongatus	GCA_004358465.1
Grus nigricollis	GCA_004360235.1
Platanista minor	GCA_004363435.1
Eubalaena japonica	GCA_004363455.1
Perognathus longimembris pacificus	GCA_004363475.1
Inia geoffrensis	GCA_004363515.1
Dasyprocta punctata	GCA_004363535.1
Solenodon paradoxus	GCA_004363575.1
Indri indri	GCA_004363605.1

Kogia breviceps	GCA_004363705.1
Ziphius cavirostris	GCA_004364475.1
Cuniculus paca	GCA_004365215.1
Tupaia tana	GCA_004365275.1
Hypophthalmichthys molitrix	GCA_004764525.1
Phataginus tricuspis	GCA_004765945.1
Channa argus	GCA_004786185.1
Pyxicephalus adspersus	GCA_004786255.1
Varanus komodoensis	GCA_004798865.1
Mandrillus sphinx	GCA_004802615.1
Planiliza haematocheilus	GCA_005024645.1
Marmota himalayana	GCA_005280165.1
Prionailurus bengalensis euptilurus	GCA_005406085.2
Macquaria australasica	GCA_005408345.1
Marmota vancouverensis	GCA_005458795.1
Pavo cristatus	GCA_005519975.1
Bagarius yarrelli	GCA_005784505.1
Bos grunniens	GCA_005887515.2
Centrocercus minimus	GCA_005890655.1
Triplophysa siluroides	GCA_006030095.1
Branta canadensis	GCA_006130075.1
Reinhardtius hippoglossoides	GCA_006182925.2
Anser indicus	GCA_006229135.1
Liparis tanakae	GCA_006348945.1
Epinephelus moara	GCA_006386435.1
Cervus albirostris	GCA_006408465.1
Muntiacus crinifrons	GCA_006408485.1
Damaliscus lunatus	GCA_006408505.1
Alcelaphus buselaphus	GCA_006408545.1
Giraffa camelopardalis	GCA_006408565.1
Antidorcas marsupialis	GCA_006408585.1
Connochaetes taurinus	GCA_006408615.1
Nanger granti	GCA_006408635.1
Tragulus kanchil	GCA_006408655.1
Madoqua kirkii	GCA_006408675.1
Aepyceros melampus	GCA_006408695.1
Sylvicapra grimmia	GCA_006408735.1
Eudorcas thomsonii	GCA_006408755.1
Tragelaphus scriptus	GCA_006410495.1

Procapra przewalskii	GCA_006410515.1
Litocranius walleri	GCA_006410535.1
Capra ibex	GCA_006410555.1
Neotragus moschatus	GCA_006410615.1
Cephalophus harveyi	GCA_006410635.1
Kobus ellipsiprymnus	GCA_006410655.1
Oreotragus oreotragus	GCA_006410675.1
Philantomba maxwellii	GCA_006410695.1
Raphicerus campestris	GCA_006410735.1
Tragelaphus eurycerus	GCA_006410755.1
Tragelaphus imberbis	GCA_006410775.1
Tragelaphus strepsiceros	GCA_006410795.1
Neotragus pygmaeus	GCA_006410875.1
Redunca redunca	GCA_006410935.1
Tragelaphus spekii	GCA_006411015.1
Tragelaphus buxtoni	GCA_006411685.1
Tragelaphus oryx	GCA_006416875.1
Ourebia ourebi	GCA_006417275.1
Moschus berezovskii	GCA_006459085.1
Hydropotes inermis	GCA_006459105.1
Moschus chrysogaster	GCA_006461725.1
Erethizon dorsatum	GCA_006547115.1
Anomalopteryx didiformis	GCA_006937325.1
Pachypanchax playfairii	GCA_006937955.1
Callopanchax toddi	GCA_006937965.1
Aphyosemion australe	GCA_006937985.1
Nothobranchius kuhntae	GCA_006942095.1
Hippotragus niger niger	GCA_006942125.1
Neogobius melanostomus	GCA_007210695.1
Zosterops borbonicus	GCA_007252995.1
Alexandromys oeconomus	GCA_007455595.1
Microtus arvalis	GCA_007455615.1
Zosterops pallidus	GCA_007556475.1
Alces alces	GCA_007570765.1
Tachycineta bicolor	GCA_007636935.1
Thylacinus cynocephalus	GCA_007646695.1
Sousa chinensis	GCA_007760645.1
Peromyscus californicus insignis	GCA_007827085.2
Bos frontalis	GCA_007844835.1

Kryptolebias hermaphroditus	GCA_007896545.1
Mesoclemmys tuberculata	GCA_007922155.1
Chelydra serpentina	GCA_007922165.1
Pelusios castaneus	GCA_007922175.1
Carettochelys insculpta	GCA_007922185.1
Podocnemis expansa	GCA_007922195.1
Emydura subglobosa	GCA_007922225.1
Dermatemys mawii	GCA_007922305.1
Pedetes capensis	GCA_007922755.1
Antrozous pallidus	GCA_007922775.1
Nycticeius humeralis	GCA_007922795.1
Macrotus californicus	GCA_007922815.1
Canis lupus	GCA_007922845.1
Coilia nasus	GCA_007927625.1
Pseudorectes ferrugineus	GCA_008033365.1
Thalassoma bifasciatum	GCA_008086565.1
Cheirogaleus medius	GCA_008086735.1
Cebidichthys violaceus	GCA_008087265.1
Oreochromis spilurus	GCA_008269305.1
Lichenostomus cassidix	GCA_008360975.1
Macquaria ambigua	GCA_008360985.1
Triplophysa tibetana	GCA_008369825.1
Oryzias sinensis	GCA_008586565.1
Alca torda	GCA_008658365.1
Colinus virginianus	GCA_008692595.1
Crocuta crocuta	GCA_008692635.1
Cyanoderma ruficeps	GCA_008694505.1
Charadrius alexandrinus	GCA_008711295.1
Mirza zaza	GCA_008750895.1
Microcebus sp. 3 GT-2019	GCA_008750915.1
Microcebus tavaratra	GCA_008750935.1
Microcebus mittermeieri	GCA_008750955.1
Microcebus ravelobensis	GCA_008750975.1
Microcebus griseorufus	GCA_008750995.1
Muntiacus muntjak	GCA_008782695.1
Muntiacus reevesi	GCA_008787405.2
Panthera leo	GCA_008795835.1
Balaenoptera physalus	GCA_008795845.1
Burhinus oedicnemus	GCA_008921705.1

Datnioides undecimradiatus	GCA_008933995.1
Ompok bimaculatus	GCA_009108245.1
Thunnus orientalis	GCA_009176245.1
Sander vitreus	GCA_009193085.1
Cairina moschata domestica	GCA_009194515.1
Spea multiplicata	GCA_009364415.1
Scaphiopus couchii	GCA_009364435.1
Scaphiopus holbrookii	GCA_009364455.1
Spea bombifrons	GCA_009364475.1
Actinemys marmorata	GCA_009430475.1
Anthochaera phrygia	GCA_009430485.1
Bos grunniens x Bos taurus	GCA_009493655.1
Ursus thibetanus thibetanus	GCA_009660055.1
Lophius piscatorius	GCA_009660295.1
Gambusia holbrooki	GCA_009663075.1
Leptobrachium leishanense	GCA_009667805.1
Hemignathus wilsoni	GCA_009690805.1
Naja naja	GCA_009733165.1
Malurus cyaneus samueli	GCA_009741485.1
Chionodraco myersi	GCA_009756435.1
Chionodraco hamatus	GCA_009756495.1
Oreamnos americanus	GCA_009758055.1
Lepus timidus	GCA_009760805.1
Pristis pectinata	GCA_009764475.1
Geothlypis trichas	GCA_009764595.1
Bucorvus abyssinicus	GCA_009769605.1
Cygnus olor	GCA_009769625.1
Eremophila alpestris peregrina	GCA_009792885.1
Cynopterus brachyotis	GCA_009793145.1
Oophaga pumilio	GCA_009801035.1
Tetrax tetrax	GCA_009806455.1
Sialia sialis	GCA_009812075.1
Sterna hirundo	GCA_009819605.1
Sylvia atricapilla	GCA_009819655.1
Phoenicopterus ruber ruber	GCA_009819775.1
Thamnaconus septentrionalis	GCA_009823395.1
Nymphicus hollandicus	GCA_009823435.1
Rhizomys pruinosus	GCA_009823505.1
Aratinga weddellii	GCA_009867205.1

Psittacus erithacus	GCA_009867235.1
Psittacus timneh	GCA_009867315.1
Culter alburnus	GCA_009869775.1
Megalobrama amblycephala	GCA_009869865.1
Anodorhynchus hyacinthinus	GCA_009936445.1
Gymnarchus niloticus	GCA_009936485.1
Ara chloropterus	GCA_010014725.1
Ara ararauna	GCA_010014805.1
Pyrrhura frontalis	GCA_010014865.1
Psitteuteles goldiei	GCA_010014875.1
Lorius garrulus	GCA_010014925.1
Syngnathus floridae	GCA_010014945.1
Pyrrhura griseipectus	GCA_010014965.1
Lophochroa leadbeateri	GCA_010015045.1
Ara militaris	GCA_010015965.1
Spheniscus magellanicus	GCA_010076225.1
Spheniscus humboldti	GCA_010076325.1
Spheniscus demersus	GCA_010077935.1
Pygoscelis antarcticus	GCA_010078415.1
Eudyptes sclateri	GCA_010078445.1
Megadyptes antipodes antipodes	GCA_010078485.1
Eudyptula novaehollandiae	GCA_010078495.1
Eudyptula minor	GCA_010080355.1
Eudyptes schlegeli	GCA_010080425.1
Eudyptula albosignata	GCA_010080465.1
Eudyptes moseleyi	GCA_010082375.1
Eudyptes chrysolophus	GCA_010084205.1
Eudyptes robustus	GCA_010085315.1
Eudyptes pachyrhynchus	GCA_010085335.1
Eudyptes chrysocome	GCA_010085355.1
Eudyptes filholi	GCA_010085365.1
Aptenodytes patagonicus	GCA_010087175.1
Larimichthys polyactis	GCA_010119295.1
Rhombomys opimus	GCA_010120015.1
Poeciliopsis turrubarensis	GCA_010277015.1
Poeciliopsis retropinna	GCA_010277075.1
Bubo bubo	GCA_010303855.1
Buteo japonicus	GCA_010312235.1
Falco tinnunculus	GCA_010332995.1

Otus sunia	GCA_010365825.1
Cervus hanglu yarkandensis	GCA_010411085.1
Protosalanx chinensis	GCA_010882115.1
Poeciliopsis occidentalis	GCA_010883035.1
Limnodynastes dumerilii	GCA_011038615.1
Pterocles orientalis	GCA_011057875.1
Stictonetta naevosa	GCA_011074415.1
Heteronetta atricapilla	GCA_011075105.1
Nettapus auritus	GCA_011076525.1
Melanerpes aurifrons	GCA_011125475.1
Cynomys gunnisoni	GCA_011316645.1
Eupsittula pertinax	GCA_011317185.1
Plectropomus leopardus	GCA_011397275.1
Epinephelus fuscoguttatus	GCA_011397635.1
Clarias macrocephalus	GCA_011419295.1
Poecile atricapillus	GCA_011421415.1
Gymnobelideus leadbeateri	GCA_011680675.1
Aeorestes cinereus	GCA_011751065.1
Pontoporia blainvillei	GCA_011754075.1
Pionus senilis	GCA_011762725.1
Pyrrhura molinae	GCA_011763355.1
Pyrrhura perlata	GCA_011763455.1
Lythrypnus dalli	GCA_011763505.1
Mormyrus iriodes	GCA_011763525.1
Eclectus roratus	GCA_011763535.1
Guaruba guarouba	GCA_011800095.1
Ardeotis arabs	GCA_011801015.1
Dissostichus mawsoni	GCA_011823955.1
Siniperca knerii	GCA_011952075.1
Siniperca chuatsi	GCA_011952085.1
Siniperca scherzeri	GCA_011952095.1
Coreoperca whiteheadi	GCA_011952105.1
Hydrolagus affinis	GCA_012026655.1
Ameiurus melas	GCA_012411365.1
Onychostoma macrolepis	GCA_012432095.1
Nisaetus nipalensis	GCA_012487455.1
Grus monacha	GCA_012487855.1
Antigone vipio	GCA_012488435.1
Martes zibellina	GCA_012583365.1

Ptyas mucosa	GCA_012654045.1
Pantherophis obsoletus	GCA_012654085.1
Trematomus loennbergii	GCA_012861695.1
Capreolus pygargus	GCA_012922965.1
Myiopsitta monachus	GCA_013036005.1
Cromileptes altivelis	GCA_013133815.1
Acipenser oxyrinchus oxyrinchus	GCA_013184475.1
Turdus rufiventris	GCA_013186435.1
Lama glama chaku	GCA_013239585.1
Lama guanicoe cacsilensis	GCA_013239625.1
Vicugna vicugna mensalis	GCA_013265495.1
Verasper variegatus	GCA_013332515.1
Antennarius maculatus	GCA_013358685.1
Menidia beryllina	GCA_013363375.1
Nyctibius grandis	GCA_013368605.1
Fulica atra	GCA_013372525.1
Percina caprodes	GCA_013375575.1
Crypturellus undulatus	GCA_013389825.1
Crypturellus soui	GCA_013389845.1
Piaya cayana	GCA_013389865.1
Geococcyx californianus	GCA_013389885.1
Sula dactylatra	GCA_013389905.1
Aegithalos caudatus	GCA_013389925.1
Fregata magnificens	GCA_013389945.1
Todus mexicanus	GCA_013389965.1
Dryoscopus gambensis	GCA_013389985.1
Calyptomena viridis	GCA_013390005.1
Pitta sordida	GCA_013390025.1
Picathartes gymnocephalus	GCA_013390045.1
Chloropsis hardwickii	GCA_013390065.1
Grus americana	GCA_013390085.1
Tichodroma muraria	GCA_013390105.1
Hydrodamalis gigas	GCA_013391785.1
Menura novaehollandiae	GCA_013396355.1
Cochlearius cochlearius	GCA_013396365.1
Ardeotis kori	GCA_013396375.1
Casuarius casuarius	GCA_013396415.1
Anhinga anhinga	GCA_013396435.1
Lophotis ruficrista	GCA_013396455.1

Smithornis capensis	GCA_013396465.1
Ptilorrhoa leucosticta	GCA_013396485.1
Chunga burmeisteri	GCA_013396505.1
Chaetorhynchus papuensis	GCA_013396525.1
Tachuris rubrigastra	GCA_013396555.1
Rhodinocichla rosea	GCA_013396575.1
Nyctibius bracteatus	GCA_013396595.1
Oceanites oceanicus	GCA_013396615.1
Penelope pileata	GCA_013396635.1
Campylorhamphus procurvoides	GCA_013396655.1
Eubucco bourcierii	GCA_013396675.1
Sakesphorus luctuosus	GCA_013396695.1
Ciccaba nigrolineata	GCA_013396715.1
Grallaria varia	GCA_013396735.1
Sclerurus mexicanus	GCA_013396755.1
Cephalopterus ornatus	GCA_013396775.1
Corythaeola cristata	GCA_013396815.1
Psilopogon haemacephalus	GCA_013396835.1
Hemiprocne comata	GCA_013396855.1
Vireo altiloquus	GCA_013396875.1
Loxia leucoptera	GCA_013396895.1
Stercorarius parasiticus	GCA_013396915.1
Baryphthengus martii	GCA_013396935.1
Regulus satrapa	GCA_013396955.1
Bucco capensis	GCA_013396975.1
Dicaeum eximium	GCA_013396995.1
Myiagra hebetior	GCA_013397015.1
Oceanodroma tethys	GCA_013397025.1
Pachycephala philippinensis	GCA_013397055.1
Rhabdornis inornatus	GCA_013397075.1
Sterrhoptilus dennistouni	GCA_013397095.1
Edolisoma coerulescens	GCA_013397115.1
Pachyramphus minor	GCA_013397135.1
Irena cyanogastra	GCA_013397155.1
Platysteira castanea	GCA_013397175.1
Hylia prasina	GCA_013397195.1
Lanius ludovicianus	GCA_013397235.1
Thryothorus ludovicianus	GCA_013397245.1
Burhinus bistriatus	GCA_013397275.1

Polioptila caerulea	GCA_013397295.1
Donacobius atricapilla	GCA_013397315.1
Leucopsar rothschildi	GCA_013397325.1
Toxostoma redivivum	GCA_013397375.1
Sinosuthora webbiana	GCA_013397395.1
Neopipo cinnamomea	GCA_013397415.1
Erythrocercus mccallii	GCA_013397435.1
Crotophaga sulcirostris	GCA_013397455.1
Furnarius figulus	GCA_013397465.1
Mionectes macconnelli	GCA_013397495.1
Upupa epops	GCA_013397515.1
Pterocles burchelli	GCA_013397535.1
Urocynchramus pylzowi	GCA_013397555.1
Ptilonorhynchus violaceus	GCA_013397565.1
Halcyon senegalensis	GCA_013397595.1
Eolophus roseicapilla	GCA_013397615.1
Columbina picui	GCA_013397635.1
Chroicocephalus maculipennis	GCA_013397655.1
Probosciger aterrimus	GCA_013397665.1
Centropus unirufus	GCA_013397695.1
Calcarius ornatus	GCA_013397715.1
Rhagologus leucostigma	GCA_013397725.1
Cnemophilus loriae	GCA_013397755.1
Eulacestoma nigropectus	GCA_013397775.1
Orthonyx spaldingii	GCA_013397795.1
Aleadryas rufinucha	GCA_013397815.1
Onychorhynchus coronatus	GCA_013397825.1
Melanocharis versteri	GCA_013397845.1
Struthidea cinerea	GCA_013397865.1
Origma solitaria	GCA_013397895.1
Daphoenositta chrysoptera	GCA_013397915.1
Dasyornis broadbenti	GCA_013397935.1
Grantiella picta	GCA_013397955.1
Machaerirhynchus nigripectus	GCA_013397975.1
Malurus elegans	GCA_013397995.1
Oreocharis arfaki	GCA_013398015.1
Panurus biarmicus	GCA_013398035.1
Ifrita kowaldi	GCA_013398055.1
Notiomystis cincta	GCA_013398075.1

Rhynochetos jubatus	GCA_013398095.1
Falcunculus frontatus	GCA_013398115.1
Aegotheles bennettii	GCA_013398125.1
Pedionomus torquatus	GCA_013398155.1
Climacteris rufus	GCA_013398175.1
Caloenas nicobarica	GCA_013398195.1
Melospiza melodia	GCA_013398205.1
Steatornis caripensis	GCA_013398225.1
Promerops cafer	GCA_013398275.1
Chionis minor	GCA_013398295.1
Nothoprocta pentlandii	GCA_013398315.1
Nothoprocta ornata	GCA_013398335.1
Nothocercus nigrocapillus	GCA_013398345.1
Aphelocoma coerulescens	GCA_013398375.1
Pheucticus melanocephalus	GCA_013398405.1
Loxia curvirostra	GCA_013398455.1
Asarcornis scutulata	GCA_013398475.1
Vidua chalybeata	GCA_013398495.1
Rhegmatorhina hoffmannsi	GCA_013398505.1
Agelaius phoeniceus	GCA_013398535.1
Certhia familiaris	GCA_013398575.1
Cercotrichas coryphoeus	GCA_013398595.1
Brachypodius atriceps	GCA_013398615.1
Copsychus sechellarum	GCA_013398635.1
Acrocephalus arundinaceus	GCA_013398685.1
Nesospiza acunhae	GCA_013398715.1
Nothocercus julius	GCA_013398735.1
Sapayoa aenigma	GCA_013398745.1
Eudromia elegans	GCA_013398775.1
Elachura formosa	GCA_013398795.1
Ibidorhyncha struthersii	GCA_013398815.1
Emberiza fucata	GCA_013398835.1
Mohoua ochrocephala	GCA_013398855.1
Prunella himalayana	GCA_013398875.1
Dromas ardeola	GCA_013398915.1
Ceuthmochares aereus	GCA_013398935.1
Callaeas wilsoni	GCA_013398955.1
Catharus fuscescens	GCA_013398975.1
Alaudala cheleensis	GCA_013398995.1

Galbula dea	GCA_013399015.1
Quiscalus mexicanus	GCA_013399035.1
Ramphastos sulfuratus	GCA_013399055.1
Chloroceryle aenea	GCA_013399075.1
Psophia crepitans	GCA_013399095.1
Anseranas semipalmata	GCA_013399115.1
Heliornis fulica	GCA_013399135.1
Odontophorus gujanensis	GCA_013399175.1
Dicrurus megarhynchus	GCA_013399195.1
Spizaetus tyrannus	GCA_013399215.1
Rhipidura dahli	GCA_013399225.1
Ciconia maguari	GCA_013399255.1
Trogon melanurus	GCA_013399275.1
Piprites chloris	GCA_013399295.1
Rostratula benghalensis	GCA_013399305.1
Fregetta grallaria	GCA_013399335.1
Herpetotheres cachinnans	GCA_013399355.1
Alopecoenas beccarii	GCA_013399365.1
Nyctiprogne leucopyga	GCA_013399395.1
Sagittarius serpentarius	GCA_013399415.1
Arenaria interpres	GCA_013399435.1
Anthoscopus minutus	GCA_013399455.1
Chauna torquata	GCA_013399475.1
Corythaixoides concolor	GCA_013399495.1
Sylvietta virens	GCA_013399515.1
Phaetusa simplex	GCA_013399535.1
Circaetus pectoralis	GCA_013399555.1
Podilymbus podiceps	GCA_013399565.1
Glaucidium brasilianum	GCA_013399595.1
Amazona guildingii	GCA_013399615.1
Chordeiles acutipennis	GCA_013399635.1
Setophaga kirtlandii	GCA_013399655.1
Mesembrinibis cayennensis	GCA_013399675.1
Formicarius rufipectus	GCA_013399695.1
Alectura lathami	GCA_013399715.1
Tyrannus savana	GCA_013399735.1
Podargus strigoides	GCA_013399755.1
Semnornis frantzii	GCA_013399775.1
Zosterops hypoxanthus	GCA_013399795.1

Leptocoma aspasia	GCA_013399835.1
Oxyruncus cristatus	GCA_013399855.1
Gymnorhina tibicen	GCA_013399875.1
Serilophus lunatus	GCA_013399895.1
Illadopsis cleaveri	GCA_013399915.1
Cinclus mexicanus	GCA_013399935.1
Ploceus nigricollis	GCA_013399945.1
Indicator maculatus	GCA_013399975.1
Oenanthe oenanthe	GCA_013399995.1
Rynchops niger	GCA_013400035.1
Nycticryphes semicollaris	GCA_013400055.1
Hippolais icterina	GCA_013400075.1
Rhinopomastus cyanomelas	GCA_013400115.1
Rhinoptilus africanus	GCA_013400135.1
Locustella ochotensis	GCA_013400155.1
Phainopepla nitens	GCA_013400175.1
Aramus guarauna	GCA_013400195.1
Cisticola juncidis	GCA_013400215.1
Oriolus oriolus	GCA_013400235.1
Sitta europaea	GCA_013400255.1
Rhadina sibilatrix	GCA_013400275.1
Larus smithsonianus	GCA_013400295.1
Bombycilla garrulus	GCA_013400315.1
Eurystomus gularis	GCA_013400335.1
Certhia brachydactyla	GCA_013400355.1
Thinocorus orbignyianus	GCA_013400375.1
Vidua macroura	GCA_013400395.1
Scytalopus superciliaris	GCA_013400415.1
Pycnonotus jocosus	GCA_013400435.1
Leiothrix lutea	GCA_013400445.1
Tricholaema leucomelas	GCA_013400475.1
Nicator chloris	GCA_013400495.1
Erpornis zantholeuca	GCA_013400515.1
Scopus umbretta	GCA_013400535.1
Jacana jacana	GCA_013400555.1
Peucedramus taeniatus	GCA_013400575.1
Chloropsis cyanopogon	GCA_013400585.1
Syrrhaptes paradoxus	GCA_013400615.1
Hypocryptadius cinnamomeus	GCA_013400635.1

Mystacornis crossleyi	GCA_013400655.1
Oxylabes madagascariensis	GCA_013400675.1
Neodrepanis coruscans	GCA_013400695.1
Prunella fulvescens	GCA_013400715.1
Pomatostomus ruficeps	GCA_013400735.1
Pelecanoides urinatrix	GCA_013400755.1
Chaetops frenatus	GCA_013400775.1
Pluvianellus socialis	GCA_013400795.1
Centropus bengalensis	GCA_013400815.1
Zapornia atra	GCA_013400835.1
Buphagus erythrorhynchus	GCA_013400855.1
Anhinga rufa	GCA_013400875.1
Thalassarche chlororhynchos	GCA_013400895.1
Pardalotus punctatus	GCA_013400915.1
Turnix velox	GCA_013400935.1
Drymodes brunneopygia	GCA_013400955.1
Atrichornis clamosus	GCA_013400975.1
Oreotrochilus melanogaster	GCA_013400995.1
Rissa tridactyla	GCA_013401015.1
Horornis vulcanius	GCA_013401035.1
Uria aalge	GCA_013401055.1
Cepphus grylle	GCA_013401065.1
Passerina amoena	GCA_013401095.1
Calonectris borealis	GCA_013401115.1
Cettia cetti	GCA_013401135.1
Xiphorhynchus elegans	GCA_013401175.1
Pomatorhinus ruficollis	GCA_013401195.1
Atlantisia rogersi	GCA_013401215.1
Pteruthius melanotis	GCA_013401235.1
Urocolius indicus	GCA_013401255.1
Pandion haliaetus	GCA_013401275.1
Glareola pratincola	GCA_013401295.1
Balaeniceps rex	GCA_013401315.1
Brachypteracias leptosomus	GCA_013401335.1
Ceyx cyanopectus	GCA_013401355.1
Spizella passerina	GCA_013401375.1
Coloeus monedula	GCA_013407035.1
Ara glaucogularis	GCA_013433245.1
Amphilophus citrinellus	GCA_013435755.1

Labeo gonius	GCA_013461565.1
Giraffa tippelskirchi	GCA_013496395.1
Clarias magur	GCA_013621035.1
Diceros bicornis minor	GCA_013634535.1
Gasterosteus nipponicus	GCA_014132575.1
Bos gaurus	GCA_014182915.1
Sciaenops ocellatus	GCA_014183145.1
Dicerorhinus sumatrensis harrissoni	GCA_014189135.1
Nibea albiflora	GCA_014281875.1
Elephas maximus	GCA_014332765.1
Aspidoscelis marmoratus	GCA_014337955.1
Cygnus cygnus	GCA_014362685.1
Columba janthina nitens	GCA_014362705.1
Corvus macrorhynchos	GCA_014363025.1
Fratercula cirrhata	GCA_014363165.1
Haliaeetus pelagicus	GCA_014363185.1
Phoebastria albatrus	GCA_014363385.1
Pteropus pselaphon	GCA_014363405.1
Eleutheronema tetradactylum	GCA_014490765.1
Ovis orientalis	GCA_014523465.1
Labeo catla	GCA_014525385.1
Marmota monax	GCA_014533835.1
Cardinalis cardinalis	GCA_014549065.1
Entosphenus tridentatus	GCA_014621495.2
Oryzias celebensis	GCA_014656515.1
Sebastes schlegelii	GCA_014673565.1
Lycocorax pyrrhopterus obiensis	GCA_014706015.1
Silurus glanis	GCA_014706435.1
Oryx dammah	GCA_014754425.2
Lucifuga dentata	GCA_014773175.1
Silurus meridionalis	GCA_014805685.1
Odontesthes bonariensis	GCA_014825785.1
Anableps anableps	GCA_014839685.1
Sylvia borin	GCA_014839755.1
Xenentodon cancila	GCA_014839995.1
Cercopithecus mona	GCA_014849445.1
Bufo gargarizans	GCA_014858855.1
Microtus fortis	GCA_014885135.1
Puma yagouaroundi	GCA_014898765.1

Rangifer tarandus granti	GCA_014898785.1
Kobus leche leche	GCA_014926565.1
Oreochromis tanganicae	GCA_015052225.1
Cyprichromis pavo	GCA_015052265.1
Hemibates koningsi	GCA_015052305.1
Lamprologus callipterus	GCA_015052345.1
Limnochromis abeelei	GCA_015052385.1
Altolamprologus calvus	GCA_015052405.1
Pseudosimochromis curvifrons	GCA_015052425.1
Cyprichromis zonatus	GCA_015052445.1
Xenotilapia sp. 'papilio sunflower'	GCA_015052465.1
Lamprologus ocellatus	GCA_015052485.1
Gnathochromis pfefferi	GCA_015052505.1
Petrochromis sp. *'orthognathus ikola'* AB-2019	GCA_015052525.1
Neolamprologus devosi	GCA_015052545.1
Pseudosimochromis marginatus	GCA_015052565.1
Tropheus sp. *'lunatus'* AB-2019	GCA_015052595.1
Benitochromis conjunctus	GCA_015052645.1
Apistogramma diplotaenia	GCA_015052665.1
Julidochromis sp. *'unterfels'* AB-2019	GCA_015052685.1
Tropheus sp. *'black'*	GCA_015052705.1
Chaenogobius annularis	GCA_015082035.1
Chalinochromis brichardi	GCA_015102055.1
Xenotilapia sima	GCA_015102085.1
Lepidiolamprologus elongatus	GCA_015102115.1
Neolamprologus fasciatus	GCA_015102195.1
Variabilichromis moorii	GCA_015102225.1
Aulonocranus dewindti	GCA_015102295.1
Neolamprologus hecqui	GCA_015102335.1
Neolamprologus nigriventris	GCA_015102355.1
Tilapia brevimanus	GCA_015102415.1
Neolamprologus sexfasciatus	GCA_015102515.1
Neolamprologus modestus	GCA_015102555.1
Julidochromis sp. *'kombe'* AB-2019	GCA_015102605.1
Xenotilapia nasus	GCA_015102635.1
Neolamprologus obscurus	GCA_015102675.1
Lepidiolamprologus kendalli	GCA_015102695.1
Telmatochromis temporalis	GCA_015102735.1

Benthochromis melanoides	GCA_015102775.1
Bathybates graueri	GCA_015102795.1
Benthochromis horii	GCA_015102815.1
Neolamprologus sp. *'kombe'* AB-2019	GCA_015102855.1
Bathybates leo	GCA_015102875.1
Neolamprologus sp. *'kombe'* AB-2019	GCA_015102895.1
Julidochromis sp. *'kombe'* AB-2019	GCA_015102915.1
Neolamprologus mustax	GCA_015102955.1
Telmatochromis sp. *'lufubu'* AB-2019	GCA_015102975.1
Neolamprologus brevis	GCA_015103015.1
Neolamprologus calliurus	GCA_015103035.1
Orthochromis indermauri	GCA_015103075.1
Haplochromis sp. *Chipwa*	GCA_015103095.1
Julidochromis transcriptus	GCA_015103115.1
Haplochromis sp. *Chipwa*	GCA_015103135.1
Petrochromis fasciolatus	GCA_015103155.1
Xenotilapia sp. *'papilio sunflower'*	GCA_015103175.1
Neolamprologus cylindricus	GCA_015103235.1
Paracyprichromis nigripinnis	GCA_015103275.1
Ptychochromis oligocanthus	GCA_015103295.1
Trematochromis benthicola	GCA_015103355.1
Callochromis macrops	GCA_015103395.1
Enantiopus melanogenys	GCA_015103415.1
Cyprichromis sp. *'kibishi'* AB-2019	GCA_015103435.1
Trematocara variabile	GCA_015103455.1
Cyprichromis sp. *'kibishi'* AB-2019	GCA_015103475.1
Ophthalmotilapia heterodonta	GCA_015103535.1
Chalinochromis popelini	GCA_015103555.1
Xenotilapia sp. *'kilesa'* AB-2019	GCA_015103595.1
Xenotilapia sp. *'kilesa'* AB-2019	GCA_015103635.1
Cyathopharynx furcifer	GCA_015103655.1
Ophthalmotilapia nasuta	GCA_015103715.1
Ctenochromis horei	GCA_015103775.1
Xenotilapia spiloptera	GCA_015103795.1
Limnotilapia dardennii	GCA_015103815.1
Petrochromis polyodon	GCA_015103915.1
Lepidiolamprologus attenuatus	GCA_015103955.1
Neolamprologus crassus	GCA_015104075.1
Xenotilapia bathyphilus	GCA_015104175.1

Reganochromis calliurus	GCA_015104255.1
Trematocara nigrifrons	GCA_015104335.1
Trematocara macrostoma	GCA_015104355.1
Bathybates fasciatus	GCA_015104405.1
Neolamprologus christyi	GCA_015104465.1
Limnochromis staneri	GCA_015104515.1
Gnathochromis permaxillaris	GCA_015104535.1
Limnochromis auritus	GCA_015104565.1
Lobochilotes labiatus	GCA_015104595.1
Cyprichromis leptosoma	GCA_015104635.1
Altolamprologus compressiceps	GCA_015104655.1
Julidochromis ornatus	GCA_015104675.1
Neolamprologus pulcher	GCA_015104715.1
Neolamprologus savoryi	GCA_015104745.1
Trematocara marginatum	GCA_015104795.1
Altolamprologus sp. *'compressiceps shell'* AB-2019	GCA_015104815.1
Telmatochromis sp. *'shell'* AB-2019	GCA_015104855.1
Telmatochromis sp. *'shell'* AB-2019	GCA_015104875.1
Altolamprologus sp. *'compressiceps shell'* AB-2019	GCA_015104895.1
Neolamprologus multifasciatus	GCA_015104955.1
Xenotilapia singularis	GCA_015104975.1
Ectodus descampsii	GCA_015104995.1
Julidochromis dickfeldi	GCA_015105015.1
Julidochromis sp. *'regani south'* AB-2019	GCA_015105035.1
Julidochromis sp. *'regani south'* AB-2019	GCA_015105075.1
Lestradea perspicax	GCA_015105095.1
Ophthalmotilapia ventralis	GCA_015105175.1
Perissodus microlepis	GCA_015105195.1
Paracyprichromis sp. *'brieni south'* AB-2019	GCA_015105215.1
Paracyprichromis sp. *'brieni south'* AB-2019	GCA_015105235.1
Haplotaxodon microlepis	GCA_015105255.1
Haplotaxodon trifasciatus	GCA_015105275.1
Neolamprologus caudopunctatus	GCA_015105295.1
Neolamprologus tetracanthus	GCA_015105335.1
Xenotilapia boulengeri	GCA_015105375.1
Interochromis loocki	GCA_015105475.1
Lepidiolamprologus cunningtoni	GCA_015105495.1

Tropheus sp. *'red'* AB-2019	GCA_015105515.1
Tropheus sp. *'red'* AB-2019	GCA_015105585.1
Cyathopharynx foae	GCA_015105635.1
Cyphotilapia gibberosa	GCA_015105655.1
Cyprichromis sp. *'jumbo'* AB-2019	GCA_015105695.1
Plecodus straeleni	GCA_015105735.1
Xenotilapia leptura	GCA_015105755.1
Xenotilapia rotundiventralis	GCA_015105935.1
Xenotilapia flavipinnis	GCA_015105975.1
Perissodus multidentatus	GCA_015106075.1
Xenotilapia nigrolabiata	GCA_015106095.1
Hemibates stenosoma	GCA_015106175.1
Xenotilapia caudafasciata	GCA_015106215.1
Greenwoodochromis bellcrossi	GCA_015106255.1
Trematocara unimaculatum	GCA_015106295.1
Cunningtonia longiventralis	GCA_015106375.1
Lamprologus signatus	GCA_015106455.1
Petrochromis horii	GCA_015106495.1
Neolamprologus prochilus	GCA_015106535.1
Haplochromis vanheusdeni	GCA_015106555.1
Tropheus annectens	GCA_015106575.1
Xenotilapia papilio	GCA_015106585.1
Thoracochromis brauschi	GCA_015106615.1
Sargochromis carlottae	GCA_015106635.1
Coptodon bakossiorum	GCA_015106655.1
Pelmatolapia mariae	GCA_015106675.1
Serranochromis macrocephalus	GCA_015106695.1
Bujurquina vittata	GCA_015106715.1
Paratilapia polleni	GCA_015106735.1
Etia nguti	GCA_015106755.1
Etroplus canarensis	GCA_015106775.1
Paracyprichromis sp. *'tembwe'* AB-2019	GCA_015106795.1
Xenotilapia ochrogenys	GCA_015106855.1
Lestradea stappersii	GCA_015106895.1
Cyprichromis microlepidotus	GCA_015106915.1
Tropheus sp. 'black'	GCA_015106955.1
Neolamprologus mondabu	GCA_015106975.1
Pseudosimochromis babaulti	GCA_015107015.1
Neolamprologus buescheri	GCA_015107095.1

Triglachromis otostigma	GCA_015107115.1
Neolamprologus sp. *'ventralis stripe'* AB-2019	GCA_015107135.1
Neolamprologus sp. *'ventralis stripe'* AB-2019	GCA_015107155.1
Cyprichromis coloratus	GCA_015107215.1
Plecodus paradoxus	GCA_015107295.1
Lamprologus laparogramma	GCA_015107315.1
Neolamprologus meeli	GCA_015107355.1
Lamprologus sp. *'ornatipinnis zambia'* AB-2019	GCA_015107375.1
Lamprologus sp. *'ornatipinnis zambia'* AB-2019	GCA_015107395.1
Bathybates vittatus	GCA_015107455.1
Bathybates hornii	GCA_015107495.1
Grammatotria lemairii	GCA_015107505.1
Boulengerochromis microlepis	GCA_015107555.1
Telmatochromis vittatus	GCA_015107575.1
Bathybates minor	GCA_015107595.1
Telmatochromis brachygnathus	GCA_015107635.1
Xenochromis hecqui	GCA_015107655.1
Tylochromis polylepis	GCA_015107715.1
Baileychromis centropomoides	GCA_015107735.1
Lamprologus lemairii	GCA_015107875.1
Eretmodus cyanostictus	GCA_015107895.1
Greenwoodochromis christyi	GCA_015107935.1
Perissodus eccentricus	GCA_015107955.1
Trematocara stigmaticum	GCA_015107995.1
Julidochromis marlieri	GCA_015108065.1
Petrochromis famula	GCA_015108095.1
Neolamprologus longicaudatus	GCA_015108155.1
Pelvicachromis taeniatus	GCA_015108175.1
Neolamprologus leleupi	GCA_015108215.1
Neolamprologus olivaceous	GCA_015108255.1
Neolamprologus gracilis	GCA_015108275.1
Neolamprologus marunguensis	GCA_015108335.1
Neolamprologus helianthus	GCA_015108395.1
Tilapia sparmanii	GCA_015108415.1
Lamprologus tigripictilis	GCA_015108455.1

Pharyngochromis acuticeps	GCA_015108475.1
Heterochromis multidens	GCA_015108495.1
Hemichromis elongatus	GCA_015108515.1
Steatocranus sp. *'ultraslender'* AB-2019	GCA_015108535.1
Coptodon rendalli	GCA_015108555.1
Heterotilapia buettikoferi	GCA_015108575.1
Amphilophus zaliosus	GCA_015108585.1
Australoheros scitulus	GCA_015108615.1
Andinoacara biseriatus	GCA_015108635.1
Paracyprichromis sp. *'tembwe'* AB-2019	GCA_015108655.1
Telmatochromis sp. *'longola'* AB-2019	GCA_015108675.1
Neolamprologus variostigma	GCA_015108695.1
Lamprologus meleagris	GCA_015108715.1
Telmatochromis sp. *'longola'* AB-2019	GCA_015108735.1
Neolamprologus pectoralis	GCA_015108755.1
Lamprologus sp. 'ornatipinnis congo' AB-2019	GCA_015108795.1
Julidochromis sp. *'unterfels'* AB-2019	GCA_015108815.1
Lamprologus sp. *'ornatipinnis congo'* AB-2019	GCA_015108835.1
Telmatochromis brichardi	GCA_015108855.1
Spathodus erythrodon	GCA_015108895.1
Telmatochromis sp. *'dhonti north'* AB-2019	GCA_015108915.1
Telmatochromis sp. *'dhonti north'* AB-2019	GCA_015108935.1
Neolamprologus furcifer	GCA_015108955.1
Neolamprologus chitamwebwai	GCA_015109035.1
Neolamprologus tretocephalus	GCA_015109125.1
Tropheus duboisi	GCA_015109155.1
Julidochromis regani	GCA_015109195.1
Neolamprologus walteri	GCA_015109215.1
Cyprichromis sp. *'dwarf jumbo'* AB-2019	GCA_015109275.1
Tropheus sp. *'lukuga'* AB-2019	GCA_015109315.1
Cyprichromis sp. *'dwarf jumbo'* AB-2019	GCA_015109335.1
Chalinochromis sp. *'ndobhoi'* AB-2019	GCA_015109345.1
Neolamprologus longior	GCA_015109375.1
Tropheus sp. *'lukuga'* AB-2019	GCA_015109395.1
Chalinochromis sp. *'ndobhoi' A*B-2019	GCA_015109415.1
Tropheus sp. *'lunatus'* AB-2019	GCA_015109455.1
Neolamprologus similis	GCA_015109475.1

Petrochromis sp. *'kazumbae'* AB-2019	GCA_015109515.1
Petrochromis sp. *'kazumbae'* AB-2019	GCA_015109535.1
Cyphotilapia sp. *'5-bar frontosa'* AB-2019	GCA_015109555.1
Orthochromis mazimeroensis	GCA_015109575.1
Neolamprologus sp. *'brevis magara'* AB-2019	GCA_015109595.1
Neolamprologus sp. *'brevis magara'* AB-2019	GCA_015109655.1
Lamprologus speciosus	GCA_015109675.1
Neolamprologus boulengeri	GCA_015109695.1
Cyphotilapia sp. *'5-bar frontosa'* AB-2019	GCA_015109715.1
Neolamprologus ventralis	GCA_015109735.1
Xenotilapia longispinis	GCA_015109775.1
Cardiopharynx schoutedeni	GCA_015109795.1
Callochromis melanostigma	GCA_015109845.1
Astatoreochromis straeleni	GCA_015109875.1
Lepidiolamprologus profundicola	GCA_015109915.1
Lamprologus ornatipinnis	GCA_015109935.1
Neolamprologus pleuromaculatus	GCA_015109975.1
Xenotilapia ornatipinnis	GCA_015110015.1
Callochromis pleurospilus	GCA_015110035.1
Neolamprologus toae	GCA_015110075.1
Spathodus marlieri	GCA_015110135.1
Tanganicodus irsacae	GCA_015110155.1
Tropheus brichardi	GCA_015110215.1
Astatotilapia stappersii	GCA_015110235.1
Ophthalmotilapia sp. *'paranasuta'* AB-2019	GCA_015110275.1
Ophthalmotilapia sp. *'paranasuta'* AB-2019	GCA_015110295.1
Paracyprichromis brieni	GCA_015110335.1
Lamprologus kungweensis	GCA_015110375.1
Petrochromis orthognathus	GCA_015110415.1
Eretmodus marksmithi	GCA_015110455.1
Neolamprologus falcicula	GCA_015110515.1
Tropheus sp. *'brichardi kipili'* AB-2019	GCA_015110675.1
Tropheus sp. *'brichardi kipili'* AB-2019	GCA_015110695.1
Ophthalmotilapia boops	GCA_015110735.1
Julidochromis marksmithi	GCA_015110755.1
Lepidiolamprologus kamambae	GCA_015110795.1
Trematocara zebra	GCA_015110815.1
Neolamprologus sp. *'cygnus'* AB-2019	GCA_015110855.1
Neolamprologus sp. *'cygnus'* AB-2019	GCA_015110875.1

Neolamprologus sp. *'eseki'* AB-2019	GCA_015110895.1
Neolamprologus sp. *'eseki'* AB-2019	GCA_015110915.1
Petrochromis sp. *'orthognathus ikola'* AB-2019	GCA_015110955.1
Cyphotilapia frontosa	GCA_015110975.1
Tropheus polli	GCA_015111015.1
Benthochromis sp. *'horii mahale'* AB-2019	GCA_015111075.1
Xenotilapia sp. *'spilopterus north'* AB-2019	GCA_015111115.1
Xenotilapia sp. *'spilopterus north'* AB-2019	GCA_015111135.1
Tropheus sp. *'mpimbwe'* AB-2019	GCA_015111155.1
Neolamprologus sp. *'caudopunctatus kipili'* AB-2019	GCA_015111175.1
Tropheus sp. *'mpimbwe'* AB-2019	GCA_015111195.1
Neolamprologus sp. *'caudopunctatus kipili'* AB-2019	GCA_015111215.1
Neolamprologus sp. 'furcifer ulwile' AB-2019	GCA_015111235.1
Neolamprologus sp. *'furcifer ulwile'* AB-2019	GCA_015111255.1
Chalinochromis sp. *'bifrenatus'*	GCA_015111275.1
Petrochromis sp. *'kipili brown'* AB-2019	GCA_015111295.1
Petrochromis sp. *'kipili brown'* AB-2019	GCA_015111315.1
Chalinochromis sp. *'bifrenatus'*	GCA_015111335.1
Petrochromis sp. *'giant'* AB-2019	GCA_015111355.1
Lepidiolamprologus mimicus	GCA_015111395.1
Benthochromis tricoti	GCA_015111415.1
Petrochromis macrognathus	GCA_015111435.1
Petrochromis sp. *'moshi yellow'* AB-2019	GCA_015111455.1
Petrochromis sp. *'moshi yellow'* AB-2019	GCA_015111475.1
Tropheus sp. *'Kirschfleck'*	GCA_015111495.1
Tropheus sp. *'Kirschfleck'*	GCA_015111515.1
Petrochromis sp. *'red'* AB-2019	GCA_015111535.1
Petrochromis sp. *'red'* AB-2019	GCA_015111575.1
Bathybates ferox	GCA_015111595.1
Neolamprologus sp. *'gracilis tanzania'* AB-2019	GCA_015111615.1
Neolamprologus sp. *'falcicula mahale'* AB-2019	GCA_015111635.1
Neolamprologus leloupi	GCA_015111655.1
Neolamprologus sp. *'falcicula mahale'* AB-2019	GCA_015111675.1

Neolamprologus sp. *'gracilis tanzania'* AB-2019	GCA_015111695.1
Telmatochromis dhonti	GCA_015111745.1
Julidochromis sp. *'marlieri south'* AB-2019	GCA_015111775.1
Oreochromis malagarasi	GCA_015111835.1
Haplochromis paludinosus	GCA_015111855.1
Julidochromis sp. *'marlieri south'* AB-2019	GCA_015111875.1
Orthochromis uvinzae	GCA_015111895.1
Orthochromis malagaraziensis	GCA_015111915.1
Neolamprologus niger	GCA_015111955.1
Telmatochromis bifrenatus	GCA_015111975.1
Ectodus sp. *'north'*	GCA_015112055.1
Ectodus sp. *'north'*	GCA_015112095.1
Telmatochromis sp. *'dhonti twiyu'* AB-2019	GCA_015112395.1
Trematocara caparti	GCA_015112415.1
Petrochromis sp. *'giant' AB-2019*	GCA_015112455.1
Telmatochromis sp. *'dhonti twiyu'* AB-2019	GCA_015112475.1
Petrochromis sp. *'polyodon texas'* AB-2019	GCA_015112515.1
Petrochromis sp. *'polyodon texas'* AB-2019	GCA_015112535.1
Neolamprologus petricola	GCA_015112555.1
Ophthalmotilapia sp. *'white cap'* AB-2019	GCA_015112595.1
Ophthalmotilapia sp. *'white cap'* AB-2019	GCA_015112615.1
Chalinochromis cyanophleps	GCA_015112635.1
Neolamprologus timidus	GCA_015112655.1
Petrochromis sp. *'macrognathus rainbow'* AB-2019	GCA_015112695.1
Petrochromis sp. *'macrognathus rainbow'* AB-2019	GCA_015112775.1
Astatotilapia flaviijosephi	GCA_015112995.1
Neolamprologus cancellatus	GCA_015113075.1
Tangachromis dhanisi	GCA_015113095.1
Plecodus elaviae	GCA_015113155.1
Tropheus sp. *'murago'* AB-2019	GCA_015113215.1
Lepidiolamprologus sp. *'meeli kipili'* AB-2019	GCA_015113235.1
Neolamprologus bifasciatus	GCA_015113255.1
Tropheus sp. *'murago'* AB-2019	GCA_015113295.1
Lepidiolamprologus sp. *'meeli kipili'* AB-2019	GCA_015113315.1

Xenotilapia tenuidentata	GCA_015118035.1
Dugong dugon	GCA_015147995.1
Pyrrhura lepida	GCA_015163975.1
Mormyrus lacerda	GCA_015163995.1
Brevimyrus niger	GCA_015164015.1
Pogoniulus pusillus	GCA_015220805.1
Hirundo rustica	GCA_015227805.2
Colaptes auratus	GCA_015227895.1
Tenualosa ilisha	GCA_015244755.2
Pao palembangensis	GCA_015343265.1
Laticauda colubrina	GCA_015471245.1
Rousettus leschenaultii	GCA_015472975.1
Fringilla coelebs	GCA_015532645.1
Sarotherodon lohbergeri	GCA_015708405.1
Stomatepia pindu	GCA_015708495.1
Sarotherodon caroli	GCA_015708515.1
Pungu maclareni	GCA_015708565.1
Sarotherodon steinbachi	GCA_015708585.1
Lethenteron reissneri	GCA_015708825.1
Potos flavus	GCA_015708855.1
Procyon lotor	GCA_015708975.1
Argyrosomus japonicus	GCA_015710095.1
Sylvilagus bachmani	GCA_015711505.1
Hydrochoerus hydrochaeris	GCA_015741225.1
Triplophysa dalaica	GCA_015846415.1
Lamprotornis superbus	GCA_015883425.1
Neoceratodus forsteri	GCA_016271365.1
Antechinus flavipes	GCA_016432865.1
Hippotragus equinus	GCA_016433095.1
Gracilinanus agilis	GCA_016433145.1
Prionailurus bengalensis	GCA_016509475.1
Pyrrhura cruentata	GCA_016545565.1
Phylloscopus trochilus trochilus	GCA_016584745.1
Arothron firmamentum	GCA_016586285.1
Platyplectrum ornatum	GCA_016617825.1
Callionymus lyra	GCA_016630915.1
Pavo muticus	GCA_016647715.1
Antechinus stuartii	GCA_016696395.1
Mugilogobius chulae	GCA_016735935.1

Psettodes erumei	GCA_016785225.1
Pseudorhombus dupliciocellatus	GCA_016785235.1
Paraplagusia blochii	GCA_016785265.1
Brachirus orientalis	GCA_016785285.1
Colistium nudipinnis	GCA_016785305.1
Chascanopsetta lugubris	GCA_016785325.1
Trinectes maculatus	GCA_016785345.1
Esox niger	GCA_016801105.1
Novumbra hubbsi	GCA_016801125.1
Umbra pygmaea	GCA_016801145.1
Esox masquinongy	GCA_016801175.1
Dallia pectoralis	GCA_016801195.1
Hystrix brachyura	GCA_016801275.1
Manis crassicaudata	GCA_016801295.1
Caracal caracal	GCA_016801355.1
Sceloporus tristichus	GCA_016801415.1
Polydactylus sextarius	GCA_016801845.1
Toxotes chatareus	GCA_016801885.1
Platichthys stellatus	GCA_016801935.1
Xiphias gladius	GCA_016859285.1
Istiophorus platypterus	GCA_016859345.1
Oryzias woworae	GCA_016861445.1
Phacochoerus africanus	GCA_016906955.1
Gasterosteus aculeatus aculeatus	GCA_016920845.1
Amia calva	GCA_016984155.1
Atractosteus spatula	GCA_016984175.1
Polyodon spathula	GCA_016984195.1
Agelaius tricolor	GCA_017165875.1
Paedocypris sp. *Pulau Singkep*	GCA_017309765.1
Paedocypris sp. *Pulau Singkep*	GCA_017309775.1
Gulo gulo	GCA_900006375.2
Leuciscus waleckii	GCA_900092035.1
Neogale vison	GCA_900108605.1
Eptatretus burgeri	GCA_900186335.2
Saxicola maurus maurus	GCA_900205225.1
Lacerta bilineata	GCA_900245895.1
Lacerta viridis	GCA_900245905.1
Melanogrammus aeglefinus	GCA_900291075.1
Osmerus eperlanus	GCA_900302275.1

Percopsis transmontana	GCA_900302285.1
Parasudis fraserbrunneri	GCA_900302295.1
Polymixia japonica	GCA_900302305.1
Phycis blennoides	GCA_900302315.1
Muraenolepis marmorata	GCA_900302325.1
Phycis phycis	GCA_900302335.1
Melanonus zugmayeri	GCA_900302345.1
Cyttopsis rosea	GCA_900302355.1
Macrourus berglax	GCA_900302365.1
Bathygadus melanobranchus	GCA_900302375.1
Lota lota	GCA_900302385.1
Bregmaceros cantori	GCA_900302395.1
Typhlichthys subterraneus	GCA_900302405.1
Trisopterus minutus	GCA_900302415.1
Brosme brosme	GCA_900302425.1
Boreogadus saida	GCA_900302515.1
Neoniphon sammara	GCA_900302535.1
Lampris guttatus	GCA_900302545.1
Myripristis jacobus	GCA_900302555.1
Gadus chalcogrammus	GCA_900302575.1
Regalecus glesne	GCA_900302585.1
Gadiculus argenteus	GCA_900302595.1
Rondeletia loricata	GCA_900302605.1
Holocentrus rufus	GCA_900302615.1
Thunnus albacares	GCA_900302625.1
Chatrabus melanurus	GCA_900302635.1
Sebastes norvegicus	GCA_900302655.1
Chaenocephalus aceratus	GCA_900302675.1
Spondyliosoma cantharus	GCA_900302685.1
Helostoma temminkii	GCA_900302695.1
Parablennius parvicornis	GCA_900302745.1
Chromis chromis	GCA_900302755.1
Mora moro	GCA_900303205.1
Trachyrincus scabrus	GCA_900303215.1
Laemonema laureysi	GCA_900303225.1
Arctogadus glacialis	GCA_900303235.1
Selene dorsalis	GCA_900303245.1
Lesueurigobius sanzi	GCA_900303255.1
Brotula barbata	GCA_900303265.1

Antennarius striatus	GCA_900303275.1
Rhinella marina	GCA_900303285.1
Merluccius merluccius	GCA_900312545.1
Lamprogrammus exutus	GCA_900312555.1
Beryx splendens	GCA_900312565.1
Acanthochaenus luetkenii	GCA_900312575.1
Malacocephalus occidentalis	GCA_900312585.1
Guentherus altivela	GCA_900312595.1
Stylephorus chordatus	GCA_900312615.1
Merluccius polli	GCA_900312625.1
Pollachius virens	GCA_900312635.1
Carapus acus	GCA_900312935.1
Merluccius capensis	GCA_900312945.1
Myoxocephalus scorpius	GCA_900312955.1
Molva molva	GCA_900323295.1
Trachyrincus murrayi	GCA_900323305.1
Borostomias antarcticus	GCA_900323325.1
Zeus faber	GCA_900323335.1
Pseudochromis fuscus	GCA_900323345.1
Merlangius merlangus	GCA_900323355.1
Monocentris japonicus	GCA_900323365.1
Benthosema glaciale	GCA_900323375.1
Danionella dracula	GCA_900490495.1
Arapaima gigas	GCA_900497675.1
Sardina pilchardus	GCA_900499035.1
Epinephelus coioides	GCA_900536245.1
Trachinotus ovatus	GCA_900607315.1
Hypoplectrus puella	GCA_900610375.1
Arctocephalus gazella	GCA_900642305.1
Diretmus argenteus	GCA_900660295.1
Cetomimus sp. NI1144	GCA_900660305.1
Diretmoides pauciradiatus	GCA_900660315.1
Opsanus beta	GCA_900660325.1
Hoplostethus atlanticus	GCA_900660355.1
Gephyroberyx darwinii	GCA_900660455.1
Lynx pardinus	GCA_900661375.1
Anoplogaster cornuta	GCA_900683385.1
Aeoliscus strigatus	GCA_901007665.1
Fistularia tabacaria	GCA_901007705.1

Dactylopterus volitans	GCA_901007715.1
Hippocampus kuda	GCA_901007745.1
Dunckerocampus dactyliophorus	GCA_901007775.1
Entelurus aequoreus	GCA_901007785.1
Hippocampus whitei	GCA_901007805.1
Macroramphosus scolopax	GCA_901007825.1
Syngnathus rostellatus	GCA_901007895.1
Nerophis ophidion	GCA_901007905.1
Syngnathus typhle	GCA_901007915.1
Anguilla obscura	GCA_901111295.1
Anguilla megastoma	GCA_901111305.1
Anguilla marmorata	GCA_901111315.1
Streptopelia turtur	GCA_901699155.1
Aratinga solstitialis	GCA_902168055.1
Peromyscus nudipes	GCA_902168325.1
Peromyscus aztecus	GCA_902168405.1
Peromyscus melanophrys	GCA_902168415.1
Peromyscus attwateri	GCA_902168425.1
Carcharodon carcharias	GCA_902204185.2
Lutra lutra	GCA_902655055.2
Neostethus bicornis	GCA_902685375.1
Sciurus carolinensis	GCA_902686445.2
Sciurus vulgaris	GCA_902686455.2
Peromyscus eremicus	GCA_902702925.1
Mus minutoides	GCA_902729475.1
Myodes glareolus	GCA_902806735.1
Microtus agrestis	GCA_902806755.1
Alauda arvensis	GCA_902810485.1
Petrochromis trewavasae	GCA_902810495.1
Tropheus moorii	GCA_902810505.1
Coregonus sp. *'balchen'*	GCA_902810595.1
Syncerus caffer	GCA_902825105.1
Harpagifer antarcticus	GCA_902827135.1
Mallotus villosus	GCA_903064625.1
Diplodus sargus	GCA_903131615.1
Mullus surmuletus	GCA_903171835.1
Ovis nivicola lydekkeri	GCA_903231385.1
Serranus cabrilla	GCA_903364295.1
Erithacus rubecula	GCA_903797595.1

Danionella translucida	GCA_903798025.1
Danio albolineatus	GCA_903798035.1
Danio jaintianensis	GCA_903798115.1
Danio choprai	GCA_903798125.1
Danio aesculapii	GCA_903798145.1
Danio kyathit	GCA_903798195.1
Danio tinwini	GCA_903798205.1
Pipistrellus pipistrellus	GCA_903992545.1
Acomys russatus	GCA_903995435.1
Dendrolagus dorianus notatus	GCA_904810745.1
Isoodon auratus barrowensis	GCA_904811015.1
Isoodon macrourus macrourus	GCA_904811065.1
Notamacropus rufogriseus banksianus	GCA_904811085.1
Trichosurus arnhemensis	GCA_904811895.1
Petrogale concinna canescens	GCA_904812245.1
Petrogale xanthopus xanthopus	GCA_904812355.1
Petrogale lateralis hacketti	GCA_904812425.1
Petrogale brachyotis victoriae	GCA_904812665.1
Isoodon obesulus obesulus	GCA_904813085.1
Microperoryctes longicauda magnus	GCA_904814325.1
Nyctereutes procyonoides	GCA_905146905.1
Trachurus trachurus	GCA_905171665.1
Bufo bufo	GCA_905171765.1
Rana temporaria	GCA_905171775.1
Homo sapiens	GCF_000001405.39
Mus musculus	GCF_000001635.27
Loxodonta africana	GCF_000001905.1
Danio rerio	GCF_000002035.6
Monodelphis domestica	GCF_000002295.2
Gallus gallus	GCF_000002315.6
Sus scrofa	GCF_000003025.6
Oryctolagus cuniculus	GCF_000003625.3
Xenopus tropicalis	GCF_000004195.4
Anolis carolinensis	GCF_000090745.1
Meleagris gallopavo	GCF_000146605.3
Myotis lucifugus	GCF_000147115.1
Cavia porcellus	GCF_000151735.1
Pteropus vampyrus	GCF_000151845.1
Dipodomys ordii	GCF_000151885.1

Carlito syrichta	GCF_000164805.1
Vicugna pacos	GCF_000164845.3
Callorhinchus milii	GCF_000165045.1
Microcebus murinus	GCF_000165445.2
Sorex araneus	GCF_000181275.1
Otolemur garnettii	GCF_000181295.1
Felis catus	GCF_000181335.3
Python bivittatus	GCF_000186305.1
Dasypus novemcinctus	GCF_000208655.1
Mustela putorius furo	GCF_000215625.1
Cricetulus griseus	GCF_000223135.1
Latimeria chalumnae	GCF_000225785.1
Pelodiscus sinensis	GCF_000230535.1
Salmo salar	GCF_000233375.1
Ictidomys tridecemlineatus	GCF_000236235.1
Maylandia zebra	GCF_000238955.4
Pundamilia nyererei	GCF_000239375.1
Neolamprologus brichardi	GCF_000239395.1
Haplochromis burtoni	GCF_000239415.1
Chrysemys picta bellii	GCF_000241765.3
Lepisosteus oculatus	GCF_000242695.1
Trichechus manatus latirostris	GCF_000243295.1
Heterocephalus glaber	GCF_000247695.1
Bos indicus	GCF_000247795.1
Ficedula albicollis	GCF_000247815.1
Octodon degus	GCF_000260255.1
Condylura cristata	GCF_000260355.1
Chinchilla lanigera	GCF_000276665.1
Geospiza fortis	GCF_000277835.1
Jaculus jaculus	GCF_000280705.1
Alligator mississippiensis	GCF_000281125.3
Ceratotherium simum simum	GCF_000283155.1
Chrysochloris asiatica	GCF_000296735.1
Erinaceus europaeus	GCF_000296755.1
Orycteropus afer afer	GCF_000298275.1
Bos mutus	GCF_000298355.1
Elephantulus edwardii	GCF_000299155.1
Eptesicus fuscus	GCF_000308155.1
Echinops telfairi	GCF_000313985.2

Microtus ochrogaster	GCF_000317375.1
Odobenus rosmarus divergens	GCF_000321225.1
Pteropus alecto	GCF_000325575.1
Myotis davidii	GCF_000327345.1
Pseudopodoces humilis	GCF_000331425.1
Orcinus orca	GCF_000331955.2
Tupaia chinensis	GCF_000334495.1
Columba livia	GCF_000337935.1
Falco peregrinus	GCF_000337955.1
Falco cherrug	GCF_000337975.1
Mesocricetus auratus	GCF_000349665.1
Leptonychotes weddellii	GCF_000349705.1
Macaca fascicularis	GCF_000364345.1
Astyanax mexicanus	GCF_000372685.2
Zonotrichia albicollis	GCF_000385455.1
Myotis brandtii	GCF_000412655.1
Lipotes vexillifer	GCF_000442215.1
Alligator sinensis	GCF_000455745.1
Panthera tigris altaica	GCF_000464555.1
Poecilia formosa	GCF_000485575.1
Balaenoptera acutorostrata scammoni	GCF_000493695.1
Peromyscus maniculatus bairdii	GCF_000500345.1
Cynoglossus semilaevis	GCF_000523025.1
Nannospalax galili	GCF_000622305.1
Poecilia reticulata	GCF_000633615.1
Egretta garzetta	GCF_000687185.1
Ursus maritimus	GCF_000687225.1
Phaethon lepturus	GCF_000687285.1
Pelecanus crispus	GCF_000687375.1
Cariama cristata	GCF_000690535.1
Colius striatus	GCF_000690715.1
Stegastes partitus	GCF_000690725.1
Eurypyga helias	GCF_000690775.1
Fulmarus glacialis	GCF_000690835.1
Gavia stellata	GCF_000690875.1
Haliaeetus albicilla	GCF_000691405.1
Leptosomus discolor	GCF_000691785.1
Merops nubicus	GCF_000691845.1
Corvus brachyrhynchos	GCF_000691975.1

Opisthocomus hoazin	GCF_000692075.1
Chlamydotis macqueenii	GCF_000695195.1
Mesitornis unicolor	GCF_000695765.1
Acanthisitta chloris	GCF_000695815.1
Galeopterus variegatus	GCF_000696425.1
Equus przewalskii	GCF_000696695.1
Nestor notabilis	GCF_000696875.1
Struthio camelus australis	GCF_000698965.1
Dryobates pubescens	GCF_000699005.1
Pygoscelis adeliae	GCF_000699105.1
Aptenodytes forsteri	GCF_000699145.1
Pterocles gutturalis	GCF_000699245.1
Antrostomus carolinensis	GCF_000700745.1
Apaloderma vittatum	GCF_000703405.1
Tinamus guttatus	GCF_000705375.1
Charadrius vociferus	GCF_000708025.1
Nipponia nippon	GCF_000708225.1
Phalacrocorax carbo	GCF_000708925.1
Cuculus canorus	GCF_000709325.1
Tauraco erythrolophus	GCF_000709365.1
Balearica regulorum gibbericeps	GCF_000709895.1
Buceros rhinoceros silvestris	GCF_000710305.1
Cyprinodon variegatus	GCF_000732505.1
Notothenia coriiceps	GCF_000735185.1
Haliaeetus leucocephalus	GCF_000737465.1
Corvus cornix cornix	GCF_000738735.5
Chaetura pelagica	GCF_000747805.1
Bison bison bison	GCF_000754665.1
Camelus bactrianus	GCF_000767855.1
Boleophthalmus pectinirostris	GCF_000788275.1
Camelus dromedarius	GCF_000803125.2
Nanorana parkeri	GCF_000935625.1
Colobus angolensis palliatus	GCF_000951035.1
Mandrillus leucophaeus	GCF_000951045.1
Cyprinus carpio	GCF_000951615.1
Aotus nancymaae	GCF_000952055.2
Cercocebus atys	GCF_000955945.1
Macaca nemestrina	GCF_000956065.1
Propithecus coquereli	GCF_000956105.1

Anser cygnoides domesticus	GCF_000971095.1
Larimichthys crocea	GCF_000972845.2
Apteryx mantelli mantelli	GCF_001039765.1
Thamnophis sirtalis	GCF_001077635.1
Pantherophis guttatus	GCF_001185365.1
Austrofundulus limnaeus	GCF_001266775.1
Equus asinus	GCF_001305755.1
Calidris pugnax	GCF_001431845.1
Poecilia latipinna	GCF_001443285.1
Poecilia mexicana	GCF_001443325.1
Xiphophorus couchianus	GCF_001444195.1
Sturnus vulgaris	GCF_001447265.1
Gekko japonicus	GCF_001447785.1
Marmota marmota marmota	GCF_001458135.1
Nothobranchius furzeri	GCF_001465895.1
Sinocyclocheilus anshuiensis	GCF_001515605.1
Sinocyclocheilus rhinocerous	GCF_001515625.1
Sinocyclocheilus grahami	GCF_001515645.1
Parus major	GCF_001522545.3
Protobothrops mucrosquamatus	GCF_001527695.2
Coturnix japonica	GCF_001577835.2
Miniopterus natalensis	GCF_001595765.1
Lepidothrix coronata	GCF_001604755.1
Cebus imitator	GCF_001604975.1
Lates calcarifer	GCF_001640805.1
Rhincodon typus	GCF_001642345.1
Kryptolebias marmoratus	GCF_001649575.2
Ictalurus punctatus	GCF_001660625.1
Xenopus laevis	GCF_001663975.1
Rhinopithecus bieti	GCF_001698545.1
Capra hircus	GCF_001704415.1
Manacus vitellinus	GCF_001715985.3
Crocodylus porosus	GCF_001723895.1
Gavialis gangeticus	GCF_001723915.1
Panthera pardus	GCF_001857705.1
Oreochromis niloticus	GCF_001858045.2
Hipposideros armiger	GCF_001890085.1
Hippocampus comes	GCF_001891065.1
Monopterus albus	GCF_001952655.1

Paralichthys olivaceus	GCF_001970005.1
Castor canadensis	GCF_001984765.1
Ailuropoda melanoleuca	GCF_002007445.1
Oncorhynchus kisutch	GCF_002021735.2
Numida meleagris	GCF_002078875.1
Phascolarctos cinereus	GCF_002099425.1
Odocoileus virginianus texanus	GCF_002102435.1
Acanthochromis polyacanthus	GCF_002109545.1
Neomonachus schauinslandi	GCF_002201575.1
Meriones unguiculatus	GCF_002204375.1
Oryzias latipes	GCF_002234675.1
Seriola dumerili	GCF_002260705.1
Bos taurus	GCF_002263795.1
Enhydra lutris	GCF_002288905.1
Delphinapterus leucas	GCF_002288925.2
Ovis aries	GCF_002742125.1
Xiphophorus maculatus	GCF_002775205.1
Amphiprion ocellaris	GCF_002776465.1
Piliocolobus tephrosceles	GCF_002776525.3
Seriola lalandi dorsalis	GCF_002814215.1
Physeter catodon	GCF_002837175.2
Equus caballus	GCF_002863925.1
Paramormyrops kingsleyae	GCF_002872115.1
Oncorhynchus tshawytscha	GCF_002872995.1
Pan troglodytes	GCF_002880755.1
Pongo abelii	GCF_002880775.1
Cyanistes caeruleus	GCF_002901205.1
Salvelinus sp. IW2-2015	GCF_002910315.2
Oryzias melastigma	GCF_002922805.2
Terrapene carolina triunguis	GCF_002925995.2
Desmodus rotundus	GCF_002940915.1
Hyaena hyaena	GCF_003009895.1
Neophocaena asiaeorientalis asiaeorientalis	GCF_003031525.1
Empidonax traillii	GCF_003031625.1
Bubalus bubalis	GCF_003121395.1
Vulpes vulpes	GCF_003160815.1
Theropithecus gelada	GCF_003255815.1
Athene cunicularia	GCF_003259725.1
Callorhinus ursinus	GCF_003265705.1

Puma concolor	GCF_003327715.1
Xiphophorus hellerii	GCF_003331165.1
Macaca mulatta	GCF_003339765.1
Nothoprocta perdicaria	GCF_003342845.1
Dromaius novaehollandiae	GCF_003342905.1
Apteryx rowi	GCF_003343035.1
Carassius auratus	GCF_003368295.1
Bos indicus x Bos taurus	GCF_003369695.1
Urocitellus parryii	GCF_003426925.1
Ursus arctos horribilis	GCF_003584765.1
Chelonoidis abingdonii	GCF_003597395.1
Marmota flaviventris	GCF_003676075.2
Lagenorhynchus obliquidens	GCF_003676395.1
Acinonyx jubatus	GCF_003709585.1
Tachysurus fulvidraco	GCF_003724035.1
Pipra filicauda	GCF_003945595.2
Corapipo altera	GCF_003945725.1
Calypte anna	GCF_003957555.1
Neopelma chrysocephalum	GCF_003984885.1
Strigops habroptila	GCF_004027225.2
Eumetopias jubatus	GCF_004028035.1
Ornithorhynchus anatinus	GCF_004115215.2
Rhinolophus ferrumequinum	GCF_004115265.1
Phyllostomus discolor	GCF_004126475.2
Phasianus colchicus	GCF_004143745.1
Podarcis muralis	GCF_004329235.1
Phoca vitulina	GCF_004348235.1
Perca flavescens	GCF_004354835.1
Anarrhichthys ocellatus	GCF_004355925.1
Peromyscus leucopus	GCF_004664715.2
Grammomys surdaster	GCF_004785775.1
Morone saxatilis	GCF_004916995.1
Monodon monoceros	GCF_005190385.1
Epinephelus lanceolatus	GCF_005281545.1
Lonchura striata domestica	GCF_005870125.1
Oncorhynchus nerka	GCF_006149115.1
Suricata suricatta	GCF_006229205.1
Nomascus leucogenys	GCF_006542625.1
Globicephala melas	GCF_006547405.1

Serinus canaria	GCF_007115625.1
Archocentrus centrarchus	GCF_007364275.1
Gopherus evgoodei	GCF_007399415.2
Lynx canadensis	GCF_007474595.2
Rhinopithecus roxellana	GCF_007565055.1
Gorilla gorilla gorilla	GCF_008122165.1
Sander lucioperca	GCF_008315115.2
Mastomys coucha	GCF_008632895.1
Phocoena sinus	GCF_008692025.1
Etheostoma spectabile	GCF_008692095.1
Papio anubis	GCF_008728515.1
Taeniopygia guttata	GCF_008822105.2
Pangasianodon hypophthalmus	GCF_009078355.1
Corvus moneduloides	GCF_009650955.1
Callithrix jacchus	GCF_009663435.1
Sapajus apella	GCF_009761245.1
Zalophus californianus	GCF_009762305.2
Notolabrus celidotus	GCF_009762535.1
Trachypithecus francoisi	GCF_009764315.1
Dermochelys coriacea	GCF_009764565.2
Thamnophis elegans	GCF_009769535.1
Cyclopterus lumpus	GCF_009769545.1
Lacerta agilis	GCF_009819535.1
Hippoglossus hippoglossus	GCF_009819705.1
Aythya fuligula	GCF_009819795.1
Catharus ustulatus	GCF_009819885.1
Hylobates moloch	GCF_009828535.2
Periophthalmus magnuspinnatus	GCF_009829125.1
Chiroxiphia lanceolata	GCF_009829145.1
Mustela erminea	GCF_009829155.1
Camelus ferus	GCF_009834535.1
Balaenoptera musculus	GCF_009873245.2
Perca fluviatilis	GCF_010015445.1
Lontra canadensis	GCF_010015895.1
Acipenser ruthenus	GCF_010645085.1
Amblyraja radiata	GCF_010909765.1
Petromyzon marinus	GCF_010993605.1
Esox lucius	GCF_011004845.1
Rattus rattus	GCF_011064425.1

Oxyura jamaicensis	GCF_011077185.1
Trichosurus vulpecula	GCF_011100635.1
Fundulus heteroclitus	GCF_011125445.2
Arvicanthis niloticus	GCF_011762505.1
Tursiops truncatus	GCF_011762595.1
Mirounga leonina	GCF_011800145.1
Zootoca vivipara	GCF_011800845.1
Fukomys damarensis	GCF_012274545.1
Melopsittacus undulatus	GCF_012275295.1
Canis lupus dingo	GCF_012295265.1
Halichoerus grypus	GCF_012393455.1
Molothrus ater	GCF_012460135.1
Oncorhynchus keta	GCF_012931545.1
Pan paniscus	GCF_013052645.1
Trachemys scripta elegans	GCF_013100865.1
Etheostoma cragini	GCF_013103735.1
Oncorhynchus mykiss	GCF_013265735.2
Hippoglossus stenolepis	GCF_013339905.1
Scophthalmus maximus	GCF_013347765.1
Anguilla anguilla	GCF_013347855.1
Electrophorus electricus	GCF_013358815.1
Oreochromis aureus	GCF_013358895.1
Megalops cyprinoides	GCF_013368585.1
Cygnus atratus	GCF_013377495.1
Myotis myotis	GCF_014108235.1
Pipistrellus kuhlii	GCF_014108245.1
Molossus molossus	GCF_014108415.1
Rousettus aegyptiacus	GCF_014176215.1
Canis lupus familiaris	GCF_014441545.1
Manis javanica	GCF_014570535.1
Manis pentadactyla	GCF_014570555.1
Ochotona princeps	GCF_014633375.1
Passer montanus	GCF_014805655.1
Sturnira hondurensis	GCF_014824575.1
Artibeus jamaicensis	GCF_014825515.1
Micropterus salmoides	GCF_014851395.1
Talpa occidentalis	GCF_014898055.1
Nematolebias whitei	GCF_014905685.1
Falco rusticolus	GCF_015220075.1

Choloepus didactylus	GCF_015220235.1
Pygocentrus nattereri	GCF_015220715.1
Sebastes umbrosus	GCF_015220745.1
Rattus norvegicus	GCF_015227675.2
Chelonia mydas	GCF_015237465.1
Chlorocebus sabaeus	GCF_015252025.1
Anas platyrhynchos	GCF_015476345.1
Motacilla alba alba	GCF_015832195.1
Tachyglossus aculeatus	GCF_015852505.1
Cyprinodon tularosa	GCF_016077235.1
Mauremys reevesii	GCF_016161935.1
Salvelinus namaycush	GCF_016432855.1
Crotalus tigris	GCF_016545835.1
Saimiri boliviensis boliviensis	GCF_016699345.1
Pimephales promelas	GCF_016745375.1
Polypterus senegalus	GCF_016835505.1
Pogona vitticeps	GCF_900067755.1
Labrus bergylta	GCF_900080235.1
Mus caroli	GCF_900094665.1
Mus pahari	GCF_900095145.1
Astatotilapia calliptera	GCF_900246225.1
Anabas testudineus	GCF_900324465.2
Mastacembelus armatus	GCF_900324485.2
Simochromis diagramma	GCF_900408965.1
Aquila chrysaetos	GCF_900496995.1
Vombatus ursinus	GCF_900497805.2
Notechis scutatus	GCF_900518725.1
Pseudonaja textilis	GCF_900518735.1
Cottoperca gobio	GCF_900634415.1
Parambassis ranga	GCF_900634625.1
Gouania willdenowi	GCF_900634775.1
Betta splendens	GCF_900634795.2
Denticeps clupeoides	GCF_900700375.1
Clupea harengus	GCF_900700415.1
Erpetoichthys calabaricus	GCF_900747795.1
Sparus aurata	GCF_900880675.1
Echeneis naucrates	GCF_900963305.1
Scleropages formosus	GCF_900964775.1
Takifugu rubripes	GCF_901000725.2

Rhinatrema bivittatum	GCF_901001135.1
Salmo trutta	GCF_901001165.1
Syngnathus acus	GCF_901709675.1
Microcaecilia unicolor	GCF_901765095.1
Camarhynchus parvulus	GCF_901933205.1
Salarias fasciatus	GCF_902148845.1
Sphaeramia orbicularis	GCF_902148855.1
Tyto alba alba	GCF_902150015.1
Myripristis murdjan	GCF_902150065.1
Gadus morhua	GCF_902167405.1
Chanos chanos	GCF_902362185.1
Geotrypetes seraphini	GCF_902459505.1
Thalassophryne amazonica	GCF_902500255.1
Pungitius pungitius	GCF_902500615.1
Sarcophilus harrisii	GCF_902635505.1
Scyliorhinus canicula	GCF_902713615.1
Pteropus giganteus	GCF_902729225.1
Pseudochaenichthys georgianus	GCF_902827115.1
Trematomus bernacchii	GCF_902827165.1
Gymnodraco acuticeps	GCF_902827175.1
Arvicola amphibius	GCF_903992535.1
Onychomys torridus	GCF_903995425.1
Colossoma macropomum	GCF_904425465.1
Acanthopagrus latus	GCF_904848185.1
Atriophallophorus winterbourni	GCA_013407085.1
Benedenia humboldti	GCA_016432935.1
Clonorchis sinensis	GCA_000236345.1
Clonorchis sinensis	GCA_003604175.2
Dibothriocephalus latus	GCA_900617775.1
Dicrocoelium dendriticum	GCA_000950715.1
Dipylidium caninum	GCA_017562135.1
Dugesia japonica	GCA_001938525.1
Echinococcus canadensis	GCA_900004735.1
Echinococcus granulosus	GCA_000524195.1
Echinococcus multilocularis	GCA_000469725.3
Echinococcus oligarthrus	GCA_900683695.1
Echinostoma caproni	GCA_900618425.1
Fasciola gigantica	GCA_006461475.1
Fasciola gigantica	GCA_002867515.3

Fasciola hepatica	GCA_900302435.1
Fasciola hepatica	GCA_002763495.2
Fasciola hepatica	GCA_000947175.1
Fasciola hepatica	GCA_000824725.2
Fasciolopsis buski	GCA_008360955.1
Girardia tigrina	GCA_001938485.1
Gyrodactylus bullatarudis	GCA_012064415.1
Gyrodactylus salaris	GCA_000715275.1
Hydatigera taeniaeformis	GCA_900622495.1
Hymenolepis diminuta	GCA_900708905.1
Hymenolepis diminuta	GCA_902177915.1
Hymenolepis diminuta	GCA_900618445.1
Hymenolepis microstoma	GCA_000469805.3
Macrostomum lignano	GCA_002269645.1
Macrostomum lignano	GCA_001188465.1
Mesocestoides corti	GCA_900604375.1
Opisthorchis felineus	GCA_004794785.1
Opisthorchis viverrini	GCA_001990785.1
Opisthorchis viverrini	GCA_000715545.1
Paragonimus heterotremus	GCA_013368495.1
Paragonimus kellicotti	GCA_014220965.1
Paragonimus skrjabini miyazakii	GCA_014338405.1
Paragonimus westermani	GCA_008508345.1
Paragonimus westermani	GCA_015252655.1
Protopolystoma xenopodis	GCA_900617795.1
Rodentolepis nana	GCA_900617975.1
Schistocephalus solidus	GCA_900618435.1
Schistosoma bovis	GCA_003958945.1
Schistosoma curassoni	GCA_900618015.1
Schistosoma haematobium	GCA_000699445.2
Schistosoma japonicum	GCA_006368765.1
Schistosoma japonicum	GCA_000151775.1
Schistosoma mansoni	GCA_000237925.2
Schistosoma margrebowiei	GCA_900618395.1
Schistosoma mattheei	GCA_900617995.1
Schmidtea mediterranea	GCA_002600895.1
Schmidtea mediterranea	GCA_000181075.1
Schmidtea mediterranea	GCA_000691995.1
Schmidtea mediterranea	GCA_000572305.1

Sparganum proliferum	GCA_902702955.1
Spirometra erinaceieuropaei	GCA_902702965.1
Spirometra erinaceieuropaei	GCA_000951995.1
Taenia asiatica	GCA_001693035.2
Taenia asiatica	GCA_900618005.1
Taenia multiceps	GCA_001923025.3
Taenia saginata	GCA_001693075.2
Taenia solium	GCA_001870725.1
Taenia solium	GCA_002221735.1
Taenia solium	GCA_002082475.1
Trichobilharzia regenti	GCA_900618515.1
Caenorhabditis brenneri	GCA_000143925.2
Caenorhabditis japonica	GCA_000147155.1
Heterodera glycines	GCA_000150805.1
Caenorhabditis angaria	GCA_000165025.1
Meloidogyne hapla	GCA_000172435.1
Meloidogyne incognita	GCA_000180415.1
Pristionchus pacificus	GCA_000180635.4
Onchocerca volvulus	GCA_000180695.1
Wuchereria bancrofti	GCA_000180755.1
Caenorhabditis tropicalis	GCA_000186765.1
Ascaris suum	GCA_000187025.3
Heterorhabditis bacteriophora	GCA_000223415.1
Bursaphelenchus xylophilus	GCA_000231135.1
Ascaris suum	GCA_000298755.1
Angiostrongylus cantonensis	GCA_000331205.1
Ascaris suum	GCA_000331935.3
Panagrellus redivivus	GCA_000341325.1
Ancylostoma ceylanicum	GCA_000402015.1
Haemonchus contortus	GCA_000442195.1
Haemonchus contortus	GCA_000469685.2
Onchocerca volvulus	GCA_000499405.2
Elaeophora elaphi	GCA_000499685.1
Steinernema monticolum	GCA_000505645.1
Trichuris muris	GCA_000612645.2
Trichuris trichiura	GCA_000613005.1
Ancylostoma ceylanicum	GCA_000688135.1
Trichuris suis	GCA_000701005.1
Trichuris suis	GCA_000701025.1

Globodera pallida	GCA_000724045.1
Loa loa	GCA_000733445.1
Meloidogyne floridensis	GCA_000751915.1
Steinernema carpocapsae	GCA_000757645.3
Steinernema feltiae	GCA_000757705.1
Steinernema scapterisci	GCA_000757745.1
Steinernema glaseri	GCA_000757755.1
Trichuris suis	GCA_000797535.1
Oesophagostomum dentatum	GCA_000797555.1
Toxocara canis	GCA_000803305.1
Dictyocaulus viviparus	GCA_000816705.1
Ancylostoma duodenale	GCA_000816745.1
Oscheius sp. MCB	GCA_000934875.1
Strongyloides papillosus	GCA_000936265.1
Caenorhabditis elegans	GCA_000939815.1
Parastrongyloides trichosuri	GCA_000941615.1
Rhabditophanes sp. KR3021	GCA_000944355.1
Strongyloides stercoralis	GCA_000947215.1
Onchocerca ochengi	GCA_000950515.2
Caenorhabditis elegans	GCA_000975215.1
Subanguina moxae	GCA_000981365.1
Rotylenchulus reniformis	GCA_001026735.1
Strongyloides venezuelensis	GCA_001028725.1
Romanomermis culicivorax	GCA_001039655.1
Onchocerca ochengi	GCA_001077375.1
Dirofilaria immitis	GCA_001077395.1
Brugia pahangi	GCA_001280985.1
Trichinella murrelli	GCA_001447425.1
Trichinella sp. T6	GCA_001447435.1
Trichinella pseudospiralis	GCA_001447445.1
Trichinella nelsoni	GCA_001447455.1
Trichinella sp. T9	GCA_001447505.1
Trichinella nativa	GCA_001447565.1
Trichinella pseudospiralis	GCA_001447575.1
Trichinella britovi	GCA_001447585.1
Trichinella spiralis	GCA_001447595.1
Trichinella pseudospiralis	GCA_001447645.1
Trichinella patagoniensis	GCA_001447655.1
Trichinella zimbabwensis	GCA_001447665.1

Trichinella pseudospiralis	GCA_001447675.1
Trichinella pseudospiralis	GCA_001447725.1
Trichinella sp. T8	GCA_001447745.1
Trichinella papuae	GCA_001447755.1
Caenorhabditis elegans	GCA_001483305.2
Oscheius sp. TEL-2014	GCA_001513535.1
Wuchereria bancrofti	GCA_001555675.1
Ditylenchus destructor	GCA_001579705.1
Oscheius sp. TEL-2014	GCA_001630785.1
Caenorhabditis nigoni	GCA_001643685.1
Caenorhabditis remanei	GCA_001643735.2
Toxocara canis	GCA_001680135.1
Globodera ellingtonae	GCA_001723225.1
Angiostrongylus cantonensis	GCA_001884285.1
Trichinella nativa	GCA_002148645.1
Diploscapter coronatus	GCA_002207785.1
Trichinella murrelli	GCA_002221485.1
Onchocerca flexuosa	GCA_002249935.1
Parascaris univalens	GCA_002259205.1
Parascaris univalens	GCA_002259215.1
Caenorhabditis remanei	GCA_002259225.1
Caenorhabditis latens	GCA_002259235.1
Diploscapter pachys	GCA_002287525.1
Teladorsagia circumcincta	GCA_002352805.1
Caenorhabditis nigoni	GCA_002742825.1
Meloidogyne graminicola	GCA_002778205.1
Plectus sambesii	GCA_002796945.1
Setaria equina	GCA_003012265.1
Caenorhabditis inopinata	GCA_003052745.1
Meloidogyne arenaria	GCA_003133805.1
Ancylostoma caninum	GCA_003336725.1
Setaria digitata	GCA_003640385.1
Meloidogyne arenaria	GCA_003693565.1
Meloidogyne floridensis	GCA_003693605.1
Meloidogyne javanica	GCA_003693625.1
Meloidogyne incognita	GCA_003693645.1
Meloidogyne enterolobii	GCA_003693675.1
Rhabditida sp. S2_005_001R2	GCA_004026265.1
Bursaphelenchus xylophilus	GCA_004128815.1

Heterodera glycines	GCA_004148225.1
Ditylenchus dipsaci	GCA_004194705.1
Caenorhabditis elegans	GCA_004526295.1
Radopholus similis	GCA_004764675.1
Plectus murrayi	GCA_004785735.1
Wuchereria bancrofti	GCA_005281725.1
Trichinella pseudospiralis	GCA_005323645.1
Strongyloides papillosus	GCA_005656395.1
Baylisascaris schroederi	GCA_006503575.1
Steinernema feltiae	GCA_007213375.1
Haemonchus contortus	GCA_007637855.2
Dirofilaria repens	GCA_008729115.1
Trichinella spiralis	GCA_008807755.1
Trichinella spiralis	GCA_008807775.1
Trichinella spiralis	GCA_008807795.1
Trichinella spiralis	GCA_008807815.1
Halicephalobus mephisto	GCA_009193035.1
Deladenus siricidicola	GCA_009724625.1
Angiostrongylus cantonensis	GCA_009735665.1
Halicephalobus sp. NKZ332	GCA_009761265.1
Dirofilaria immitis	GCA_009829315.1
Caenorhabditis remanei	GCA_010183535.1
Bursaphelenchus xylophilus	GCA_010367655.1
Bursaphelenchus xylophilus	GCA_010367685.1
Trichinella sp. 17WV049-YT159	GCA_011764315.1
Brugia pahangi	GCA_012070555.1
Radopholus similis	GCA_013357305.1
Radopholus similis	GCA_013357325.1
Litomosoides sigmodontis	GCA_013365315.1
Dipetalonema caudispina	GCA_013365325.1
Madathamugadia hiepei	GCA_013365335.1
Dirofilaria immitis	GCA_013365355.1
Cruorifilaria tuberocauda	GCA_013365365.1
Litomosoides brasiliensis	GCA_013365375.1
Caenorhabditis elegans	GCA_013403715.1
Oscheius tipulae	GCA_013425905.1
Ascaris suum	GCA_013433145.1
Steinernema diaprepesi	GCA_013436035.1
Meloidogyne incognita	GCA_014132215.1

Meloidogyne graminicola	GCA_014773135.1
Koerneria luziae	GCA_014805365.1
Levipalatum texanum	GCA_014805385.1
Bunonema sp. RGD898	GCA_014805405.1
Allodiplogaster sudhausi	GCA_014805415.1
Diplogasteroides magnus	GCA_014805445.1
Meloidogyne chitwoodi	GCA_015183025.1
Meloidogyne chitwoodi	GCA_015183035.1
Meloidogyne chitwoodi	GCA_015183065.1
Ascaris lumbricoides	GCA_015227635.1
Heterodera glycines	GCA_015680885.1
Hoplolaimus galeatus	GCA_016432905.1
Hoplolaimus columbus	GCA_016433075.1
Steinernema khuongi	GCA_016648015.1
Caenorhabditis tropicalis	GCA_016735795.1
Caenorhabditis elegans	GCA_016989095.1
Caenorhabditis elegans	GCA_016989105.1
Caenorhabditis elegans	GCA_016989115.1
Caenorhabditis elegans	GCA_016989125.1
Caenorhabditis elegans	GCA_016989145.1
Caenorhabditis elegans	GCA_016989235.1
Caenorhabditis elegans	GCA_016989245.1
Caenorhabditis elegans	GCA_016989275.1
Caenorhabditis elegans	GCA_016989285.1
Caenorhabditis elegans	GCA_016989295.1
Caenorhabditis elegans	GCA_016989365.1
Caenorhabditis elegans	GCA_016989385.1
Caenorhabditis elegans	GCA_016989455.1
Caenorhabditis elegans	GCA_016989505.1
Meloidogyne arenaria	GCA_017562155.1
Meloidogyne javanica	GCA_900003945.1
Meloidogyne arenaria	GCA_900003985.1
Globodera rostochiensis	GCA_900079975.1
Setaria digitata	GCA_900083525.1
Heligmosomoides polygyrus bakeri	GCA_900096555.1
Caenorhabditis elegans	GCA_900160655.1
Meloidogyne incognita	GCA_900182535.1
Oscheius tipulae	GCA_900184235.1
Nippostrongylus brasiliensis	GCA_900200055.1

Pristionchus exspectatus	GCA_900380275.1
Acrobeloides nanus	GCA_900406225.1
Pristionchus arcanus	GCA_900490705.1
Pristionchus maxplancki	GCA_900490775.1
Pristionchus entomophagus	GCA_900490825.1
Pristionchus japonicus	GCA_900490845.1
Pristionchus mayeri	GCA_900490875.1
Pristionchus fissidentatus	GCA_900490895.1
Micoletzkya japonica	GCA_900490955.1
Parapristionchus giblindavisi	GCA_900491355.1
Caenorhabditis sp. 21 LS-2015	GCA_900536235.3
Caenorhabditis panamensis	GCA_900536275.1
Caenorhabditis sp. 26 LS-2015	GCA_900536285.3
Caenorhabditis sp. 31 LS-2015	GCA_900536295.3
Caenorhabditis sp. 40 LS-2015	GCA_900536305.3
Caenorhabditis becei	GCA_900536315.3
Caenorhabditis sp. 32 LS-2015	GCA_900536325.3
Caenorhabditis sp. 39 LS-2015	GCA_900536345.3
Caenorhabditis sp. 38 MB-2015	GCA_900536415.3
Onchocerca ochengi	GCA_900537205.1
Acanthocheilonema viteae	GCA_900537255.1
Litomosoides sigmodontis	GCA_900537275.1
Enterobius vermicularis	GCA_900576705.1
Anisakis simplex	GCA_900576815.1
Haemonchus placei	GCA_900617895.1
Gongylonema pulchrum	GCA_900617915.1
Cylicostephanus goldi	GCA_900617965.1
Anisakis simplex	GCA_900617985.1
Brugia timori	GCA_900618025.1
Onchocerca flexuosa	GCA_900618345.1
Brugia pahangi	GCA_900618355.1
Thelazia callipaeda	GCA_900618365.1
Nippostrongylus brasiliensis	GCA_900618405.1
Soboliphyme baturini	GCA_900618415.1
Heligmosomoides polygyrus	GCA_900618505.1
Wuchereria bancrofti	GCA_900622535.1
Toxocara canis	GCA_900622545.1
Strongylus vulgaris	GCA_900624965.1
Angiostrongylus costaricensis	GCA_900624975.1

Dracunculus medinensis	GCA_900625125.1
Mesorhabditis belari	GCA_900631915.1
Mesorhabditis belari	GCA_900631935.1
Caenorhabditis panamensis	GCA_900883565.2
Panagrolaimus sp. JU765	GCA_901765185.1
Panagrolaimus sp. PS1159	GCA_901765195.1
Panagrolaimus superbus	GCA_901766145.1
Panagrolaimus sp. ES5	GCA_901766855.1
Panagrolaimus davidi	GCA_901779475.1
Panagrolaimus sp. PS1579	GCA_901779485.1
Meloidogyne luci	GCA_902706615.1
Caenorhabditis bovis	GCA_902829315.1
Meloidogyne enterolobii	GCA_903797545.1
Meloidogyne enterolobii	GCA_903994135.1
Bursaphelenchus okinawaensis	GCA_904066225.1
Bursaphelenchus xylophilus	GCA_904066235.1
Bursaphelenchus xylophilus	GCA_904067135.1
Bursaphelenchus okinawaensis	GCA_904067145.1
Caenorhabditis elegans	GCF_000002985.6
Brugia malayi	GCF_000002995.3
Caenorhabditis briggsae	GCF_000004555.1
Caenorhabditis remanei	GCF_000149515.1
Trichinella spiralis	GCF_000181795.1
Loa loa	GCF_000183805.2
Necator americanus	GCF_000507365.1
Strongyloides ratti	GCF_001040885.1
Abscondita terminalis	GCA_013368085.1
Acanthoscurria geniculata	GCA_000661875.1
Acartia tonsa	GCA_900241095.1
Acheta domesticus	GCA_014858955.1
Achipteria coleoptrata	GCA_000988765.1
Acromyrmex charruanus	GCA_017607545.1
Acromyrmex echinatior	GCF_000204515.1
Acromyrmex heyeri	GCA_017607565.1
Acromyrmex insinuator	GCA_017607455.1
Actias luna	GCA_010014465.1
Aculops lycopersici	GCA_015350385.1
Acyrthosiphon pisum	GCF_000142985.2
Acyrthosiphon pisum	GCF_005508785.1

Adela reaumurella	GCA_009867175.1
Adoxophyes honmai	GCA_005406045.1
Adranes taylori	GCA_009867245.1
Aedes aegypti	GCA_001014885.1
Aedes aegypti	GCA_014049715.1
Aedes aegypti	GCF_000004015.4
Aedes aegypti	GCF_002204515.2
Aedes albopictus	GCA_001444175.2
Aedes albopictus	GCA_001574995.1
Aedes albopictus	GCA_006496715.1
Aedes albopictus	GCA_006516635.1
Aedes albopictus	GCF_001876365.2
Aedes albopictus	GCF_006496715.1
Aenictocupidus jacobsonorum	GCA_010014765.1
Aethina tumida	GCF_001937115.1
Agrilus planipennis	GCF_000699045.2
Agrotis ipsilon	GCA_004193855.1
Agrypnia vestita	GCA_016648135.1
Alaus oculatus	GCA_009852465.1
Aleochara bilineata	GCA_003054995.1
Amphibalanus amphitrite	GCA_009805615.1
Amphinemura sulcicollis	GCA_001676325.1
Amphipyra tragopoginis	GCA_905220425.1
Amphipyra tragopoginis	GCA_905220435.1
Amyelois transitella	GCF_001186105.1
Anastatus disparis	GCA_017163975.1
Andricus curvator	GCA_900474205.1
Andricus grossulariae	GCA_011634705.1
Andricus grossulariae	GCA_900474195.1
Andricus inflator	GCA_900474215.1
Andricus quercusramuli	GCA_900474265.1
Androctonus mauritanicus	GCA_011317285.1
Anelosimus studiosus	GCA_008297655.1
Anisolabis maritima	GCA_010014785.1
Anopheles albimanus	GCA_000349125.2
Anopheles albimanus	GCA_014083485.1
Anopheles albimanus	GCA_015501965.1
Anopheles albimanus	GCF_013758885.1
Anopheles aquasalis	GCA_002846955.1

Anopheles arabiensis	GCA_000349185.1
Anopheles arabiensis	GCF_016920715.1
Anopheles atroparvus	GCA_000473505.1
Anopheles atroparvus	GCA_015501955.1
Anopheles bwambae	GCA_007018985.1
Anopheles christyi	GCA_000349165.1
Anopheles coluzzii	GCA_000150765.1
Anopheles coluzzii	GCA_004136515.2
Anopheles coluzzii	GCA_016097095.1
Anopheles coluzzii	GCA_016097175.1
Anopheles coluzzii	GCA_016097185.1
Anopheles coluzzii	GCA_016097195.1
Anopheles coluzzii	GCA_016097205.1
Anopheles coluzzii	GCA_016097225.1
Anopheles coluzzii	GCA_016508615.1
Anopheles coluzzii	GCF_016920705.1
Anopheles cracens	GCA_002091845.1
Anopheles culicifacies	GCA_000473375.1
Anopheles darlingi	GCA_000211455.3
Anopheles dirus	GCA_000349145.1
Anopheles epiroticus	GCA_000349105.1
Anopheles farauti	GCA_000473445.2
Anopheles farauti	GCA_000956265.1
Anopheles farauti No. 4	GCA_000956215.1
Anopheles fontenillei	GCA_008817895.1
Anopheles funestus	GCA_000349085.1
Anopheles funestus	GCA_003951495.1
Anopheles funestus-like sensu Spillings et al. (2009)	GCA_016170035.1
Anopheles gambiae	GCA_000150785.1
Anopheles gambiae	GCA_001014525.1
Anopheles gambiae	GCA_001542645.1
Anopheles gambiae?	GCF_000005575.2
Anopheles koliensis	GCA_000956275.1
Anopheles longipalpis	GCA_016170015.1
Anopheles maculatus	GCA_000473185.1
Anopheles maculatus	GCA_002091835.1
Anopheles melas	GCA_000473525.2
Anopheles merus	GCA_000473845.2

Anopheles merus	GCA_017562075.1
Anopheles minimus	GCA_000349025.1
Anopheles nili	GCA_000439205.1
Anopheles parensis	GCA_016254315.1
Anopheles punctulatus	GCA_000956255.1
Anopheles quadriannulatus	GCA_000349065.1
Anopheles sinensis	GCA_000441895.2
Anopheles sinensis	GCA_000472065.2
Anopheles sp. NFL-2015	GCA_016171315.1
Anopheles stephensi	GCA_000300775.2
Anopheles stephensi	GCA_000349045.1
Anopheles stephensi	GCA_003448955.2
Anopheles stephensi	GCA_003448975.2
Anopheles stephensi	GCA_017562265.1
Anopheles stephensi	GCF_013141755.1
Anopheles vaneedeni	GCA_016170025.1
Anoplophora glabripennis	GCF_000390285.2
Antheraea mylitta	GCA_014332785.1
Antheraea pernyi	GCA_015888305.1
Aphaenogaster ashmeadi	GCA_003063805.1
Aphaenogaster floridana	GCA_003063835.1
Aphaenogaster fulva	GCA_003063765.1
Aphaenogaster miamiana	GCA_003063725.1
Aphaenogaster picea	GCA_003063865.1
Aphaenogaster rudis	GCA_003063745.1
Aphaenogaster rudis	GCA_003063815.1
Aphidius ervi	GCA_011426455.1
Aphidius ervi	GCA_015776835.1
Aphidius gifuensis	GCA_014905175.1
Aphis craccivora	GCA_009835225.1
Aphis glycines	GCA_009761285.1
Aphis glycines	GCA_009928515.1
Aphis gossypii	GCF_004010815.1
Apis cerana	GCF_001442555.1
Apis cerana cerana	GCA_002290385.1
Apis cerana cerana	GCA_011100585.1
Apis cerana japonica	GCA_002217905.1
Apis dorsata	GCA_009792835.1
Apis dorsata	GCF_000469605.1

Apis florea	GCF_000184785.2
Apis florea	GCF_000184785.3
Apis laboriosa	GCA_014066325.1
Apis mellifera	GCF_000002195.4
Apis mellifera	GCF_003254395.2
Apis mellifera carnica	GCA_013841245.1
Apis mellifera caucasica	GCA_013841205.1
Apis mellifera intermissa	GCA_000819425.1
Apis mellifera mellifera	GCA_003314205.1
Apocyclops royi	GCA_900607525.1
Apolygus lucorum	GCA_009739505.2
Apotomis turbidana	GCA_905147195.1
Apotomis turbidana	GCA_905147355.1
Aptinothrips rufus	GCA_902196195.1
Araneus ventricosus	GCA_013235015.1
Arctia plantaginis	GCA_902825445.1
Arctia plantaginis	GCA_902825455.1
Argiope bruennichi	GCA_015342795.1
Aricia agestis	GCA_905147165.1
Aricia agestis	GCA_905147365.1
Armadillidium nasatum	GCA_009176605.1
Armadillidium vulgare	GCA_001887335.1
Armadillidium vulgare	GCA_004104545.1
Asbolus verrucosus	GCA_004193795.1
Asobara japonica	GCA_017141405.1
Atethmia centrago	GCA_905333075.2
Athalia rosae	GCF_000344095.2
Atta cephalotes	GCF_000143395.1
Atta colombica	GCF_001594045.1
Atta texana	GCA_004480015.1
Aulacorthum solani	GCA_008528875.1
Autographa gamma	GCA_905146835.1
Autographa gamma	GCA_905146925.1
Bactrocera dorsalis	GCF_000789215.1
Bactrocera latifrons	GCF_001853355.1
Bactrocera oleae	GCA_001014625.1
Bactrocera oleae	GCF_001188975.1
Bactrocera oleae	GCF_001188975.3
Bactrocera tryoni	GCA_000695345.1

Bactrocera tryoni	GCF_016617805.1
Baetis rhodani	GCA_001676355.1
Belgica antarctica	GCA_000775305.1
Belonocnema kinseyi	GCF_010883055.1
Bemisia tabaci	GCA_003994315.1
Bemisia tabaci	GCA_004919745.1
Bemisia tabaci	GCA_902825415.1
Bemisia tabaci	GCA_902825425.1
Bemisia tabaci	GCA_903994095.1
Bemisia tabaci	GCA_903994105.1
Bemisia tabaci	GCA_903994115.1
Bemisia tabaci	GCA_903994125.1
Bemisia tabaci	GCF_001854935.1
Bicyclus anynana	GCF_900239965.1
Blastobasis lacticolella	GCA_905147135.1
Blastobasis lacticolella	GCA_905147205.1
Blattella germanica	GCA_000762945.2
Blattella germanica	GCA_003018175.1
Bolitophila cinerea	GCA_010015015.1
Boloria selene	GCA_905231865.2
Boloria selene	GCA_905231875.1
Bombus bifarius	GCF_011952205.1
Bombus breviceps	GCA_014825925.1
Bombus confusus	GCA_014737475.1
Bombus consobrinus	GCA_014737455.1
Bombus cullumanus	GCA_014737535.1
Bombus difficillimus	GCA_014737525.1
Bombus haemorrhoidalis	GCA_014825975.1
Bombus ignitus	GCA_014825875.1
Bombus impatiens	GCF_000188095.3
Bombus opulentus	GCA_014737405.1
Bombus picipes	GCA_014737485.1
Bombus polaris	GCA_014737335.1
Bombus pyrosoma	GCA_014825855.1
Bombus sibiricus	GCA_014737505.1
Bombus skorikovi	GCA_014737355.1
Bombus soroeensis	GCA_014737365.1
Bombus superbus	GCA_014737385.1
Bombus terrestris	GCF_000214255.1

Bombus turneri	GCA_014825825.1
Bombus vancouverensis nearcticus	GCF_011952275.1
Bombus vosnesenskii	GCA_011952255.1
Bombus vosnesenskii	GCF_011952255.1
Bombus waltoni	GCA_014737395.1
Bombyx huttoni	GCA_002197625.1
Bombyx mandarina	GCF_003987935.1
Bombyx mori	GCA_000151715.1
Bombyx mori	GCA_000181175.1
Bombyx mori	GCA_000325555.1
Bombyx mori	GCF_000151625.1
Bombyx mori reference	GCF_014905235.1
Bradysia coprophila	GCF_014529535.1
Bradysia odoriphaga	GCA_016920775.1
Brevipalpus yothersi	GCA_003956705.1
Busseola fusca	GCA_007844875.1
Calanus finmarchicus	GCA_002740975.1
Calanus glacialis	GCA_002740985.1
Calephelis nemesis	GCA_002245505.1
Calephelis virginiensis	GCA_002245475.1
Caligus rogercresseyi	GCA_001005125.1
Caligus rogercresseyi	GCA_001005385.1
Caligus rogercresseyi	GCA_013387185.1
Calliphora vicina	GCA_001017275.1
Callosobruchus maculatus	GCA_900659725.1
Calopteryx splendens	GCA_002093875.1
Calycopis cecrops	GCA_001625245.1
Campodea augens	GCA_009757345.1
Campoletis sonorensis	GCA_013761285.1
Camponotus floridanus	GCF_000147175.1
Camponotus floridanus	GCF_003227725.1
Carcinoscorpius rotundicauda	GCA_011833715.1
Carcinoscorpius rotundicauda	GCA_015741065.1
Caridina multidentata	GCA_002091895.1
Carposina sasakii	GCA_014607495.2
Cataglyphis hispanica	GCA_004195275.1
Cataglyphis niger	GCA_004329405.1
Catajapyx aquilonaris	GCA_000934665.2
Catotricha subobsoleta	GCA_011634745.1

Cecidostiba fungosa	GCA_900474305.1
Cecidostiba semifascia	GCA_900474235.1
Cecropterus lyciades	GCA_002930495.1
Celastrina argiolus	GCA_905147145.1
Celastrina argiolus	GCA_905187575.1
Centruroides sculpturatus	GCF_000671375.1
Cephus cinctus	GCF_000341935.1
Ceratina australensis	GCA_004307685.1
Ceratina calcarata	GCF_001652005.1
Ceratitis capitata	GCA_905071925.1
Ceratitis capitata	GCF_000347755.3
Ceratophysella communis	GCA_009869905.1
Ceratosolen solmsi marchali	GCF_000503995.1
Chaoborus trivitattus	GCA_001014815.1
Chelonus insularis	GCF_013357705.1
Cherax destructor	GCA_009830355.1
Cherax quadricarinatus	GCA_009761615.1
Chilo suppressalis	GCA_000636095.1
Chilo suppressalis	GCA_004000445.1
Chionoecetes opilio	GCA_016584305.1
Chironomus riparius	GCA_001014505.1
Chironomus riparius	GCA_902825295.1
Chironomus tentans	GCA_000786525.1
Chrysina resplendens	GCA_010091895.1
Chrysomya rufifacies	GCA_014858655.1
Chrysomya rufifacies	GCA_014858665.1
Chrysomya rufifacies	GCA_014858695.1
Cimex hemipterus	GCA_001663875.1
Cimex lectularius	GCA_001460545.1
Cimex lectularius	GCF_000648675.2
Cinara cedri	GCA_902439185.1
Cirrula hians	GCA_001015075.1
Clitarchus hookeri	GCA_002778355.1
Cloeon dipterum	GCA_902829235.1
Clogmia albipunctata	GCA_001014945.1
Clunio marinus	GCA_900005825.1
Cnaphalocrocis medinalis	GCA_014851415.1
Coboldia fuscipes	GCA_001014335.1
Coccinella septempunctata	GCA_003568925.1

Cochliomyia hominivorax	GCA_004302925.1
Coelopa frigida	GCA_017309665.1
Colias croceus	GCA_009982905.1
Colias croceus	GCA_905220415.1
Colias croceus	GCA_905220445.1
Colletes gigas	GCA_013123115.1
Columbicola columbae	GCA_016920875.1
Condylostylus patibulatus	GCA_001014875.1
Conopomorpha cramerella	GCA_012932125.1
Contarinia nasturtii	GCA_009176525.2
Contarinia nasturtii	GCF_009176525.2
Copidosoma floridanum	GCF_000648655.2
Coptotermes formosanus	GCA_013340265.1
Cordylochernes scorpioides	GCA_003123905.1
Cosmia trapezina	GCA_905163495.1
Cosmia trapezina	GCA_905163595.1
Cotesia vestalis	GCA_000956155.1
Cotesia vestalis	GCA_001675545.1
Craniophora ligustri	GCA_905163465.1
Craniophora ligustri	GCA_905163585.1
Crematogaster levior	GCA_901521435.1
Cryptolaemus montrouzieri	GCA_013387265.1
Cryptotermes secundus	GCF_002891405.2
Ctenocephalides felis	GCF_003426905.1
Culex pipiens pallens	GCF_016801865.1
Culex quinquefasciatus	GCA_015732745.1
Culex quinquefasciatus	GCF_000209185.1
Culex quinquefasciatus	GCF_015732765.1
Culicoides sonorensis	GCA_900002565.1
Culicoides sonorensis	GCA_900258525.2
Cyaniris semiargus	GCA_905147265.1
Cyaniris semiargus	GCA_905187585.1
Cydia pomonella	GCA_003425675.2
Cyphomyrmex costatus	GCF_001594065.1
Dactylopius coccus	GCA_000833685.1
Danaus chrysippus	GCA_004959915.1
Danaus chrysippus	GCA_902713345.1
Danaus chrysippus	GCA_902713385.1
Danaus melanippus	GCA_010014825.1

Danaus plexippus plexippus	GCA_000235995.2
Danaus plexippus plexippus	GCF_009731565.1
Dasypogon diadema	GCA_006980735.1
Deilephila porcellus	GCA_905220455.1
Deilephila porcellus	GCA_905220465.1
Delias pasithoe	GCA_010014985.1
Dendroctonus ponderosae	GCA_000346045.2
Dendroctonus ponderosae	GCF_000355655.1
Dendrolimus punctatus	GCA_012273795.1
Dermacentor silvarum	GCF_013339745.1
Dermanyssus gallinae	GCA_003439945.1
Dermatophagoides farinae	GCA_000767015.1
Dermatophagoides farinae	GCA_002085665.1
Dermatophagoides farinae	GCA_002085665.2
Dermatophagoides pteronyssinus	GCA_003076615.1
Dermatophagoides pteronyssinus	GCF_001901225.1
Diabrotica virgifera	GCF_003013835.1
Diachasma alloeum	GCF_001412515.2
Diadegma semiclausum	GCA_011421695.1
Diadromus collaris	GCA_009394715.1
Diaphorina citri	GCF_000475195.1
Diatraea saccharalis	GCA_014843785.1
Dinoponera quadriceps	GCF_001313825.1
Dinothrombium tinctorium	GCA_003675995.1
Dione vanillae vanillae	GCA_013421085.1
Diptera sp. S2_005_002R2	GCA_004026295.1
Diuraphis noxia	GCA_001465515.1
Diuraphis noxia	GCA_001938505.1
Diuraphis noxia	GCF_001186385.1
Drepana arcuata	GCA_016069955.1
Drosophila albomicans	GCA_000298335.1
Drosophila albomicans	GCA_001014995.1
Drosophila albomicans	GCA_016746445.1
Drosophila albomicans	GCF_009650485.1
Drosophila americana	GCA_001245305.1
Drosophila americana	GCA_001245395.1
Drosophila americana	GCA_900465395.1
Drosophila ananassa	GCF_003285975.2
Drosophila ananassae	GCA_003285975.2

Drosophila ananassae	GCA_017639315.1
Drosophila ananassae	GCF_000005115.1
Drosophila arizonae	GCF_001654025.1
Drosophila asahinai	GCA_008042795.1
Drosophila athabasca	GCA_003185025.1
Drosophila athabasca	GCA_008121215.1
Drosophila athabasca	GCA_008121225.1
Drosophila auraria	GCA_008042615.1
Drosophila azteca	GCA_005876895.1
Drosophila bakoue	GCA_008044335.1
Drosophila biarmipes	GCA_002222855.1
Drosophila biarmipes	GCA_005234255.1
Drosophila biarmipes	GCF_000233415.1
Drosophila bifasciata	GCA_009664405.1
Drosophila bipectinata	GCA_002222825.1
Drosophila bipectinata	GCF_000236285.1
Drosophila birchii	GCA_008042755.1
Drosophila bocki	GCA_008042715.1
Drosophila bunnanda	GCA_008042815.1
Drosophila burlai	GCA_008042655.1
Drosophila busckii	GCA_001014355.1
Drosophila busckii	GCF_001277935.1
Drosophila busckii	GCF_011750605.1
Drosophila elegans	GCA_011057505.1
Drosophila elegans	GCF_000224195.1
Drosophila erecta	GCF_000005135.1
Drosophila erecta	GCF_003286155.1
Drosophila eugracilis	GCF_000236325.1
Drosophila ficusphila	GCF_000220665.1
Drosophila grimshawi	GCF_000005155.2
Drosophila guanche	GCA_900245975.1
Drosophila guanche	GCF_900245975.1
Drosophila gunungcola	GCA_011057485.1
Drosophila hydei	GCA_003285905.2
Drosophila hydei	GCF_002780465.1
Drosophila hydei	GCF_003285905.1
Drosophila innubila	GCA_004354385.1
Drosophila innubila	GCF_004354385.1
Drosophila jambulina	GCA_008042695.1

Drosophila kanapiae	GCA_008042475.1
Drosophila kikkawai	GCF_000224215.1
Drosophila lacertosa	GCA_004143845.1
Drosophila lacertosa	GCA_004143865.1
Drosophila lacteicornis	GCA_008044355.1
Drosophila leontia	GCA_008042735.1
Drosophila lowei	GCA_008121275.1
Drosophila mauritiana	GCA_004382145.1
Drosophila mauritiana	GCF_004382145.1
Drosophila mayri	GCA_008042485.1
Drosophila melanica	GCA_004143765.1
Drosophila melanica	GCA_004143785.1
Drosophila melanogaster	GCA_000705575.1
Drosophila melanogaster	GCA_000778455.1
Drosophila melanogaster	GCA_001014345.1
Drosophila melanogaster	GCA_002050065.1
Drosophila melanogaster	GCA_002300595.1
Drosophila melanogaster	GCA_002310755.1
Drosophila melanogaster	GCA_002310775.1
Drosophila melanogaster	GCA_003397115.2
Drosophila melanogaster	GCA_003401685.1
Drosophila melanogaster	GCA_003401735.1
Drosophila melanogaster	GCA_003401745.1
Drosophila melanogaster	GCA_003401795.1
Drosophila melanogaster	GCA_003401805.1
Drosophila melanogaster	GCA_003401855.1
Drosophila melanogaster	GCA_003401885.1
Drosophila melanogaster	GCA_003401915.1
Drosophila melanogaster	GCA_003401925.1
Drosophila melanogaster	GCA_003401975.1
Drosophila melanogaster	GCA_003402005.1
Drosophila melanogaster	GCA_003402015.1
Drosophila melanogaster	GCA_003402055.1
Drosophila melanogaster	GCA_004798055.1
Drosophila melanogaster	GCA_004798075.1
Drosophila melanogaster	GCA_004798075.2
Drosophila melanogaster	GCA_009869825.1
Drosophila melanogaster	GCA_012273825.1
Drosophila melanogaster	GCA_015832445.1

Drosophila melanogaster	GCA_015852585.1
Drosophila melanogaster	GCF_000001215.4
Drosophila micromelanica	GCA_004143825.1
Drosophila micromelanica	GCA_004143905.1
Drosophila miranda	GCA_001014365.1
Drosophila miranda	GCA_003369915.1
Drosophila miranda	GCF_000269505.1
Drosophila miranda	GCF_003369915.1
Drosophila mojavensis	GCF_000005175.2
Drosophila montana	GCA_003086615.1
Drosophila nasuta	GCA_002222885.1
Drosophila nasuta	GCA_016747895.1
Drosophila nasuta	GCA_017165715.1
Drosophila navojoa	GCF_001654015.1
Drosophila navojoa	GCF_001654015.2
Drosophila neonasuta	GCA_005889595.1
Drosophila nigromelanica	GCA_004149445.1
Drosophila nigromelanica	GCA_004149465.1
Drosophila nikananu	GCA_008042635.1
Drosophila novamexicana	GCA_003285875.3
Drosophila novamexicana	GCA_900465405.1
Drosophila novamexicana	GCF_003285875.2
Drosophila obscura	GCF_002217835.1
Drosophila orena	GCA_005876975.1
Drosophila pectinifera	GCA_008042775.1
Drosophila persimilis	GCF_000005195.2
Drosophila persimilis	GCF_003286085.1
Drosophila pseudoobscura	GCA_001014495.1
Drosophila pseudoobscura	GCA_004329205.1
Drosophila pseudoobscura	GCF_009870125.1
Drosophila pseudoobscura pseudoobscura	GCA_000001765.3
Drosophila pseudoobscura pseudoobscura	GCA_000149495.1
Drosophila pseudoobscura pseudoobscura	GCF_000001765.3
Drosophila punjabiensis	GCA_008042585.1
Drosophila rhopaloa	GCF_000236305.1
Drosophila robusta	GCA_004143805.1
Drosophila robusta	GCA_004143875.1
Drosophila rufa	GCA_008044435.1
Drosophila santomea	GCF_016746245.1

Drosophila sechellia	GCA_000525085.1
Drosophila sechellia	GCA_004382195.1
Drosophila sechellia	GCF_000005215.3
Drosophila sechellia	GCF_004382195.1
Drosophila seguyi	GCA_008042675.1
Drosophila serrata	GCA_008042835.1
Drosophila serrata	GCF_002093755.1
Drosophila simulans	GCA_000180855.1
Drosophila simulans	GCA_000180875.1
Drosophila simulans	GCA_000180915.1
Drosophila simulans	GCA_000180935.1
Drosophila simulans	GCA_000180955.1
Drosophila simulans	GCA_000180975.1
Drosophila simulans	GCA_000259045.1
Drosophila simulans	GCA_000525065.1
Drosophila simulans	GCA_000820565.1
Drosophila simulans	GCA_004382185.1
Drosophila simulans	GCF_000259055.1
Drosophila simulans	GCF_000754195.2
Drosophila simulans	GCF_016746395.1
Drosophila subobscura	GCA_002749795.1
Drosophila subobscura	GCA_900682585.1
Drosophila subobscura	GCA_903684685.1
Drosophila subobscura	GCF_008121235.1
Drosophila subpulchrella	GCF_014743375.2
Drosophila suzukii	GCA_000326985.1
Drosophila suzukii	GCA_013340185.1
Drosophila suzukii	GCF_000472105.1
Drosophila suzukii	GCF_013340165.1
Drosophila takahashii	GCA_002223445.1
Drosophila takahashii	GCF_000224235.1
Drosophila tani	GCA_008042535.1
Drosophila teissieri	GCA_016746235.1
Drosophila triauraria	GCA_008044375.1
Drosophila triauraria	GCA_014170255.2
Drosophila truncata	GCA_008042515.1
Drosophila virilis	GCA_000004125.1
Drosophila virilis	GCA_003285735.2
Drosophila virilis	GCA_007989325.2

Drosophila virilis	GCA_016920725.1
Drosophila virilis	GCF_000005245.1
Drosophila virilis	GCF_003285735.1
Drosophila vulcana	GCA_008042555.1
Drosophila watanabei	GCA_008042575.1
Drosophila willistoni	GCF_000005925.1
Drosophila yakuba	GCA_016746335.1
Drosophila yakuba	GCF_000005975.2
Drosophila yakuba	GCF_016746365.1
Druon quercuslanigerum	GCA_010014905.1
Dryococelus australis	GCA_002236955.1
Dufourea novaeangliae	GCF_001272555.1
Dysdera silvatica	GCA_003076615.2
Dysdera silvatica	GCA_006491805.1
Ectropis grisescens	GCA_017562165.1
Elaeidobius kamerunicus	GCA_014849505.1
Elymnias hypermnestra	GCA_015832385.1
Endotricha flammealis	GCA_905160925.1
Endotricha flammealis	GCA_905163395.1
Ennomos fuscantarius	GCA_905220475.1
Ennomos fuscantarius	GCA_905220485.1
Epargyreus clarus	GCA_014595695.1
Epargyreus clarus clarus	GCA_014595695.2
Ephemera danica	GCA_000507165.2
Ephydra gracilis	GCA_001014675.1
Ericerus pela	GCA_011428145.1
Ericerus pela	GCA_016591455.1
Eriocheir sinensis	GCA_003336515.1
Eriocheir sinensis	GCA_013436485.1
Eriosoma lanigerum	GCA_013282895.1
Eristalis dimidiata	GCA_001015145.1
Eristalis tenax	GCA_905231845.1
Eristalis tenax	GCA_905231855.1
Erynnis tages	GCA_905147235.1
Erynnis tages	GCA_905147245.1
Eufriesea mexicana	GCF_001483705.1
Euglossa dilemma	GCA_002201625.1
Eumaeus atala	GCA_017140195.1
Eumeta japonica	GCA_005406025.1

Eupelmus annulatus	GCA_900480025.1
Eupelmus urozonus	GCA_900480035.1
Euproctis similis	GCA_905147215.1
Euproctis similis	GCA_905147225.1
Euroglyphus maynei	GCA_002135145.1
Eurytemora affinis	GCF_000591075.1
Eurytoma adleriae	GCA_900480045.1
Eurytoma brunniventris	GCA_900475205.1
Euschistus heros	GCA_003667255.1
Eutreta diana	GCA_001015115.1
Ferrisia virgata	GCA_900060175.1
Folsomia candida	GCF_002217175.1
Fopius arisanus	GCF_000806365.1
Formica exsecta	GCF_003651465.1
Formica selysi	GCA_009859135.1
Frankliniella occidentalis	GCF_000697945.2
Frieseomelitta varia	GCA_011392965.1
Galendromus occidentalis	GCF_000255335.1
Galleria mellonella	GCA_002589825.1
Galleria mellonella	GCA_004355975.1
Galleria mellonella	GCF_003640425.1
Galleria mellonella	GCF_003640425.2
Gammarus roeselii	GCA_016164225.1
Ganaspis brasiliensis	GCA_009823575.1
Ganaspis sp. Gsp50	GCA_011057455.1
Gerris buenoi	GCA_001010745.2
Glossina austeni	GCA_000688735.1
Glossina brevipalpis	GCA_000671755.1
Glossina fuscipes	GCF_014805625.1
Glossina fuscipes fuscipes	GCA_000671735.1
Glossina morsitans	GCA_001014515.1
Glossina morsitans morsitans	GCA_001077435.1
Glossina pallidipes	GCA_000688715.1
Glossina palpalis gambiensis	GCA_000818775.1
Glossosoma conforme	GCA_003347265.1
Goniozus legneri	GCA_003055095.1
Graphium doson	GCA_014048405.1
Gryllus bimaculatus	GCA_017312745.1
Habropoda laboriosa	GCF_001263275.1

Haemaphysalis longicornis	GCA_008122185.1
Haemaphysalis longicornis	GCA_013339765.1
Haematobia irritans	GCA_003123925.1
Halyomorpha halys	GCF_000696795.2
Harmonia axyridis	GCA_003402655.1
Harmonia axyridis	GCA_003865275.1
Harmonia axyridis	GCA_003865295.1
Harmonia axyridis	GCA_003865315.1
Harmonia axyridis	GCA_009805905.1
Harmonia axyridis	GCA_011033045.1
Harpegnathos saltator	GCF_000147195.1
Harpegnathos saltator	GCF_003227715.1
Heliconius aoede	GCA_900068225.1
Heliconius cydno alithea	GCA_900068235.1
Heliconius cydno alithea	GCA_900068245.1
Heliconius cydno alithea	GCA_900068255.1
Heliconius cydno chioneus	GCA_900068035.1
Heliconius cydno chioneus	GCA_900068085.1
Heliconius cydno chioneus	GCA_900068115.1
Heliconius cydno chioneus	GCA_900068205.1
Heliconius cydno cordula	GCA_001485705.1
Heliconius cydno cordula	GCA_001485745.1
Heliconius cydno cordula	GCA_001485765.1
Heliconius cydno cordula	GCA_001485865.1
Heliconius cydno cordula	GCA_900068265.1
Heliconius cydno cydnides	GCA_900068275.1
Heliconius cydno cydnides	GCA_900068285.1
Heliconius cydno hermogenes	GCA_001510915.1
Heliconius cydno weymeri	GCA_900068295.1
Heliconius cydno weymeri	GCA_900068305.1
Heliconius cydno zelinde	GCA_900068315.1
Heliconius elevatus	GCA_001486085.1
Heliconius elevatus	GCA_900067965.1
Heliconius elevatus	GCA_900067985.1
Heliconius elevatus	GCA_900068385.1
Heliconius elevatus	GCA_900068395.1
Heliconius elevatus	GCA_900068405.1
Heliconius elevatus bari	GCA_900068355.1
Heliconius elevatus bari	GCA_900068365.1

Heliconius elevatus bari	GCA_900068375.1
Heliconius erato x Heliconius himera	GCA_013421005.1
Heliconius ethilla	GCA_001485825.1
Heliconius ethilla	GCA_001485845.1
Heliconius ethilla	GCA_001485905.1
Heliconius ethilla aerotome	GCA_001485785.1
Heliconius ethilla aerotome	GCA_001485985.1
Heliconius hecale	GCA_900068415.1
Heliconius hecale	GCA_900068425.1
Heliconius hecale	GCA_900068435.1
Heliconius hecale	GCA_900068445.1
Heliconius hecale felix	GCA_001486065.1
Heliconius hecale felix	GCA_001486125.1
Heliconius hecuba flava	GCA_900068455.1
Heliconius hermathena	GCA_013403705.1
Heliconius heurippa	GCA_900067975.1
Heliconius heurippa	GCA_900068465.1
Heliconius hierax	GCA_900068475.1
Heliconius himera	GCA_013421025.1
Heliconius himera	GCA_013421065.1
Heliconius ismenius	GCA_001485965.1
Heliconius ismenius metaphorus	GCA_001485725.1
Heliconius melpomene aglaope	GCA_001485885.1
Heliconius melpomene aglaope	GCA_001485925.1
Heliconius melpomene aglaope	GCA_001486005.1
Heliconius melpomene aglaope	GCA_001486045.1
Heliconius melpomene aglaope	GCA_001486105.1
Heliconius melpomene aglaope	GCA_900068055.1
Heliconius melpomene aglaope	GCA_900068105.1
Heliconius melpomene aglaope	GCA_900068165.1
Heliconius melpomene amandus	GCA_900068485.1
Heliconius melpomene amandus	GCA_900068495.1
Heliconius melpomene amaryllis	GCA_001485805.1
Heliconius melpomene amaryllis	GCA_001485945.1
Heliconius melpomene amaryllis	GCA_001486025.1
Heliconius melpomene amaryllis	GCA_001486145.1
Heliconius melpomene amaryllis	GCA_900068075.1
Heliconius melpomene amaryllis	GCA_900068175.1
Heliconius melpomene amaryllis	GCA_900068195.1

Heliconius melpomene amaryllis	GCA_900068505.1
Heliconius melpomene bellula	GCA_900068535.1
Heliconius melpomene bellula	GCA_900068545.1
Heliconius melpomene cythera	GCA_900068515.1
Heliconius melpomene cythera	GCA_900068525.1
Heliconius melpomene ecuadorensis	GCA_900068555.1
Heliconius melpomene ecuadorensis	GCA_900068565.1
Heliconius melpomene malleti	GCA_001510935.1
Heliconius melpomene malleti	GCA_900068585.1
Heliconius melpomene malleti x *Heliconius melpomene plesseni*	GCA_900068575.1
Heliconius melpomene melpomene	GCA_000313835.2
Heliconius melpomene melpomene	GCA_000326025.1
Heliconius melpomene melpomene	GCA_013420985.1
Heliconius melpomene melpomene	GCA_900067995.1
Heliconius melpomene melpomene	GCA_900068025.1
Heliconius melpomene melpomene	GCA_900068045.1
Heliconius melpomene melpomene	GCA_900068135.1
Heliconius melpomene melpomene	GCA_900068145.1
Heliconius melpomene melpomene	GCA_900068185.1
Heliconius melpomene melpomene	GCA_900068595.1
Heliconius melpomene melpomene	GCA_900068605.1
Heliconius melpomene melpomene	GCA_900068615.1
Heliconius melpomene meriana	GCA_900068625.1
Heliconius melpomene meriana	GCA_900068635.1
Heliconius melpomene plesseni	GCA_900068645.1
Heliconius melpomene plesseni	GCA_900068655.1
Heliconius melpomene rosina	GCA_900067955.1
Heliconius melpomene rosina	GCA_900068065.1
Heliconius melpomene rosina	GCA_900068155.1
Heliconius melpomene rosina	GCA_900068215.1
Heliconius melpomene rosina	GCA_900068665.1
Heliconius melpomene thelxiopeia	GCA_900068675.1
Heliconius melpomene vulcanus	GCA_900068685.1
Heliconius melpomene vulcanus	GCA_900068695.1
Heliconius nattereri	GCA_013403645.1
Heliconius numata	GCA_016802465.1
Heliconius numata	GCA_016802485.1
Heliconius numata	GCA_016802505.1

Heliconius numata	GCA_016802525.1
Heliconius numata	GCA_016802545.1
Heliconius numata	GCA_016802565.1
Heliconius numata	GCA_016802585.1
Heliconius numata	GCA_016802605.1
Heliconius numata	GCA_016802625.1
Heliconius numata	GCA_016802665.1
Heliconius numata	GCA_016802685.1
Heliconius numata	GCA_016802705.1
Heliconius numata arcuella	GCA_001486725.1
Heliconius numata bicoloratus	GCA_900068705.1
Heliconius numata bicoloratus	GCA_900068715.1
Heliconius numata silvana	GCA_001486245.1
Heliconius numata tarapotensis	GCA_001486745.1
Heliconius pachinus	GCA_900068725.1
Heliconius pachinus	GCA_900068735.1
Heliconius pardalinus	GCA_900068005.1
Heliconius pardalinus sergestus	GCA_001486225.1
Heliconius pardalinus sergestus	GCA_001486285.1
Heliconius pardalinus sergestus	GCA_001486325.1
Heliconius pardalinus sergestus	GCA_001486625.1
Heliconius pardalinus sergestus	GCA_001486765.1
Heliconius pardalinus ssp. JD-2011	GCA_001486605.1
Heliconius pardalinus ssp. n. KMK-2015	GCA_900068745.1
Heliconius pardalinus ssp. n. KMK-2015	GCA_900068755.1
Heliconius pardalinus ssp. n. KMK-2015	GCA_900068765.1
Heliconius timareta	GCA_001486305.1
Heliconius timareta	GCA_001486405.1
Heliconius timareta	GCA_001486425.1
Heliconius timareta	GCA_001486705.1
Heliconius timareta	GCA_900068015.1
Heliconius timareta	GCA_900068095.1
Heliconius timareta	GCA_900068125.1
Heliconius timareta ssp. JD-2011	GCA_001486185.1
Heliconius timareta ssp. JD-2011	GCA_001486345.1
Heliconius timareta ssp. JD-2011	GCA_001486545.1
Heliconius timareta ssp. JD-2011	GCA_001486685.1
Heliconius timareta ssp. NG-2008	GCA_001486265.1
Heliconius timareta ssp. NG-2008	GCA_001486485.1

Heliconius timareta ssp. NG-2008	GCA_001486565.1
Heliconius timareta ssp. NG-2008	GCA_001486645.1
Heliconius timareta timareta	GCA_001486205.1
Heliconius timareta timareta	GCA_001486365.1
Heliconius timareta timareta	GCA_001486385.1
Heliconius timareta timareta	GCA_001486465.1
Heliconius timareta timareta	GCA_900068775.1
Heliconius timareta timareta	GCA_900068785.1
Heliconius timareta timareta	GCA_900068795.1
Heliconius timareta timareta	GCA_900068805.1
Heliconius wallacei	GCA_900068815.1
Heliconius xanthocles	GCA_900068825.1
Helicorthomorpha holstii	GCA_013389785.1
Helicoverpa armigera	GCA_017165865.1
Helicoverpa armigera	GCF_002156985.1
Helicoverpa zea	GCA_002150865.1
Heliothis virescens	GCA_002382865.1
Hermetia illucens	GCA_001014895.1
Hermetia illucens	GCA_009835165.1
Hermetia illucens	GCF_905115235.1
Hesperophylax magnus	GCA_016648045.1
Hestina assimilis	GCA_016906905.1
Hexapoda sp.	GCA_002916965.1
Holacanthella duospinosa	GCA_002738285.1
Holcocephala fusca	GCA_001015215.1
Homalodisca vitripennis	GCA_000696855.2
Hormaphis cornu	GCA_017140985.1
Hyalella azteca	GCF_000764305.1
Hyalomma asiaticum	GCA_013339685.1
Hycleus cichorii	GCA_013841215.1
Hycleus phaleratus	GCA_013841185.1
Hydropsyche tenuis	GCA_009617725.1
Hylaea fasciaria	GCA_905147295.1
Hylaea fasciaria	GCA_905147375.1
Hyles vespertilio	GCA_009982885.1
Hypena proboscidalis	GCA_905147285.1
Hypena proboscidalis	GCA_905147305.1
Hyphantria cunea	GCA_003709505.1
Hypochthonius rufulus	GCA_000988845.1

Hypolimnas misippus	GCA_008963455.1
Hyposmocoma kahamanoa	GCF_003589595.1
Hypothenemus hampei	GCA_001012855.1
Hypothenemus hampei	GCA_013372445.1
Ignelater luminosus	GCA_011009095.1
Ips typographus	GCA_016097725.1
Isoperla grammatica	GCA_001676475.1
Ixodes persulcatus	GCA_013358835.1
Ixodes ricinus	GCA_000973045.2
Ixodes scapularis	GCA_002892825.2
Ixodes scapularis	GCF_000208615.1
Ixodes scapularis	GCF_002892825.2
Ixodes scapularis	GCF_016920785.1
Ladona fulva	GCA_000376725.2
Lamprigera yunnana	GCA_013368075.1
Laodelphax striatellus	GCA_003335185.2
Laodelphax striatellus	GCA_014465815.1
Laodelphax striatellus	GCA_017141395.1
Laothoe populi	GCA_905220495.1
Laothoe populi	GCA_905220505.1
Laparus doris	GCA_900068325.1
Laparus doris delila	GCA_900068345.1
Laparus doris doris	GCA_900068335.1
Lasioglossum albipes	GCA_000346575.1
Lasius niger	GCA_001045655.1
Laspeyria flexula	GCA_905147015.1
Laspeyria flexula	GCA_905147035.1
Latrodectus hesperus	GCA_000697925.2
Laupala kohalensis	GCA_002313205.1
Lednia tumana	GCA_003287335.1
Lepeophtheirus salmonis	GCA_000181255.2
Lepeophtheirus salmonis	GCA_001005205.1
Lepeophtheirus salmonis	GCA_001005235.1
Lepidotrigona ventralis hoosana	GCA_002806875.1
Leptidea sinapis	GCA_900199415.1
Leptidea sinapis	GCA_900199415.2
Leptidea sinapis	GCA_900199445.1
Leptinotarsa decemlineata	GCF_000500325.1
Leptinotarsa defecta	GCA_015342085.1

Leptinotarsa haldemani	GCA_015342125.1
Leptinotarsa juncta	GCA_015342165.1
Leptinotarsa juncta	GCA_015342185.1
Leptinotarsa lineolata	GCA_015342045.1
Leptinotarsa peninsularis	GCA_015342205.1
Leptinotarsa rubiginosa	GCA_015342145.1
Leptinotarsa texana	GCA_015342105.1
Leptinotarsa tumamoca	GCA_015342025.1
Leptinotarsa undecemlineata	GCA_015342065.1
Leptopilina boulardi	GCA_003121605.1
Leptopilina boulardi	GCA_011634795.1
Leptopilina boulardi	GCA_015476485.1
Leptopilina clavipes	GCA_001855655.1
Leptopilina heterotoma	GCA_009025955.1
Leptopilina heterotoma	GCA_009026005.1
Leptopilina heterotoma	GCA_009602685.1
Leptopilina heterotoma	GCA_010016045.1
Leptopilina heterotoma	GCA_015476425.1
Leptotrombidium deliense	GCA_003675905.1
Leptotrombidium deliense	GCA_003675905.2
Leptotrombidium pallidum	GCA_013372475.1
Lerema accius	GCA_001278395.1
Ligia exotica	GCA_002091915.1
Limenitis camilla	GCA_905147315.1
Limenitis camilla	GCA_905147385.1
Limnephilus lunatus	GCA_000648945.2
Limonius californicus	GCA_014611495.1
Limulus polyphemus	GCF_000517525.1
Linepithema humile	GCF_000217595.1
Lipothrix lubbocki	GCA_009872335.1
Liriomyza trifolii	GCA_001014935.1
Listronotus bonariensis	GCA_014170235.1
Locusta migratoria	GCA_000516895.1
Loxosceles reclus	GCA_001188405.1
Lucilia cuprina	GCA_001187945.1
Lucilia cuprina	GCF_000699065.1
Lucilia sericata	GCA_001014835.1
Lucilia sericata	GCF_015586225.1
Lutzomyia longipalpis	GCA_000265325.1

Lycaena phlaeas	GCA_905333005.1
Lymantria dispar	GCA_016802235.1
Lymantria dispar dispar	GCA_004115105.1
Lymantria monacha	GCA_905163515.1
Lymantria monacha	GCA_905163525.1
Lysandra coridon	GCA_905220515.1
Lysandra coridon	GCA_905220525.1
Lysiphlebus fabarum	GCA_011426435.1
Machilis hrabei	GCA_003456935.1
Maconellicoccus hirsutus	GCA_003261595.1
Maconellicoccus hirsutus	GCA_900064465.1
Macrobrachium nipponense	GCA_015104395.1
Macrocentrus cingulum	GCA_002156465.1
Macrosiphum rosae	GCA_016617965.1
Magicicada septendecim	GCA_011326945.1
Magicicada septendecula	GCA_011763675.1
Mamestra brassicae	GCA_905163405.1
Mamestra brassicae	GCA_905163435.1
Mamestra configurata	GCA_002192655.1
Mamestra configurata	GCA_002192655.2
Manduca sexta	GCA_000262585.1
Manduca sexta	GCF_000262585.1
Manduca sexta	GCF_014839805.1
Maniola hyperantus	GCA_902806615.1
Maniola hyperantus	GCF_902806685.1
Maniola jurtina	GCA_009667785.1
Marronus borbonicus	GCA_902655005.1
Marronus borbonicus	GCA_902655005.2
Mayetiola destructor	GCA_000149185.1
Mayetiola destructor	GCA_001014435.1
Medauroidea extradentata	GCA_003012365.1
Medioppia subpectinata	GCA_904067535.1
Megachile rotundata	GCF_000220905.1
Megalopta genalis	GCF_011865705.1
Megaselia abdita	GCA_001015175.1
Megaselia scalaris	GCA_000341915.2
Megastigmus dorsalis	GCA_900490025.1
Megastigmus stigmatizans	GCA_900490015.1
Megathymus violae	GCA_003671415.1

Melanaphis sacchari	GCF_002803265.2
Melipona quadrifasciata	GCA_001276565.1
Melitaea cinxia	GCA_000716385.1
Melitaea cinxia	GCA_905220555.1
Melitaea cinxia	GCA_905220565.1
Mellicta athalia	GCA_905220535.1
Mellicta athalia	GCA_905220545.1
Mengenilla moldrzyki	GCA_000281935.1
Mesaphorura yosii	GCA_009869945.1
Mesobuthus martensii	GCA_000484575.1
Microplitis demolitor	GCF_000572035.2
Mimas tiliae	GCA_905332985.1
Mochlonyx cinctipes	GCA_001014845.1
Monomorium pharaonis	GCA_003575265.1
Monomorium pharaonis	GCF_000980195.1
Monomorium pharaonis	GCF_003260585.2
Monomorium pharaonis	GCF_013373865.1
Musca domestica	GCA_002191195.1
Musca domestica	GCA_014843735.1
Musca domestica	GCF_000371365.1
Mythimna impura	GCA_905147275.1
Mythimna impura	GCA_905147345.1
Myzus persicae	GCF_001856785.1
Nasonia giraulti	GCA_000004775.1
Nasonia giraulti	GCA_016647725.1
Nasonia longicornis	GCA_000004795.1
Nasonia vitripennis	GCF_000002325.3
Nasonia vitripennis	GCF_009193385.2
Nasutitermes exitiosus	GCA_001404035.1
Neelides sp. FZ-2019	GCA_009869795.1
Neodiprion lecontei	GCF_001263575.1
Neodiprion pinetum	GCA_004916985.1
Neoneuromus ignobilis	GCA_014529405.1
Nesidiocoris tenuis	GCA_902806785.1
Neuroterus quercusbaccarum	GCA_900490065.1
Neuroterus valhalla	GCA_011762715.1
Nicrophorus vespilloides	GCF_001412225.1
Nilaparvata lugens	GCA_015708395.1
Nilaparvata lugens	GCF_000757685.1

Nilaparvata lugens	GCF_014356525.1
Noctua fimbriata	GCA_905163415.1
Noctua fimbriata	GCA_905163425.1
Noctua pronuba	GCA_905220345.1
Nomia melanderi	GCA_003710045.1
Nomia melanderi	GCF_003710045.1
Notocelia uddmanniana	GCA_905163555.1
Notocelia uddmanniana	GCA_905163575.1
Notodonta dromedarius	GCA_905147325.1
Notodonta dromedarius	GCA_905147855.1
Nylanderia fulva	GCF_005281655.1
Nymphalis io	GCA_905147045.1
Nymphalis io	GCA_905147125.1
Nymphalis polychloros	GCA_905220575.1
Nymphalis polychloros	GCA_905220585.1
Nymphalis urticae	GCA_905147055.1
Nymphalis urticae	GCA_905147175.1
Nymphon striatum	GCA_016618385.1
Odontomachus brunneus	GCF_010583005.1
Oithona nana	GCA_900157175.1
Oncopeltus fasciatus	GCA_000696205.2
Oncopodura yosiiana	GCA_009869805.1
Onthophagus taurus	GCF_000648695.1
Ooceraea biroi	GCF_000611835.1
Ooceraea biroi	GCF_003672135.1
Operophtera brumata	GCA_001266575.1
Ophraella communa	GCA_902651945.1
Oppiella nova	GCA_904067525.1
Orchesella cincta	GCA_001718145.1
Orchestia grillus	GCA_014899125.1
Orius insidiosus	GCA_014119065.1
Ormyrus nitidulus	GCA_900474335.1
Ormyrus pomaceus	GCA_900474385.1
Ornithodoros moubata	GCA_905119505.1
Ornithodoros moubata	GCA_905119535.1
Ornithodoros moubata	GCA_905119575.1
Ornithodoros moubata	GCA_905119585.1
Ornithodoros moubata	GCA_905119595.1
Ornithodoros moubata	GCA_905119615.1

Ornithodoros moubata	GCA_905119635.1
Ornithodoros moubata	GCA_905119645.1
Ornithodoros moubata	GCA_905119655.1
Ornithodoros moubata	GCA_905119715.1
Ornithodoros porcinus	GCA_905119495.1
Ornithodoros porcinus	GCA_905119675.1
Ornithodoros porcinus	GCA_905119735.1
Ornithoptera priamus	GCA_011317755.1
Orussus abietinus	GCF_000612105.2
Oryctes borbonicus	GCA_001443705.1
Oryctes borbonicus	GCA_902654985.2
Oryzaephilus surinamensis	GCA_004796505.1
Osmia bicornis bicornis	GCF_004153925.1
Osmia lignaria	GCF_012274295.1
Ostrinia furnacalis	GCF_004193835.1
Ostrinia nubilalis	GCA_008921685.1
Pachypsylla venusta	GCA_000695645.2
Pachypsylla venusta	GCA_012654025.1
Palaemon carinicauda	GCA_004011675.1
Pandalus platyceros	GCA_005815305.1
Panonychus citri	GCA_014898815.1
Papilio aristodemus	GCA_016277805.1
Papilio bianor	GCA_011763625.1
Papilio dardanus tibullus	GCA_013186455.1
Papilio glaucus	GCA_000931545.1
Papilio machaon	GCF_001298355.1
Papilio memnon	GCA_003118335.3
Papilio memnon	GCA_003118415.2
Papilio polytes	GCF_000836215.1
Papilio xuthus	GCA_001298345.1
Papilio xuthus	GCF_000836235.1
Paracoccus marginatus	GCA_900065295.1
Paralithodes platypus	GCA_013283005.1
Pararge aegeria	GCA_900499025.1
Pararge aegeria	GCA_905163545.1
Pararge aegeria	GCF_905163445.1
Parasteatoda tepidariorum	GCF_000365465.2
Pardosa pseudoannulata	GCA_008065355.1
Parhyale hawaiensis	GCA_001587735.2

Paykullia maculata	GCA_003055125.1
Pediculus humanus corporis	GCF_000006295.1
Penaeus chinensis	GCA_016920825.1
Penaeus japonicus	GCA_017312705.1
Penaeus monodon	GCA_002291185.1
Penaeus monodon	GCA_007890405.1
Penaeus monodon	GCF_015228065.1
Penaeus vannamei	GCF_003789085.1
Penaeus vannamei?	GCA_003730335.1
Pentalonia nigronervosa	GCA_014851325.1
Periplaneta americana	GCA_002939525.1
Phalera bucephala	GCA_905147805.1
Phalera bucephala	GCA_905147815.1
Phenacoccus solenopsis	GCA_009761765.1
Philaenus spumarius	GCA_002233535.1
Phlebotomus papatasi	GCA_000262795.1
Phlogophora meticulosa	GCA_905147725.1
Phlogophora meticulosa	GCA_905147745.1
Phoebis sennae	GCA_001586405.1
Phormia regina	GCA_001735545.1
Phormia regina	GCA_001735585.1
Phortica variegata	GCA_001014415.1
Photinus pyralis	GCA_008802855.1
Photinus pyralis	GCF_008802855.1
Pieris brassicae	GCA_905147085.1
Pieris brassicae	GCA_905147105.1
Pieris napi	GCA_905231885.1
Pieris napi	GCA_905231895.1
Pieris rapae	GCA_905147735.1
Pieris rapae	GCA_905147795.1
Pieris rapae	GCF_001856805.1
Piezodorus guildinii	GCA_000786065.1
Pissodes strobi	GCA_016904865.1
Platorchestia sp. MABIK	GCA_014220935.1
Platynothrus peltifer	GCA_000988905.1
Plectrocnemia conspersa	GCA_009617715.1
Pleistodontes nigriventris	GCA_903653215.1
Plodia interpunctella	GCA_001368715.1
Plodia interpunctella	GCA_900182495.1

Plutella xylostella	GCA_000325945.1
Plutella xylostella	GCA_902505565.1
Plutella xylostella	GCA_902505575.1
Plutella xylostella	GCA_902505585.1
Plutella xylostella	GCF_000330985.1
Plutella xylostella	GCF_905116875.1
Pogonomyrmex barbatus	GCF_000187915.1
Pogonomyrmex californicus	GCA_017141365.1
Pogonus chalceus	GCA_002278615.1
Polistes canadensis	GCF_001313835.1
Polistes dominula	GCF_001465965.1
Polistes dorsalis	GCA_010416905.1
Polistes fuscatus	GCA_010416935.1
Polistes metricus	GCA_010416925.1
Pollicipes pollicipes	GCF_011947565.2
Polypedilum pembai	GCA_014622435.1
Popillia japonica	GCA_004785975.1
Portunus trituberculatus	GCA_008373055.1
Portunus trituberculatus	GCA_017591435.1
Priacma serrata	GCA_000281835.1
Procambarus virginalis	GCA_002838885.1
Proctacanthus coquilletti	GCA_001932985.1
Propylea japonica	GCA_013421045.1
Protaetia brevitarsis	GCA_004143645.1
Pseudachorutes palmiensis	GCA_009869845.1
Pseudoatta argentina	GCA_017607525.1
Pseudobourletiella spinata	GCA_009870155.1
Pseudococcus longispinus	GCA_900064475.1
Pseudomyrmex concolor	GCA_014839585.1
Pseudomyrmex cubaensis	GCA_014825685.1
Pseudomyrmex dendroicus	GCA_014825645.1
Pseudomyrmex elongatus	GCA_014825605.1
Pseudomyrmex flavicornis	GCA_014825475.1
Pseudomyrmex gracilis	GCF_002006095.1
Pseudomyrmex pallidus	GCA_014825455.1
Pseudomyrmex sp. PSW-54	GCA_014825495.1
Pseudoneuroterus saliens	GCA_900490055.1
Psoroptes ovis	GCA_002943765.1
Pteromalus puparum	GCA_012977825.2

Pygmarrhopalites habei	GCA_009870185.1
Rhagoletis pomonella	GCA_013731165.1
Rhagoletis pomonella	GCF_013731165.1
Rhagoletis zephyria	GCF_001687245.1
Rhinocypha anisoptera	GCA_011762765.1
Rhipicephalus annulatus	GCA_013436015.1
Rhipicephalus microplus	GCA_000181235.2
Rhipicephalus microplus	GCA_002176555.1
Rhipicephalus microplus	GCA_013435995.1
Rhipicephalus microplus	GCF_013339725.1
Rhipicephalus sanguineus	GCF_013339695.1
Rhodnius prolixus	GCA_000181055.3
Rhopalosiphum maidis	GCF_003676215.2
Rhynchophorus ferrugineus	GCA_012979105.1
Rhynchophorus ferrugineus	GCA_014462685.1
Rhynchophorus ferrugineus	GCA_014490705.1
Samia ricini	GCA_014132275.1
Sarcophaga bullata	GCA_001017455.1
Sarcophaga bullata	GCA_005959815.1
Sarcophaga peregrina	GCA_014635995.1
Sarcophagidae sp. BV-2014	GCA_001047195.1
Sarcoptes scabiei	GCA_000828355.1
Sarcoptes scabiei	GCA_014595675.1
Scaeva pyrastri	GCA_905146935.1
Scaeva pyrastri	GCA_905147095.1
Scaptodrosophila lebanonensis	GCA_001014445.1
Scaptodrosophila lebanonensis	GCA_003285725.2
Scaptodrosophila lebanonensis	GCF_003285725.1
Scaptomyza flava	GCA_003952975.1
Schizaphis graminum	GCA_003264975.1
Semibalanus balanoides	GCA_003709985.1
Semibalanus balanoides	GCA_014673585.1
Sericostoma sp. HW-2014	GCA_003003475.1
Sinella curviseta	GCA_004115045.1
Sinella curviseta	GCA_004115045.3
Sipha flava	GCF_003268045.1
Sitobion miscanthi	GCA_008086715.1
Sitodiplosis mosellana	GCA_009176505.1
Sitophilus oryzae	GCA_002938485.1

Sitophilus oryzae	GCF_002938485.1
Sminthurides bifidus	GCA_009872375.1
Sogatella furcifera	GCA_014356515.1
Sogatella furcifera	GCA_017141385.1
Solenopsis fugax	GCA_003595255.1
Solenopsis invicta	GCA_009299965.1
Solenopsis invicta	GCA_009299975.1
Solenopsis invicta	GCA_009650705.1
Solenopsis invicta	GCA_010367695.1
Solenopsis invicta	GCF_000188075.2
Solenopsis invicta	GCF_016802725.1
Sphyracephala brevicornis	GCA_001015235.1
Spilosoma lubricipeda	GCA_905220595.1
Spilosoma lubricipeda	GCA_905220605.1
Spodoptera exigua	GCA_011316535.1
Spodoptera exigua	GCA_015679615.1
Spodoptera frugiperda	GCA_000753635.2
Spodoptera frugiperda	GCA_002213285.1
Spodoptera frugiperda	GCA_002811805.1
Spodoptera frugiperda	GCA_012979215.2
Spodoptera frugiperda	GCA_015832365.1
Spodoptera frugiperda	GCA_900240015.1
Spodoptera frugiperda	GCF_011064685.1
Spodoptera litura	GCF_002706865.1
Steganacarus magnus	GCA_000988885.1
Stegodyphus dumicola	GCF_010614865.1
Stegodyphus mimosarum	GCA_000611955.2
Stenopsyche tienmushanensis	GCA_008973525.1
Stiretrus anchorago	GCA_010014745.1
Stomoxys calcitrans	GCF_001015335.1
Strigamia maritima	GCA_000239455.1
Synergus gifuensis	GCA_904066015.1
Synergus itoensis	GCA_904066005.1
Synergus japonicus	GCA_900474275.1
Synergus umbraculus	GCA_900474325.1
Syritta pipiens	GCA_905147025.1
Syritta pipiens	GCA_905187475.1
Tachina fera	GCA_905220375.1
Tachina fera	GCA_905220395.1

Tachypleus gigas	GCA_014155125.1
Tachypleus tridentatus	GCA_004102145.1
Tachypleus tridentatus	GCA_004210375.1
Tachypleus tridentatus	GCA_010883065.1
Tachypleus tridentatus	GCA_015741085.1
Taenaris catops	GCA_009936525.1
Teleogryllus occipitalis	GCA_011170035.1
Teleopsis dalmanni	GCA_001017525.1
Teleopsis dalmanni	GCA_002237135.1
Teleopsis dalmanni	GCA_014706505.1
Teleopsis dalmanni	GCF_002237135.1
Temnothorax curvispinosus	GCF_003070985.1
Temnothorax longispinosus	GCA_004794745.1
Tenebrio molitor	GCA_014282415.1
Tephritis californica	GCA_001017515.1
Tetragonula carbonaria	GCA_010645115.1
Tetragonula clypearis	GCA_010645135.1
Tetragonula davenporti	GCA_010645165.1
Tetragonula hockingsi	GCA_010645185.1
Tetragonula mellipes	GCA_011634685.1
Tetragonula mellipes	GCA_011634955.1
Tetramorium bicarinatum	GCA_011636605.1
Tetramorium immigrans	GCA_011636585.1
Tetramorium parvispinum	GCA_011638315.1
Tetramorium simillimum	GCA_011636635.1
Tetranychus urticae	GCF_000239435.1
Thalassaphorura encarpata	GCA_009869925.1
Thaumetopoea pityocampa	GCA_017165845.1
Themira minor	GCA_001014575.1
Thrips palmi	GCF_012932325.1
Thyatira batis	GCA_905147775.1
Thyatira batis	GCA_905147785.1
Tigriopus californicus	GCA_007210705.1
Tigriopus japonicus	GCA_010645155.1
Tigriopus kingsejongensis	GCA_012959195.1
Timema bartmani	GCA_902151455.1
Timema californicum	GCA_902141385.1
Timema cristinae	GCA_002009905.3
Timema cristinae	GCA_002926335.1

Timema cristinae	GCA_002928295.1
Timema cristinae	GCA_902151435.1
Timema douglasi	GCA_901482245.1
Timema genevievae	GCA_902155825.1
Timema monikensis	GCA_902151445.1
Timema podura	GCA_902151475.1
Timema poppensis	GCA_902141375.1
Timema shepardi	GCA_902151425.1
Timema tahoe	GCA_902151465.1
Tinea trinotella	GCA_905220615.1
Tinea trinotella	GCA_905220625.1
Tipula oleracea	GCA_001017535.1
Tisbe holothuriae	GCA_900659605.1
Tomocerus minor	GCA_009936455.1
Tomocerus qinae	GCA_009869885.1
Torymus auratus	GCA_900474315.1
Torymus geranii	GCA_900474355.1
Trabutina mannipara	GCA_900080175.1
Trachelipus rathkii	GCA_015478945.1
Trachymyrmex cornetzi	GCF_001594075.1
Trachymyrmex septentrionalis	GCF_001594115.1
Trachymyrmex zeteki	GCF_001594055.1
Trialeurodes vaporariorum	GCA_009741425.1
Trialeurodes vaporariorum	GCA_011764245.1
Triatoma infestans	GCA_011037195.1
Tribolium castaneum	GCF_000002335.3
Tribolium madens	GCA_015345945.1
Trichoceridae sp. BV-2014	GCA_001014425.1
Trichogramma brassicae	GCA_902806795.1
Trichogramma evanescens	GCA_902732785.1
Trichogramma pretiosum	GCF_000599845.2
Trichomalopsis sarcophagae	GCA_002249905.1
Trichonephila clavipes	GCA_002102615.1
Trichonephila clavipes	GCA_002291165.1
Trichoplusia ni	GCA_003604225.1
Trichoplusia ni	GCA_009663345.1
Trichoplusia ni	GCA_013978545.1
Trichoplusia ni	GCF_003590095.1
Trigoniulus corallinus	GCA_013389805.1

Trinorchestia longiramus	GCA_006783055.1
Trionymus perrisii	GCA_900050545.1
Tropilaelaps mercedesae	GCA_002081605.1
Trupanea jonesi	GCA_001014665.1
Trypoxylus dichotomus	GCA_014905495.1
Tuta absoluta	GCA_004799115.1
Tyrophagus putrescentiae	GCA_012066115.1
Vanessa atalanta	GCA_905147705.1
Vanessa atalanta	GCA_905147765.1
Vanessa cardui	GCA_905220355.1
Vanessa cardui	GCA_905220365.1
Vanessa tameamea	GCF_002938995.1
Varroa destructor	GCA_000181155.2
Varroa destructor	GCF_002443255.1
Varroa jacobson	GCF_002532875.1
Vespa mandarinia	GCA_014083525.1
Vespa mandarinia	GCF_014083535.2
Vespula germanica	GCA_014466195.1
Vespula pensylvanica	GCA_014466175.1
Vespula vulgaris	GCA_014466185.1
Vollenhovia emeryi	GCF_000949405.1
Wasmannia auropunctata	GCF_000956235.1
Xenocatantops brachycerus	GCA_900249655.1
Xestia xanthographa	GCA_905147715.1
Xestia xanthographa	GCA_905147755.1
Xylota sylvarum	GCA_905220385.1
Xylota sylvarum	GCA_905220405.1
Zanna intricata	GCA_010016005.2
Zaprionus indianus	GCA_001752445.1
Zerene cesonia	GCA_012273895.2
Zerene cesonia	GCF_012273895.1
Zeugodacus cucurbitae	GCF_000806345.1
Zootermopsis nevadensis	GCF_000696155.1
Daphnia dubia	GCA_013387435.1
Daphnia magna	GCA_001632505.1
Daphnia magna	GCA_003990815.1
Daphnia magna	GCF_003990815.1
Daphnia obtusa	GCA_016170125.1
Daphnia pulex	GCA_000187875.1

Daphnia pulex	GCA_900092285.1
Daphnia pulex	GCA_900092285.2
Daphnia sinensis	GCA_013167095.1
Eulimnadia texana	GCA_002872375.1
Lepidurus apus lubbocki	GCA_003723985.1
Lepidurus arcticus	GCA_003724045.1
Triops cancriformis	GCA_000981345.1
Brachionus sp. *'Tiscar'*	GCA_006352455.1
Brachionus koreanus	GCA_009177125.1
Brachionus koreanus	GCA_016987375.1
Biomphalaria glabrata	GCA_014524965.1
Biomphalaria glabrata	GCA_014525025.1
Hydra vulgaris	GCA_000219015.1
Mnemiopsis leidyi	GCA_000226015.1
Crassostrea gigas	GCA_000297895.2
Capitella teleta	GCA_000328365.1
Adineta vaga	GCA_000513175.1
Pleurobrachia bachei	GCA_000695325.1
Mytilus galloprovincialis	GCA_000715055.1
Dreissena polymorpha	GCA_000806325.1
Thelohanellus kitauei	GCA_000827895.1
Conus tribblei	GCA_001262575.1
Rotaria magnacalcarata	GCA_001317505.1
Kudoa iwatai	GCA_001407235.2
Kudoa iwatai	GCA_001407335.1
Hypsibius dujardini	GCA_001455005.1
Sphaeromyxa zaharoni	GCA_001455285.1
Enteromyxum leei	GCA_001455295.2
Hypsibius dujardini	GCA_001579985.1
Corbicula fluminea	GCA_001632725.1
Intoshia linei	GCA_001642005.1
Mytilus galloprovincialis	GCA_001676915.1
Hydroides elegans	GCA_001703475.1
Orbicella faveolata	GCA_001896105.1
Ramazzottius varieornatus	GCA_001949185.1
Radix auricularia	GCA_002072015.1
Gigantidas platifrons	GCA_002080005.1
Modiolus philippinarum	GCA_002080025.1
Hypsibius exemplaris	GCA_002082055.1

Pinctada imbricata	GCA_002216045.1
Phoronis australis	GCA_002633005.1
Notospermus geniculatus	GCA_002633025.1
Brachionus calyciflorus	GCA_002922825.1
Euperipatoides rowelli	GCA_003024985.2
Limnoperna fortunei	GCA_003130415.1
Haliotis rufescens	GCA_003343065.1
Trichoplax sp. H2	GCA_003344405.1
Venustaconcha ellipsiformis	GCA_003401595.1
Saccostrea glomerata	GCA_003671525.1
Craspedacusta sowerbii	GCA_003687565.1
Brachionus plicatilis	GCA_003710015.1
Nemopilema nomurai	GCA_003864495.1
Haliotis rubra	GCA_003918875.1
Octopus vulgaris	GCA_003957725.1
Morbakka virulenta	GCA_003991215.1
Elysia chlorotica	GCA_003991915.1
Eisenia fetida	GCA_003999395.1
Hydra viridissima	GCA_004118115.1
Hydra oligactis	GCA_004118135.1
Conus consors	GCA_004193615.1
Aurelia aurita complex sp. *Pacific*	GCA_004194395.1
Aurelia aurita	GCA_004194415.1
Physella acuta	GCA_004329575.1
Argopecten irradians irradians	GCA_004382745.1
Argopecten irradians concentricus	GCA_004382765.1
Euprymna scolopes	GCA_004765925.1
Pomacea maculata	GCA_004794325.1
Pomacea canaliculata	GCA_004794335.1
Lanistes nyassanus	GCA_004794575.1
Marisa cornuarietis	GCA_004794655.1
Crassostrea gigas	GCA_005518195.2
Brachionus asplanchnoidis	GCA_006352335.1
Brachionus rotundiformis	GCA_006352405.1
Brachionus plicatilis	GCA_006352415.1
Brachionus asplanchnoidis	GCA_006352485.1
Architeuthis dux	GCA_006491835.1
Montipora capitata	GCA_006542545.1
Dreissena rostriformis	GCA_007657795.1

Sinonovacula constricta	GCA_007844125.1
Haliotis laevigata	GCA_008038995.1
Lutraria rhynchaena	GCA_008271625.1
Alatina alata	GCA_008930755.1
Ruditapes philippinarum	GCA_009026015.1
Lamellibrachia luymesi	GCA_009193005.1
Cristatella mucedo	GCA_009760855.2
Achatina immaculata	GCA_009760885.1
Sinonovacula constricta	GCA_009762815.1
Phymanthus crucifer	GCA_009858155.1
Limacina bulimoides	GCA_009866985.1
Henneguya salminicola	GCA_009887335.1
Chrysaora fuscescens	GCA_009936425.1
Anentome helena	GCA_009936545.1
Alatinidae sp. Z8VKAUB7J3	GCA_010016025.1
Carybdea marsupialis auct. non (Linnaeus, 1758)	GCA_010016065.1
Myxobolus squamalis	GCA_010108815.1
Brachionus plicatilis	GCA_010279815.1
Bugula neritina	GCA_010799875.2
Crassostrea gigas	GCA_011032805.1
Beroe forskalii	GCA_011033025.1
Actinia equina	GCA_011057435.1
Dicyema japonicum	GCA_011109175.1
Babylonia areolata	GCA_011634625.1
Aurelia coerulea	GCA_011634815.1
Mytilus coruscus	GCA_011752425.2
Heteractis magnifica	GCA_011763375.1
Chrysaora chesapeakei	GCA_011763395.1
Stichodactyla mertensii	GCA_011800005.1
Hirudo medicinalis	GCA_011800805.1
Chrysaora quinquecirrha	GCA_012295145.1
Chrysomallon squamiferum	GCA_012295275.1
Cyclina sinensis	GCA_012932295.1
Pomphorhynchus laevis	GCA_012934845.1
Sanderia malayensis	GCA_013076295.1
Rhopilema esculentum	GCA_013076305.1
Ephydatia muelleri	GCA_013339895.1
Tegillarca granosa	GCA_013375625.1

Adineta vaga	GCA_013411005.1
Acropora millepora	GCA_013753865.1
Cepaea nemoralis	GCA_014155875.1
Biomphalaria glabrata	GCA_014524955.1
Chrysaora quinquecirrha	GCA_014526335.1
Pocillopora verrucosa	GCA_014529365.1
Acropora tenuis	GCA_014633955.1
Acropora acuminata	GCA_014633975.1
Acropora awi	GCA_014634005.1
Acropora cytherea	GCA_014634045.1
Acropora digitifera	GCA_014634065.1
Acropora echinata	GCA_014634105.1
Acropora gemmifera	GCA_014634125.1
Acropora hyacinthus	GCA_014634145.1
Acropora microphthalma	GCA_014634165.1
Astreopora myriophthalma	GCA_014634185.1
Acropora nasuta	GCA_014634205.1
Acropora yongei	GCA_014634225.1
Montipora cactus	GCA_014634245.1
Montipora efflorescens	GCA_014634505.1
Acropora selago	GCA_014634525.1
Acropora muricata	GCA_014634545.1
Acropora intermedia	GCA_014634585.1
Acropora florida	GCA_014634605.1
Hydra viridissima	GCA_014706445.1
Mercenaria mercenaria	GCA_014805675.1
Archivesica marissinica	GCA_014843695.1
Stichodactyla helianthus	GCA_015163945.1
Heteractis crispa	GCA_015164035.1
Chrysaora achlyos	GCA_015164055.1
Poecilobdella manillensis	GCA_015345955.1
Watasenia scintillans	GCA_015471945.1
Hapalochlaena maculosa	GCA_015501135.1
Crassostrea hongkongensis	GCA_015776775.1
Margaritifera margaritifera	GCA_015947965.1
Anthopleura sola	GCA_016068315.1
Gigantopelta aegis	GCA_016097555.1
Dracogyra subfuscus	GCA_016106625.1
Pinna nobilis	GCA_016161895.1

Magallana hongkongensis	GCA_016163765.1
Acanthopleura granulata	GCA_016165875.1
Trachythela sp. YZ-2020	GCA_016169945.1
Amphimedon queenslandica	GCA_016292275.1
Pachyseris speciosa	GCA_016490735.1
Megalonaias nervosa	GCA_016617855.1
Potamilus streckersoni	GCA_016746295.1
Conus betulinus	GCA_016801955.1
Brachionus paranguensis	GCA_016802215.1
Brachionus rotundiformis	GCA_016802295.1
Mytilus coruscus	GCA_017311375.1
Eisenia fetida	GCA_900000155.1
Colubraria reticulata	GCA_900004695.1
Lymnaea stagnalis	GCA_900036025.1
Renilla reniformis	GCA_900177555.1
Amynthas corticis	GCA_900184015.1
Amynthas corticis	GCA_900184025.1
Anemonia viridis	GCA_900234385.1
Adineta vaga	GCA_900239655.1
Adineta vaga	GCA_900239665.1
Rotaria macrura	GCA_900239685.1
Rotaria macrura	GCA_900239695.1
Rotaria magnacalcarata	GCA_900239705.1
Adineta ricciae	GCA_900239715.1
Rotaria magnacalcarata	GCA_900239745.1
Beroe ovata	GCA_900239995.1
Adineta ricciae	GCA_900240375.1
Calvadosia cruxmelitensis	GCA_900245855.1
Aplysina aerophoba	GCA_900275565.1
Aplysina aerophoba	GCA_900275575.1
Aplysina aerophoba	GCA_900275585.1
Aplysina aerophoba	GCA_900275595.1
Aplysina aerophoba	GCA_900275605.1
Porites rus	GCA_900290455.1
Cassiopea xamachana	GCA_900291935.1
Mytilus galloprovincialis	GCA_900618805.1
Pecten maximus	GCA_902652895.1
Clytia hemisphaerica	GCA_902728285.1
Panopea generosa	GCA_902825435.1

Hirudo medicinalis	GCA_903470615.1
Sepia pharaonis	GCA_903632075.3
Ostrea lurida	GCA_903981925.1
Dimorphilus gyrociliatus	GCA_904063045.1
Aplysia californica	GCF_000002075.1
Hydra vulgaris	GCF_000004095.1
Amphimedon queenslandica	GCF_000090795.1
Trichoplax adhaerens	GCF_000150275.1
Nematostella vectensis	GCF_000209225.1
Acropora digitifera	GCF_000222465.1
Helobdella robusta	GCF_000326865.1
Lottia gigantea	GCF_000327385.1
Biomphalaria glabrata	GCF_000457365.1
Priapulus caudatus	GCF_000485595.1
Octopus bimaculoides	GCF_001194135.1
Exaiptasia diaphana	GCF_001417965.1
Crassostrea virginica	GCF_002022765.2
Orbicella faveolata	GCF_002042975.1
Mizuhopecten yessoensis	GCF_002113885.1
Stylophora pistillata	GCF_002571385.1
Pomacea canaliculata	GCF_003073045.1
Pocillopora damicornis	GCF_003704095.1
Acropora millepora	GCF_004143615.1
Dendronephthya gigantea	GCF_004324835.1
Octopus sinensis	GCF_006345805.1
Actinia tenebrosa	GCF_009602425.1
Pecten maximus	GCF_902652985.1
Crassostrea gigas	GCF_902806645.1
Lingula anatina	GCF_001039355.2
Trichomonas vaginalis G3	GCA_000002825.1
Tritrichomonas foetus	GCA_001839685.1
Trichomonas vaginalis	GCA_002891335.1
Trichomonas gallinae	GCA_008369845.1
Trichomonas tenax	GCA_900231805.1
Tritrichomonas foetus	GCA_905133005.1
Trichomonas vaginalis	GCF_000002825.2
Trypanosoma brucei brucei TREU927	GCA_000002445.1
Eimeria tenella strain Houghton	GCA_000002835.1
Monosiga brevicollis MX1	GCA_000002865.1

Plasmodium yoelii yoelii 17XNL	GCA_000003085.2
Plasmodium knowlesi strain H	GCA_000006355.3
Hemiselmis andersenii	GCA_000018645.1
Globisporangium ultimum DAOM BR144	GCA_000143045.1
Plasmodium falciparum HB3	GCA_000149665.2
Toxoplasma gondii GT1	GCA_000149715.2
Phytophthora ramorum	GCA_000149735.1
Phytophthora sojae	GCA_000149755.2
Plasmodium falciparum Dd2	GCA_000149795.1
Toxoplasma gondii VEG	GCA_000150015.2
Plasmodium falciparum VS/1	GCA_000150295.1
Plasmodium falciparum Senegal_V34.04	GCA_000150315.1
Plasmodium falciparum RO-33	GCA_000150335.1
Plasmodium falciparum K1	GCA_000150355.1
Plasmodium falciparum FCC-2/Hainan	GCA_000150375.1
Plasmodium falciparum D10	GCA_000150395.1
Plasmodium falciparum D6	GCA_000150415.1
Plasmodium falciparum 7G8	GCA_000150435.3
Plasmodium falciparum Santa Lucia	GCA_000150455.3
Saprolegnia parasitica CBS 223.65	GCA_000151545.2
Ascogregarina taiwanensis	GCA_000172235.1
Hyaloperonospora arabidopsidis Emoy2	GCA_000173235.2
Giardia intestinalis ATCC 50581	GCA_000182405.1
Giardia lamblia P15	GCA_000182665.1
Plasmodium falciparum RAJ116	GCA_000186025.2
Plasmodium falciparum IGH-CR14	GCA_000186055.2
Trypanosoma cruzi	GCA_000188675.2
Acanthamoeba castellanii	GCA_000193105.1
Cryptomonas paramecium	GCA_000194455.1
Neospora caninum Liverpool	GCA_000208865.2
Entamoeba histolytica HM-1:IMSS	GCA_000208925.2
Entamoeba dispar SAW760	GCA_000209125.2
Cryptosporidium parvum	GCA_000209695.1
Astrammina rara	GCA_000211355.2
Gregarina niphandrodes	GCA_000223845.4
Toxoplasma gondii RUB	GCA_000224805.2
Toxoplasma gondii TgCATBr9	GCA_000224825.2
Toxoplasma gondii VAND	GCA_000224845.2
Toxoplasma gondii MAS	GCA_000224865.2

Toxoplasma gondii p89	GCA_000224885.2
Toxoplasma gondii FOU	GCA_000224905.2
Nannochloropsis oceanica	GCA_000226695.1
Leishmania donovani	GCA_000227135.2
Trypanosoma vivax Y486	GCA_000227375.1
Trypanosoma congolense IL3000	GCA_000227395.2
Tetrahymena elliotti 4EA	GCA_000231825.2
Tetrahymena malaccensis 436	GCA_000231845.2
Leishmania mexicana MHOM/GT/2001/U1103	GCA_000234665.4
Phytophthora parasitica INRA-31*0*	GCA_000247585.2
Leishmania major strain SD 75.1	GCA_000250755.2
Toxoplasma gondii ARI	GCA_000250965.2
Pseudoperonospora cubensis	GCA_000252605.1
Toxoplasma gondii CAST	GCA_000256705.2
Toxoplasma gondii TgCatPRC2	GCA_000256725.2
Hammondia hammondi	GCA_000258005.2
Toxoplasma gondii TgCATBr5	GCA_000259835.1
Tetrahymena borealis	GCA_000260095.1
Tetrahymena thermophila SB210	GCA_000261185.1
Polysphondylium violaceum	GCA_000277445.1
Dictyostelium intermedium	GCA_000277465.1
Dictyostelium firmibasis	GCA_000277485.1
Toxoplasma gondii CtCo5	GCA_000278365.1
Leishmania donovani Ld 2001	GCA_000283395.1
Dictyostelium citrinum	GCA_000286055.1
Chroomonas mesostigmatica CCMP1168	GCA_000286095.1
Oxytricha trifallax	GCA_000295675.2
Trypanosoma cruzi marinkellei	GCA_000300495.1
Ectocarpus siliculosus	GCA_000310025.1
Albugo candida	GCA_000313105.1
Leishmania donovani Ld 39	GCA_000316305.1
Phytophthora lateralis MPF4	GCA_000318465.2
Bigelowiella natans	GCA_000320545.1
Plasmodium vivax India VII	GCA_000320625.2
Plasmodium vivax Brazil I	GCA_000320645.2
Plasmodium vivax Mauritania I	GCA_000320665.2
Plasmodium vivax North Korean	GCA_000320685.2
Plasmodium cynomolgi strain B	GCA_000321355.1

Toxoplasma gondii GAB2-2007-GAL-DOM2	GCA_000325525.2
Stylonychia lemnae 2x8/2	GCA_000325865.3
Phytophthora capsici LT1534	GCA_000325885.1
Albugo candida	GCA_000326045.1
Albugo candida	GCA_000326065.1
Hyphochytrium catenoides	GCA_000327025.2
Trypanosoma cruzi strain Esmeraldo	GCA_000327425.1
Phytomonas serpens 9T	GCA_000331125.1
Crithidia fasciculata	GCA_000331325.2
Leishmania major strain LV39c5	GCA_000331345.1
Trypanosoma cruzi JR cl. 4	GCA_000331405.1
Phytophthora lateralis	GCA_000333055.2
Phytophthora kernoviae 00238/432	GCA_000333075.3
Phytophthora kernoviae 00629/1	GCA_000333095.2
Phytophthora kernoviae 00844/4	GCA_000333115.2
Endotrypanum monterogeii	GCA_000333855.2
Leptomonas seymouri BHU1095	GCA_000333875.2
Phytophthora ramorum	GCA_000336535.2
Toxoplasma gondii COUG	GCA_000338675.2
Phytophthora lateralis MPF6	GCA_000338795.2
Phytophthora lateralis SMST21	GCA_000338815.2
Entamoeba histolytica KU27	GCA_000338855.1
Leishmania braziliensis MHOM/BR/75/M2903	GCA_000340355.2
Phytophthora ramorum	GCA_000340395.2
Leishmania panamensis MHOM/COL/81/L13	GCA_000340495.1
Entamoeba histolytica HM-1:IMSS-B	GCA_000344925.1
Entamoeba histolytica HM-3:IMSS	GCA_000346345.1
Trypanosoma cruzi Tula cl2	GCA_000365225.1
Entamoeba histolytica HM-1:IMSS-A	GCA_000365475.1
Phytophthora parasitica P1569	GCA_000365505.1
Phytophthora parasitica P1976	GCA_000365525.1
Phytophthora parasitica CJ01A1	GCA_000365545.1
Phytophthora parasitica P10297	GCA_000367145.1
Globisporangium irregulare DAOM BR486	GCA_000387425.2
Pythium aphanidermatum DAOM BR444	GCA_000387445.2
Globisporangium iwayamae DAOM BR242034	GCA_000387465.2

Pythium arrhenomanes ATCC 12531	GCA_000387505.2
Globisporangium ultimum var. *sporangiiferum* BR650	GCA_000387525.2
Phytopythium vexans DAOM BR484	GCA_000387545.2
Fonticula alba	GCA_000388065.2
Plasmodium falciparum NF54	GCA_000401695.2
Plasmodium falciparum UGT5.1	GCA_000401715.2
Leishmania sp. MAR LEM2494	GCA_000409445.2
Leishmania arabica	GCA_000410695.2
Leishmania tropica L590	GCA_000410715.1
Leishmania enriettii	GCA_000410755.2
Physarum polycephalum	GCA_000413255.3
Leishmania amazonensis	GCA_000438535.1
Phytophthora x alni	GCA_000439335.1
Leishmania turanica	GCA_000441995.1
Strigomonas culicis	GCA_000442495.1
Leishmania gerbilli	GCA_000443025.1
Phytophthora cambivora	GCA_000443045.1
Leishmania aethiopica L147	GCA_000444285.2
Hammondia hammondi	GCA_000447165.1
Phytophthora kernoviae	GCA_000448265.2
Phytophthora cryptogea	GCA_000468175.2
Leishmania donovani	GCA_000470725.1
Crithidia acanthocephali	GCA_000482105.1
Strigomonas galati	GCA_000482125.1
Strigomonas culicis	GCA_000482145.1
Strigomonas oncopelti	GCA_000482165.1
Herpetomonas muscarum	GCA_000482205.1
Trypanosoma rangeli SC58	GCA_000492115.1
Trypanosoma cruzi Dm28c	GCA_000496795.1
Spironucleus salmonicida	GCA_000497125.1
Heterococcus sp. DN1	GCA_000498555.1
Giardia intestinalis	GCA_000498715.1
Giardia intestinalis	GCA_000498735.1
Naegleria fowleri	GCA_000499105.1
Eimeria praecox	GCA_000499445.1
Eimeria tenella	GCA_000499545.1
Eimeria brunetti	GCA_000499725.1
Phytophthora lateralis	GCA_000500205.2

Phytophthora pinifolia	GCA_000500225.2
Plasmodium yoelii 17X	GCA_000505035.1
Breviolum minutum Mf 1.05b.01	GCA_000507305.1
Phytophthora parasitica	GCA_000509465.1
Phytophthora parasitica	GCA_000509485.1
Phytophthora parasitica	GCA_000509505.1
Phytophthora parasitica	GCA_000509525.1
Reticulomyxa filosa	GCA_000512085.1
Plasmodium falciparum Vietnam Oak-Knoll (FVO)	GCA_000521015.1
Plasmodium falciparum MaliPS096_E11	GCA_000521035.1
Plasmodium falciparum Tanzania (2000708)	GCA_000521055.1
Plasmodium falciparum NF135/5.C10	GCA_000521075.1
Plasmodium falciparum Palo Alto/Uganda	GCA_000521095.1
Plasmodium falciparum CAMP/Malaysia	GCA_000521115.1
Plasmodium falciparum FCH/4	GCA_000521155.1
Plasmodium vinckei petteri	GCA_000524515.1
Nannochloropsis gaditana	GCA_000569095.1
Plasmodium gaboni	GCA_000576715.1
Phytomonas sp. *isolate* EM1	GCA_000582765.1
Chromera velia	GCA_000585135.1
Lotmaria passim	GCA_000635995.1
Phytophthora fragariae	GCA_000686205.4
Phytophthora rubi	GCA_000687305.2
Lotharella oceanica	GCA_000698435.2
Plasmodium vinckei vinckei	GCA_000709005.1
Oxytricha trifallax	GCA_000711775.1
Paramecium caudatum	GCA_000715435.1
Plasmodium reichenowi	GCA_000723685.1
Plasmodium coatneyi	GCA_000725905.1
Sarcocystis neurona	GCA_000727475.1
Paramecium sexaurelia	GCA_000733375.1
Paramecium biaurelia	GCA_000733385.1
Stylonychia lemnae	GCA_000751175.1
Phytophthora pisi	GCA_000751395.2
Pythium insidiosum	GCA_000764265.1
Mastigamoeba balamuthi	GCA_000765095.1
Cyclospora cayetanensis	GCA_000769155.2
Phytophthora kernoviae	GCA_000785725.3

Phytophthora kernoviae	GCA_000785735.3
Acytostelium subglobosum LB1	GCA_000787575.2
Cryptosporidium hominis	GCA_000804495.1
Schizochytrium sp. CCTCC M209059	GCA_000818945.1
Acanthamoeba astronyxis	GCA_000826245.1
Acanthamoeba culbertsoni	GCA_000826265.1
Acanthamoeba lenticulata	GCA_000826285.1
Acanthamoeba healyi	GCA_000826305.1
Acanthamoeba palestinensis	GCA_000826325.1
Acanthamoeba polyphaga	GCA_000826345.1
Acanthamoeba royreba	GCA_000826365.1
Acanthamoeba rhysodes	GCA_000826385.1
Acanthamoeba divionensis	GCA_000826405.1
Acanthamoeba lugdunensis	GCA_000826425.1
Acanthamoeba quina	GCA_000826445.1
Acanthamoeba mauritaniensis	GCA_000826465.1
Acanthamoeba castellanii	GCA_000826485.1
Acanthamoeba pearcei	GCA_000826505.1
Eimeria nieschulzi	GCA_000826945.1
Cryptosporidium sp. chipmunk LX-2015	GCA_000831705.1
Sarcocystis neurona	GCA_000875885.1
Albugo candida	GCA_000961115.1
Blastocystis sp. subtype 2	GCA_000963365.1
Blastocystis sp. subtype 3	GCA_000963385.1
Blastocystis sp. subtype 4	GCA_000963395.1
Blastocystis sp. subtype 6	GCA_000963415.1
Blastocystis sp. subtype 8	GCA_000963455.1
Blastocystis sp. subtype 9	GCA_000963465.1
Leishmania sp. AIIMS/LM/SS/PKDL/LD-974	GCA_000981925.2
Phytomonas sp. isolate Hart1	GCA_000982615.1
Pythium insidiosum	GCA_001029375.1
Plasmodiophora brassicae	GCA_001049375.1
Albugo candida	GCA_001078535.1
Vitrella brassicaformis CCMP3155	GCA_001179505.1
Balamuthia mandrillaris	GCA_001185145.1
Perkinsela sp. CCAP 1560/4	GCA_001235845.1
Balamuthia mandrillaris	GCA_001262475.1
Urostyla sp. PUJRC_G1	GCA_001272955.2

Paraurostyla sp. PUJRC_G6	GCA_001272965.2
Laurentiella sp. PUJRC_G5	GCA_001272975.2
Tetmemena sp. SeJ-2015	GCA_001273295.2
Sterkiella histriomuscorum	GCA_001273305.2
Chrysochromulina tobinii	GCA_001275005.1
Phytophthora ramorum	GCA_001278135.1
Phytophthora ramorum	GCA_001278145.1
Phytophthora ramorum	GCA_001278155.1
Phytophthora ramorum	GCA_001278165.1
Phytophthora ramorum	GCA_001278215.1
Phytophthora ramorum	GCA_001278225.1
Phytophthora ramorum	GCA_001278235.1
Oxytricha trifallax	GCA_001297925.1
Leptomonas seymouri	GCA_001299535.1
Cryptosporidium hominis	GCA_001305325.1
Cryptosporidium parvum	GCA_001305335.1
Cryptosporidium hominis	GCA_001305395.1
Cryptosporidium parvum	GCA_001305415.1
Cryptosporidium parvum	GCA_001305435.1
Cryptosporidium parvum	GCA_001305455.2
Cryptosporidium parvum	GCA_001305475.1
Cyclospora cayetanensis	GCA_001305735.1
Cryptosporidium parvum	GCA_001306235.1
Cryptosporidium parvum	GCA_001306245.1
Albugo candida	GCA_001306755.1
Albugo candida	GCA_001306775.1
Cryptosporidium hominis	GCA_001307845.1
Phytophthora multivora	GCA_001314345.1
Phytophthora multivora	GCA_001314355.1
Phytophthora cinnamomi	GCA_001314365.1
Phytophthora taxon totara	GCA_001314375.1
Phytophthora pluvialis	GCA_001314425.1
Phytophthora agathidicida	GCA_001314435.1
Phytophthora agathidicida	GCA_001314445.1
Phytophthora pluvialis	GCA_001314455.1
Phytophthora cinnamomi	GCA_001314505.1
Phytophthora taxon totara	GCA_001314925.1
Leishmania peruviana	GCA_001403675.1
Leishmania peruviana	GCA_001403695.1

Hyaloperonospora arabidopsidis	GCA_001414265.1
Hyaloperonospora arabidopsidis	GCA_001414525.1
Pseudocohnilembus persalinus	GCA_001447515.1
Trypanosoma equiperdum	GCA_001457755.2
Bodo saltans	GCA_001460835.1
Aurantiochytrium sp. T66	GCA_001462505.1
Phytophthora nicotianae	GCA_001482985.1
Phytophthora nicotianae	GCA_001483015.1
Cryptosporidium hominis	GCA_001483505.1
Cryptosporidium hominis	GCA_001483515.1
Cryptosporidium hominis	GCA_001483535.1
Giardia intestinalis assemblage A	GCA_001493575.1
Condylostoma magnum	GCA_001499635.1
Giardia intestinalis assemblage B	GCA_001543975.1
Acanthamoeba polyphaga	GCA_001567625.1
Pythium oligandrum	GCA_001573145.1
Sphaeroforma sirkka	GCA_001586965.3
Toxoplasma gondii RH	GCA_001593265.1
Cryptosporidium meleagridis	GCA_001593445.1
Cryptosporidium baileyi	GCA_001593455.1
Cryptosporidium hominis	GCA_001593465.1
Cryptosporidium hominis	GCA_001593475.1
Pilasporangium apinafurcum	GCA_001600475.1
Pilasporangium apinafurcum	GCA_001600495.1
Tieghemostelium lacteum	GCA_001606155.1
Nannochloropsis gaditana	GCA_001614215.1
Nannochloropsis limnetica	GCA_001614225.1
Nannochloropsis oceanica OZ-1	GCA_001614235.1
Nannochloropsis salina CCMP1776	GCA_001614245.1
Haemoproteus tartakovskyi	GCA_001625125.1
Euglena gracilis var. *bacillaris*	GCA_001638955.1
Monocercomonoides exilis	GCA_001643675.1
Blastocystis sp. ATCC 50177/Nand II	GCA_001651215.1
Prorocentrum minimum	GCA_001652855.1
Uroleptopsis citrina	GCA_001653735.1
Diplonema papillatum	GCA_001655075.1
Lenisia limosa	GCA_001655205.1
Phytophthora infestans	GCA_001661535.1
Entamoeba histolytica	GCA_001662325.1

Halocafeteria seosinensis	GCA_001687465.1
Plasmopara viticola	GCA_001695595.3
Phytophthora kernoviae	GCA_001707905.2
Nothophytophthora sp. Chile5	GCA_001712635.2
Phytophthora kernoviae	GCA_001712645.2
Phytophthora kernoviae	GCA_001712655.1
Phytophthora kernoviae	GCA_001712705.2
Phytophthora kernoviae	GCA_001712715.2
Plasmodium falciparum 3D7	GCA_001715585.1
Cladosiphon okamuranus	GCA_001742925.1
Phytomonas francai	GCA_001766655.1
Cryptosporidium ubiquitum	GCA_001835705.1
Cryptosporidium ubiquitum	GCA_001835715.1
Cryptosporidium andersoni	GCA_001835725.1
Cryptosporidium andersoni	GCA_001835735.1
Plasmodium falciparum 58.1	GCA_001861045.1
Plasmodium falciparum 327.1	GCA_001861055.1
Plasmodium falciparum 377.1	GCA_001861065.1
Plasmodium falciparum 303.1	GCA_001861075.1
Plasmodium falciparum 309.1	GCA_001861135.1
Plasmodium falciparum 318.1	GCA_001861165.1
Plasmodium falciparum 365.1	GCA_001861175.1
Plasmodium falciparum 383.1	GCA_001861195.1
Plasmodium falciparum 397.1	GCA_001861215.1
Plasmodium falciparum 326.1	GCA_001861235.1
Plasmodium falciparum 366.1	GCA_001861275.1
Plasmodium falciparum 398.1	GCA_001861305.1
Cryptosporidium andersoni	GCA_001865355.1
Nannochloropsis oceanica strain IMET1	GCA_001870945.1
Euplotes focardii	GCA_001880345.1
Moneuplotes crassus	GCA_001880385.1
Plasmodium brasilianum	GCA_001885115.2
Sclerospora graminicola	GCA_001887855.2
Pythium periplocum	GCA_001922765.1
Phytophthora ramorum	GCA_001933315.1
Phytophthora ramorum	GCA_001933325.1
Phytophthora ramorum	GCA_001933335.1
Phytophthora ramorum	GCA_001933345.1
Phytophthora ramorum	GCA_001933395.1

Phytophthora ramorum	GCA_001933405.1
Phytophthora ramorum	GCA_001933415.1
Phytophthora ramorum	GCA_001933455.1
Phytophthora ramorum	GCA_001933465.1
Phytophthora ramorum	GCA_001933485.1
Symbiodinium microadriaticum	GCA_001939145.1
Cryptosporidium hominis	GCA_001945495.1
Pythium insidiosum	GCA_001950795.1
Pythium insidiosum	GCA_001950815.1
Pythium insidiosum	GCA_001950835.1
Pythium insidiosum	GCA_001950855.1
Pythium insidiosum	GCA_001950875.1
Pythium insidiosum	GCA_001950895.1
Pythium insidiosum	GCA_001950915.1
Pythium insidiosum	GCA_001950935.1
Pythium insidiosum	GCA_001950955.1
Phytophthora ramorum	GCA_001955675.1
Stentor coeruleus	GCA_001970955.1
Plasmopara viticola	GCA_001974925.1
Leishmania donovani	GCA_001989955.1
Leishmania donovani	GCA_001989975.1
Cyclospora cayetanensis	GCA_002019455.1
Cyclospora cayetanensis	GCA_002019465.1
Cyclospora cayetanensis	GCA_002019475.1
Cyclospora cayetanensis	GCA_002019905.1
Creolimax fragrantissima	GCA_002024145.1
Acanthamoeba comandoni	GCA_002025285.1
Phytophthora fragariae	GCA_002025845.1
Phytophthora rubi	GCA_002025925.1
Pythium oligandrum	GCA_002073175.1
Protostelium mycophagum	GCA_002081555.1
Thraustotheca clavata	GCA_002081575.1
Achlya hypogyna	GCA_002081595.1
Phytophthora cactorum	GCA_002081965.1
Entodinium caudatum	GCA_002087855.2
Entodinium caudatum	GCA_002087855.3
Cryptosporidium parvum	GCA_002093595.1
Cryptosporidium parvum	GCA_002093605.1
Cryptosporidium parvum	GCA_002093615.1

Plasmodiophora brassicae	GCA_002093825.1
Peronospora tabacina	GCA_002099245.1
Acanthamoeba lenticulata	GCA_002105255.1
Plasmodium knowlesi	GCA_002140095.1
Paramoeba pemaquidensis	GCA_002151225.1
Thraustochytrium sp. ATCC 26185	GCA_002154235.1
Acanthamoeba lenticulata	GCA_002179805.1
Aphanomyces astaci	GCA_002197585.2
Phytophthora megakarya	GCA_002215365.1
Lotmaria passim	GCA_002216525.1
Crithidia mellificae	GCA_002216535.1
Crithidia mellificae	GCA_002216565.1
Crithidia bombi	GCA_002216585.1
Crithidia bombi	GCA_002216605.1
Lotmaria passim	GCA_002216625.1
Fistulifera solaris	GCA_002217885.1
Trypanosoma cruzi cruzi	GCA_002219105.2
Cryptosporidium hominis	GCA_002223825.1
Leishmania donovani	GCA_002243465.1
Proteromonas lacertae	GCA_002245135.1
Peronospora effusa	GCA_002245715.1
Peronospora effusa	GCA_002245725.1
Peronospora effusa	GCA_002245735.1
Phytophthora plurivora	GCA_002247145.1
Asterionella formosa	GCA_002256025.1
Eimeria falciformis	GCA_002271815.1
Lagenidium giganteum	GCA_002286825.1
Trypanosoma congolense	GCA_002287245.1
Phytophthora colocasiae	GCA_002288995.1
Cystoisospora suis	GCA_002600585.1
Phytophthora cinnamomi	GCA_002734105.1
Phytophthora cinnamomi	GCA_002734125.1
Trypanosoma cruzi	GCA_002749415.1
Trypanosoma cruzi	GCA_002749425.1
Ichthyophonus hoferi	GCA_002751075.1
Plasmodium vivax	GCA_002754635.1
Ichthyosporea sp. XGB-2017a	GCA_002811675.1
Abeoforma whisleri	GCA_002812265.1
Pirum gemmata	GCA_002812295.1

Phytophthora litchii	GCA_002812785.1
Plasmodium falciparum NF54	GCA_002831795.1
Nannochloropsis gaditana	GCA_002838785.1
Peronospora belbahrii	GCA_002864105.1
Chrysochromulina parva	GCA_002887195.1
Cyclospora cayetanensis	GCA_002893305.1
Cyclospora cayetanensis	GCA_002893315.1
Cyclospora cayetanensis	GCA_002893335.1
Cyclospora cayetanensis	GCA_002893365.1
Cyclospora cayetanensis	GCA_002893375.1
Cyclospora cayetanensis	GCA_002893405.1
Cyclospora cayetanensis	GCA_002893425.1
Cyclospora cayetanensis	GCA_002893445.1
Cyclospora cayetanensis	GCA_002893465.1
Cyclospora cayetanensis	GCA_002893485.1
Hondaea fermentalgiana	GCA_002897355.1
Phytophthora palmivora var. *palmivora*	GCA_002911725.1
Entamoeba moshkovskii	GCA_002914575.1
Paratrypanosoma confusum	GCA_002921335.1
Sclerospora graminicola	GCA_002933675.1
Phytophthora ramorum	GCA_002968915.1
Lagenidium giganteum	GCA_002980415.1
Paralagenidium karlingii	GCA_002980425.1
Trypanosoma congolense IL3000	GCA_003013265.1
Leishmania infantum	GCA_003020905.1
Planoprotostelium fungivorum	GCA_003024175.1
Cryptosporidium parvum	GCA_003024765.1
Cyclospora cayetanensis	GCA_003057635.1
Cryptosporidium parvum	GCA_003057855.1
Leishmania tropica	GCA_003067545.1
Aurantiochytrium sp. KH105	GCA_003116975.1
Plasmopara viticola	GCA_003123765.1
Cryptosporidium parvum	GCA_003148445.1
Trypanosoma cruzi	GCA_003177095.1
Trypanosoma cruzi	GCA_003177105.1
Phytophthora cactorum	GCA_003287315.1
Symbiodinium sp. clade A Y106	GCA_003297005.1
Symbiodinium sp. clade C Y103	GCA_003297045.1
Leishmania braziliensis	GCA_003304975.1

Naegleria lovaniensis	GCA_003324165.1
Naegleria lovaniensis	GCA_003324165.2
Phytophthora nicotianae	GCA_003328465.1
Cyclospora cayetanensis	GCA_003351235.1
Cyclospora cayetanensis	GCA_003351245.1
Cyclospora cayetanensis	GCA_003351265.1
Cyclospora cayetanensis	GCA_003351285.1
Leishmania tropica	GCA_003352575.1
Globobulimina sp.	GCA_003354225.1
Plasmodium vivax	GCA_003402195.1
Plasmodium vivax	GCA_003402215.1
Phytopythium vexans	GCA_003413675.1
Trypanosoma brucei equiperdum	GCA_003543875.1
Aphanomyces invadans	GCA_003546525.1
Aphanomyces astaci	GCA_003546545.1
Aphanomyces astaci	GCA_003546565.1
Aphanomyces astaci	GCA_003546585.1
Aphanomyces astaci	GCA_003546605.1
Aphanomyces astaci	GCA_003546625.1
Aphanomyces astaci	GCA_003546765.1
Aphanomyces astaci	GCA_003546785.1
Aphanomyces astaci	GCA_003546805.1
Aphanomyces astaci	GCA_003546825.1
Kipferlia bialata	GCA_003568945.1
Goniomonas avonlea	GCA_003573635.1
Blastocystis hominis	GCA_003575125.1
Trypanosoma cruzi	GCA_003594385.1
Trypanosoma cruzi	GCA_003594405.1
Trypanosoma cruzi	GCA_003594425.1
Trypanosoma cruzi	GCA_003594445.1
Trypanosoma cruzi	GCA_003594465.1
Trypanosoma cruzi	GCA_003594485.1
Trypanosoma cruzi	GCA_003594585.1
Trypanosoma cruzi	GCA_003594605.1
Trypanosoma cruzi	GCA_003594625.1
Trypanosoma cruzi	GCA_003594645.1
Trypanosoma cruzi	GCA_003594685.1
Trypanosoma cruzi	GCA_003594705.1
Trypanosoma cruzi	GCA_003594715.1

Dictyostelium purpureum	GCA_003640415.1
Plasmopara halstedii	GCA_003640465.1
Plasmopara obducens	GCA_003640485.1
Plasmopara halstedii	GCA_003640505.1
Plasmopara obducens	GCA_003640625.1
Leishmania lainsoni	GCA_003664395.1
Leishmania guyanensis	GCA_003664525.1
Aphanomyces astaci	GCA_003666305.1
Heterostelium multicystogenum	GCA_003667245.1
Speleostelium caveatum	GCA_003667305.1
Leishmania infantum	GCA_003671315.1
Trypanosomatidae sp. JR-2017a	GCA_003671325.1
Trypanosomatidae sp. JR-2017a	GCA_003671345.1
Plasmopara muralis	GCA_003676415.1
Polymyxa betae	GCA_003693705.1
Peronospora effusa	GCA_003704535.1
Trypanosoma cruzi	GCA_003719155.1
Trypanosoma cruzi	GCA_003719455.1
Leishmania donovani	GCA_003719575.1
Plasmopara halstedii	GCA_003724065.1
Leishmania donovani	GCA_003730175.1
Leishmania donovani	GCA_003730215.1
Pythium guiyangense	GCA_003730235.1
Plasmodiophora brassicae	GCA_003833335.1
Peronospora effusa	GCA_003843895.1
Cyclospora cayetanensis	GCA_003935065.1
Cyclospora cayetanensis	GCA_003944945.1
Cyclospora cayetanensis	GCA_003944955.1
Cyclospora cayetanensis	GCA_003944965.1
Cyclospora cayetanensis	GCA_003944975.1
Cyclospora cayetanensis	GCA_003944985.1
Cyclospora cayetanensis	GCA_003945045.1
Cyclospora cayetanensis	GCA_003945055.1
Cyclospora cayetanensis	GCA_003945065.1
Cyclospora cayetanensis	GCA_003945075.1
Cyclospora cayetanensis	GCA_003945085.1
Cyclospora cayetanensis	GCA_003945135.1
Cyclospora cayetanensis	GCA_003945145.1
Cyclospora cayetanensis	GCA_003945155.1

Cyclospora cayetanensis	GCA_003945175.1
Phytophthora ramorum	GCA_003956735.1
Pseudoperonospora humuli	GCA_003991265.1
Leishmania mexicana	GCA_003992435.1
Leishmania aethiopica	GCA_003992445.1
Leishmania amazonensis	GCA_003992505.1
Plasmodiophora brassicae	GCA_003992685.1
Globisporangium irregulare	GCA_004117815.1
Phytophthora capsici	GCA_004137865.1
Phytophthora capsici	GCA_004137885.1
Phytophthora capsici	GCA_004137955.1
Phytophthora capsici	GCA_004137965.1
Phytophthora capsici	GCA_004137975.1
Pythium insidiosum	GCA_004138015.1
Phytophthora capsici	GCA_004138045.1
Plasmodiophora brassicae	GCA_004156335.1
Plasmodiophora brassicae	GCA_004195245.1
Aurantiochytrium acetophilum	GCA_004332575.1
Nannochloropsis oceanica CCMP531	GCA_004335385.1
Nannochloropsis granulata CCMP529	GCA_004335405.1
Nannochloropsis oculata CCMP525	GCA_004335455.1
Nannochloropsis salina CCMP537	GCA_004335465.1
Halamphora sp.	GCA_004335815.1
Halamphora sp.	GCA_004335955.1
Cryptosporidium viatorum	GCA_004337795.1
Cryptosporidium ubiquitum	GCA_004337805.1
Cryptosporidium ubiquitum	GCA_004337815.1
Cryptosporidium parvum	GCA_004337825.1
Cryptosporidium cuniculus	GCA_004337835.1
Cryptosporidium parvum	GCA_004337865.1
Cryptosporidium parvum	GCA_004337875.1
Cryptosporidium meleagridis	GCA_004337925.1
Cryptosporidium parvum	GCA_004337945.1
Phytophthora ramorum	GCA_004343245.1
Cryptosporidium meleagridis	GCA_004348035.1
Bremia lactucae	GCA_004359215.1
Perkinsus sp. BL_2016	GCA_004369235.1
Plasmopara halstedii	GCA_004380875.1
Nannochloropsis oceanica	GCA_004519485.1

Nannochloropsis salina CCMP1776	GCA_004565275.1
Ectocarpus sp.	GCA_004764655.1
Schizochytrium sp. TIO01	GCA_004764695.1
Cryptosporidium sp. *chipmunk genotype I*	GCA_004936735.1
Amoebophrya sp. AT5.2	GCA_005223375.1
Leishmania amazonensis	GCA_005317125.1
Pythium oligandrum	GCA_005966545.1
Giardia muris	GCA_006247105.1
Halteria grandinella	GCA_006369765.1
Globisporangium splendens	GCA_006386115.1
Stentor roeselii	GCA_006503475.1
Diophrys appendiculata	GCA_006510565.1
Pseudokeronopsis carnea	GCA_006510595.1
Cryptosporidium tyzzeri	GCA_007210665.1
Phytophthora citricola	GCA_007655245.1
Poteriospumella lacustris	GCA_008000325.1
Poteriospumella lacustris	GCA_008000375.1
Poteriospumella lacustris	GCA_008000405.1
Phytophthora palmivora	GCA_008079305.1
Phytophthora kernoviae	GCA_008080845.1
[Pythium] brassicae (nom. inval.)	GCA_008271595.1
Cafeteria roenbergensis	GCA_008330625.1
Cafeteria roenbergensis	GCA_008330635.1
Cafeteria roenbergensis	GCA_008330645.1
Cafeteria roenbergensis	GCA_008330665.1
Naegleria fowleri	GCA_008403515.1
Phytophthora melonis	GCA_008553785.1
Sphaeroforma arctica	GCA_008580545.1
Psammoneis japonica	GCA_008632985.1
Streblomastix strix	GCA_008636045.1
Psammoneis japonica	GCA_008690995.1
Saccharina japonica	GCA_008828725.1
Nannochloropsis oceanica	GCA_009014695.1
Nannochloropsis oceanica	GCA_009014725.1
Giardia intestinalis	GCA_009192805.1
Giardia intestinalis	GCA_009192825.1
Plasmodiophora brassicae	GCA_009726225.1
Plasmodiophora brassicae	GCA_009726235.1
Plasmodiophora brassicae	GCA_009726245.1

Plasmodiophora brassicae	GCA_009726255.1
Plasmodiophora brassicae	GCA_009726265.1
Plasmodiophora brassicae	GCA_009726325.1
Plasmodiophora brassicae	GCA_009726335.1
Plasmodiophora brassicae	GCA_009726345.1
Plasmodiophora brassicae	GCA_009726355.1
Plasmodiophora brassicae	GCA_009726365.1
Plasmodiophora brassicae	GCA_009726425.1
Plasmodiophora brassicae	GCA_009726435.1
Plasmodiophora brassicae	GCA_009726445.1
Plasmodiophora brassicae	GCA_009726455.1
Plasmodiophora brassicae	GCA_009726465.1
Plasmodiophora brassicae	GCA_009726525.1
Plasmodiophora brassicae	GCA_009726535.1
Plasmodiophora brassicae	GCA_009726545.1
Plasmodiophora brassicae	GCA_009726555.1
Plasmodiophora brassicae	GCA_009726565.1
Plasmodiophora brassicae	GCA_009726625.1
Plasmodiophora brassicae	GCA_009726635.1
Plasmodiophora brassicae	GCA_009726645.1
Plasmodiophora brassicae	GCA_009726655.1
Plasmodiophora brassicae	GCA_009726665.1
Plasmodiophora brassicae	GCA_009726725.1
Plasmodiophora brassicae	GCA_009726735.1
Plasmodiophora brassicae	GCA_009726745.1
Plasmodiophora brassicae	GCA_009726755.1
Plasmodiophora brassicae	GCA_009726765.1
Plasmodiophora brassicae	GCA_009726825.1
Plasmodiophora brassicae	GCA_009726835.1
Plasmodiophora brassicae	GCA_009726845.1
Plasmodiophora brassicae	GCA_009726855.1
Plasmodiophora brassicae	GCA_009726865.1
Plasmodiophora brassicae	GCA_009726925.1
Plasmodiophora brassicae	GCA_009726935.1
Plasmodiophora brassicae	GCA_009726945.1
Plasmodiophora brassicae	GCA_009726955.1
Plasmodiophora brassicae	GCA_009726965.1
Plasmodiophora brassicae	GCA_009727025.1
Plasmodiophora brassicae	GCA_009727035.1

Plasmodiophora brassicae	GCA_009727045.1
Phytophthora fragariae	GCA_009729435.1
Phytophthora fragariae	GCA_009729455.1
Leishmania tarentolae	GCA_009731335.1
Paulinella micropora	GCA_009731375.1
Phytophthora rubi	GCA_009732905.1
Phytophthora fragariae	GCA_009732925.1
Phytophthora rubi	GCA_009732945.1
Phytophthora fragariae	GCA_009732985.1
Phytophthora fragariae	GCA_009733005.1
Phytophthora fragariae	GCA_009733025.1
Phytophthora fragariae	GCA_009733045.1
Phytophthora fragariae	GCA_009733065.1
Phytophthora fragariae	GCA_009733085.1
Phytophthora fragariae	GCA_009733105.1
Phytophthora fragariae	GCA_009733125.1
Phytophthora rubi	GCA_009733145.1
Toxoplasma gondii	GCA_009761385.1
Plasmodium falciparum	GCA_009761425.1
Plasmodium falciparum	GCA_009761475.1
Plasmodium falciparum	GCA_009761515.1
Plasmodium falciparum	GCA_009761555.1
Symbiodinium kawagutii	GCA_009767595.1
Cryptosporidium bovis	GCA_009768925.1
Leishmania tarentolae	GCA_009770625.1
Cryptosporidium ryanae	GCA_009792415.1
Aphanomyces euteiches	GCA_009835175.1
Aphanomyces stellatus	GCA_009835185.1
Aphanomyces astaci	GCA_009835205.1
Phytophthora sojae	GCA_009848525.1
Andalucia godoyi	GCA_009859145.1
Trypanosoma cruzi	GCA_010117175.1
Trypanosoma cruzi	GCA_010117215.1
Phytophthora cactorum	GCA_010194725.1
Bacillariophyta sp	GCA_010367165.1
Strombidium stylifer	GCA_010577775.1
Strombidium stylifer	GCA_010577775.2
Leishmania tropica	GCA_011316065.1
Phytophthora infestans	GCA_011316315.1

Phytophthora betacei	GCA_011320135.1
Giardia intestinalis	GCA_011634545.1
Giardia intestinalis	GCA_011634555.1
Giardia intestinalis	GCA_011634595.1
Peronospora destructor	GCA_011800735.1
Phytophthora macrochlamydospora	GCA_011947325.1
Phytophthora constricta	GCA_011947335.1
Phytophthora quininea	GCA_011947345.1
Phytophthora boehmeriae	GCA_011947355.1
Macrocystis pyrifera	GCA_012273945.1
Phytophthora infestans	GCA_012295175.1
Phytophthora chlamydospora	GCA_012295415.1
Phytophthora pseudosyringae	GCA_012295425.1
Phytophthora gonapodyides	GCA_012295475.1
Triparma laevis f. inornata	GCA_012489335.1
Phytophthora infestans	GCA_012552325.1
Phytophthora hibernalis	GCA_012656075.1
Phytophthora syringae	GCA_012656105.1
Phytophthora nicotianae	GCA_012658955.1
Undaria pinnatifida	GCA_012845835.1
Thraustochytrium aureum	GCA_012862495.1
Parietichytrium sp. I65-24A	GCA_012862575.1
Plasmodium falciparum NF54	GCA_012898025.1
Phytophthora palmivora	GCA_012932265.1
Toxoplasma gondii	GCA_013099955.1
Perkinsus olseni	GCA_013115115.1
Perkinsus olseni	GCA_013115125.1
Perkinsus olseni	GCA_013115135.1
Perkinsus chesapeaki	GCA_013115145.1
Perkinsus olseni	GCA_013115865.1
Perkinsus olseni	GCA_013115895.1
Peronospora effusa	GCA_013122855.1
Cyclotella cryptica	GCA_013187285.1
Thraustochytrium sp. TN22	GCA_013306675.1
Trypanosoma cruzi	GCA_013358655.1
Dictyostelium rosarium	GCA_013375675.1
Dictyostelium brefeldianum	GCA_013375695.1
Pseudokeronopsis flava	GCA_013420745.1
Hondaea fermentalgiana	GCA_014084085.1

Leishmania tropica	GCA_014139745.1
Leishmania chagasi	GCA_014466935.1
Leishmania chagasi	GCA_014466975.1
Schmidingerella arcuata	GCA_014524975.1
Cryptosporidium felis	GCA_014529505.1
Phytophthora quercina	GCA_014706105.1
Phytophthora castanetorum	GCA_014706115.1
Phytophthora ohioensis (nom. inval.)	GCA_014706125.1
Phytophthora tubulina	GCA_014706135.1
Phytophthora sp. ST_20190627	GCA_014706145.1
Phytophthora versiformis	GCA_014706215.1
Naegleria fowleri	GCA_014843625.1
Plasmodium vivax	GCA_014843675.1
Plasmodium vivax	GCA_014843685.1
Plasmodium vivax	GCA_014843935.1
Plasmodium vivax	GCA_014843945.1
Fucus vesiculosus	GCA_014849475.1
Asterionellopsis glacialis	GCA_014885115.2
Toxoplasma gondii	GCA_014898695.1
Trypanosoma cruzi	GCA_015033625.1
Trypanosoma cruzi	GCA_015033655.1
Entamoeba histolytica	GCA_015099795.1
Mallomonas annulata	GCA_015143345.1
Pedospumella encystans	GCA_015143685.1
Dinobryon sp. LO226KS	GCA_015143705.1
Synura sp. LO234KE	GCA_015143735.1
Chromulinospumella sphaerica	GCA_015144965.1
Epipyxis sp. PR26KG	GCA_015144975.1
Poterioochromonas sp. DS	GCA_015145745.1
Dinobryon divergens	GCA_015146095.1
Spumella sp. AR4D6	GCA_015146115.1
Ochromonas danica	GCA_015146655.1
Spumella vulgaris	GCA_015148565.1
Pedospumella encystans	GCA_015148585.1
Chromulina nebulosa	GCA_015148605.1
Labyrinthula sp. Ha	GCA_015227615.1
Cryptosporidium parvum	GCA_015245375.1
Dictyostelium capitatum	GCA_015252635.1
Dictyostelium gargantuum	GCA_015252675.1

Trypanosoma cruzi	GCA_015455285.1
Cardiosporidium cionae	GCA_015476325.1
Neospora caninum	GCA_016097395.1
Neospora caninum	GCA_016097425.1
Phytophthora vignae	GCA_016169925.1
Phytophthora vignae f. sp. *adzukicola*	GCA_016169935.1
Phytophthora vignae f. sp. *vignae*	GCA_016169955.1
Tetrahymena thermophila	GCA_016584475.1
Nitzschia putrida	GCA_016586335.1
Neovahlkampfia damariscottae	GCA_016618085.1
Phytophthora capsici	GCA_016618375.1
Trypanosomatidae sp. JR-2017a	GCA_016642125.1
Trypanosomatidae sp. JR-2017a	GCA_016642145.1
Trypanosomatidae sp. JR-2017a	GCA_016642365.1
Trypanosomatidae sp. JR-2017b	GCA_016643115.1
Paramecium bursaria	GCA_016759035.1
Paramecium bursaria	GCA_016759055.1
Neospora caninum	GCA_016801225.1
Paramecium bursaria	GCA_016802755.1
Paramecium bursaria	GCA_016802775.1
Toxoplasma gondii	GCA_016807865.1
Toxoplasma gondii	GCA_016807875.1
Toxoplasma gondii	GCA_016807885.1
Paramecium bursaria	GCA_016807985.1
Paramecium bursaria	GCA_016808005.1
Paramecium bursaria	GCA_016808045.1
Paramecium bursaria	GCA_016808065.1
Toxoplasma gondii	GCA_016808245.1
Phytophthora cactorum	GCA_016864635.1
Phytophthora cactorum	GCA_016864655.1
Phytophthora cactorum	GCA_016864665.1
Phytophthora cactorum	GCA_016864695.1
Phytophthora cactorum	GCA_016864715.1
Phytophthora cactorum	GCA_016864735.1
Phytophthora cactorum	GCA_016864755.1
Phytophthora cactorum	GCA_016864775.1
Phytophthora cactorum	GCA_016864795.1
Phytophthora cactorum	GCA_016864815.1
Phytophthora cactorum	GCA_016864835.1

Phytophthora cactorum	GCA_016864855.1
Phytophthora cactorum	GCA_016865055.1
Phytophthora cactorum	GCA_016865075.1
Phytophthora cactorum	GCA_016865085.1
Phytophthora idaei	GCA_016865095.1
Phytophthora idaei	GCA_016880175.1
Phytophthora idaei	GCA_016880985.1
Phytophthora cactorum	GCA_016906365.1
Phytophthora cactorum	GCA_017309695.1
Tribonema minus	GCA_017506865.1
Skeletonema costatum	GCA_018806925.1
Nitzschia inconspicua	GCA_019154785.2
Nitzschia palea	GCA_019593585.1
Fistulifera pelliculosa	GCA_019693425.1
Cylindrotheca fusiformis	GCA_019693525.1
Chaetoceros muellerii	GCA_019693545.1
Thalassiosira oceanica	GCA_019693575.1
Thalassiosira sundarbana	GCA_020086505.1
Plasmodium chabaudi chabaudi	GCA_900002335.1
Plasmodium yoelii	GCA_900002395.1
Plasmodium knowlesi strain H	GCA_900004885.2
Plasmodium knowlesi strain H	GCA_900005025.2
Plasmodium berghei	GCA_900044335.1
Giardia intestinalis	GCA_900069105.1
Plasmodium berghei	GCA_900088445.1
Hyphochytrium catenoides	GCA_900088475.1
Plasmodium ovale wallikeri	GCA_900088485.1
Plasmodium ovale wallikeri	GCA_900088545.1
Plasmodium ovale curtisi	GCA_900088555.1
Plasmodium ovale curtisi	GCA_900088565.1
Plasmodium ovale	GCA_900090025.2
Plasmodium ovale	GCA_900090035.2
Rostrostelium ellipticum	GCA_900092235.1
Acytostelium leptosomum	GCA_900092245.1
Synstelium polycarpum	GCA_900092255.1
Coremiostelium polycephalum	GCA_900092265.1
Cavenderia deminutiva	GCA_900092275.1
Plasmodium vivax	GCA_900093535.1
Plasmodium vivax	GCA_900093545.1

Plasmodium vivax	GCA_900093555.2
Fragilariopsis cylindrus	GCA_900095095.1
Plasmodium berghei	GCA_900095545.1
Plasmodium chabaudi chabaudi	GCA_900095555.1
Plasmodium chabaudi adami	GCA_900095565.1
Plasmodium berghei	GCA_900095585.1
Plasmodium sp. *gorilla* clade G1	GCA_900095595.1
Plasmodium chabaudi chabaudi	GCA_900095605.1
Plasmodium chabaudi adami	GCA_900095625.1
Plasmodium berghei	GCA_900095635.1
Plasmodium reichenowi	GCA_900097025.1
Plasmodium sp. *gorilla* clade G3	GCA_900097035.1
Plasmodium knowlesi	GCA_900162085.1
Plasmodium vivax	GCA_900178095.1
Plasmodium cynomolgi	GCA_900180395.1
Plasmodium sp. DRC-Itaito	GCA_900240055.1
Crithidia expoeki	GCA_900240875.1
Crithidia bombi	GCA_900240985.1
Aphanomyces stellatus	GCA_900243725.1
Aphanomyces astaci	GCA_900243735.1
Trypanosoma cruzi	GCA_900252365.1
Plasmodium sp. DRC-Itaito	GCA_900257145.2
Licmophora abbreviata	GCA_900291995.1
Plasmodiophora brassicae	GCA_900303365.2
Aphanomyces euteiches	GCA_900312765.1
Spongospora subterranea	GCA_900404475.1
Trypanosoma brucei brucei	GCA_900497135.1
Leishmania infantum	GCA_900500625.2
Leishmania braziliensis MHOM/BR/75/M2904	GCA_900537975.1
Hydrurus foetidus	GCA_900617105.1
Plasmodium falciparum	GCA_900617135.1
Plasmodium vinckei petteri	GCA_900617785.1
Plasmodium vinckei vinckei	GCA_900617805.1
Plasmodium vinckei brucechwatti	GCA_900617815.1
Plasmodium yoelii killicki	GCA_900617825.1
Plasmodium yoelii killicki	GCA_900617835.1
Plasmodium yoelii	GCA_900617845.1
Plasmodium yoelii	GCA_900617855.1

Plasmodium yoelii	GCA_900617865.1
Plasmodium yoelii	GCA_900617875.1
Plasmodium yoelii	GCA_900617885.1
Plasmodium falciparum	GCA_900631975.1
Plasmodium falciparum	GCA_900631985.1
Plasmodium falciparum	GCA_900631995.1
Plasmodium falciparum	GCA_900632005.1
Plasmodium falciparum	GCA_900632015.1
Plasmodium falciparum	GCA_900632025.1
Plasmodium falciparum	GCA_900632035.1
Plasmodium falciparum	GCA_900632045.1
Plasmodium falciparum	GCA_900632055.1
Plasmodium falciparum	GCA_900632065.1
Plasmodium falciparum	GCA_900632075.1
Plasmodium falciparum	GCA_900632085.1
Plasmodium falciparum	GCA_900632095.1
Leishmania donovani	GCA_900635355.2
Fragilaria radians	GCA_900642245.1
Pseudo-nitzschia multistriata	GCA_900660405.1
Aphanomyces stellatus	GCA_900708865.1
Euglena gracilis	GCA_900893395.1
Giardia intestinalis	GCA_902209425.1
Giardia intestinalis	GCA_902221465.1
Giardia intestinalis	GCA_902221485.1
Giardia intestinalis	GCA_902221515.1
Giardia intestinalis	GCA_902221535.1
Giardia intestinalis	GCA_902221545.1
Leishmania braziliensis	GCA_902369275.1
Leishmania adleri	GCA_902369305.1
Leishmania guyanensis	GCA_902369315.1
Leishmania infantum	GCA_902369335.1
Leishmania major strain Friedlin	GCA_902369385.1
Hepatocystis sp. *ex Piliocolobus tephrosceles*	GCA_902459845.2
Leishmania major strain Friedlin	GCA_902498725.1
Ectocarpus sp.	GCA_902602495.3
Mastigamoeba balamuthi	GCA_902651635.1
Naegleria fowleri	GCA_902703645.1
Albugo laibachii	GCA_902706625.1
Peronospora belbahrii	GCA_902712285.1

Acanthamoeba triangularis	GCA_902749335.1
Willaertia magna	GCA_902984215.1
Acanthamoeba castellanii	GCA_903821525.1
Plasmodium vinckei brucechwatti	GCA_903994205.1
Plasmodium vinckei lentum	GCA_903994225.1
Plasmodium vinckei petteri	GCA_903994235.1
Plasmodium vinckei	GCA_903994265.1
Albugo candida	GCA_905220665.1
Symbiodinium natans	GCA_905221605.1
Symbiodinium sp. CCMP2592	GCA_905221615.1
Symbiodinium sp. KB8	GCA_905221625.1
Symbiodinium sp. CCMP2456	GCA_905221635.1
Symbiodinium pilosum	GCA_905231905.1
Symbiodinium necroappetens	GCA_905231915.1
Symbiodinium microadriaticum	GCA_905231925.1
Polarella glacialis	GCA_905237085.1
Polarella glacialis	GCA_905237095.1
Eimeria tenella	GCA_905310635.1
Plasmodium vivax	GCF_000002415.2
Giardia intestinalis	GCF_000002435.2
Leishmania major strain Friedlin	GCF_000002725.2
Plasmodium falciparum	GCF_000002765.5
Leishmania braziliensis MHOM/BR/75/M2904	GCF_000002845.2
Monosiga brevicollis	GCF_000002865.3
Leishmania infantum JPCM5	GCF_000002875.2
Dictyostelium discoideum AX4	GCF_000004695.1
Heterostelium album PN500	GCF_000004825.1
Naegleria gruberi	GCF_000004985.1
Plasmodium knowlesi	GCF_000006355.1
Perkinsus marinus ATCC 50983	GCF_000006405.1
Cryptosporidium hominis TU502	GCF_000006425.1
Cryptosporidium muris RN66	GCF_000006515.1
Toxoplasma gondii ME49	GCF_000006565.2
Paramecium tetraurelia	GCF_000141845.1
Thecamonas trahens ATCC 50062	GCF_000142905.1
Phytophthora infestans T30-4	GCF_000142945.1
Thalassiosira pseudonana	GCF_000149405.2
Phytophthora sojae	GCF_000149755.1

Phaeodactylum tricornutum	GCF_000150955.2
Capsaspora owczarzaki ATCC 30864	GCF_000151315.2
Saprolegnia parasitica	GCF_000151545.1
Blastocystis hominis	GCF_000151665.1
Cryptosporidium parvum Iowa II	GCF_000165345.1
Paramecium tetraurelia strain d4-2	GCF_000165425.1
Aureococcus anophagefferens	GCF_000186865.1
Salpingoeca rosetta	GCF_000188695.1
Tetrahymena thermophila SB210	GCF_000189635.1
Dictyostelium purpureum	GCF_000190715.1
Cavenderia fasciculata	GCF_000203815.1
Neospora caninum	GCF_000208865.1
Entamoeba histolytica	GCF_000208925.1
Trypanosoma cruzi strain CL Brener	GCF_000209065.1
Entamoeba dispar	GCF_000209125.1
Trypanosoma brucei gambiense DAL972	GCF_000210295.1
Ichthyophthirius multifiliis	GCF_000220395.1
Gregarina niphandrodes	GCF_000223845.1
Leishmania donovani	GCF_000227135.1
Leishmania mexicana	GCF_000234665.1
Nannochloropsis gaditana CCMP526	GCF_000240725.1
Phytophthora parasitica	GCF_000247585.1
Entamoeba nuttalli P19	GCF_000257125.1
Saprolegnia diclina VS20	GCF_000281045.1
Acanthamoeba castellanii str. Neff	GCF_000313135.1
Guillardia theta	GCF_000315625.1
Entamoeba invadens IP1	GCF_000330505.1
Emiliania huxleyi CCMP1516	GCF_000372725.1
Fonticula alba	GCF_000388065.1
Eimeria necatrix	GCF_000499385.1
Eimeria acervulina	GCF_000499425.1
Eimeria tenella	GCF_000499545.2
Eimeria maxima	GCF_000499605.1
Eimeria mitis	GCF_000499745.2
Aphanomyces astaci	GCF_000520075.1
Aphanomyces invadans	GCF_000520115.1
Plasmodium inui San Antonio 1	GCF_000524495.1
Trypanosoma grayi	GCF_000691245.1
Blastocystis sp. subtype 4	GCF_000743755.1

Leishmania panamensis	GCF_000755165.1
Acytostelium subglobosum	GCF_000787575.1
Plasmodium fragile	GCF_000956335.1
Sphaeroforma arctica JP610	GCF_001186125.1
Leptomonas pyrrhocoris	GCF_001293395.1
Plasmodium reichenowi	GCF_001601855.1
Plasmodium gaboni	GCF_001602025.1
Plasmodium coatneyi	GCF_001680005.1
Cryptosporidium ubiquitum	GCF_001865345.1
Trypanosoma theileri	GCF_002087225.1
Plasmodium gonderi	GCF_002157705.1
Besnoitia besnoiti	GCF_002563875.1
Cyclospora cayetanensis	GCF_002999335.1
Trypanosoma rangeli	GCF_003719475.1
Trypanosoma conorhini	GCF_003719485.1
Plasmopara halstedii	GCF_900000015.1
Plasmodium chabaudi	GCF_900002335.2
Plasmodium berghei ANKA	GCF_900002375.2
Plasmodium yoelii	GCF_900002385.2
Plasmodium relictum	GCF_900005765.1
Plasmodium gallinaceum	GCF_900005855.1
Plasmodium sp. *gorilla* clade G2	GCF_900097015.1
Plasmodium vinckei	GCF_900681995.1

Table S2. Phylogeny congruence test for plant mitochondrial linear plasmids

Event Costs[a]	Total Cost	No. of Events					*P* value[b]
		Cospeciation	Duplication	Duplication and Host Switching	Loss	Failure to Diverge	
0,1,2,1,1	348	2	15	8	278	39	$P > 0.05$
0,1,1,2,0	582	1	5	19	279	39	$P > 0.05$
-1,0,0,0,0	-3	3	5	17	291	39	$P > 0.05$

Table S3. Information on fungus and plant genomes with the presence of mitochondrial linear plasmids

Host	Gene ID	Gene location
Actinidia rufa	BJWL01000049.1	3889-21323
Camelina sativa	CM002730.1	5815539-5848762
Triticum dicoccoides	CM007933.2	166415928-166445829
Triticum urartu	CM009799.1	213262428-213292606
Punica granatum	CM018618.1	11740587-11747674
Primulina huaijiensis	CM022693.1	10849788-10876056
Primulina huaijiensis	CM022696.1	9903435-9930364
Chrysopogon serrulatus	JADLZL010001976.1	696195-706929
Dorcoceras hygrometricum	KV020259.1	1-7034
Aquilegia coerulea	KZ305023.1	1606963-1613537
Lactuca saligna	LR794333.1	363773966-363788128
Triticum urartu	MKGO01009592.1	19598-27368
Liriodendron chinense	ML058429.1	7016647-7026093
Cucurbita maxima	NEWN01000485.1	71-17754
Berberis thunbergii	QNQO01000768.1	10512-22826
Puccinellia tenuiflora	QRDG01001842.1	64496-88028
Cocos nucifera	QRFJ01003360.1	88713-94596
Dactylis glomerata	QXEO01000555.1	4208113-4237827
Abrus precatorius	QYUI01000001.1	919072-929183
Glycine soja	QZWG01001099.1	60-35575
Glycine soja	QZWG01001110.1	4999-14381
Glycine soja	QZWG01001112.1	8048-17264
Actinidia chinensis	VIGQ01001125.1	500865-535908
Litsea cubeba	WHSA01000238.1	102373-126044
Apium graveolens	WRXP01002374.1	24659-37044
mitochondrion *Daucus carota* subsp. *sativus*	LNRQ01000010.1	234953-244979
mitochondrion *Prunus mira*	NC_065231.1	176101-192456
mitochondrion *Solanum melongena*	NC_050334.1	395172-402238
mitochondrion *Lactuca saligna*	MZ159956.1	353525-368269
mitochondrion *Acer yangbiense*	CM017774.1	425964-455933
mitochondrion *Nicotiana tabacum*	NC_006581.1	101253-113523
mitochondrion *Brassica napus*	NC_004946.1	5-11636
mitochondrion *Dysphania ambrosioides*	CM048684.1	201146-218536

mitochondrion *Cocos nucifera*	NC_031696.1	64531-70414
mitochondrion *Silene latifolia*	NC_014487.1	24181-56684
Hypoxylon rickii	CADCWY010000027.1	4918-23979
Pyrenopolyporus hunteri	CADCXC010000024.1	1-8640
Myriosclerotinia sulcatula	CADDYZ010000002.1	19077-42325
Mortierella sp. AM989	JAAAUM010000950.1	2086-10200
Tulosesus angulatus	JAACJK010000214.1	1503-14301
Leucoagaricus leucothites	JAACJO010000066.1	8107-29895
Apophysomyces sp. BC1034	JAAZWW010000050.1	52366-73192
Termitomyces sp. Mn162	JABAJY010008481.1	10-8742
Mycena chlorophos	JACAZE010000064.1	4-12121
Mycena kentingensis	JACAZG010000057.1	15-11731
Pleurotus tuber-regium	JACFYU010000033.1	3-12115
Pseudocercospora ulei	JACWNB010000062.1	76296-105181
Sphaerulina populicola P02.02b	JH792543.1	43031-71956
Fusarium langsethiae	JXCE01000687.1	1-8869
Melampsora pinitorqua Mpini7	KE730102.1	1273-9744
Mycosphaerella populi pn0402	KZ119071.1	3-8768
Epichloe sylvatica	LCTT01001189.1	18-6514
Chrysoporthe cubensis	LJCY02000041.1	13-8749
Diaporthe aspalathi	LJJS01001734.1	2-9500
Tilletia controversa	LWDE02001218.1	1-7556
Smittium simulii	MBFR01000812.1	2-6751
Trametes polyzona	MKKQ01003640.1	9-9175
Trametes pubescens	MNAD01001656.1	49518-59486
Phlegmacium glaucopus	MU130443.1	30-140844
Juglanconis oblonga	MUZH01002850.1	3-5994
Scheffersomyces stambukii	NBZC01000117.1	2-30230
Ceratocystis smalleyi	NETT01000797.1	1-6989
Lobosporangium transversale	NW_019029103.1	3-7306
Postia placenta MAD-698-R-SB12	NW_020169878.1	1904-10595
Alternaria brassicicola	PHFN01000012.1	64529-72260
Pichia heedii	PPIR02000135.1	6-7057
Botrytis tulipae	PQXH01000439.1	1-10404
Venturia inaequalis	QWWT01000031.1	31595-55458
Neurospora sp. FGSC 26632	RAOH01001087.1	2-6015
Jimgerdemannia flammicorona	RBNI01006129.1	1806-10287
Tilletia laevis	RDSF01001239.1	1-7044

Pleurotus pulmonarius	SJKF01000022.1	9055-24171
Hypomyces perniciosus	SPDT01000004.1	252388-258263
Hericium erinaceus	SZZO02000021.1	21686-51214
Hericium erinaceus	SZZO02000022.1	2-27235
Hericium erinaceus	SZZO02000023.1	22163-40084
Hericium erinaceus	SZZO02000029.1	9074-25862
Hericium erinaceus	SZZO02000030.1	4-5687
Mortierella sp. BCC40632	WAEK01000063.1	1-8565
Rhodofomes roseus	SEKV01001066.1	52-8833
mitochondrion *Lecanosticta acicola*	NXEF01003541.1	16593-42555
mitochondrion *Pyrenophora tritici-repentis*	CM025805.1	84231-116448
mitochondrion *Pyrenophora tritici-repentis*	CM031751.1	87258-119449
mitochondrion *Marasmius oreades*	CM032192.1	50662-65528
mitochondrion *Agaricus subrufescens*	CM057070.1	4886-40298
mitochondrion *Taiwanofungus camphoratus*	CP021234.1	136383-140860
mitochondrion *Venturia effusa*	CP042205.1	86269-92416
mitochondrion *Ustilaginoidea virens*	CP049932.1	9741-18356
mitochondrion *Epichloe elymi*	CP098280.1	14089-23140
mitochondrion *Epichloe amarillans*	CP099640.1	17323-24802
mitochondrion *Sclerotinia sclerotiorum* 1980 UF-70	KT283062.1	98251-127034
mitochondrion *Inonotus obliquus*	LC497415.1	89818-94601
mitochondrion *Ustilaginoidea virens*	NW_025064823.1	9740-18351
mitochondrion *Marasmius oreades*	NW_025064824.1	50662-65528
mitochondrion *Pleurotus ostreatus*	OX344748.1	1-9313
